# Structure–Activity Optimization of Phenoxy-1,2-dioxetane Precursors as Probes for Singlet Oxygen Yields Unprecedented Detection Sensitivity

**DOI:** 10.1021/jacsau.5c00465

**Published:** 2025-05-23

**Authors:** Rozan Tannous, Tal Kopp, Doron Shabat

**Affiliations:** † School of Chemistry, Raymond and Beverly Sackler Faculty of Exact Sciences, 26745 Tel-Aviv University, Tel Aviv 69978, Israel

**Keywords:** chemiluminescence, 1,2-dioxetane precursors, structure−activity relationship, singlet oxygen, enzyme-mediated oxidation

## Abstract

Chemiluminescence imaging has emerged as a powerful alternative to fluorescence-based methods, offering significant advantages such as reduced background noise, elimination of autofluorescence, and prevention of photobleaching. These benefits are particularly critical for singlet oxygen detection, where the excitation light in fluorescence techniques can inadvertently generate singlet oxygen, compromising measurement accuracy. Despite this potential, the development of highly sensitive chemiluminescent probes for singlet oxygen detection under physiological conditions remains an urgent challenge. Here, we present a comprehensive structure–activity optimization of phenoxy-1,2-dioxetane precursors as probes for singlet oxygen detection in physiological environments. By systematically evaluating key parameters—steric hindrance at the oxidation site, the chemiexcitation rate of the luminophore, and total light emission—we significantly increased the detection sensitivity of the singlet oxygen probe. Notably, a cyclobutyl-enolether probe (SOCL-CB) and a dimethyl-enolether probe (SOCL-DM) demonstrated 57-fold and 118-fold higher signal-to-noise (S/N) ratios, respectively, compared to the previously reported chemiluminescent adamantyl-enolether probe (SOCL-AD). The superior detection sensitivity of probe SOCL-DM was validated in an enzymatic model where singlet oxygen production was mediated by horseradish peroxidase. Remarkably, probe SOCL-DM detected singlet oxygen concentrations as low as 127 nM in this system, outperforming the previously reported probe SOCL-AD. These results establish probe SOCL-DM as the most sensitive chemiluminescent probe for singlet oxygen detection under physiological conditions reported to date. This study underscores the potential of chemiluminescent probes like SOCL-DM to facilitate real-time monitoring of singlet oxygen, providing invaluable tools for studying oxidative stress, elucidating cellular processes, and advancing diagnostic applications.

## Graphical abstract

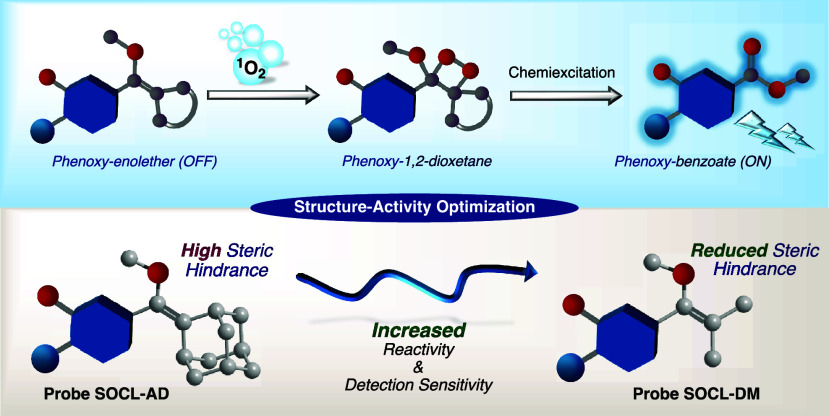

## Introduction

Reactive oxygen species (ROS), particularly singlet oxygen (^1^O_2_), play a crucial role in cell signaling,^1^ stress responses,^2^ and various physiological processes,^3^ such as immune defense,^4^ gene expression,^5^ and regulation of mitochondrial membrane permeability.^6^ In the context of photodynamic therapy (PDT) for cancer, ^1^O_2_ acts as a key cytotoxic agent generated by photosensitizers to selectively kill tumor cells upon light activation.^7–11^ Therefore, real-time monitoring of ^1^O_2_ levels under physiologically relevant conditions is essential for understanding its biological functions and optimizing therapeutic applications. While ^1^O_2_ can be detected through its weak phosphorescence at 1270 nm,^12^ the low quantum yield and poor signal-to-noise ratio in aqueous environments significantly limit its utility for biological applications.^13^ As an alternative, reaction-based fluorescent probes have gained popularity due to their sensitivity and applicability in cellular imaging.^14–19^ However, these probes present a notable drawback: the requirement for external light excitation can lead to unwanted ^1^O_2_ generation, introducing phototoxicity and background interference.^20^

Chemiluminescence imaging has emerged as a superior approach, offering significant advantages over fluorescence methods.^21–23^ By eliminating the need for light irradiation, chemiluminescent probes reduce background noise, autofluorescence, and photobleaching.^24^ This makes them particularly advantageous for detecting ^1^O_2_, as they avoid the complications introduced by light-induced ^1^O_2_ generation. Thus, the development of highly sensitive chemiluminescent probes for ^1^O_2_ detection under physiological conditions remains a pressing need.

In 2017, our group explored new approaches for amplifying chemiluminescence light intensity under physiological conditions.^25^ A remarkable enhancement of light emission was obtained by simply improving the emissive nature of the excited species, formed during the chemiexcitation of unsubstituted phenoxy-dioxetanes. Phenoxy-dioxetane probes, bearing conjugated electron-withdrawing substituents at their *ortho* position, release a benzoate derivative, during their chemiexcitation, which is highly emissive under aqueous conditions. These new phenoxy-dioxetane luminophores exhibited light emission intensity up to 3000-fold greater than that of its original parent dioxetane.^26–34^

Shortly after, we took advantage of this discovery to develop a new, efficient chemiluminescence probe (SOCL-AD), for the detection and imaging of ^1^O_2_.^35^ The probe reacts with ^1^O_2_ to form a dioxetane intermediate, which spontaneously decomposes under physiological conditions through a chemiexcitation pathway, emitting green light with extraordinary intensity. Probe SOCL-AD demonstrated high selectivity and sensitivity toward ^1^O_2_. Additionally, a cell-permeable version of the probe showed a promising ability to detect and image intracellular ^1^O_2_ produced by a photosensitizer in tumoral cells during the PDT mode of action.

The general activation pathway of our singlet oxygen chemiluminescent probes bearing an electron-withdrawing group at the *ortho* position of their phenol is depicted in Figure 1A. Upon reaction with singlet oxygen, the enolether unit of the probe is oxidized via a [2 + 2] cycloaddition mechanism, to form the corresponding phenol-1,2-dioxetane luminophore.^36^ This luminophore undergoes a spontaneous chemiexcitation upon generation of its phenolate ion in water, resulting in the formation of an excited benzoate ester. Subsequently, the decay of the latter to its ground state is accompanied by the emission of visible light.

**Figure 1:**
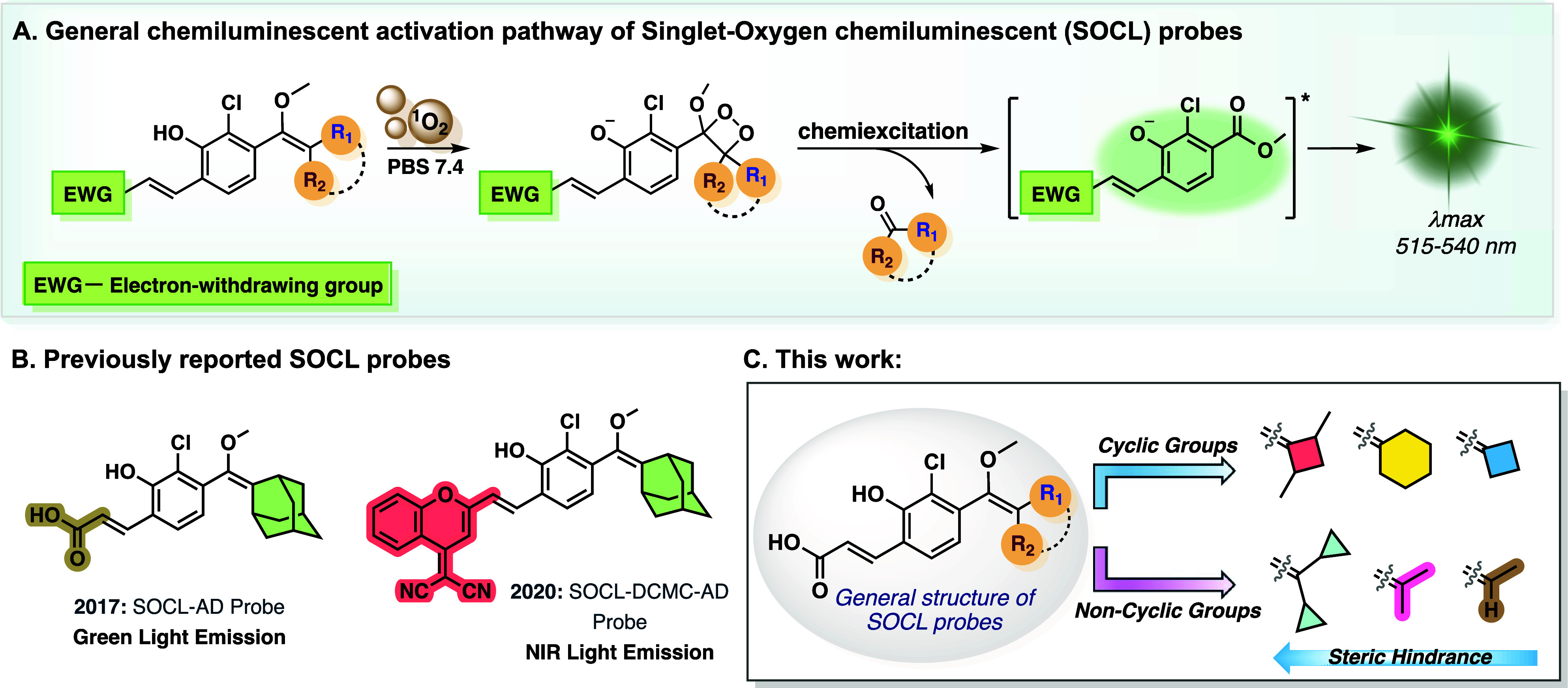
(A) Activation and chemiexcitation pathway of Singlet-oxygen chemiluminescent (SOCL) probes upon reaction with ^1^O_2_. (B) General structure and characteristics of previously reported adamantyl-based SOCL probes. (C) This work: Structure–Activity optimization of SOCL probes.

Figure 1B shows the molecular structures of two phenoxy-enolether-based chemiluminescent probes previously developed by our group for the detection of singlet oxygen. The first probe (SOCL-AD) features an *ortho*-substituted acrylic acid group and emits green light,^35^ while the second incorporates an *ortho*-substituted dicyanomethylene-4H-chromene (DCMC) group, resulting in near-infrared (NIR) light emission.^37^ Probe SOCL-AD is one of the most efficient chemiluminescence probes for detecting ^1^O_2_, known to date. Therefore, we hypothesized that exchanging the rigid and bulky adamantyl unit in probe SOCL-AD with more compact moieties that differ in their chemical and electronic properties, could lead to improved activities upon the reaction with ^1^O_2_.

Here we report the structure–activity optimization of phenoxy-1,2-dioxetane precursors as probes for ^1^O_2_ detection in physiological environments. Our optimized probes demonstrate significant improvements in detection limit, offering a powerful tool for real-time monitoring of ^1^O_2_ in physiological conditions (Figure 1C).

## Results and Discussion

The molecular design of our SOCL probes (Figure 2A) features an enolether skeleton with an acrylic acid group serving as the electron-withdrawing group. This substituent was selected for its ability to enhance water solubility and to improve the emissive properties of the excited intermediate formed during the chemiexcitation of the corresponding dioxetane in aqueous conditions. In addition, these probes feature various R_1_ and R_2_ substituents, that play a pivotal role in modulating the chemiluminescent response. In order to explore the structure–activity relationship of the R_1_ and R_2_ substituents on the ability to detect ^1^O_2_, we designed and synthesized a series of new phenoxy-enolethers (1,2-dioxetane precursors) with varying steric bulk, ring strain, and electronic properties. These SOCL probes incorporate R_1_ and R_2_ groups, which can exist as separate entities or be interconnected to form a cyclic structure. Inspired by our previous findings that demonstrated an enhanced chemiexcitation rate and quantum yields through the introduction of different cyclic moieties,^38,39^ we synthesized a six-membered ring probe (SOCL-CH) and several strained four-membered ring probes (SOCL-CB, SOCL-DM-CB, SOCL-Ph-CB, SOCL-OBn-CB, and SOCL-Ox), each possessing different steric and electronic characteristics. To assess the influence of noncyclic substituents, we synthesized three probes featuring groups with varying degrees of steric hindrance. Probe SOCL-DCP incorporates a highly hindered dicyclopropyl substituent, while probe SOCL-DM features a less hindered dimethyl substituent, and probe SOCL-MM includes the least hindered monomethyl group. Additionally, the known adamantyl-enolether, probe SOCL-AD, was used as a reference compound.

**Figure 2:**
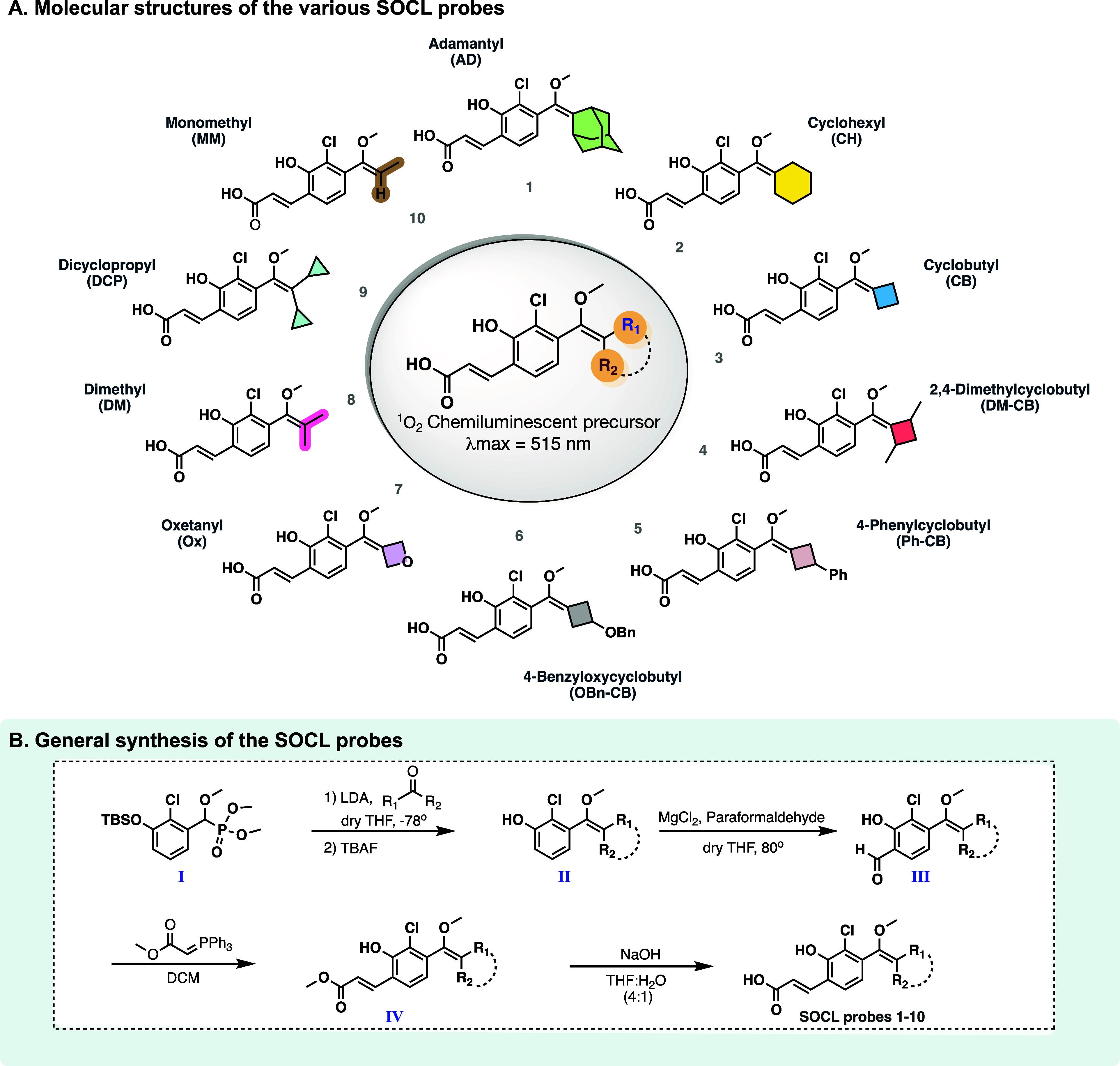
(A) Molecular structures of the various SOCL probes. (B) General synthetic pathway for the preparation of the ^1^O_2_ chemiluminescent precursors.

The synthesis of the SOCL probes followed a general synthetic route, as described in Figure 2B. Phosphonate **I** was first reacted via a Horner-Wittig reaction with the relevant ketone, followed by TBS deprotection to form phenol enolether **II**. The latter was then treated with magnesium chloride and paraformaldehyde to produce salicylic aldehyde **III**. Wittig reaction of salicylic aldehyde **III** with methyl-(triphenylphosphoranylidene)-acetate afforded methyl acrylate enolether **IV**. Finally, the methyl ester group of enolether **IV** was hydrolyzed using aqueous sodium hydroxide to yield the desired SOCL probes (detailed synthesis and characterization are presented in Supporting Information S4–S17).

We initially sought to evaluate the chemiluminescent response of SOCL probes to singlet oxygen by incubation with 3-(1,4-dihydro-1,4-epidioxy-4-methyl-1-naphthyl)-propionic acid (EP-1). The latter is a known water-soluble compound that serves as a singlet oxygen donor through thermal decomposition.^40^ The chemiluminescence kinetic profiles of the SOCL probes in PBS, pH 7.4 are presented in Figure 3A. Upon incubation with EP-1, the probes exhibited a typical chemiluminescence kinetic profile, with an initial light-emission increase to a maximum, followed by the gradual decay of the signal over 200 min. In contrast, negligible chemiluminescence was observed in the absence of EP-1 (see Figures S1 and S2). All SOCL probes followed the same kinetic pattern, which reflects the decomposition kinetics of EP-1 under the measurement conditions. The intensity of the chemiluminescence signals of the SOCL probes was determined by measuring the total light emitted by each probe upon activation with EP-1 (Figure 3B). Notably, six out of nine SOCL probes bearing *cyclohexyl* (SOCL-CH), *benzyloxy-cyclobutyl* (SOCL-OBn-CB), *dicyclopropyl* (SOCL-DCP), *phenyl-cyclobutyl* (SOCL-Ph-CB), *dimethyl* (SOCL-DM), and *cyclobutyl* (SOCL-CB) motifs exhibited significant enhancement in the chemiluminescent signals compared to the parent adamantyl- probe SOCL-AD. This enhancement is likely attributed to a decrease in steric hindrance in the vicinity of the enol-ether unit, which increases its accessibility for singlet oxygen trapping, thereby improving oxidation efficiency. Among these, *cyclobutyl* (SOCL-CB) and *dimethyl* (SOCL-DM) enolethers, featuring the least sterically hindered moieties, exhibited the highest signals with 14-fold and 12-fold enhancement compared to their adamantyl counterpart (probe SOCL-AD), respectively. Probes with larger steric hindrance, such as *dicyclopropyl* (SOCL-DCP) and *cyclohexyl* (SOCL-CH), exhibit 8-fold and 5-fold higher chemiluminescence signals compared to probe SOCL-AD, respectively. The substituted *cyclobutyl* probes, including *phenyl-cyclobutyl* (SOCL-Ph-CB) and *benzyloxy-cyclobutyl* (SOCL-OBn-CB), also demonstrate enhanced chemiluminescence signals, with increases of 8-fold and 5-fold, respectively, in comparison to probe SOCL-AD. Nevertheless, these probes produced weaker signals compared to the unsubstituted cyclobutyl, probe SOCL-CB. The reduced signal intensity of the substituted cyclobutyl probes is likely a result of the higher electronegativity of the substituents, which reduces the electron cloud density of the enolether unit. This electron deficiency results in decreased reactivity of the probes toward singlet oxygen, thereby reducing their overall chemiluminescent response.

**Figure 3:**
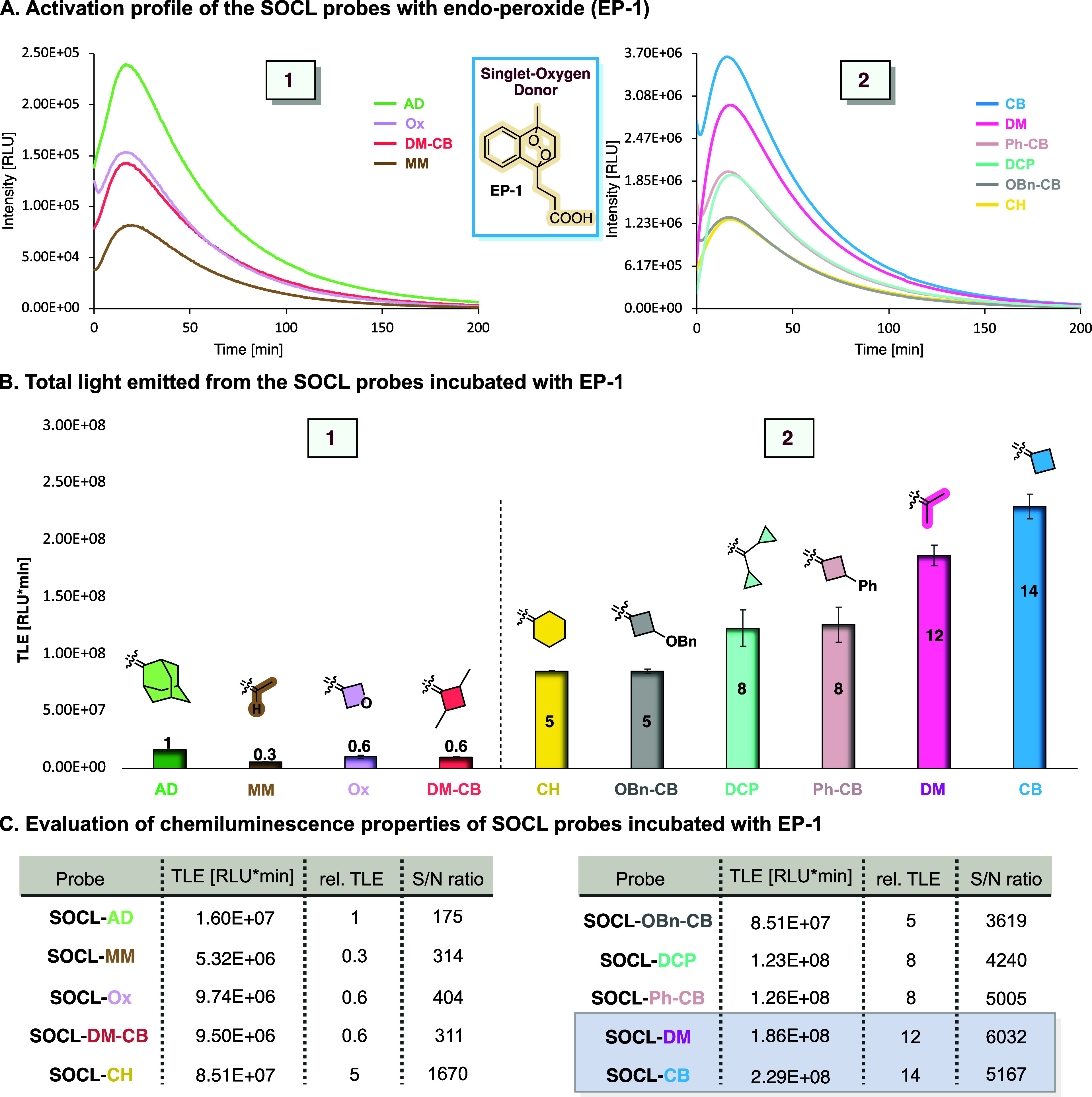
(A) Chemiluminescence kinetic profiles and (B) total light emission (TLE) with relative TLE values of SOCL probes [100 μM] in the presence and absence of endoperoxide (EP-1) [500 μM] in PBS (100 mM, pH 7.4), with 10% DMSO at 37 °C. The TLE of probe 1, SOCL-AD, is used as the reference. (C) Tables summarizing the chemiluminescent properties of the SOCL probes: Total light emission values, relative total light emission using probe SOCL-AD as reference, and signal-to-noise value. The signal-to-noise value is calculated according to the ratio between the total emitted light in the presence and absence of EP-1 (see Figures S1–S3).

Probes containing the *monomethyl* (SOCL-MM), *oxetanyl* (SOCL-Ox), and *dimethyl-cyclobutyl* (SOCL-DM-CB) groups exhibited reduced chemiluminescent intensities, yielding signals that were 0.3-fold, 0.6-fold, and 0.6-fold relative to the adamantyl counterpart, respectively. The diminished signal intensity observed for probes SOCL-MM and SOCL-Ox is likely attributed to the electron deficiency of the enolether unit, despite their reduced steric bulk. In the case of probe SOCL-MM, this deficiency may result from decreased electron donation from the hydrogen atom compared to that of an alkyl group, while for probe SOCL-Ox, it is likely attributed to the electron-withdrawing effect of the oxygen heteroatom. In the case of probe SOCL-DM-CB, the dimethyl substituents are likely to increase the steric hindrance around the enolether unit, thereby impairing its reactivity toward singlet oxygen and resulting in a reduced chemiluminescence signal. These proposed mechanisms are strongly supported by DFT calculations, which revealed smaller HOMO–LUMO energy gaps for probe SOCL-CB and SOCL-DM compared to their electron-withdrawing counterparts SOCL-Ox and SOCL-MM (see Appendix I in the Supporting Information). The computational results confirm that the larger HOMO–LUMO gaps in SOCL-Ox and SOCL-MM correlate with their experimentally observed lower reactivity toward singlet oxygen, providing an electronic-level understanding of the structure–reactivity relationships observed for our chemiluminescent probes.

The total light emission (TLE), relative total light emission (rel. TLE), and calculated signal-to-noise (S/N) values of the nine different SOCL probes are summarized in Figure 3C. Remarkably, all nine SOCL probes, including those with lower chemiluminescent response toward ^1^O_2_, demonstrated superior S/N values than the adamantyl counterpart, probe SOCL-AD, due to the higher background signal of the latter. Chemiluminescent precursors (SOCL probes) that present higher chemiluminescent signals emit a larger number of photons upon reaction with ^1^O_2_ and are expected to exhibit a higher detection sensitivity.

Probe SOCL-CB, which contains a cyclobutyl group, and probe SOCL-DM featuring a dimethyl group, exhibited the highest light emission signal, with S/N values of 5167 and 6032, respectively. These values are significantly greater than the signal produced by the adamantyl enolether, probe SOCL-AD (S/N = 175). Given their superior chemiluminescent responses and sensitivity, the chemiluminescent performance of probes SOCL-CB and SOCL-DM for the detection of ^1^O_2_ was further evaluated.

To assess the practical applicability of probes SOCL-CB and SOCL-DM (Figure 4A), it is essential to establish their selectivity in the presence of various reactive oxygen species (ROS) found in biological systems. Therefore, the probes were incubated in the presence of ^1^O_2_, and seven other ROS (H_2_O_2_, ClO^–^, TBHP, TBO·, ·OH, O_2_^–^, ONOO^–^), and the resulting chemiluminescent signal was measured (Figure 4B1). Notably, none of the ROS, except for ^1^O_2_, induced a significant increase in the chemiluminescent signal observed for probe SOCL-CB (see supporting Figure S4 for probes SOCL-AD and SOCL-DM). To further confirm the specificity of the probes, NaN_3_, a known ^1^O_2_-specific quencher, was added to the reaction system of probe SOCL-CB with EP-1.^41^ As shown in Figure 4B2, the chemiluminescent signal decreased by 92%, indicating that the detectable chemiluminescence was generated by the specific reaction of probe SOCL-CB with ^1^O_2_. Similar results were observed for probes SOCL-AD and SOCL-DM (see Figure S5).

**Figure 4:**
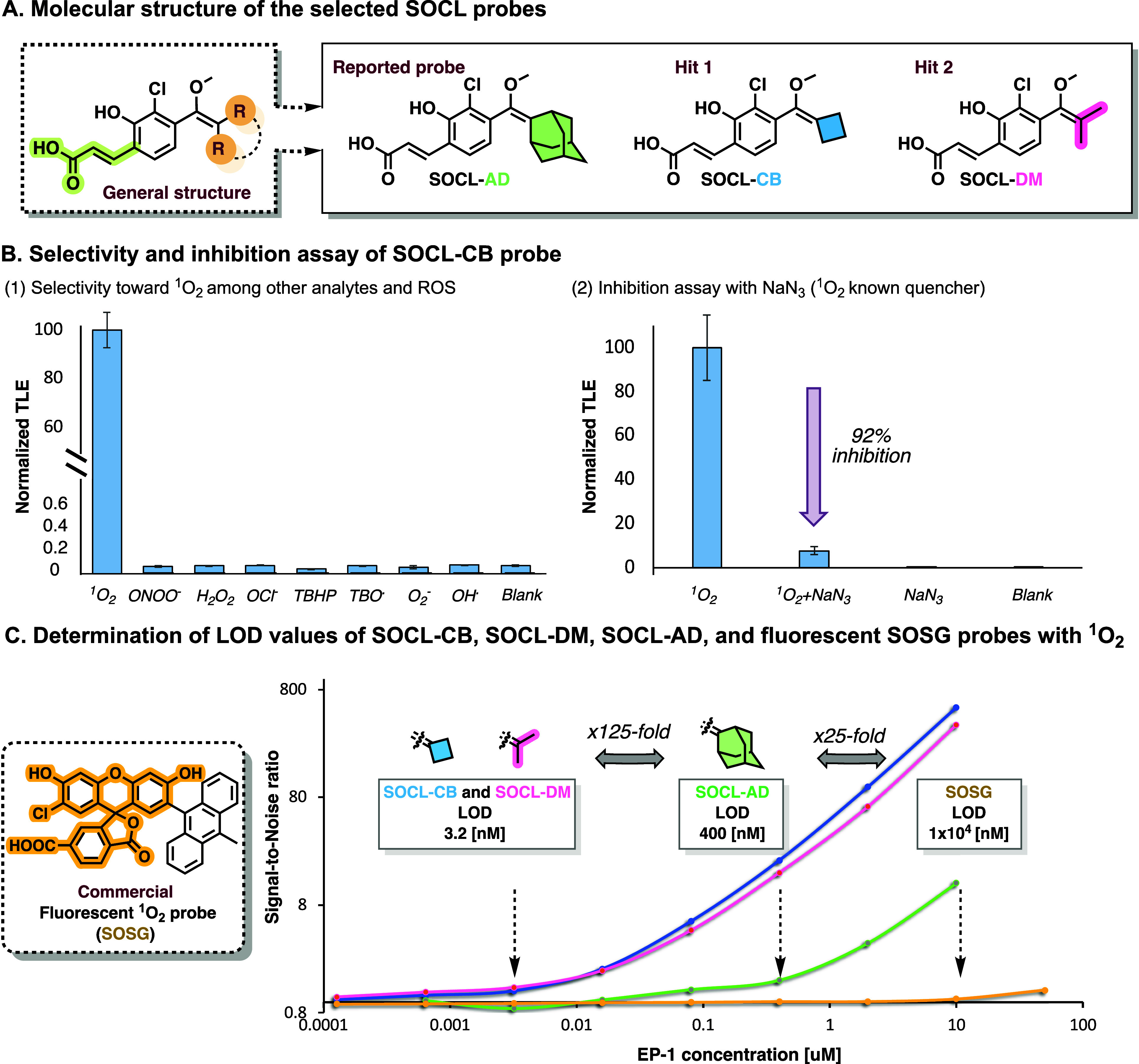
(A) Molecular structure of previously reported probe SOCL-AD vs that of probes SOCL-CB and SOCL-DM. (B) (1) Representative chemiluminescence responses of probe SOCL-CB [100 μM] toward ^1^O_2_, ONOO^–^, and other relevant ROS: H_2_O_2_, ClO^–^, TBHP, TBO^•^, ^•^OH and O_2_^–^ [500 μM] in PBS (100 mM, pH 7.4), with 10% DMSO at 37 °C. (2) Representative chemiluminescence signal attenuation of probe SOCL-CB [100 μM] in the presence and absence of endoperoxide (EP-1) [500 μM] with and without NaN_3_ [10 mM] in PBS (100 mM, pH 7.4), with 10% DMSO at 37 °C (see Figures S4 and S5). (C) Determination of the limit of detection (LOD) values for probes SOCL-AD, SOCL-CB, and SOCL-DM [10 μM] vs commercially available fluorescent SOSG probe [10 μM]. Measurements were taken with various EP-1 concentrations [50–1.28 × 10^–4^ μM] after 60 min (see Figures S6–S13). All measurements were performed in triplicate using independent samples.

Next, we sought to harness the enhanced detection sensitivity of probes SOCL-CB and SOCL-DM toward ^1^O_2_ to determine the limit-of-detection (LOD) in comparison to that of probe SOCL-AD and the commercially available fluorescent probe, singlet oxygen sensor green (SOSG). The LOD was evaluated by measuring the light emission signal over a varied range of EP-1 concentrations (Figure 4C). Remarkably, the LOD value obtained by probes SOCL-CB and SOCL-DM was 3.2 nM, representing a 125-fold and 3125-fold improvement in sensitivity compared to probes SOCL-AD and SOSG, respectively. The obtained linear correlation between the EP-1 concentration and integrated chemiluminescence signal (Figure S6–S11) suggests that the ^1^O_2_ concentration could be straightforwardly determined quantitatively. The exceptional detection sensitivity presented by probes SOCL-CB and SOCL-DM (LOD for ^1^O_2_ below 2 nM), highlights their potential for sensitive and quantitative detection of ^1^O_2_ under physiological conditions.

The reaction mechanism of ^1^O_2_ with the enolether precursors (the SOCL probes) involves the oxidation of the enolether to 1,2-dioxetane through [2 + 2] cycloaddition. This oxidation can also produce an ene-product via the elimination of a proton from the allylic position of the enolether (Figure 5A).^42^ While the dioxetane product undergoes chemiexcitation to generate an excited-state benzoate, which subsequently emits light during its decay, the side ene-product does not exhibit any luminescence.

**Figure 5:**
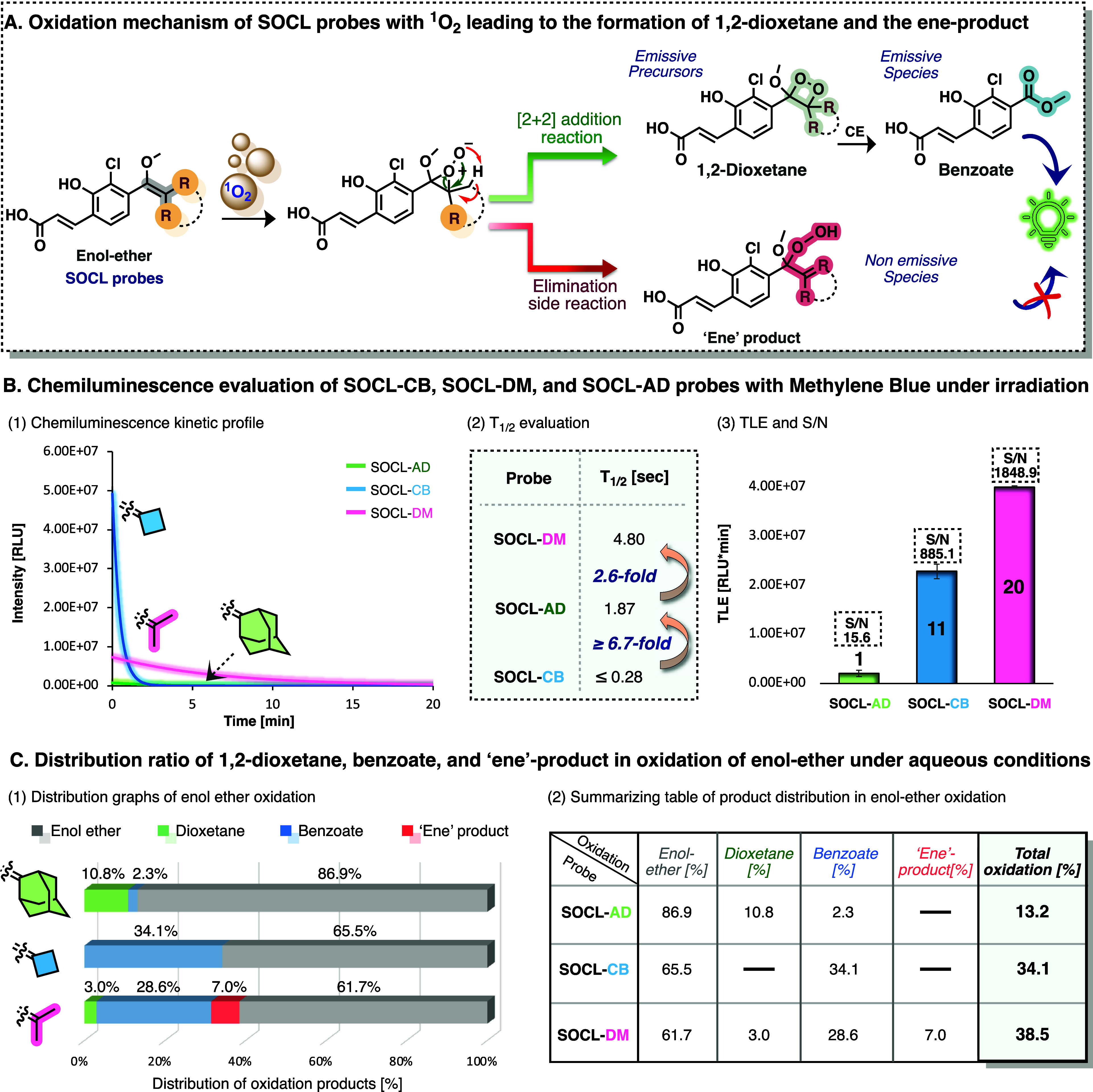
(A) Oxidation pathway of the enolether of the SOCL probes by singlet oxygen leading to an ene-product or a 1,2-dioxetane product. The latter undergoes chemiexcitation decomposition to the corresponding benzoate. (B) (1) Chemiluminescent kinetic profiles of probes SOCL-AD, SOCL-CB, and SOCL-DM [100 μM] in the presence of methylene blue (MB) [10 μM] in PBS (100 mM, pH 7.4), with 1% DMSO after exposure to a white LED lamp for 5 s. (2) Determination of half-life value, *t*_1/2_, (defined as the time point at which half of the total light emission was observed). (3) Total light emission and signal-to-noise values measured for probes SOCL-AD, SOCL-CB, and SOCL-DM [100 μM] incubated in the presence and absence of MB [10 μM] after exposure to a white LED lamp for 5 s in PBS (100 mM, pH 7.4), with 1% DMSO. All measurements were conducted using a SpectraMax iD3 instrument, with injector settings fixed on an integration time of 10 ms. (C) Oxidation of probes SOCL-AD, SOCL-CB, and SOCL-DM [300 μM] incubated with MB [300 μM] and exposure to a white LED lamp for 10 min in acetate buffer (pH 4.2). The product distribution was determined using RP-HPLC (see Figure S25).

The high selectivity and sensitivity obtained by probes SOCL-CB and SOCL-DM toward the detection of ^1^O_2_ prompted us to further validate their performance and evaluate their chemiluminescence properties using a different ^1^O_2_-generating system.

EP-1 has proven effective for ^1^O_2_ generation via thermal decomposition in a stoichiometric manner. However, since thermal degradation is the rate-limiting step, EP-1 is not suitable for assessing the chemiexcitation rate of the probes. In contrast, methylene blue (MB) is a well-known photosensitizer that generates ^1^O_2_ catalytically, upon light irradiation. The formation of ^1^O_2_ is exclusively light-dependent, and the observed kinetic profiles postirradiation reflect the chemiexcitation process of the corresponding 1,2-dioxetane products. Consequently, MB provides a complementary ^1^O_2_-generating system for further evaluating the performance and chemiluminescent properties of the probes upon reaction with ^1^O_2_.

Therefore, probes SOCL-AD, SOCL-CB, and SOCL-DM were incubated in the presence of MB in PBS, pH 7.4, and irradiated with light from a white LED lamp for 5 s. The light emission profiles over 20 min are presented in Figure 5B1. Probe SOCL-CB generated a strong chemiluminescence signal that decayed completely within 2 min, while the probe SOCL-DM exhibited slightly lower intensity but a longer-lasting signal, persisting for over 20 min. Under these conditions, probe SOCL-AD demonstrated a significantly weaker signal, with complete decay after 14 min.

The relative chemiexcitation rates of the three probes were calculated by measuring their total light emission *T*_1/2_ values according to the plots presented in Figure S21. In agreement with our previous findings, the chemiexcitation rate of the probe SOCL-CB was over 7-fold faster than that of the adamantyl counterpart (probe SOCL-AD).^38^ The chemiexcitation rate of probe SOCL-DM was 3-fold slower compared to probe SOCL-AD (Figure 5B2). Additionally, the relative chemiluminescence signals and S/N values were measured in the presence and absence of MB (Figure 5B3), using probe SOCL-AD as a reference after 5 s of exposure to a white light. Notably, the chemiluminescent signals produced by probes SOCL-CB and SOCL-DM were 11-fold and 20-fold more intense than that produced by probe SOCL-AD. Furthermore, the S/N values obtained by probes SOCL-CB and SOCL-DM were 57-fold and 118-fold greater than that obtained by probe SOCL-AD.

The significantly high S/N ratios observed for probes SOCL-CB and SOCL-DM compared to probe SOCL-AD can be attributed to two key factors. First, the reduced steric bulkiness of probes SOCL-CB and SOCL-DM promotes enhanced reactivity with ^1^O_2_, leading to an increased chemiluminescent signal. Second, the previously developed chemiluminescent probe SOCL-AD, exhibited a noticeable background signal caused by self-photooxidation, even in the absence of the photosensitizer, whereas probes SOCL-CB and SOCL-DM displayed a significantly lower background signal (Figure S23).This phenomenon is attributed to the electron-rich carbon–carbon double bonds in the enolether moieties, which undergo oxidation upon light irradiation to form unstable 1,2-dioxetane intermediates that subsequently emit light through chemiexcitation. While this may explain the background signal, the difference in susceptibility to self-photooxidation between the probes is still not fully understood.

To validate the hypothesis that reduced steric hindrance near the enolether unit contributes to higher oxidation efficiency, we compared the oxidation yields of probes SOCL-CB and SOCL-DM to that of probe SOCL-AD under identical conditions. The probes were incubated with MB in acetate buffer (pH 4.2) and subsequently subjected to 10 min of light irradiation using a white LED lamp. The product distribution obtained through the oxidation process was monitored by RP-HPLC (Figure 5C). The oxidation of adamantyl-enolether (probe SOCL-AD) resulted in a 13% conversion, yielding 11% dioxetane and 2% of the corresponding benzoate. In contrast, the oxidation of the cyclobutyl-enolether (probe SOCL-CB) resulted in 34% conversion to benzoate. The oxidation of dimethyl-enolether (probe SOCL-DM) resulted in a 38% conversion, producing 3% dioxetane, 29% benzoate, and 7% of the side ene-product. Expectedly, both probes SOCL-CB and SOCL-DM exhibited higher conversions of the enolether to the desired 1,2-dioxetane and the corresponding benzoate compared to probe SOCL-AD.

We have previously reported that the oxidation of the cyclobutyl enolether (probe SOCL-CB), did not result in any formation of the side ene-product, because the elimination reaction would lead to the generation of a highly constrained cyclic alkene.^38^ In contrast, the oxidation of the dimethyl enol ether (probe SOCL-DM) in an organic solvent resulted in the near-complete formation of the undesired ene-product. Surprisingly, the same oxidation reaction under aqueous conditions favored the [2 + 2] cycloaddition over the elimination side reaction to yield about 78% formation of the dioxetane vs the ene-product.

The superior reactivity and sensitivity presented by probes SOCL-CB and SOCL-DM toward ^1^O_2_ obtained through the various reasons described above, demonstrated their superior capability, compared to probe SOCL-AD, for detecting ^1^O_2_ under physiological conditions. Therefore, we next aimed to assess the capability of our new chemiluminescent probes to detect singlet oxygen produced by a relevant enzymatic model.^43^ Peroxidases are ubiquitous enzymes in all forms of life. In organisms, these enzymes play a pivotal role in detoxifying reactive oxygen species (ROS) and oxidation of numerous compounds.^44,45^ In the past decades, several reports presented the formation of singlet oxygen by peroxidases via direct or indirect processes.^44,46^ Horseradish peroxidase (HRP) is a type of peroxidase widely used in biochemistry and molecular biology for its ability to catalyze the oxidation of various substrates in the presence of hydrogen peroxide (H_2_O_2_).^47^ For instance, HRP plays a crucial role in immunoassays, such as ELISA (enzyme-linked immunosorbent assay), due to its ability to amplify signals by producing colored or luminescent products.^48^ The direct enzymatic production of singlet oxygen by HRP, which requires H_2_O_2_ as a substrate, is illustrated in Figure 6A. Generally, the mechanism is based on the oxidation of the HRP cofactor, iron protoporphyrin IX, with H_2_O_2_ to generate various ROS, including singlet oxygen.^44^ Understanding the HRP’s role in singlet oxygen generation would advance research in chemoenzymatic synthesis and shed light on oxidative mechanisms in biological systems. In addition, quantifying singlet oxygen production in such a system is important because it is a reactive oxygen species (ROS) that can significantly impact cellular processes, oxidative stress, and cell damage. Although several studies have demonstrated the visualization and detection of singlet oxygen formation in cellular environments, quantitative analysis of singlet oxygen has rarely been conducted.^35,49^

**Figure 6:**
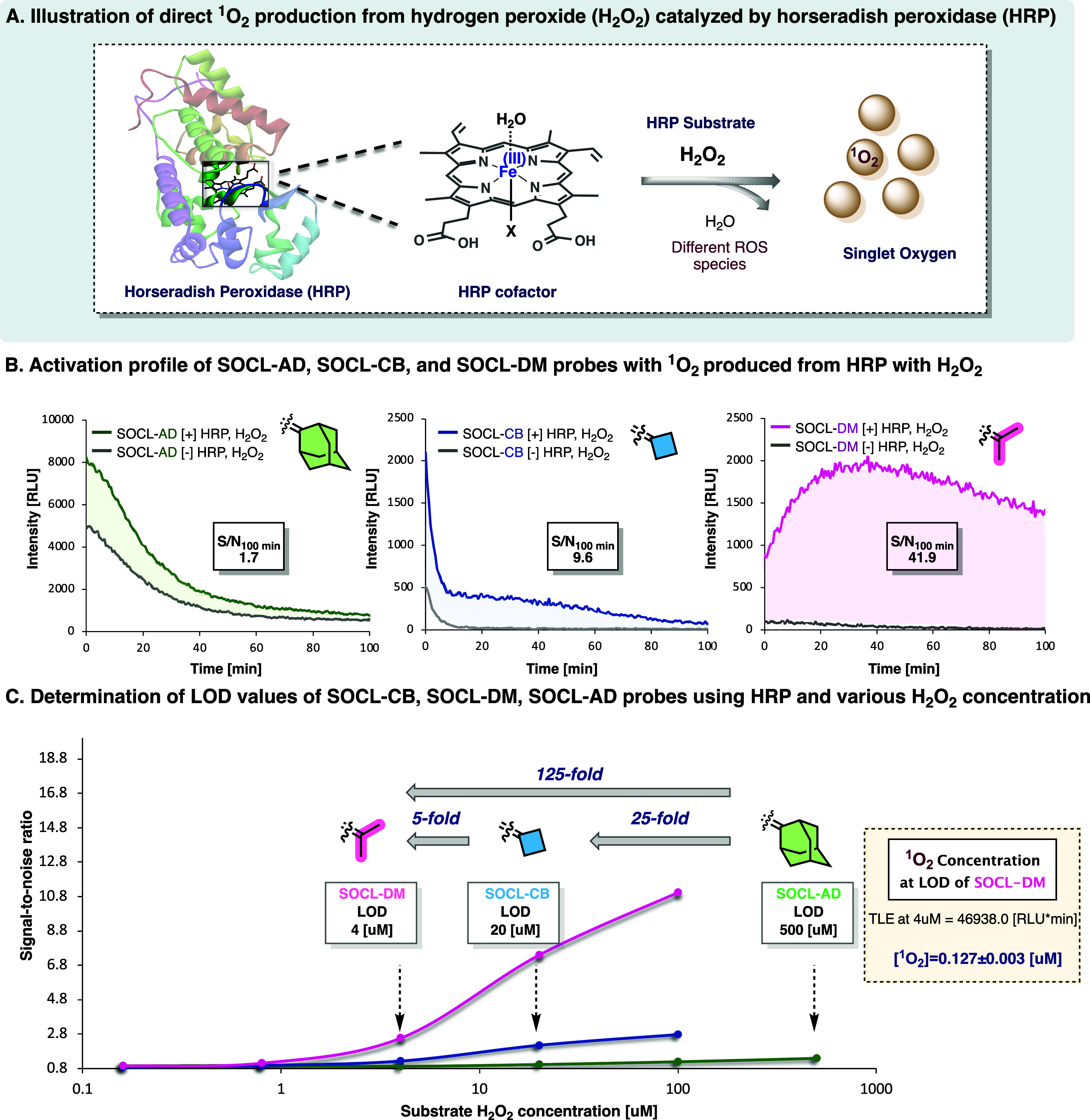
(A) Illustration of direct singlet oxygen production by horseradish peroxidase (HRP) in the presence of H_2_O_2_. (B) Chemiluminescence kinetic profiles and signal-to-noise values over 100 min for probes SOCL-AD, SOCL-CB, and SOCL-DM [100 μM] incubated in the presence and absence of HRP [10 μg/mL] and H_2_O_2_ [100 μM] in PB (50 mM, pH 6.0), 1% DMSO at 30 °C. (C) Determination of the limit of detection value (LOD) for probes SOCL-AD, SOCL-CB, and SOCL-DM [100 μM] for singlet oxygen generated by HRP. Measurements were taken in the presence of HRP [5 μg/mL] and various H_2_O_2_ concentrations [500–0.16 μM] (see Figures S30–S33). All measurements were performed in triplicate using independent samples. Determination of the singlet oxygen concentration detected at the limit of detection of probe SOCL-DM. Calculations were performed as shown in Figure S34.

The high sensitivity and quantitative detection obtained by our chemiluminescent probes toward the detection of ^1^O_2_ encouraged us to evaluate the probes’ ability to detect ^1^O_2_ in enzymatic assay with HRP. Therefore, probes SOCL-AD, SOCL-CB, and SOCL-DM were initially incubated in the presence and absence of HRP and H_2_O_2_ substrate in buffer solution, and the light emission signal was monitored over 100 min (Figure 6B). Expectedly, The S/N values obtained by probes SOCL-DM and SOCL-CB were 25-fold and 6-fold higher than that of probe SOCL-AD, respectively. Noticeably, the S/N value obtained for probe SOCL-DM was 4-fold higher than that of probe SOCL-CB. This result is likely attributed to the accelerated chemiexcitation rate of cyclobutyl-dioxetane compared to dimethyl-dioxetane, resulting in a loss of light within the measurement interval under these conditions. These data demonstrate the superior ability of probe SOCL-DM, over that of probes SOCL-AD and SOCL-CB, to detect ^1^O_2_ produced in enzymatic assays.

Next, we determined the LOD values of the three probes for the detection of ^1^O_2_ produced by HRP with various H_2_O_2_ concentrations. Probes SOCL-CB and SOCL-DM exhibited an LOD value of 20 μM and 4 μM of H_2_O_2_, respectively, while probe SOCL-AD detected ^1^O_2_ at an LOD value of 500 μM of H_2_O_2_ (Figure 6C, left). Expectedly, probe SOCL-DM exhibited higher detection sensitivity compared to that observed by probes SOCL-AD and SOCL-CB. A linear correlation between the singlet oxygen concentration and the integrated chemiluminescence signal (Figure S34) indicates that the probe SOCL-DM is capable of detecting ^1^O_2_ concentrations as low as 127 nM under the applied conditions (Figure 6C, right).

The current study focuses on investigating the structure–activity relationships of singlet oxygen probes that are based on substituted enolether reactive moieties. To extend this study to biological models, substantial additions are necessary. In particular, the influence of the probes’ molecular structure on properties such as cell permeability and water solubility should be assessed and, if needed, optimized accordingly. Preliminary evaluation using bacterial cells as an *in vitro* model was performed with the probes SOCL-AD, SOCL-CB, and SOCL-DM in the presence of a photosensitizer. Following a standard washing protocol, the cells were exposed to light irradiation, and the resulting chemiluminescence signals were measured. Notably, probe SOCL-DM demonstrated higher detection sensitivity (about 5-fold) toward singlet oxygen compared to probe SOCL-AD. The experimental conditions and the obtained data are provided in the Supporting Information (Figures S37 and S38). Furthermore, cell toxicity assays revealed that all probes (SOCL-AD, SOCL-CB, and SOCL-DM) exhibited minimal to no cytotoxicity in the absence of a photosensitizer (Figure S39).

Detecting singlet oxygen (^1^O_2_), among other reactive oxygen species (ROS), is challenging due to its extremely short lifetime in aqueous conditions. Chemiluminescence assays provide a powerful solution for this challenge, as they enable quantitative detection with enhanced sensitivity due to the extremely low background luminescence of both samples and reagents. This is advantageous over the qualitative end-point assays typically conducted with fluorogenic and chromogenic probes. Thus, developing ultrasensitive chemiluminescent probes to detect ^1^O_2_ represents a major advancement in chemiluminescence sensing.^50–53^

A key breakthrough in achieving such sensitivity involved replacing the bulky adamantyl group in chemiluminescent probe structures with compact alternative groups. Traditionally, chemists were cautious about making this substitution due to concerns that it might compromise the chemical stability of the dioxetane. However, the detection of ^1^O_2_ requires employing an enolether, serving as the turn-on reporter, whereas the dioxetane forms in situ and directly undergoes the chemiexcitation process followed by light emission. The enolether is thermally stable compared to its corresponding dioxetane; therefore, replacing the bulky adamantyl group does not compromise its stability.

Additionally, there are also concerns that such changes could affect the oxidation mechanistic pathway, potentially leading to undesired ene-products, which are obtained through the elimination of a proton positioned at the allylic position of the enolether.^54^ This concern limited the replacement of the adamantyl group with a saturated substituent. Herein, we discovered a distinct behavior when employing minimal dimethyl substituent instead of the bulky adamantyl group. Surprisingly, under aqueous conditions, the cycloaddition reaction leading to dioxetane formation is favored (about 78%), whereas in organic solvents, the oxidation process led predominantly to ene-product formation. This unexpected result may be attributed to the polarity of the solvent, whereas polar environments tend to favor dioxetane formation, and nonpolar solvents increase the ene-reaction products.^55,56^ In polar solvents, this reaction tendency occurs due to the stabilization of the transition state for the 1,2-cycloaddition pathway, which leads to dioxetane. Conversely, nonpolar solvents lack this stabilizing effect, allowing the ene reaction to become more dominant. This important finding challenges prior assumptions that such probes are not suitable as chemiluminescent reporters and highlights the crucial role of the solvent in dictating oxidation pathways. The ability of such a probe to generate the dioxetane product rather than the ene-product in water is particularly advantageous for applications involving ^1^O_2_ detection in biological systems, where nonpolar organic solvents are irrelevant.

Addressing the limitations mentioned above, opened a door in designing novel chemiluminescent probes by replacing the bulky adamantyl group with a different compact substituent in order to improve their reactivity toward ^1^O_2_. This modification leads to a reduction in steric hindrance around the reactive site, which allows for greater reactivity with the ^1^O_2_, and improves the oxidation efficiency. The higher reactivity of the probes toward ^1^O_2_, leads to increased photon emission and, consequently, enhanced detection sensitivity.

In this study, we demonstrated that incorporating compact moieties of *cyclobutyl* (probe SOCL-CB) and *dimethyl* groups (probe SOCL-DM), as vinylic substituents in the chemiluminescent probe skeleton, significantly increased the light emission in the presence of ^1^O_2_ compared to both traditional adamantyl-based probe (SOCL-AD) and the commercially available fluorescent probe (SOSG). This increase in light emission signal is directly translated into substantial enhancement of the detection sensitivity (S/N) in the chemiluminescence assay. We observed this enhancement in detection sensitivity across two singlet oxygen-generating systems, endoperoxide (EP-1) thermal decomposition and methylene blue (MB) photosensitization, with an increase ranging from 10- to 100-fold.

The significantly enhanced sensitivity observed for probes SOCL-CB and SOCL-DM prompted us to examine their capability to detect ^1^O_2_ produced in an enzymatic system. One such system involves HRP, one of the most important types of peroxidase enzyme. Previous studies indicated that HRP is capable of producing singlet oxygen, among other ROS, through the catalytic oxidation of hydrogen peroxide.^44,57^ The incubation of the traditional adamantyl probe (SOCL-AD) in the presence of HRP and a high concentration of H_2_O_2_ yielded no significant response, as indicated by a negligible S/N value of 1.7. In contrast, the newly developed probes, SOCL-CB and SOCL-DM, demonstrated substantially enhanced sensitivity, effectively detecting singlet oxygen at concentrations 25-fold and 125-fold lower, respectively, than the detection limit of probe SOCL-AD. This noticeable improvement underscores their potential for low-concentration singlet oxygen detection in enzymatic assays.

It is noteworthy to mention that the superior detection capabilities of probe SOCL-DM compared to probe SOCL-CB, allow for more accurate quantification of ^1^O_2_ produced by HRP at the lowest detectable concentrations of H_2_O_2_ (at the limit of detection, LOD). Such capabilities are vital for effectively monitoring and studying enzymatic processes and estimating singlet oxygen levels involved in these pathways. The combination of high stability and improved oxidation rates positions probes SOCL-CB and SOCL-DM as leading candidates for sensitive, real-time detection of singlet oxygen under physiological conditions. These results are supported by comparative analyses, which included SOCL-AD, previously established as the most sensitive chemiluminescent probe for singlet oxygen detection.

## Conclusions

In summary, we conducted a comprehensive structure–activity optimization study of singlet oxygen probes based on substituted enolethers as precursors for phenoxy-1,2-dioxetane chemiluminescent luminophores. The optimization was achieved by screening three essential parameters: steric hindrance at the enolether oxidation site, the chemiexcitation rate of the produced dioxetane, and total light emission. The S/N values obtained with the cyclobutyl-enolether, probe SOCL-CB, and the dimethyl-enolether, probe SOCL-DM, were 57-fold and 118-fold higher, respectively, than that achieved with the previously reported chemiluminescent singlet oxygen probe, adamantyl-enolether (probe SOCL-AD). The ability of probe SOCL-DM to detect singlet oxygen was evaluated in a relevant enzymatic model, where its production is mediated by horseradish peroxidase. Expectedly, probe SOCL-DM exhibited higher detection sensitivity compared to that observed by probes SOCL-CB and SOCL-AD. Remarkably, probe SOCL-DM was capable of detecting singlet oxygen concentrations as low as 127 nM in the studied HRP-enzymatic assay. As such, probe SOCL-DM is currently the most sensitive chemiluminescent probe for detecting singlet oxygen under physiological conditions. This study highlights the potential for further development of chemiluminescent probes optimized for singlet oxygen detection, offering promising tools for studying oxidative stress, monitoring cellular processes, and improving diagnostic assays.

## Methods

### Chemiluminescence Kinetic Assays of SOCL Probes Using EP-1 (Figures S1–S3)

SOCL Probes and EP-1 stock solutions were prepared in DMSO at 10 mM concentration. Chemiluminescence kinetics were measured using a SpectraMax iD3 plate reader at 37 °C. In a white 96-well Corning plate, each well was loaded with 90 μL PBS (100 mM, pH 7.4), 5 μL SOCL probe (2 mM in DMSO), and 5 μL EP-1 (10 mM in DMSO), resulting in final concentrations of 100 μM probe and 500 μM EP-1 in a total volume of 100 μL. Probes were preincubated in PBS for 30 min before EP-1 addition.

### Selectivity Assays of Probes SOCL-AD, SOCL-CB, and SOCL-DM toward Various ROS (Figure S4)

Selectivity of the probes SOCL-AD, SOCL-CB, and SOCL-DM was evaluated against eight reactive oxygen species: (^1^O_2_, ONOO^–^, H_2_O_2_, ClO^–^, TBHP, TBO·, OH·, O_2_^–^). EP-1 was used as the ^1^O_2_ source. Fresh Solutions of all analytes were prepared immediately before measurements at 50 mM concentrations. In a white 96-well plate, 99 μL of probe solution (100 μM in PBS, pH 7.4) was mixed with 1 μL of each analyte, yielding final analyte concentrations of 500 μM. Chemiluminescence intensity was recorded immediately.

### LOD Determination for EP-1 and Conversion to ^1^O_2_ Concentrations (Figures S6–S13)

A serial 1:5 dilution of EP-1 in DMSO was prepared starting from 1 mM stock solution. Each EP-1 dilution (5 μL) was added to wells containing preincubated SOCL probes [10 μM] or SOSG [10 μM] in PBS (pH 7.4, with 5% DMSO), resulting in EP-1 concentrations ranging from 50 μM to 1.28 × 10^–4^ μM. The total light emission of SOCL probes was measured at 37 °C, and the LOD for EP-1 was determined according to the standard method (blank +3 SD).

#### Conversion to ^1^O_2_ Concentrations

EP-1 undergoes first-order decomposition with a rate constant (*k*) of 4.16 × 10^–4^ s^–1^ at 37 °C. Under these conditions, 78% of EP-1 decomposes within 60 min. Given that EP-1 generates ^1^O_2_ with an 82% yield, this corresponds to the production of 636 nM ^1^O_2_ from an initial 1 μM EP-1 concentration.

### Chemiluminescence Kinetics of SOCL Probes Using Methylene Blue (Figures S15–S24)

Methylene blue (MB) was used as a photosensitizer for ^1^O_2_ generation under light irradiation. Each well contained 98 μL PBS (100 mM, pH 7.4), 1 μL SOCL probe [10 mM], and 1 μL MB [1 mM], resulting in final concentrations of 100 μM probe and 10 μM MB. The plate was irradiated using a PAR38 LED lamp (19 W, 3000 K) for 5 s before measurement. Probes were preincubated for 30 min in PBS before MB addition.

### Oxidation Rate Determination of Probes SOCL-AD, SOCL-CB, and SOCL-DM (Figure S25)

The SOCL probes (enol-ethers) (30 μL) were added to 1 mL acetate buffer (100 mM, pH 4.2), followed by 30 μL methylene blue (final concentration: 300 μM each). After 10 min of light irradiation, samples were analyzed by RP-HPLC using a gradient of 30–100% ACN in water with 0.1% TFA. Oxidation yields were determined by peak integration of the dioxetane, benzoate, and “ene” products relative to the unreacted enol ether (absorbance at 330 nm).

### Biological Evaluation

#### Detection of HRP-Mediated ^1^O_2_ Generation (Figures S26–S28)

Reactions were performed in a white 96-well plate containing 97 μL phosphate buffer (50 mM, pH 6.0) and 1 μL SOCL probe [10 mM], for a final probe concentration of 100 μM. After 30 min incubation at 30 °C, 1 μL HRP [1 mg/mL] and 1 μL H_2_O_2_ [10 mM] were added, yielding 10 μg/mL HRP and 100 μM H_2_O_2_. Chemiluminescence was recorded immediately.

#### LOD Assay for H_2_O_2_-Mediated ^1^O_2_ Generation via HRP (Figures S30–S33)

To determine the limit of detection (LOD), a serial 1:5 dilution of H_2_O_2_ was prepared in phosphate buffer (PB, 50 mM, pH 6.0), starting from a 10 mM stock solution. Measurements were conducted in a white 96-well plate, with each well containing 93 μL of PB (50 mM, pH 6.0) and 1 μL of SOCL probe (10 mM), resulting in a final probe concentration of 100 μM. After a 30 min incubation at 30 °C, 1 μL of horseradish peroxidase (HRP, 1 mg/mL) was added to achieve a final HRP concentration of 10 μg/mL. Subsequently, 5 μL of H_2_O_2_ solutions were added to obtain final concentrations ranging from 500 μM to 0.16 μM. The total emitted light after 300 min was recorded to evaluate the LOD. The LOD for H_2_O_2_ was determined using the standard method (blank +3SD).

#### LOD Calculation of ^1^O_2_ Using Probe SOCL-DM (Figure S34)

To determine the LOD for ^1^O_2_ using the probe SOCL-DM, a calibration curve was first generated. This was accomplished by measuring the total light emission of probe SOCL-DM in the presence of varying concentrations of EP-1. Since EP-1 concentration correlates linearly with ^1^O_2_ production, a calibration curve plotting TLE against ^1^O_2_ concentration after 60 min was constructed. The LOD for ^1^O_2_ generated by the HRP/H_2_O_2_ system was extrapolated from the slope of this calibration curve, using the TLE corresponding to the LOD concentration of H_2_O_2_.

#### Detection of Intracellular ^1^O_2_ in Bacteria (Figures S37, S38)

*Bacillus subtilis* ATCC 14945 was purchased from the American Type Culture Collection and cultured in LB medium at 30 °C for 24 h with shaking. The bacterial cultures were diluted with LB medium to obtain OD_600_ of 0.85. The bacterial suspension was then divided into 4 different aliquots, each containing 1 mL of the suspension.

The bacteria were incubated under four conditions: (1) bacteria alone, (2) bacteria with SOCL probe [100 μM], (3) bacteria with methylene blue (MB) [50 μM], and (4) bacteria with both SOCL probe and MB, all with 1% DMSO in LB. For appropriate samples, 10 μL of SOCL probe (from 10 mM stock) or 10 μL of methylene blue (MB) (from 5 mM stock) was added to achieve final concentrations of 100 μM and 50 μM, respectively. After 3 h of incubation, the bacteria was centrifuged, and the bacterial pellet was washed twice with PBS (pH 7.4) (at 5000 rpm, 5 min), followed by resuspension in 1 mL of PBS (pH 7.4).

A 96-well lidded clear plate (Corning) was utilized, with each well preloaded with 100 μL of each of the four combinations for each probe under identical conditions. The plate was subjected to irradiation using PAR38 LED lamp (19W, 3000K) for 1 min. The chemiluminescence signal was monitored using Molecular Devices Spectramax iD3 over 15 min of incubation at 37 °C.

#### Cytotoxicity Following Probe and Light Exposure (Figure S39)

*Bacillus subtilis* ATCC 14945 was cultured in LB at 30 °C for 24 h with shaking. The initial culture of the bacteria was centrifuged (at 5000 rpm, 5 min), washed with PBS (pH 7.4), and the bacterial pellets were resuspended in PBS (pH 7.4) to obtain OD_600_ of 0.85. Next, 100 μL of bacterial suspension was added to a 96-well plate (Corning). Bacterial cells were treated with SOCL probes [100 μM], and MB [10 μM] or both, and irradiated for 5 min. Control wells without SOCL probes and MB containing bacterial cells were also prepared. The amount of viable cells was assessed by modified 3-(4,5-dimethylthiazol-2-yl)-2,5-diphenyltetrazolinium bromide (MTT) assay. After 5 min of irradiation, the cells were centrifuged (at 4000 rpm, 8 min) and washed with PBS. The cells were resuspended in PBS, and MTT solution in PBS (100 μL of 1 mg/mL) was added to the wells to achieve a final concentration of 0.5 mg/mL. The cells were incubated for 4 h at 30 °C. The 96-well plate was then centrifuged, and the medium was replaced with 100 μL of isopropanol containing 5% HCl 1 M to dissolve the formazan crystals formed. The plate was incubated for 16 h at room temperature. Absorbance of the solution was measured at 570 nm by a Tecan pro 200 plate reader. The percentage of viable cells was normalized to the viability of nontreated cells (100% viability).

### Materials

All general reagents, including salts and solvents, were purchased from Sigma-Aldrich and used as received. 2,4-Dimethylcyclobutanone was supplied by Biosynth. The singlet oxygen probe SOSG was obtained from Lumiprobe. Horseradish peroxidase (type VI-A) was purchased from Sigma-Aldrich. Light irradiation for photochemical reactions was carried out using an LED PAR38 lamp (19 W, 3000 K). All SOCL probes were synthesized as described in the Supporting Information. The detailed instrumentation for characterizing synthesized materials and the spectroscopic methods can be found in the Supporting Information.

## Supplementary Material



## Notes

Synthetic procedures and characterization of all compounds, including ^1^H NMR, ^13^C NMR, HPLC, and MS; experimental protocols; DFT calculations; Figures S1–S39 (PDF)
