# Supplementary material for: Structure–Activity Optimization of Phenoxy-1,2-dioxetane Precursors as Probes for Singlet Oxygen Yields Unprecedented Detection Sensitivity
Source: JACS Au. 2025 May 23;5(6):2871–83. doi: 10.1021/jacsau.5c00465 (PMC12188417; doi:10.1021/jacsau.5c00465)
Supplement: Supplementary file 1 [file au5c00465_si_001.pdf]

Supporting Information

Structure-Activity Optimization of Phenoxy-1,2-Dioxetane Precursors as Probes for Singlet Oxygen Yields Unprecedented Detection Sensitivity

Rozan Tannous,[a] Tal Kopp,[a] and Doron Shabat[a]*

[a]School of Chemistry, Raymond and Beverly Sackler Faculty of Exact Sciences, Tel-Aviv University, Tel Aviv 69978 Israel.

***Corresponding Author:**

Doron Shabat, Email: chdoron@tauex.tau.ac.il

Table of Contents

General Methods **S3**

Synthesis and Characterization **S4-S18**

General synthetic procedures of the SOCL probes S4-S5

Synthesis and characterization of SOCL Probes 2-10........ S6-S17

Characterization of the 'ene'-product from oxidation of probe SOCL-DM S18

Experimental protocols **S19-S21**

Bacterial experiments **S22**

Appendix I – Computational data **S23-S35**

Appendix II - Supplementary Figures **S36-S56**

Appendix III – Spectral data of Compounds **S57-S98**

Appendix IV – HPLC of Key Compounds **S99-S108**

References **S109-S110**

General methods

All reactions requiring anhydrous conditions were performed under an Argon atmosphere. All reactions were carried out at room temperature unless stated otherwise. Chemicals and solvents were either A.R. grade or purified by standard techniques. Thin-layer chromatography (TLC): silica gel plates Merck 60 F254: compounds were visualized by irradiation with UV light. Reverse-phase high-pressure liquid chromatography (RP-HPLC): C18 5u, 250x4.6mm, eluent given in parentheses. Preparative RP-HPLC: C18 5u, 250x21mm, eluent given in parentheses. ^{1}H-NMR spectra were measured using Bruker Avance operated at 400MHz. ^{13}C-NMR spectra were measured using Bruker Avance operated at 100 MHz. Chemical shifts were reported in ppm on the δ scale relative to a residual solvent ($CDCl_3$: δ = 7.26 for ^{1}H-NMR and 77.16 for ^{13}C-NMR and DMSO-d_6: δ = 2.50 for ^{1}H-NMR and 39.52 for ^{13}C-NMR). Mass spectra were measured on Waters Xevo TQD. Chemiluminescence was recorded on Molecular Devices Spectramax iD3. Fluorescence was recorded on Tecan Infinite 200 Pro. All general reagents, including salts and solvents, were purchased from Sigma-Aldrich. 2,4-Dimethylcyclobutanone was purchased from Biosynth. Probe Singlet Oxygen Sensor Green (SOSG) was purchased from Lumiprobe. Light irradiation for photochemical reactions: LED PAR38 lamp (19W, 3000K). Horseradish peroxidase (type VI-A) was purchased from Sigma-Aldrich.

Abbreviations

$CHCl_3$- Chloroform, **DCM**- Dichloromethane, **DMF**- Dimethylformamide, **EtOAc**- Ethyl acetate, **Et_3N**– Triethylamine, **Hex**- Hexanes, **LDA**- Lithium diisopropylamide, **MB**- Methylene blue, **PFA**-Paraformaldehyde, **TBS**– Tert-butyldimethylsilyl, **THF**- Tetrahydrofuran.

General synthetic procedures of the Singlet oxygen chemiluminescent (SOCL) probes

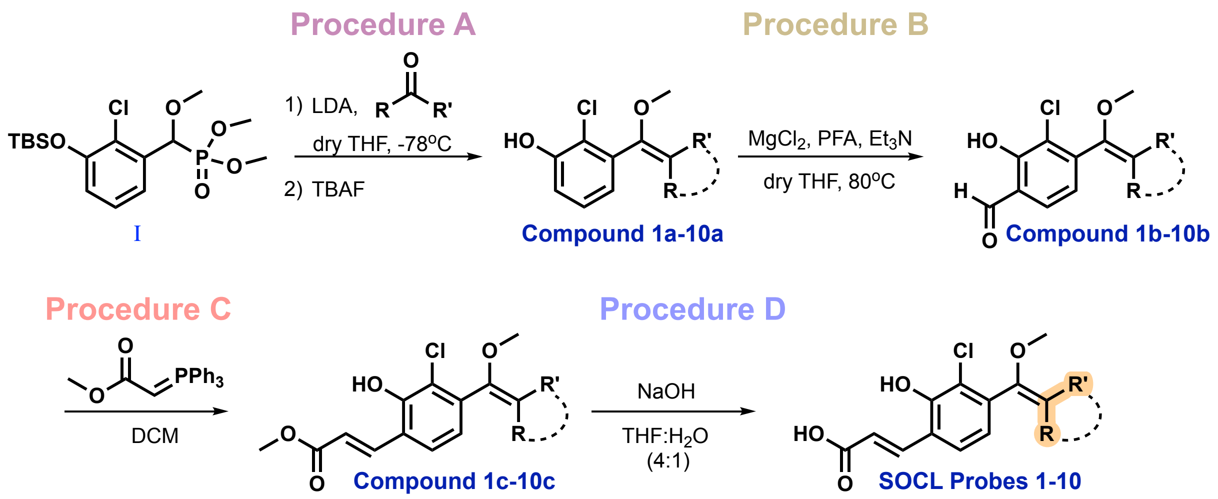

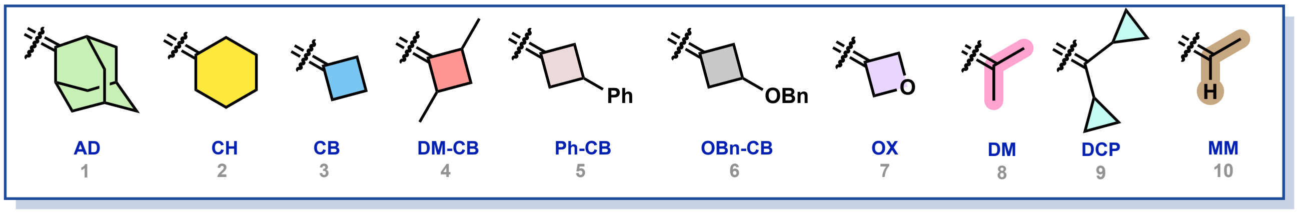

Probe SOCL-AD (1) was synthesized according to a known procedure.[1] All compounds were synthesized according to general **procedure A-D**.

Procedure A *- Wittig-Horner reaction and TBS deprotection.*

Phosphonate **I**[2] (1.0 equiv.) was dissolved in anhydrous THF under an argon atmosphere and cooled to -78℃. LDA (2.0 M in THF, 1.5 equiv.) was added dropwise, and the solution was stirred for 20 minutes. Cycloalkyl ketone (~1.2-3.0 equiv.) was added, and the reaction was warmed immediately to room temperature and stirred for an additional 20 minutes. The reaction was monitored by TLC (Hex:EtOAc mixture). Upon completion, TBAF (1.0 M in THF, 1.1 eq) was added, and the reaction was stirred for an additional 10 minutes at room temperature. The reaction was monitored by TLC (Hex:EtOAc mixture) and after full conversion, the reaction mixture was diluted with EtOAc and washed with 1M HCl followed by two additional washes with brine. The organic layer was dried over Na_2SO_4 and concentrated under reduced pressure. The crude product was further purified by column chromatography (Hex:EtOAc mixture). Subsequently, the sample was placed under a high vacuum to remove residual TBS-F, affording compounds **1a-10a**.

Procedure B *– Ortho-formylation*

In pressure flask compounds **1a-10a** (1.0 equiv.) were dissolved in dry THF, and Et_3N (4.0 equiv.) was added. Then, $MgCl_2$ (2.0 equiv.) and paraformaldehyde (PFA) (8.0 equiv.) were added, and the reaction was heated to 80 °C. The reaction was monitored by TLC (Hex:EtOAc mixture) and upon

completion, the mixture was diluted with EtOAc and washed with 1M HCl. The organic layer was separated and washed twice with brine, dried over Na_2SO_4, and concentrated under reduced pressure. The crude product was purified by column chromatography (Hex:EtOAc mixture) affording compounds **1b-10b**.

Procedure C – *Wittig reaction*

To a solution of compounds **1b-10b** (1.0 equiv.) in DCM, methyl (triphenyl-phosphoranylidene) acetate (1.1 equiv.) was added. The reaction was stirred for 10 minutes and monitored by TLC (Hex:EtOAc). Upon completion, the reaction mixture was diluted with DCM and washed with saturated ammonium chloride solution followed by an additional wash with brine. The organic layer was then dried over Na_2SO_4, filtered, and concentrated under reduced pressure. The crude product was purified by column chromatography (Hex:EtOAc) to afford compounds **1c-10c**.

Procedure D – *Hydrolysis*

Compound **1c-10c** (1.0 equiv.) and NaOH (10 equiv.) were dissolved in a mixture of THF:H_2O (4:1). The reaction mixture was stirred at 40 °C and monitored by TLC (Hex:EtOAc). Upon completion, the reaction mixture was diluted with EtOAc and washed with 1M HCl. The organic layer was separated, washed twice with brine, dried over Na_2SO_4, and concentrated under reduced pressure. The crude product was further purified by column chromatography (Hex:EtOAc) to afford the desired **SOCL probes 1-10**.

Synthesis and characterization of SOCL Probes 2-10

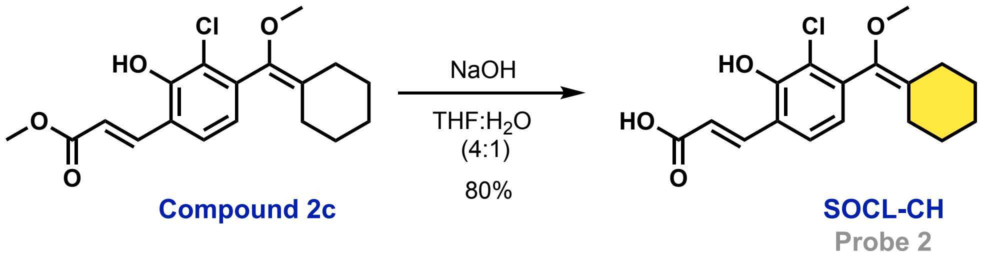

Probe 2 – SOCL-CH

Compound **2c**[3] (90 mg, 0.27 mmol) was dissolved in 800 μL of THF and 200 μL of H_2O and reacted with NaOH (106 mg, 2.67 mmol) according to **procedure D**. The crude residue was purified by column chromatography (60:40, Hex:EtOAc), to obtain **Probe 2** in the form of a white solid (69 mg, 0.21 mmol, 80%).

^{1}H NMR (400 MHz, $CDCl_3$) δ 8.05 (d, *J* = 16.1 Hz, 1H), 7.42 (d, *J* = 8.0 Hz, 1H), 6.89 (d, *J* = 8.0 Hz, 1H), 6.65 (d, *J* = 16.1 Hz, 1H), 3.30 (s, 3H), 2.41 (t, *J* = 5.6 Hz, 2H), 1.87 – 1.81 (m, 2H), 1.65 – 1.44 (m, 6H).

^{13}C NMR (100 MHz, $CDCl_3$) δ 172.25, 150.70, 142.40, 141.23, 137.02, 127.03, 125.36, 123.76, 121.62, 121.59, 119.20, 57.08, 29.90, 27.67, 27.56, 26.79, 26.70.

MS (ES-): m/z calc. for $C_{17}H_{19}ClO_4$: 322.1; found: 321.4 $[M-H]^-$.

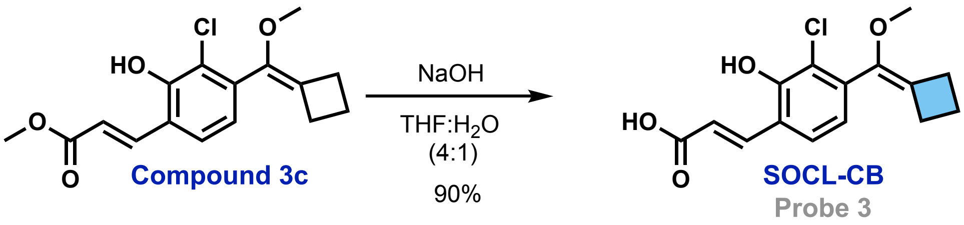

Probe 3 – SOCL-CB

Compound **3c**[4] (150 mg, 0.49 mmol) was dissolved in 1200 μL of THF and 300 μL of H_2O and reacted with NaOH (194 mg, 4.86 mmol) according to **procedure D**. The crude residue was purified by column chromatography (60:40, Hex:EtOAc), to obtain **Probe 3** in the form of a white solid (128.9 mg, 0.44 mmol, 90%).

^{1}H NMR (400 MHz, $CDCl_3$) δ 8.04 (d, *J* = 16.1 Hz, 1H), 7.41 (d, *J* = 8.1 Hz, 1H), 6.94 (d, *J* = 8.1 Hz, 1H), 6.64 (d, *J* = 16.1 Hz, 1H), 3.54 (s, 3H), 3.01 – 2.93 (m, 2H), 2.61 – 2.54 (m, 2H), 2.08 – 1.98 (m, 2H).

^{13}C NMR (100 MHz, $CDCl_3$) δ 171.85, 150.86, 143.54, 141.21, 136.52, 127.10, 122.91, 122.71, 121.52, 120.28, 119.02, 57.61, 28.91, 28.51, 17.49.

MS (ES-): m/z calc. for $C_{15}H_{15}ClO_4$: 294.1; found: 293.3 $[M-H]^-$.

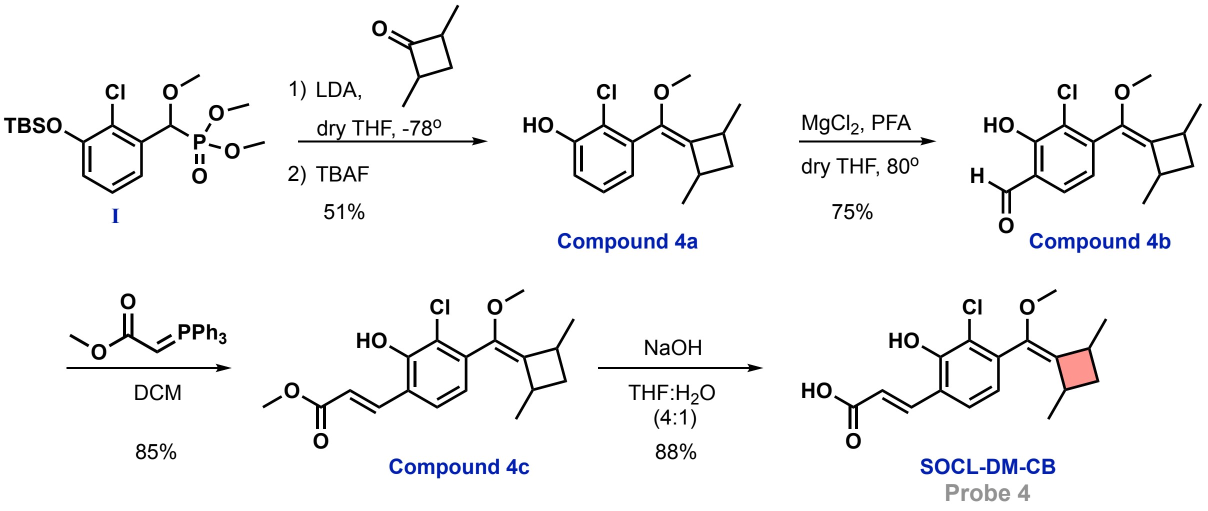

Compound 4a

Compound **4a** was synthesized according to **Procedure A**, using phosphonate **I**[2] (600 mg, 1.52 mmol) in dry THF (5 mL), LDA (2.0 M in THF, 1.14 mL, 2.28 mmol) and 2,4-Dimethylcyclobutanone (178.9 mg, 1.82 mmol). The reaction was monitored by TLC (90:10, Hex:EtOAc). Upon completion, TBAF (1.0 M in THF, 1.67 mL, 1.67 mmol) was added to the solution. The crude residue was purified by column chromatography (80:20, Hex:EtOAc), to afford compound **4a** in the form of a colorless oil (195 mg, 0.77 mmol, 51%).

^{1}H NMR (400 MHz, $CDCl_3$) δ 7.16 (t, *J* = 7.8 Hz, 1H), 7.01 (dd, *J* = 8.2, 1.6 Hz, 1H), 6.90 (dd, *J* = 7.5, 1.6 Hz, 1H), 3.37 (s, 3H), 3.11 (dquin, *J* = 8.9, 7.0 Hz, 1H), 2.82 (dquin, *J* = 9.0, 6.9 Hz, 1H), 2.38 (dt, *J* = 11.0, 9.0 Hz, 1H), 1.36 (d, *J* = 7.0 Hz, 3H), 1.14 (dt, *J* = 11.0, 6.8 Hz, 1H), 0.72 (d, *J* = 7.0 Hz, 3H).

^{13}C NMR (100 MHz, $CDCl_3$) δ 151.54, 144.88, 134.63, 133.35, 127.29, 123.91, 120.04, 115.64, 56.66, 34.28, 33.61, 32.58, 20.85, 19.92.

MS (ES-): m/z calc. for $C_{14}H_{17}ClO_2$: 252.1; found: 251.2 $[M-H]^-$.

Compound 4b

To a solution of compound **4a** (80 mg, 0.32 mmol) in dry THF (2 mL), Et_3N (178 µL, 1.27 mmol), $MgCl_2$ (60 mg, 0.64 mmol), and paraformaldehyde (77 mg, 2.56 mmol) were added following **Procedure B**. The crude residue was purified by column chromatography (80:20, Hex:EtOAc), to afford compound **4b** in the form of a yellow oil (67 mg, 0.24 mmol, 75%).

^{1}H NMR (400 MHz, $CDCl_3$) δ 11.61 (s, 1H), 9.88 (s, 1H), 7.46 (d, *J* = 7.9 Hz, 1H), 7.04 (d, *J* = 7.9 Hz, 1H), 3.37 (s, 3H), 3.17 – 3.05 (m, 1H), 2.94 – 2.80 (m, 1H), 2.38 (dt, *J* = 10.9, 9.1 Hz, 1H), 1.34 (d, *J* = 7.0 Hz, 3H), 1.15 (dt, *J* = 10.9, 6.9 Hz, 1H), 0.70 (d, *J* = 6.9 Hz, 3H).

^{13}C NMR (100 MHz, $CDCl_3$) δ 195.84, 157.63, 144.21, 143.21, 136.05, 130.88, 122.70, 122.39, 120.54, 57.30, 34.30, 34.03, 32.87, 20.70, 19.82.

MS (ES-): m/z calc. for $C_{15}H_{17}ClO_3$: 280.1; found: 279.3 $[M-H]^-$.

Compound 4c

Compound **4b** (40 mg, 0.14 mmol) was reacted following **Procedure C**. The crude residue was purified by column chromatography (70:30, Hex:EtOAc), to obtain compound **4c** in the form of a white solid (41 mg, 0.12 mmol, 85%).

^{1}H NMR (400 MHz, $CDCl_3$) δ 7.98 (d, *J* = 16.2 Hz, 1H), 7.37 (d, *J* = 8.0 Hz, 1H), 6.92 (d, *J* = 8.0 Hz, 1H), 6.62 (d, *J* = 16.2 Hz, 1H), 3.82 (s, 3H), 3.35 (s, 3H), 3.16 – 3.05 (m, 1H), 2.88 – 2.78 (m, 1H), 2.37 (dt, *J* = 10.8, 9.1 Hz, 1H), 1.34 (d, *J* = 7.0 Hz, 3H), 1.14 (dt, *J* = 11.0, 6.9 Hz, 1H), 0.72 (d, *J* = 6.9 Hz, 3H).

^{13}C NMR (100 MHz, $CDCl_3$) δ 167.73, 150.62, 144.59, 139.26, 136.23, 134.61, 126.56, 123.38, 122.12, 120.94, 119.58, 56.88, 51.76, 34.21, 33.74, 32.65, 20.72, 19.89.

MS (ES-): m/z calc. for $C_{18}H_{21}ClO_4$: 336.1; found: 335.4 $[M-H]^-$.

Probe 4 – SOCL-DM-CB

Compound **4c** (41 mg, 0.12 mmol) was dissolved in 320 μL of THF and 80 μL of H_2O and reacted with NaOH (49 mg, 1.21 mmol) according to **procedure D**. The crude residue was purified by column chromatography (60:40, Hex:EtOAc), to obtain **Probe 4** in the form of a white solid (34 mg, 0.11 mmol, 88%).

^{1}H NMR (400 MHz, $CDCl_3$) δ 8.05 (d, *J* = 16.1 Hz, 1H), 7.42 (d, *J* = 8.1 Hz, 1H), 6.96 (d, *J* = 8.0 Hz, 1H), 6.65 (d, *J* = 16.1 Hz, 1H), 3.38 (s, 3H), 3.18 – 3.07 (m, 1H), 2.91 – 2.80 (m, 1H), 2.39 (dt, *J* = 11.0, 9.1 Hz, 1H), 1.37 (d, *J* = 7.0 Hz, 3H), 1.16 (dt, *J* = 11.1, 6.9 Hz, 1H), 0.74 (d, *J* = 6.9 Hz, 3H).

^{13}C NMR (100 MHz, $CDCl_3$) δ 172.45, 150.69, 144.49, 141.22, 136.66, 134.95, 126.95, 123.50, 121.72, 120.93, 119.25, 56.96, 34.21, 33.79, 32.68, 20.71, 19.88.

MS (ES-): m/z calc. for $C_{17}H_{19}ClO_4$: 322.1; found: 321.4 $[M-H]^-$

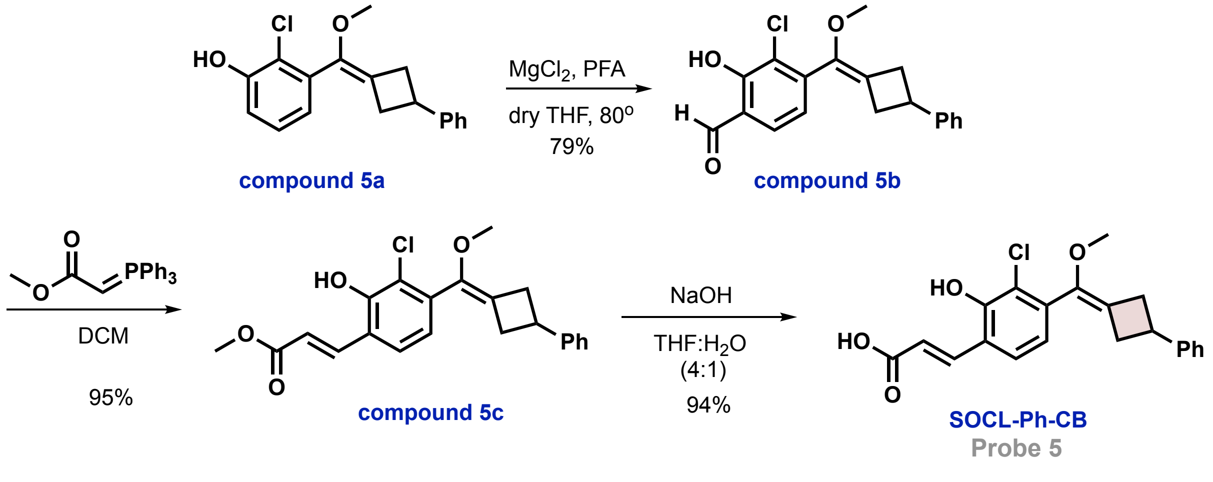

Compound 5b

To a solution of compound **5a**[4] (113 mg, 0.38 mmol) in dry THF (2.5 mL), Et_3N (213 μL, 1.52 mmol), $MgCl_2$ (72 mg, 0.76 mmol) and paraformaldehyde (91 mg, 3.04 mmol) were added following

Procedure B. The crude residue was purified by column chromatography (80:20, Hex:EtOAc), to afford compound **5b** in the form of a yellow oil (99 mg, 0.30 mmol, 79%).

^{1}H NMR (400 MHz, $CDCl_3$) δ 11.65 (s, 1H), 9.88 (s, 1H), 7.48 (d, *J* = 8.0 Hz, 1H), 7.35 – 7.26 (m, 4H), 7.21 (t, *J* = 6.9 Hz, 1H), 7.06 (d, *J* = 7.9 Hz, 1H), 3.60 (s, 3H), 3.66 – 3.55 (m, 1H), 3.50 – 3.41 (m, 1H), 3.14 – 3.05 (m, 1H), 2.97 – 2.87 (m, 1H), 2.83 – 2.74 (m, 1H).

^{13}C NMR (100 MHz, $CDCl_3$) δ 195.85, 157.95, 145.53, 144.12, 142.80, 131.18, 128.58, 126.55, 126.32, 126.17, 121.90, 120.55, 119.54, 57.97, 36.99, 36.61, 35.71.

MS (ES-): m/z calc. for $C_{19}H_{17}ClO_3$: 328.1; found: 327.3 $[M-H]^-$.

Compound 5c

Compound **5b** (50 mg, 0.17 mmol) was reacted following **Procedure C**. The crude residue was purified by column chromatography (80:20, Hex:EtOAc), to obtain compound **5c** in the form of a white solid (52 mg, 0.16 mmol, 95%).

^{1}H NMR (400 MHz, $CDCl_3$) δ 7.98 (d, *J* = 16.2 Hz, 1H), 7.40 (d, *J* = 8.1 Hz, 1H), 7.37 – 7.27 (m, 4H), 7.22 (tt, *J* = 6.3, 1.5 Hz, 1H), 6.96 (d, *J* = 8.0 Hz, 1H), 6.63 (d, *J* = 16.2 Hz, 1H), 6.43 (brs, 1H), 3.84 (s, 3H), 3.67 – 3.57 (m, 1H), 3.58 (s, 3H), 3.50 – 3.41 (m, 1H), 3.13 – 3.05 (m, 1H), 2.98 – 2.90 (m, 1H), 2.83 – 2.74 (m, 1H).

^{13}C NMR (100 MHz, $CDCl_3$) δ 167.68, 150.78, 145.52, 144.38, 139.15, 135.96, 128.42, 126.89, 126.41, 126.14, 122.60, 122.12, 120.34, 119.76, 118.13, 57.62, 51.78, 36.81, 36.30, 35.55.

MS (ES-): m/z calc. for $C_{22}H_{21}ClO_4$: 384.1; found: 383.5 $[M-H]^-$.

Probe 5 – SOCL-Ph-CB

Compound **5c** (51 mg, 0.13 mmol) was dissolved in 400 μL of THF and 100 μL of H_2O and reacted with NaOH (53 mg, 1.32 mmol) according to **procedure D**. The crude residue was purified by column chromatography (60:40, Hex:EtOAc), to obtain **Probe 5** in the form of a white solid (45 mg, 0.12 mmol, 94%).

^{1}H NMR (400 MHz, CDCl3) δ 8.06 (d, J = 16.1 Hz, 1H), 7.43 (d, J = 8.1 Hz, 1H), 7.37 – 7.29 (m, 4H), 7.26 – 7.20 (m, 1H), 6.98 (d, J = 8.1 Hz, 1H), 6.65 (d, J = 16.1 Hz, 1H), 3.68 – 3.57 (m, 1H), 3.59 (s, 3H), 3.49 – 3.41 (m, 1H), 3.09 (ddd, J = 15.5, 7.3, 2.6 Hz, 1H), 2.99 – 2.89 (m, 1H), 2.79 (ddd, J = 15.3, 7.4, 2.5 Hz, 1H).

^{13}C NMR (100 MHz, $CDCl_3$) δ 172.16, 150.91, 145.48, 144.30, 141.16, 136.38, 128.42, 127.19, 126.40, 126.15, 122.67, 121.74, 120.34, 119.24, 118.37, 57.65, 36.82, 36.31, 35.55.

MS (ES-): m/z calc. for $C_{21}H_{19}ClO_4$: 370.1; found: 369.4 $[M-H]^-$.

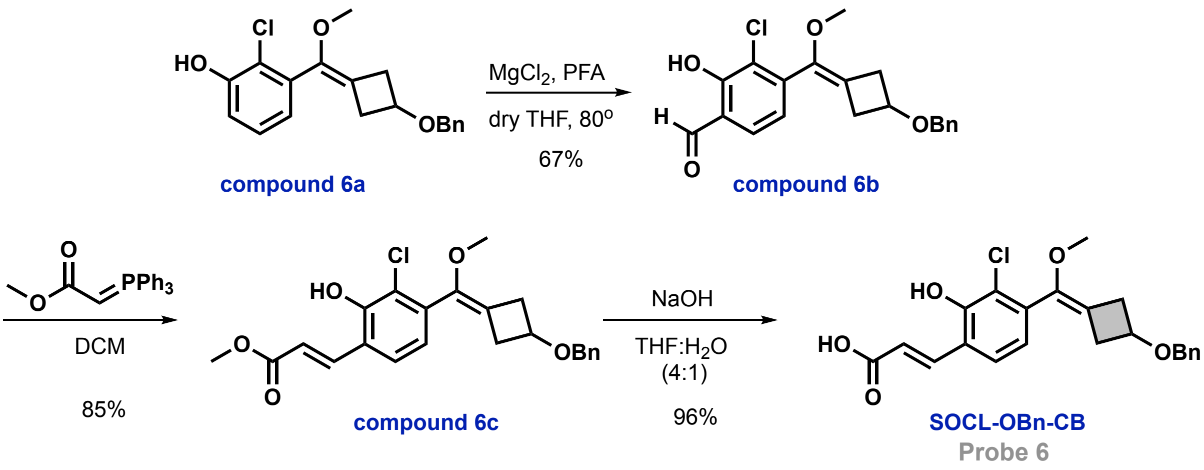

Compound 6b

To a solution of compound **6a**[4] (124 mg, 0.37 mmol) in dry THF (2.5 mL), Et_3N (210 µL, 1.50 mmol), $MgCl_2$ (72 mg, 0.76 mmol) and paraformaldehyde (91 mg, 3.04 mmol) were added following **Procedure B**. The crude residue was purified by column chromatography (80:20, Hex:EtOAc), to afford compound **6b** in the form of a yellow oil (90 mg, 0.25 mmol, 67%).

^{1}H NMR (400 MHz, $CDCl_3$) δ 11.63 (s, 1H), 9.90 (s, 1H), 7.48 (d, *J* = 8.0 Hz, 1H), 7.38 – 7.28 (m, 5H), 7.01 (d, *J* = 8.0 Hz, 1H), 4.46 (s, 2H), 4.22 – 4.12 (m, 1H), 3.56 (s, 3H), 3.27 (ddt, *J* = 15.7, 6.9, 2.6 Hz, 1H), 2.95 (dddd, *J* = 15.7, 6.0, 2.8, 1.4 Hz, 1H), 2.76 – 2.64 (m, 2H).

^{13}C NMR (100 MHz, $CDCl_3$) δ 195.63, 157.79, 145.15, 142.64, 137.91, 130.95, 128.43, 127.88, 127.75, 121.66, 120.43, 114.97, 70.42, 69.39, 57.77, 37.74, 37.20.

MS (ES-): m/z calc. for $C_{20}H_{19}ClO_4$: 358.1; found: 357.4 $[M-H]^-$.

Compound 6c

Compound **6b** (85 mg, 0.24 mmol) was reacted following **Procedure C**. The crude residue was purified by column chromatography (70:30, Hex:EtOAc), to obtain compound **6c** in the form of a white solid (84 mg, 0.20 mmol, 85%).

^{1}H NMR (400 MHz, $CDCl_3$) δ 7.94 (d, *J* = 16.2 Hz, 1H), 7.38 (d, *J* = 8.2 Hz, 1H), 7.36 – 7.28 (m, 5H), 6.90 (d, *J* = 8.0 Hz, 1H), 6.61 (d, *J* = 16.2 Hz, 1H), 6.32 (brs, 1H), 4.45 (s, 2H), 4.20 – 4.11 (m, 1H), 3.83 (s, 3H), 3.53 (s, 3H), 3.25 (ddt, *J* = 15.7, 7.0, 2.0 Hz, 1H), 2.98 – 2.89 (m, 1H), 2.76 – 2.64 (m, 2H).

^{13}C NMR (100 MHz, $CDCl_3$) δ 167.60, 150.67, 145.56, 139.03, 137.90, 135.92, 128.45, 127.91, 127.76, 126.88, 122.52, 122.13, 120.22, 119.86, 113.69, 70.40, 69.40, 57.59, 51.76, 37.68, 37.05.

MS (ES-): m/z calc. for $C_{23}H_{23}ClO_5$: 414.1; found: 413.5 $[M-H]^-$.

Probe 6 – SOCL-OBn-CB

Compound **6c** (25 mg, 0.06 mmol) was dissolved in 320 µL of THF and 80 µL of H_2O and reacted with NaOH (24 mg, 0.6 mmol) according to **procedure D**. The crude residue was purified by column

chromatography (50:50, Hex:EtOAc), to obtain **Probe 6** in the form of a white solid (23 mg, 0.06 mmol, 96%).

^{1}H NMR (400 MHz, $CDCl_3$) δ 8.04 (d, *J* = 16.1 Hz, 1H), 7.41 (d, *J* = 8.1 Hz, 1H), 7.39 – 7.28 (m, 5H), 6.92 (d, *J* = 8.1 Hz, 1H), 6.63 (d, *J* = 16.1 Hz, 1H), 4.46 (s, 2H), 4.22 – 4.12 (m, 1H), 3.54 (s, 3H), 3.29 – 3.21 (m, 1H), 2.98 – 2.89 (m, 1H), 2.77 – 2.62 (m, 2H).

^{13}C NMR (100 MHz, $CDCl_3$) δ 172.11, 150.91, 145.50, 141.09, 137.86, 136.36, 128.45, 127.93, 127.78, 127.13, 122.56, 121.81, 120.29, 119.29, 113.90, 70.41, 69.40, 57.62, 37.70, 37.07.

MS (ES-): m/z calc. for $C_{22}H_{21}ClO_5$: 400.1; found: 399.4 $[M-H]^-$.

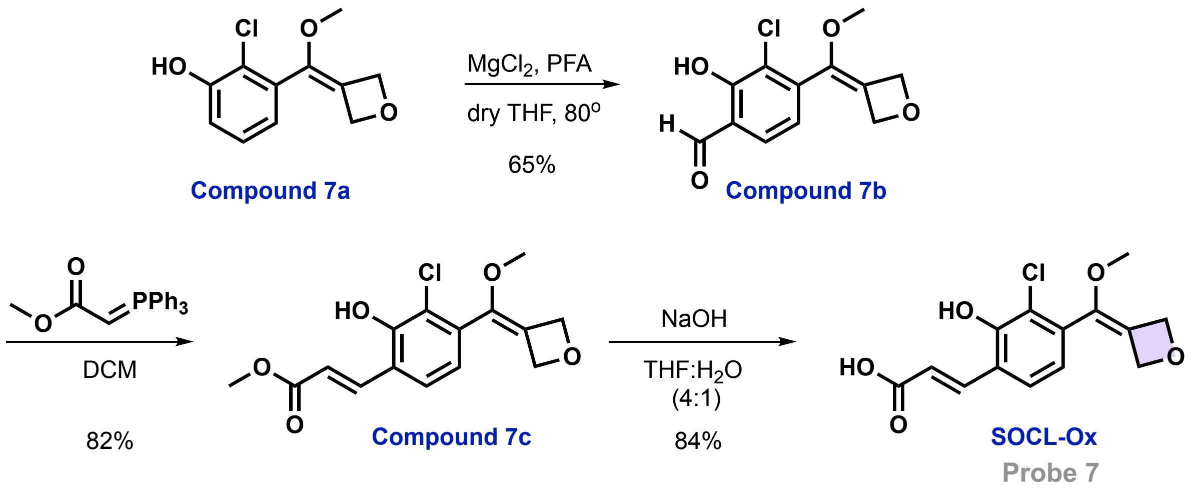

Compound 7b

To a solution of compound **7a**[4] (80 mg, 0.35 mmol) in dry THF (2.5 mL), Et_3N (198 µL, 1.41 mmol), $MgCl_2$ (67 mg, 0.71 mmol) and paraformaldehyde (84 mg, 2.80 mmol) were added following **Procedure B**. The crude residue was purified by column chromatography (30:70, Hex:EtOAc), to afford compound **7b** in the form of a yellow oil (58 mg, 0.23 mmol, 65%).

^{1}H NMR (400 MHz, $CDCl_3$) δ 11.60 (s, 1H), 9.89 (s, 1H), 7.49 (d, *J* = 7.9 Hz, 1H), 7.00 (d, *J* = 7.9 Hz, 1H), 5.53 (t, *J* = 2.6 Hz, 2H), 5.05 (t, *J* = 2.6 Hz, 2H), 3.63 (s, 3H).

^{13}C NMR (100 MHz, $CDCl_3$) δ 195.85, 157.92, 142.37, 141.21, 131.50, 121.78, 121.23, 121.02, 113.46, 77.48, 77.31, 57.30.

MS (ES-): m/z calc. for $C_{12}H_{11}ClO_4$: 254.0; found: 253.1 $[M-H]^-$.

Compound 7c

Compound **7b** (58 mg, 0.23 mmol) was reacted following **Procedure C**. The crude residue was purified by column chromatography (20:80, Hex:EtOAc), to obtain compound **7c** in the form of a white solid (58 mg, 0.19 mmol, 82%).

^{1}H NMR (400 MHz, $CDCl_3$) δ 7.91 (d, *J* = 16.2 Hz, 1H), 7.37 (d, *J* = 8.1 Hz, 1H), 6.88 (d, *J* = 8.0 Hz, 1H), 6.60 (d, *J* = 16.2 Hz, 1H), 6.37 (brs, 1H), 5.52 (t, *J* = 2.5 Hz, 2H), 5.06 (t, *J* = 2.6 Hz, 2H), 3.81 (s, 3H), 3.60 (s, 3H).

^{13}C NMR (100 MHz, $CDCl_3$) δ 167.64, 151.07, 142.85, 138.95, 134.75, 127.26, 123.19, 122.00, 120.39, 120.22, 112.37, 77.62, 77.38, 57.08, 51.93.
MS (ES-): m/z calc. for $C_{15}H_{15}ClO_5$: 310.1; found: 309.3 $[M-H]^-$.

Probe 7 – SOCL-Ox

Compound **7c** (58 mg, 0.19 mmol) was dissolved in 400 μL of THF and 100 μL of H_2O and reacted with NaOH (75 mg, 1.87 mmol) according to **procedure D**. The crude residue was purified by column chromatography (100% EtOAc), to obtain **Probe 7** in the form of a white solid (47 mg, 0.16 mmol, 84%).
^{1}H NMR (400 MHz, DMSO) δ 7.84 (d, *J* = 16.1 Hz, 1H), 7.59 (d, *J* = 8.1 Hz, 1H), 6.88 (d, *J* = 8.0 Hz, 1H), 6.55 (d, *J* = 16.1 Hz, 1H), 5.45 (t, *J* = 2.4 Hz, 2H), 4.91 (t, *J* = 2.5 Hz, 2H), 3.56 (s, 3H).
^{13}C NMR (100 MHz, DMSO) δ 168.09, 152.44, 143.17, 138.60, 135.63, 126.79, 124.77, 121.96, 121.61, 121.01, 110.57, 76.65, 56.92.
MS (ES-): m/z calc. for $C_{14}H_{13}ClO_5$: 296.1; found:295.3 $[M-H]^-$.

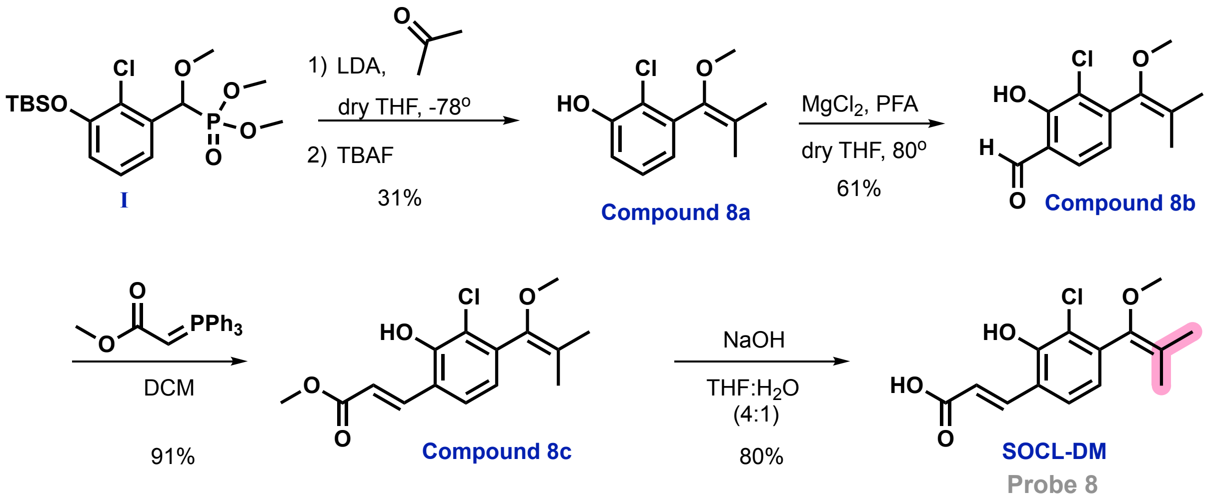

Compound 8a

Compound **8a** was synthesized according to **Procedure A**, using phosphonate **I**[2] (700 mg, 1.77 mmol) in dry THF (6 mL), LDA (2.0 M in THF, 1.33 mL, 2.66 mmol), and dry dimethyl ketone (Acetone) (395 μL, 5.31 mmol). The reaction was monitored by TLC (90:10, Hex:EtOAc). Upon completion, TBAF (1.0 M in THF, 1.95 mL, 1.95 mmol) was added to the solution. The crude residue was purified by column chromatography (90:10, Hex:EtOAc), to afford compound **8a** in the form of a colorless oil (117 mg, 0.55 mmol, 31%).
^{1}H NMR (400 MHz, $CDCl_3$) δ 7.17 (t, *J* = 7.8 Hz, 1H), 7.00 (dd, *J* = 8.1, 1.4 Hz, 1H), 6.85 (dd, *J* = 7.5, 1.4 Hz, 1H), 5.76 (brs, 1H), 3.29 (s, 3H), 1.83 (s, 3H), 1.45 (s, 3H).
^{13}C NMR (100 MHz, $CDCl_3$) δ 151.75, 145.41, 135.23, 127.56, 124.04, 120.75, 116.45, 115.62, 56.67, 19.37, 16.86.
MS (ES-): m/z calc. for $C_{11}H_{13}ClO_2$: 212.1; found: 211.3 $[M-H]^-$.

Compound 8b

To a solution of compound **8a** (100 mg, 0.47 mmol) in dry THF (2.5 mL), Et_3N (264 µL, 1.88 mmol), $MgCl_2$ (89 mg, 0.94 mmol), and paraformaldehyde (113 mg, 3.76 mmol) were added following **Procedure B**. The crude residue was purified by column chromatography (80:20, Hex:EtOAc), to afford compound **8b** in the form of a yellow oil (69 mg, 0.29 mmol, 61%).

^{1}H NMR (400 MHz, $CDCl_3$) δ 11.65 (s, 1H), 9.92 (s, 1H), 7.52 (d, *J* = 7.9 Hz, 1H), 7.02 (d, *J* = 7.9 Hz, 1H), 3.32 (s, 3H), 1.86 (s, 3H), 1.48 (s, 3H).

^{13}C NMR (100 MHz, $CDCl_3$) δ 195.74, 157.58, 144.40, 143.53, 130.91, 123.00, 122.75, 120.37, 118.32, 56.97, 19.23, 16.86.

MS (ES-): m/z calc. for $C_{12}H_{13}ClO_3$: 240.1; found: 239.2 $[M-H]^-$.

Compound 8c

Compound **8b** (50 mg, 0.21 mmol) was reacted following **Procedure C**. The crude residue was purified by column chromatography (85:15, Hex:EtOAc), to obtain compound **8c** in the form of a white solid (56 mg, 0.19 mmol, 91%).

^{1}H NMR (400 MHz, $CDCl_3$) δ 7.96 (d, *J* = 16.2 Hz, 1H), 7.41 (d, *J* = 8.0 Hz, 1H), 6.90 (d, *J* = 8.0 Hz, 1H), 6.63 (d, *J* = 16.2 Hz, 1H), 6.27 (brs, 1H), 3.83 (s, 3H), 3.31 (s, 3H), 1.85 (s, 3H), 1.49 (s, 3H).

^{13}C NMR (100 MHz, $CDCl_3$) δ 167.61, 150.48, 144.96, 139.07, 136.61, 126.80, 123.61, 121.94, 121.54, 119.84, 117.39, 56.77, 51.76, 19.30, 16.82.

MS (ES-): m/z calc. for $C_{15}H_{17}ClO_4$: 296.1; found: 295.2 $[M-H]^-$.

Probe 8 – SOCL-DM

Compound **8c** (50 mg, 0.17 mmol) was dissolved in 400 µL of THF and 100 µL of H_2O and reacted with NaOH (67 mg, 1.68 mmol) according to **procedure D**. The crude residue was purified by column chromatography (50:50, Hex:EtOAc), to obtain **Probe 8** in the form of a white solid (38 mg, 0.14 mmol, 80%).

^{1}H NMR (400 MHz, $CDCl_3$) δ 8.07 (d, *J* = 16.1 Hz, 1H), 7.45 (d, *J* = 8.0 Hz, 1H), 6.92 (d, *J* = 8.0 Hz, 1H), 6.67 (d, *J* = 16.1 Hz, 1H), 3.32 (s, 3H), 1.85 (s, 3H), 1.49 (s, 3H).

^{13}C NMR (100 MHz, $CDCl_3$) δ 172.49, 150.77, 144.90, 141.23, 137.08, 127.09, 123.67, 121.65, 119.30, 117.58, 56.81, 19.31, 16.83.

MS (ES-): m/z calc. for $C_{14}H_{15}ClO_4$: 282.1; found: 281.3 $[M-H]^-$.

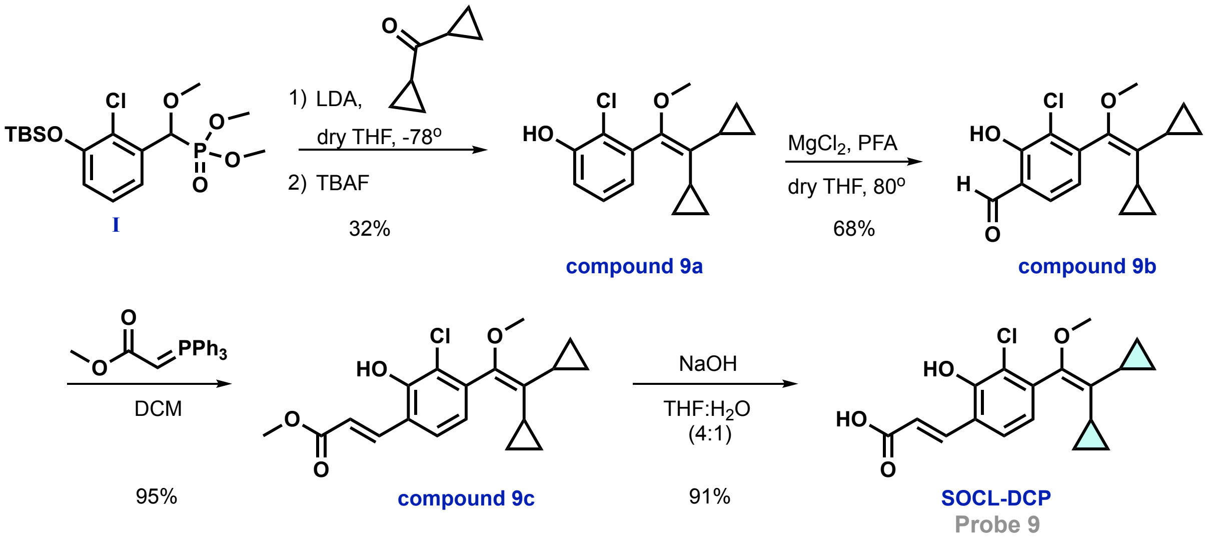

Compound 9a

Compound **9a** was synthesized according to **Procedure A**, using phosphonate I[2] (400 mg, 1.01 mmol) in dry THF (3 mL), LDA (2.0 M in THF, 0.76 mL, 1.52 mmol), and Dicyclopropyl ketone (195 μL, 1.72 mmol). The reaction was monitored by TLC (90:10, Hex:EtOAc). Upon completion, TBAF (1.0 M in THF, 1.11 mL, 1.11 mmol) was added to the solution. The crude residue was purified by column chromatography (90:10, Hex:EtOAc), to afford compound **9a** in the form of a colorless oil (86 mg, 0.32 mmol, 32%).

^{1}H NMR (400 MHz, $CDCl_3$) δ 7.18 (t, *J* = 7.8 Hz, 1H), 7.01 (dd, *J* = 8.1, 1.3 Hz, 1H), 6.90 (dd, *J* = 7.5, 1.4 Hz, 1H), 5.76 (brs, 1H), 3.33 (s, 3H), 1.78 (ddd, *J* = 14.2, 8.5, 5.8 Hz, 1H), 0.99 (ddd, *J* = 14.0, 8.3, 5.8 Hz, 1H), 0.80 (d, *J* = 3.7 Hz, 2H), 0.74 – 0.68 (m, 2H), 0.30 (d, *J* = 7.2 Hz, 2H), 0.08 (d, *J* = 4.3 Hz, 2H).

^{13}C NMR (100 MHz, $CDCl_3$) δ 151.45, 148.74, 135.76, 127.24, 124.11, 120.57, 117.68, 115.33, 56.58, 11.01, 10.52, 5.18, 4.93.

MS (ES-): m/z calc. for $C_{15}H_{17}ClO_2$: 264.1; found: 263.3 $[M-H]^-$.

Compound 9b

To a solution of compound **9a** (70 mg, 0.26 mmol) in dry THF (2 mL), Et_3N (145 μL, 1.04 mmol), $MgCl_2$ (49 mg, 0.52 mmol), and paraformaldehyde (62 mg, 2.08 mmol) were added following **Procedure B**. The crude residue was purified by column chromatography (80:20, Hex:EtOAc), to afford compound **9b** in the form of a yellow oil (52 mg, 0.18 mmol, 68%).

^{1}H NMR (400 MHz, $CDCl_3$) δ 11.64 (s, 1H), 9.90 (s, 1H), 7.48 (d, *J* = 8.0 Hz, 1H), 7.03 (d, *J* = 8.0 Hz, 1H), 3.35 (s, 3H), 1.88 – 1.77 (m, 1H), 1.06 – 0.98 (m, 1H), 0.82 (brs, 2H), 0.72 (d, *J* = 8.5 Hz, 2H), 0.32 (brs, 2H), 0.03 (brs, 2H).

^{13}C NMR (100 MHz, $CDCl_3$) δ 195.82, 157.61, 147.99, 144.40, 130.71, 126.75, 123.13, 123.02, 120.33, 57.23, 11.52, 10.52, 5.44, 5.25.

MS (ES-): m/z calc. for $C_{16}H_{17}ClO_3$: 292.1; found: 291.3 $[M-H]^-$.

Compound 9c

Compound **9b** (45 mg, 0.15 mmol) was reacted following **Procedure C**. The crude residue was purified by column chromatography (80:20, Hex:EtOAc), to obtain compound **9c** in the form of a white solid (51 mg, 0.14 mmol, 95%).

^{1}H NMR (400 MHz, $CDCl_3$) δ 7.94 (d, *J* = 16.2 Hz, 1H), 7.38 (d, *J* = 8.1 Hz, 1H), 6.92 (d, *J* = 8.0 Hz, 1H), 6.62 (d, *J* = 16.2 Hz, 1H), 6.23 (brs, 1H), 3.81 (s, 3H), 3.32 (s, 3H), 1.78 (ddd, *J* = 14.1, 8.4, 5.6 Hz, 1H), 1.02 – 0.95 (m, 1H), 0.79 (d, *J* = 3.6 Hz, 2H), 0.71 (d, *J* = 8.5 Hz, 2H), 0.31 (d, *J* = 7.6 Hz, 2H), 0.06 (d, *J* = 5.1 Hz, 2H).

^{13}C NMR (100 MHz, $CDCl_3$) δ 167.75, 150.47, 148.46, 139.23, 137.47, 126.72, 125.57, 123.91, 121.87, 121.60, 119.87, 56.99, 51.88, 11.32, 10.63, 5.48, 5.17.

MS (ES-): m/z calc. for $C_{19}H_{21}ClO_4$: 348.1; found: 347.4 $[M-H]^-$.

Probe 9 – SOCL-DCP

Compound **9c** (40 mg, 0.11 mmol) was dissolved in 400 μL of THF and 100 μL of H_2O and reacted with NaOH (46 mg, 1.14 mmol) according to **procedure D**. The crude residue was purified by column chromatography (30:70, Hex:EtOAc), to obtain **Probe 9** in the form of a white solid (34 mg, 0.10 mmol, 91%).

^{1}H NMR (400 MHz, $CDCl_3$) δ 8.05 (d, *J* = 16.1 Hz, 1H), 7.41 (d, *J* = 8.1 Hz, 1H), 6.94 (d, *J* = 8.0 Hz, 1H), 6.64 (d, *J* = 16.1 Hz, 1H), 3.33 (s, 3H), 1.79 (ddd, *J* = 14.1, 8.5, 5.7 Hz, 1H), 0.99 (ddd, *J* = 14.2, 8.5, 6.0 Hz, 1H), 0.80 (d, *J* = 4.8 Hz, 2H), 0.71 (d, *J* = 8.5 Hz, 2H), 0.32 (d, *J* = 8.1 Hz, 2H), 0.06 (d, *J* = 5.1 Hz, 2H).

^{13}C NMR (100 MHz, $CDCl_3$) δ 172.35, 150.73, 148.41, 141.30, 137.93, 126.96, 125.76, 123.96, 121.69, 121.58, 119.33, 57.03, 11.36, 10.62, 5.51, 5.19.

MS (ES-): m/z calc. for $C_{18}H_{19}ClO_4$: 334.1; found: 333.4 $[M-H]^-$.

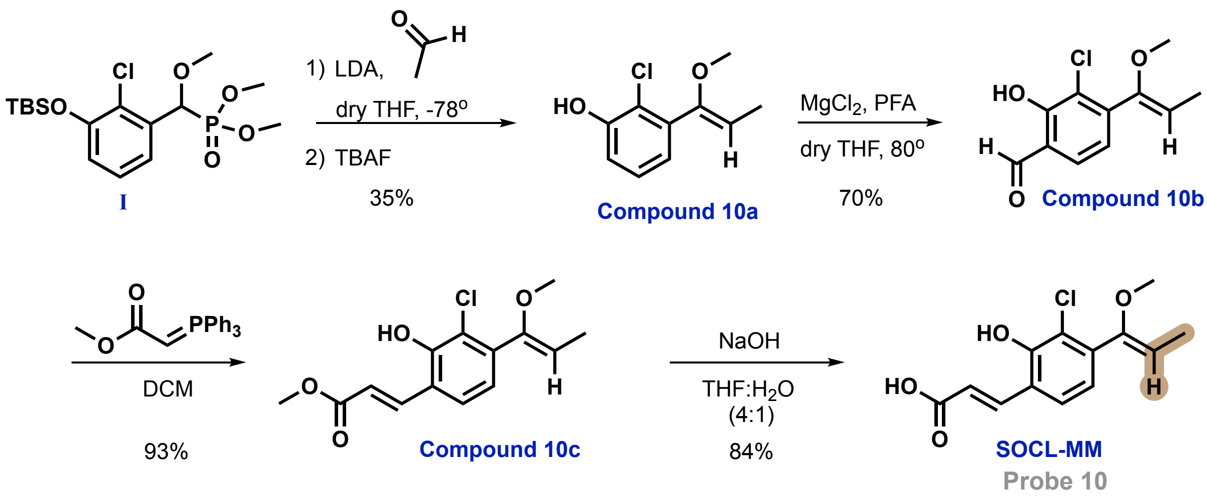

Compound 10a

Compound **10a** was synthesized according to **Procedure A**, using phosphonate **I**[2] (600 mg, 1.52 mmol) in dry THF (5 mL), LDA (2.0 M in THF, 1.14 mL, 2.28 mmol), and Acetaldehyde (171 µL, 3.04 mmol). The reaction was monitored by TLC (90:10, Hex:EtOAc). Upon completion, TBAF (1.0 M in THF, 1.67 mL, 1.67 mmol) was added to the solution. The crude residue was purified by column chromatography (90:10, Hex:EtOAc), to afford compound **10a** in the form of a colorless oil (105 mg, 0.53 mmol, 35%). Compound **10a** was obtained as a diastereomeric mixture of *trans* and *cis* (E:Z) in 2:1 ratio, respectively.

^{1}H NMR (400 MHz, $CDCl_3$) (major) δ 7.19 (t, *J* = 8.0 Hz, 1H), 7.02 (dd, *J* = 8.2, 1.5 Hz, 1H), 6.88 (dd, *J* = 7.5, 1.5 Hz, 1H), 5.88 (brs, 1H), 4.91 (q, *J* = 6.9 Hz, 1H), 3.66 (s, 3H), 1.48 (d, *J* = 6.9 Hz, 3H).

^{1}H NMR (400 MHz, $CDCl_3$) (minor) δ 7.16 (t, *J* = 8.0 Hz, 1H), 7.01 (dd, *J* = 8.2, 1.6 Hz, 1H), 6.93 (dd, *J* = 7.5, 1.5 Hz, 1H), 5.88 (brs, 1H), 4.95 (q, *J* = 6.9 Hz, 1H), 3.40 (s, 3H), 1.80 (d, *J* = 6.8 Hz, 3H).

^{13}C NMR (100 MHz, $CDCl_3$) (Diastereomeric mixture) δ [153.13, 152.23], [151.74, 151.64], [135.89, 135.79], [127.63, 127.55], [123.15, 122.99], [119.69, 119.05], [115.88, 115.78], [109.49, 96.08], [56.80, 55.15], [12.24, 10.54].

MS (ES-): m/z calc. for $C_{10}H_{11}ClO_2$: 198.0; found: 197.2 $[M-H]^-$.

Compound 10b

To a solution of compound **10a** (60 mg, 0.30 mmol) in dry THF (2 mL), Et_3N (169 µL, 1.20 mmol), $MgCl_2$ (57 mg, 0.60 mmol) and paraformaldehyde (72 mg, 2.40 mmol) were added following **Procedure B**. The crude residue was purified by column chromatography (80:20, Hex:EtOAc), to afford compound **10b** in the form of a yellow oil (47 mg, 0.21 mmol, 70%). Compound **10b** was obtained as a diastereomeric mixture of *trans* and *cis* (E:Z) in 2:1 ratio, respectively.

^{1}H NMR (400 MHz, $CDCl_3$) (major) δ 11.61 (s, 1H), 9.92 (s, 1H), 7.52 (d, *J* = 7.9 Hz, 1H), 7.01 (d, *J* = 7.9 Hz, 1H), 4.95 (q, *J* = 7.0 Hz, 1H), 3.68 (s, 3H), 1.48 (d, *J* = 7.0 Hz, 3H).

^{1}H NMR (400 MHz, $CDCl_3$) (minor) δ 11.61 (s, 1H), 9.91 (s, 1H), 7.49 (d, *J* = 8.0 Hz, 1H), 7.08 (d, *J* = 8.0 Hz, 1H), 5.16 (q, *J* = 6.9 Hz, 1H), 3.43 (s, 3H), 1.83 (d, *J* = 6.9 Hz, 3H).

^{13}C NMR (100 MHz, $CDCl_3$) (Diastereomeric mixture) δ [195.86, 195.64], 157.65, [152.24, 151.40], 143.61, [131.17, 131.08], [129.75, 129.49], [122.10, 121.71], [120.64, 120.39], [112.61, 96.70], [57.29, 55.38], [12.15, 10.71].

MS (ES-): m/z calc. for $C_{11}H_{11}ClO_3$: 226.0; found: 225.2 $[M-H]^-$.

Compound 10c

Compound **10b** (47 mg, 0.21 mmol) was reacted following **Procedure C**. The crude residue was purified by column chromatography (80:20, Hex:EtOAc), to obtain compound **10c** in the form of a white solid (54 mg, 0.19 mmol, 93%). Compound **10c** was obtained as a diastereomeric mixture of *trans* and *cis* (E:Z) in 2:1 ratio, respectively.

^{1}**H NMR** (400 MHz, $CDCl_3$) (major) δ 7.95 (d, *J* = 16.2 Hz, 1H), 7.41 (d, *J* = 8.0 Hz, 1H), 6.90 (d, *J* = 8.0 Hz, 1H), 6.62 (d, *J* = 16.2 Hz, 1H), 4.92 (q, *J* = 6.9 Hz, 1H), 3.83 (s, 3H), 3.66 (s, 3H), 1.49 (d, *J* = 6.9 Hz, 3H).

^{1}**H NMR** (400 MHz, $CDCl_3$) (minor) δ 7.95 (d, *J* = 16.2 Hz, 1H), 7.39 (d, *J* = 8.3 Hz, 1H), 6.96 (d, *J* = 8.1 Hz, 1H), 6.62 (d, *J* = 16.2 Hz, 1H), 5.04 (q, *J* = 6.9 Hz, 1H), 3.83 (s, 3H), 3.41 (s, 3H), 1.80 (d, *J* = 6.9 Hz, 3H).

^{13}**C NMR** (100 MHz, $CDCl_3$) (Diastereomeric mixture) δ 167.60, [152.65, 151.89], [150.58, 150.50], [139.10, 139.02], [137.12, 137.08], [129.75, 129.49], [127.00, 126.94], [122.76, 122.64], [122.28, 122.12], [119.95, 119.83], [110.95, 96.49], [57.09, 55.24], 51.75, [12.27, 10.63].

MS (ES-): m/z calc. for $C_{14}H_{15}ClO_4$: 282.1; found: 281.3 $[M-H]^-$.

Probe 10 – SOCL-MM

Compound **10c** (50 mg, 0.18 mmol) was dissolved in 400 μL of THF and 100 μL of H_2O and reacted with NaOH (71 mg, 1.77 mmol) according to **procedure D**. The crude residue was purified by column chromatography (30:70, Hex:EtOAc), to obtain **Probe 10** in the form of a white solid (41 mg, 0.15 mmol, 84%). **Probe 10** was obtained as a diastereomeric mixture of *trans* and *cis* (E:Z) in 2:1 ratio, respectively.

^{1}**H NMR** (400 MHz, $CDCl_3$) (major) δ 8.06 (d, *J* = 16.1 Hz, 1H), 7.45 (d, *J* = 8.2 Hz, 1H), 6.93 (d, *J* = 8.0 Hz, 1H), 6.66 (d, *J* = 16.1 Hz, 1H), 4.94 (q, *J* = 7.0 Hz, 1H), 3.66 (s, 3H), 1.50 (d, *J* = 7.0 Hz, 3H).

^{1}**H NMR** (400 MHz, $CDCl_3$) (minor) δ 8.06 (d, *J* = 16.1 Hz, 1H), 7.42 (d, *J* = 8.3 Hz, 1H), 6.98 (d, *J* = 8.1 Hz, 1H), 6.65 (d, *J* = 16.1 Hz, 1H), 5.06 (q, *J* = 6.8 Hz, 1H), 3.42 (s, 3H), 1.81 (d, *J* = 6.9 Hz, 3H).

^{13}**C NMR** (100 MHz, $CDCl_3$) (Diastereomeric mixture) δ 172.42, [152.58, 151.84], [150.81, 150.74], [141.26, 141.18], [137.57, 137.52], [127.30, 127.24], [122.84, 122.68], [121.94, 121.77], [120.75, 119.83], [119.39, 119.26], [111.19, 96.59], [57.14, 55.26], [12.27, 10.66].

MS (ES-): m/z calc. for $C_{13}H_{13}ClO_4$: 268.1; found: 267.3 $[M-H]^-$.

Characterization of the 'ene'-Product from oxidation of probe SOCL-DM

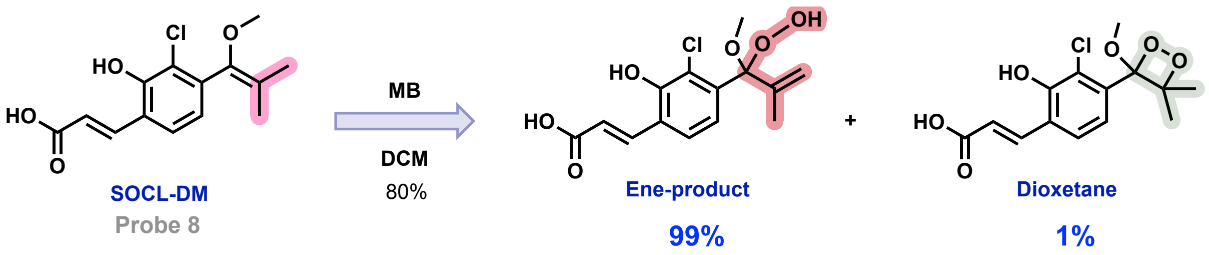

'Ene'-product

SOCL-DM probe (15 mg, 0.05 mmol) was dissolved in 5 mL of DCM followed by the addition of a catalytic amount of methylene blue. Then, oxygen was bubbled through the solution while irradiating with yellow light. The reaction was monitored by RP-HPLC (gradient of 30-100% ACN, 0.1% TFA). Upon completion (about 5 min) the Ene-product was obtained in 99% conversion. Then, the solvent was removed under reduced pressure and the crude product was purified by column chromatography (30:70, Hex:EtOAc) to afford **'ene'-product** in the form of a white solid (13.4 mg, 80% yield).

^{1}H NMR (400 MHz, $CDCl_3$) δ 8.03 (d, *J* = 16.1 Hz, 1H), 7.47 – 7.41 (m, 2H), 6.64 (d, *J* = 16.1 Hz, 1H), 5.45 (s, 1H), 5.30 (s, 1H), 3.35 (s, 3H), 1.59 (s, 3H).

^{13}C NMR (100 MHz, $CDCl_3$) δ 172.15, 151.17, 141.25, 138.96, 137.90, 126.89, 122.31, 121.55, 119.73, 118.84, 117.88, 106.12, 49.89, 19.27.

HRMS (ES-): m/z calc. for $C_{14}H_{15}ClO_6$: 314.06; found: 313.05 $[M-H]^-$.

Experimental protocols

Chemiluminescent kinetic assays of SOCL Probes 1-10 for $^1O_2$ from Endo-Peroxide (EP-1)

Stock solutions of SOCL **Probe 1-10** and EP-1 were prepared in DMSO at a concentration of 10 mM. Chemiluminescent kinetic profiles were recorded using Spectramax iD3 plate reader at 37°C with endo-peroxide (EP-1) serving as the $^1O_2$ donor. Measurements were conducted in a lidded white 96-well Corning™ plate, each well contained 90 μL of PBS (100 mM, pH 7.4), 5μL of **Probe 1-10** [2 mM], and 5μL of EP-1 [10 mM] with a final volume of 100 μL. The final concentrations of SOCL **probe 1-10** and EP-1 are 100 μM and 500 μM, respectively. SOCL **Probes 1-10** were pre-incubated for 30 minutes in PBS prior to the addition of EP-1.

Selectivity assay of probes SOCL-AD, SOCL-CB, and SOCL-DM to different ROS

The selectivity of probes SOCL-AD, SOCL-CB, and SOCL-DM was assessed in the presence of eight reactive oxygen species ($^1O_2$, $ONOO^-$, H_2O_2, ClO^-, TBHP, TBO·, OH·, O_2^-). EP-1 was used as $^1O_2$ donor. Stock solutions of the analytes were prepared at a concentration of 50 mM according to previous reports.[5-7] Fresh Solutions were prepared immediately before measurements. To a 96-well plate containing the SOCL probes in a concentration of 100 μM in PBS (100 mM, pH 7.4) at a volume of 99 μL, 1 μL of each analyte was added to achieve a final concentration of 500 μM. The chemiluminescence intensity was then measured.

LOD experiment procedure for EP-1 and conversion to $^1O_2$ concentrations

For the limit of detection assay, a serial of 1:5 dilutions of EP-1 concentrations starting from 1 mM in DMSO were prepared. Next, 5 μL of each EP-1 concentration was added to a 96-well plate loaded with pre-incubated SOCL probes [10 μM] or SOSG fluorescent probe [10 μM] in PBS (pH 7.4, 5% DMSO) to achieve the final EP-1 concentration of [50 μM - 1.28×10^{-4} μM]. The total light emission of SOCL probes was measured at 37°C and the LOD for EP-1 was determined according to the standard method (blank + 3 SD).

<u>Conversion to $^1O_2$ concentrations</u>: The quantification of $^1O_2$ concentrations was done according to previous reports[1, 8, 9]. EP-1 undergoes **first-order** decomposition with a rate constant (*k*) of 4.16×10^{-4} s^{-1} at 37°C, following the equation:

$$[\text{EP-1}]_{decomposed}=1-[\text{EP-1}]_{initial}\cdot e^{-kt}$$

where (*t*) represents time in seconds. Under these conditions, 78% of EP-1 decomposes within <u>60 minutes</u>. Given that EP-1 generates $^1O_2$ with an 82% yield, this corresponds to the production of 636 nM $^1O_2$ from an initial 1 μM EP-1 concentration. To ensure consistency in data interpretation, the limit of detection (LOD) values for all probes were determined at the 60-minute timepoint.

For extended reaction times, EP-1 decomposition continues according to the same first-order kinetics. For example, at 120 minutes, 95% of EP-1 decomposes with the same 82% yield, resulting in 779 nM $^1O_2$ generated per 1 μM of initial EP-1. As a result, the probe's signals upon incubation with EP-1 increase proportionally with reaction time due to higher $^1O_2$ formation. This relationship is shown in Figure **S14**.

Chemiluminescent kinetic assays of SOCL Probes 1-10 for $^1O_2$ from Methylene blue (MB)

Chemiluminescent kinetic profiles were recorded using Spectramax iD3 at room temperature, whereas MB serves as a photosensitizer for producing $^1O_2$ under light irradiation. The measurement settings were fixed on the following parameters: Integration time of 10 msec and measuring interval time of 8 sec. Measurements were conducted in a white 96-well Corning™ plate, each well contained 98 μL of PBS (100 mM, pH 7.4), 1 μL of SOCL **Probe 1-10** [10 mM], and 1 μL of MB [1 mM] to obtain a final concentration of 100 μM of tested probes and 10 μM of MB. Next, LED PAR38 lamp (19W, 3000K) was utilized, and the plate was irradiated for 5 sec, followed by an immediate measure of the chemiluminescent kinetic profile for each probe. All SOCL Probes 1-10 were pre-incubated for 30 minutes in PBS pH 7.4 prior to the addition of MB.

Determination of oxidation rate of probes SOCL-AD, SOCL-CB, and SOCL-DM

Adamantyl-, cyclobutyl-, and dimethyl- enol ethers (30 μL) were dissolved in 1 mL of Acetate buffer (100 mM, pH 4.2) followed by the addition of 30 μL of MB to obtain final concentrations of [300 μM]. Then, the solutions were irradiated for 10 minutes, and the reaction was analyzed by RP-HPLC (30-100% ACN in water with 0.1% TFA). The oxidation rate was determined as the ratio of the amount of oxidation products—dioxetane, benzoate, and 'ene' product—to the initial amount of the enol ether (starting material). This ratio was calculated by integrating the peak areas corresponding to these compounds in the RP-HPLC at 330 nm.

Detection of Horseradish peroxidase (HRP) activity in producing $^1O_2$ using H_2O_2 substrate

Measurements were conducted in a lidded white 96-well plate, each well contained 97 μL of PB (50 mM, pH 6.0), and 1 μL of **SOCL probes** [10 mM] to obtain a final concentration of 100 μM. The plate was incubated for 30 minutes at 30°C. Subsequently, 1 μL of horseradish peroxidase (HRP, 1 mg/mL) and 1 μL of hydrogen peroxide (H_2O_2, 10 mM) were added, resulting in final concentrations of 10 μg/mL for HRP and 100 μM for H_2O_2. The chemiluminescence kinetic profile was then measured.

LOD experiment procedure for $^1O_2$ detection from HRP using various H_2O_2 Concentrations

To determine the limit of detection (LOD), a serial of 1:5 dilution of H_2O_2 concentrations starting from 10 mM in PB (50 mM, pH 6.0) was prepared. Measurements were performed in a white 96-well plate, each well containing 93 µL of phosphate buffer (50 mM, pH 6.0) and 1 µL of SOCL probes (10 mM), resulting in a final probe concentration of 100 µM. After a 30-minute incubation at 30°C, 1 µL of horseradish peroxidase (HRP, 1 mg/mL) was added to achieve a final HRP concentration of 10 µg/mL. Subsequently, 5 µL of H_2O_2 solutions were added to obtain final concentrations [500 µM - 0.16 µM], and the total emitted light after 300 minutes was calculated to evaluate the LOD. LOD for H_2O_2 was determined according to the standard method (blank + 3 SD).

<u>$^1O_2$ LOD calculations of SOCL-DM probe</u>

To determine the LOD for $^1O_2$ concentration using probe SOCL-DM, we first generated a calibration curve. This was achieved by measuring the total light emission (TLE) of probe SOCL-DM in the presence of various concentrations of EP-1. Since EP-1 concentration is directly related to singlet oxygen production, and there is a linear correlation between EP-1 concentration and TLE, the calibration curve plots TLE against $^1O_2$ concentration after 60 minutes. The LOD value for $\mathbf{^1O_2}$ concentration produced by HRP and H_2O_2 was extrapolated from the slope of the calibration curve, using the TLE value obtained at the LOD of hydrogen peroxide (H_2O_2) concentration.

IVIS-visualization

As described above, a pre-loaded 96-well plate with 98 µL of SOCL probes [100 µM] in PB (50 mM, pH 6.0) was prepared. Then, 5 µL of HRP (1 mg/mL) and H_2O_2 (10 mM) were added to obtain final concentration of 50 µg/mL and 500 µM, respectively. Next, the chemiluminescence emission spectra of the probes were measured using IVIS® Lumina imager. The images were taken after 20 minutes of incubation with an exposure time parameter set at 50 sec.

Bacterial experiments

Detection of $^1O_2$ produced in bacteria cells by SOCL-DM, SOCL-CB and SOCL-AD probes

Bacillus subtilis ATCC 14945 was purchased from the American Type Culture Collection and cultured in LB medium at 30°C for 24 hours with shaking. The bacterial cultures were diluted with LB medium to obtain OD_{600} of 0.85. The bacterial suspension was then divided into 4 different aliquots, each containing 1 mL of the suspension.

The bacteria were incubated under four conditions: (1) bacteria alone, (2) bacteria with SOCL probe [100 µM], (3) bacteria with methylene blue (MB) [50 µM], and (4) bacteria with both SOCL probe and MB, all with 1% DMSO in LB. For appropriate samples, 10 µL of SOCL probe (from 10 mM stock) or 10 µL of methylene blue (MB) (from 5 mM stock) was added to achieve final concentrations of 100 µM and 10 µM, respectively. After 3 hours of incubation, the bacteria were centrifuged, and the bacterial pellet was washed twice with PBS (pH 7.4) (at 5000 rpm, 5 minutes), followed by resuspension in 1 mL of PBS (pH 7.4).

A 96-well lidded clear plate (Corning) was utilized, with each well pre-loaded with 100 µL of each of the four combinations for each probe under identical conditions. The plate was subjected to irradiation using LED PAR38 lamp (19W, 3000K) for 1 minute. The chemiluminescence signal was monitored using Molecular Devices Spectramax iD3 over the course of 15 minutes of incubation at 37°C.

Cytotoxicity evaluation following treatments and irradiation

Bacillus subtilis ATCC 14945 was cultured in LB at 30°C for 24 hours with shaking. The initial culture of the bacteria was centrifuged (at 5000 rpm, 5 minutes), washed with PBS (pH 7.4), and the bacterial pellets were resuspended in PBS (pH 7.4) to obtain OD_{600} of 0.85. Next, 100 µL of bacterial suspension were added to a 96-well plate (Corning). Bacterial cells were treated with SOCL probes [100 µM], and MB [10 µM] or both, and irradiated for 5 minutes. Control wells without SOCL probes and MB containing bacterial cells were also prepared. The amount of viable cells was assessed by modified 3-(4,5-dimethylthiazol-2-yl)-2,5-diphenyltetrazolinium bromide (MTT) assay. After 5 minutes of irradiation, the cells were centrifuged (at 4000 rpm, 8 minutes) and washed with PBS. The cells were resuspended in PBS, and MTT solution in PBS (100 µL of 1 mg/mL) was added to the wells to achieve a final concentration of 0.5 mg/mL. The cells were incubated for 4 hours at 30°C. The 96-well plate was then centrifuged, and the medium was replaced with 100 µL of isopropanol containing 5% HCl 1M to dissolve the formazan crystals formed. The plate was incubated for 16 hours at room temperature. Absorbance of the solution was measured at 570 nm by a Tecan pro 200 plate reader. The percentage of viable cells was normalized to the viability of non-treated cells (100% viability).

Appendix I- Computational data

The electronic structures of cyclobutyl-, oxetanyl-, methyl-, and dimethyl-enol ethers were studied by DFT calculations performed at the wB97XD/def2-TZVP level of theory. The molecular orbital analysis reveals that the calculated HOMO-LUMO energy gap in cyclobutyl-enol ether (**SOCL-CB**) (7.476 eV) is smaller than its electron-withdrawing analogue – oxetanyl-enol ether (**SOCL-Ox**) (7.553 eV) with an energy difference of 1.77 kcal/mol. A similar trend is observed when comparing the HOMO-LUMO energy gap of methyl-enol ether (**SOCL-MM**) (7.664 eV) and dimethyl-enol ether (**SOCL-DM**) (7.522 eV) with a 3.27 kcal/mol energy difference. The smaller HOMO-LUMO gap suggests higher reactivity of cyclobutyl-, and dimethyl-enol ethers compared to oxetanyl- and methyl-enol ether, respectively.

Moreover, the oxidation potential for both oxetanyl- and cyclobutyl-enol ethers were calculated using uwB97XD/def2-TZVP level of theory. A spontaneous reaction with a ΔG = -0.01379 [Hartree] (**Table S1**) suggests that cyclobutyl-enol ether (**SOCL-CB**) possesses higher HOMO energy compared to oxetanyl-enol ether (**SOCL-Ox**), thus indicating a more electron-donating character.

Computational methods

DFT calculations were performed using Gaussian 09.[10] Geometry optimization of all the molecules, were carried out using the wB97XD/def2-TZVP and uwB97XD/def2-TZVP level of theory.[11-15] Thermal energy corrections were extracted from the results of frequency analysis performed at the same level of theory. Frequency analysis of all the molecules contained no imaginary frequency showing that these are energy minima. The computational pictures were generated using Avagadro.[16] The Cartesian coordinates and energies of all optimized molecules are provided.

Calculation of HOMO-LUMO energy gap

DFT calculations were performed at the wB97XD/def2-TZVP level of theory.

Oxytanyl-enol ether (SOCL-Ox)

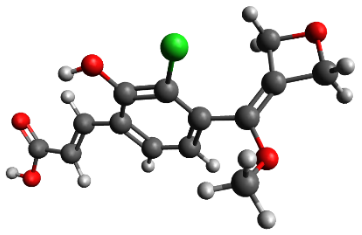

C	0.82697	-0.94406	0.40332
C	-0.54727	-0.68770	0.47729
C	-1.07246	0.52109	0.03848

C	-0.19542	1.49200	-0.44993
C	1.15387	1.24580	-0.52931
C	1.69235	0.02529	-0.11697
H	-0.60340	2.43524	-0.78744
H	1.81165	1.99237	-0.95485
O	1.25758	-2.13138	0.86964
H	2.21596	-2.15370	0.90575
C	-2.52103	0.84814	0.06829
O	-2.88053	1.96985	0.76714
C	-2.49821	1.97309	2.13153
H	-2.86726	2.90485	2.55449
H	-2.94681	1.12736	2.66080
H	-1.41082	1.93063	2.24544
C	-3.43224	0.21797	-0.64827
C	-3.46061	-0.90426	-1.64438
H	-3.25508	-1.90160	-1.24475
H	-2.87886	-0.74901	-2.55843
Cl	-1.55394	-1.90322	1.18541
C	3.12041	-0.25971	-0.24047
C	4.10553	0.63552	-0.20332
C	5.50314	0.19542	-0.38162
O	5.86866	-0.93986	-0.54228
O	6.35667	1.23240	-0.33785
H	7.24417	0.87263	-0.45651
H	3.42695	-1.29118	-0.40533
H	3.93341	1.69025	-0.03386
C	-4.90240	0.38362	-0.89127
H	-5.21022	1.33353	-1.33772
H	-5.55307	0.15331	-0.04188
O	-4.86805	-0.68642	-1.85388

Sum of electronic and zero-point Energies= -1377.372422

Sum of electronic and thermal Energies=	-1377.352953
Sum of electronic and thermal Enthalpies=	-1377.352009
Sum of electronic and thermal Free Energies=	-1377.422633

Cyclobutyl-enol ether (SOCL-CB)

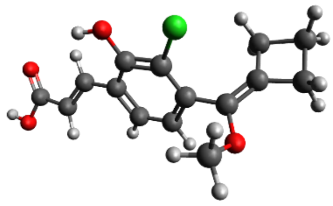

C	0.82718	-0.93113	0.40490
C	-0.54531	-0.66582	0.47170
C	-1.06504	0.53896	0.01572
C	-0.18023	1.49865	-0.48120
C	1.16828	1.24587	-0.55221
C	1.69966	0.02755	-0.12352
H	-0.58269	2.43979	-0.83110
H	1.83144	1.98439	-0.98365
O	1.25099	-2.11568	0.88674
H	2.20901	-2.13962	0.92861
C	-2.51263	0.87338	0.04571
O	-2.86402	1.97164	0.78932
C	-2.49567	1.91128	2.15430
H	-2.85084	2.83150	2.61362
H	-2.96330	1.05262	2.64556
H	-1.41004	1.84365	2.27758
C	-3.42434	0.27329	-0.70041
C	-3.38599	-0.86123	-1.69675
H	-2.91134	-0.59231	-2.64272
H	-2.94505	-1.79337	-1.34269
Cl	-1.56084	-1.86664	1.19379
C	3.12629	-0.26520	-0.23801

C	4.11628	0.62567	-0.21239
C	5.51147	0.17608	-0.37891
O	5.87324	-0.96282	-0.52350
O	6.37107	1.20920	-0.34434
H	7.25662	0.84199	-0.45407
H	3.42905	-1.30048	-0.38544
H	3.94895	1.68362	-0.05925
C	-4.91343	0.46423	-0.84852
H	-5.18445	1.38007	-1.37711
H	-5.48723	0.42339	0.07788
C	-4.93827	-0.80981	-1.73663
H	-5.38172	-0.68841	-2.72329
H	-5.40396	-1.66330	-1.24666

Sum of electronic and zero-point Energies=	-1341.452028
Sum of electronic and thermal Energies=	-1341.432294
Sum of electronic and thermal Enthalpies=	-1341.431350
Sum of electronic and thermal Free Energies=	-1341.502378

Monomethyl-enol ether (SOCL-MM)

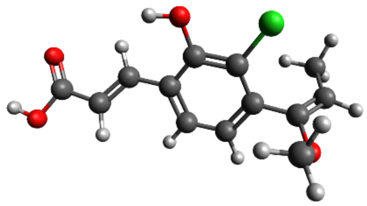

C	0.28858	0.96793	-0.21929
C	-1.08753	0.71413	-0.19113
C	-1.58044	-0.51533	0.22536
C	-0.67074	-1.50652	0.59764
C	0.68162	-1.26367	0.57990
C	1.18986	-0.02602	0.17987
H	-1.05349	-2.46621	0.91783

H	1.36944	-2.02881	0.91553
O	0.68320	2.17798	-0.65980
H	1.63559	2.20330	-0.77129
C	-3.03113	-0.84932	0.25983
O	-3.40773	-1.87693	-0.56687
C	-3.09961	-1.69549	-1.93606
H	-3.47419	-2.57468	-2.45605
H	-3.59004	-0.80028	-2.33036
H	-2.02091	-1.61299	-2.10169
C	-3.91913	-0.32776	1.09572
C	-3.64554	0.69464	2.14764
H	-4.15161	1.63509	1.91526
H	-4.02211	0.35624	3.11589
H	-2.58131	0.90405	2.25323
H	-4.93382	-0.70239	1.01060
Cl	-2.14940	1.96642	-0.73528
C	2.62509	0.24846	0.18875
C	3.59610	-0.64796	0.02309
C	5.00739	-0.22201	0.08866
O	5.39685	0.90262	0.26814
O	5.84488	-1.25850	-0.08711
H	6.74217	-0.90704	-0.03729
H	2.95439	1.27039	0.36859
H	3.40105	-1.69360	-0.17539

Sum of electronic and zero-point Energies= -1264.095279

Sum of electronic and thermal Energies= -1264.076972

Sum of electronic and thermal Enthalpies= -1264.076028

Sum of electronic and thermal Free Energies= -1264.143293

Dimethyl-enol ether (SOCL-DM)

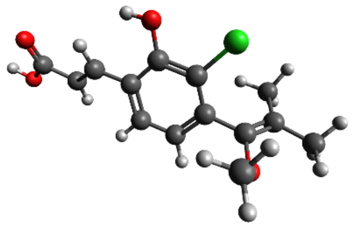

C	-0.61838	1.01800	0.14982
C	0.76610	0.81300	0.12919
C	1.30259	-0.46371	0.02701
C	0.42533	-1.54900	-0.02131
C	-0.93495	-1.35924	0.00968
C	-1.48688	-0.07906	0.09565
H	0.84071	-2.54635	-0.07304
H	-1.59664	-2.21563	-0.00296
O	-1.05070	2.29171	0.21913
H	-2.00214	2.33274	0.10507
C	2.76504	-0.74601	0.02675
O	3.19165	-1.48587	1.10380
C	2.95474	-0.89913	2.36909
H	3.35025	-1.59012	3.11097
H	3.46638	0.06436	2.45456
H	1.88590	-0.75099	2.55273
C	3.60609	-0.44921	-0.96220
C	3.16420	0.22609	-2.22454
H	3.62924	1.21195	-2.31163
H	3.48539	-0.35667	-3.09265
H	2.08521	0.35548	-2.28564
Cl	1.78720	2.20460	0.25251
C	-2.93117	0.12680	0.16436
C	-3.86804	-0.67784	-0.33504
C	-5.29294	-0.34558	-0.14805

O	-5.72227	0.62803	0.41499
O	-6.09383	-1.27452	-0.69818
H	-7.00261	-0.99007	-0.54229
H	-3.30359	1.00337	0.69163
H	-3.63527	-1.57639	-0.89102
C	5.06314	-0.79292	-0.89031
H	5.32632	-1.50846	-1.67464
H	5.66663	0.10272	-1.06327
H	5.33379	-1.22214	0.07083

Sum of electronic and zero-point Energies= -1303.387921
Sum of electronic and thermal Energies= -1303.368051
Sum of electronic and thermal Enthalpies= -1303.367107
Sum of electronic and thermal Free Energies= -1303.438180

Calculation of oxidation potential for SOCL-CB and SOCL-Ox probes

DFT calculations were calculated using uwb97xd/def2-TZVP level of theory.

<u>Oxytanyl-enol ether (SOCL-Ox)</u>

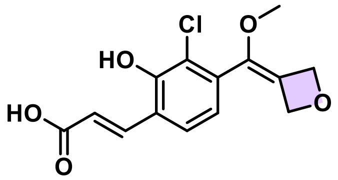

C	0.82697	-0.94406	0.40332
C	-0.54727	-0.68770	0.47729
C	-1.07246	0.52109	0.03848
C	-0.19542	1.49200	-0.44993
C	1.15387	1.24580	-0.52931
C	1.69235	0.02529	-0.11697
H	-0.60340	2.43524	-0.78744
H	1.81165	1.99237	-0.95485
O	1.25758	-2.13138	0.86964
H	2.21596	-2.15370	0.90575

C	-2.52103	0.84814	0.06829
O	-2.88053	1.96985	0.76714
C	-2.49821	1.97309	2.13153
H	-2.86726	2.90485	2.55449
H	-2.94681	1.12736	2.66080
H	-1.41082	1.93063	2.24544
C	-3.43224	0.21797	-0.64827
C	-3.46061	-0.90426	-1.64438
H	-3.25508	-1.90160	-1.24475
H	-2.87886	-0.74900	-2.55843
Cl	-1.55394	-1.90322	1.18541
C	3.12041	-0.25971	-0.24047
C	4.10553	0.63552	-0.20332
C	5.50314	0.19542	-0.38162
O	5.86866	-0.93986	-0.54228
O	6.35667	1.23240	-0.33785
H	7.24417	0.87263	-0.45651
H	3.42695	-1.29118	-0.40533
H	3.93341	1.69025	-0.03386
C	-4.90240	0.38362	-0.89127
H	-5.21022	1.33353	-1.33772
H	-5.55307	0.15331	-0.04188
O	-4.86805	-0.68642	-1.85388

Sum of electronic and zero-point Energies= -1377.372422

Sum of electronic and thermal Energies= -1377.352953

Sum of electronic and thermal Enthalpies= -1377.352009

Sum of electronic and thermal Free Energies= -1377.422633

Cyclobutyl-enol ether (SOCL-CB)

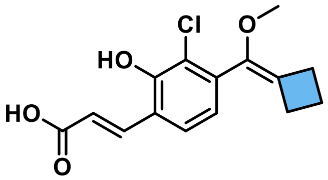

C	0.82718	-0.93113	0.40490
C	-0.54531	-0.66582	0.47170
C	-1.06504	0.53896	0.01572
C	-0.18023	1.49865	-0.48120
C	1.16828	1.24587	-0.55221
C	1.69966	0.02755	-0.12352
H	-0.58269	2.43979	-0.83110
H	1.83144	1.98439	-0.98365
O	1.25099	-2.11568	0.88674
H	2.20901	-2.13962	0.92861
C	-2.51263	0.87338	0.04571
O	-2.86402	1.97164	0.78932
C	-2.49567	1.91128	2.15430
H	-2.85084	2.83150	2.61362
H	-2.96330	1.05262	2.64556
H	-1.41004	1.84365	2.27758
C	-3.42434	0.27329	-0.70041
C	-3.38599	-0.86123	-1.69675
H	-2.91134	-0.59231	-2.64272
H	-2.94505	-1.79337	-1.34269
Cl	-1.56084	-1.86664	1.19379
C	3.12629	-0.26520	-0.23801
C	4.11628	0.62567	-0.21239
C	5.51147	0.17608	-0.37891
O	5.87324	-0.96282	-0.52350
O	6.37107	1.20920	-0.34434
H	7.25662	0.84199	-0.45407
H	3.42905	-1.30048	-0.38544

H	3.94895	1.68362	-0.05925
C	-4.91343	0.46423	-0.84852
H	-5.18445	1.38007	-1.37711
H	-5.48723	0.42339	0.07788
C	-4.93827	-0.80981	-1.73663
H	-5.38172	-0.68841	-2.72329
H	-5.40396	-1.66330	-1.24666

Sum of electronic and zero-point Energies= -1341.452028

Sum of electronic and thermal Energies= -1341.432294

Sum of electronic and thermal Enthalpies= -1341.431350

Sum of electronic and thermal Free Energies= -1341.502378

<u>Radical cation of oxytanyl-enol ether (SOCL-Ox)</u>

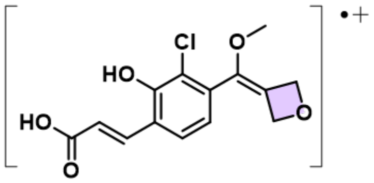

C	-0.80139	0.88070	0.51177
C	0.56296	0.60155	0.59869
C	1.07452	-0.57845	0.06388
C	0.22222	-1.49489	-0.55126
C	-1.12101	-1.20429	-0.65985
C	-1.65307	-0.02581	-0.14221
H	0.62011	-2.40178	-0.98737
H	-1.77372	-1.88678	-1.18726
O	-1.22785	2.01055	1.08000
H	-2.18762	2.06752	1.08685
C	2.51985	-0.83705	0.06985
O	3.05579	-1.84630	0.68129
C	2.30182	-2.69972	1.57078
H	3.02876	-3.07276	2.28501

H	1.51591	-2.13162	2.06386
H	1.88142	-3.52103	0.99454
C	3.40962	-0.04745	-0.65345
C	3.31131	1.09299	-1.59194
H	2.96068	2.03670	-1.15937
H	2.74192	0.88684	-2.50710
Cl	1.58907	1.69155	1.45441
C	-3.07605	0.30049	-0.27757
C	-4.07305	-0.57523	-0.22528
C	-5.47128	-0.09444	-0.39718
O	-5.77984	1.05521	-0.54660
O	-6.33958	-1.10737	-0.35932
H	-7.22824	-0.74550	-0.47280
H	-3.34976	1.33975	-0.44993
H	-3.92171	-1.63264	-0.04756
C	4.87056	-0.03878	-0.89089
H	5.28193	-0.93959	-1.36163
H	5.47792	0.20818	-0.01104
O	4.72599	1.05772	-1.79578

Sum of electronic and zero-point Energies=	-1377.094188
Sum of electronic and thermal Energies=	-1377.074701
Sum of electronic and thermal Enthalpies=	-1377.073757
Sum of electronic and thermal Free Energies=	-1377.144901

Radical cation of cyclobutyl-enol ether (SOCL-CB)

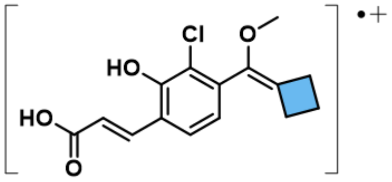

C	-0.80659	0.86709	0.52361
C	0.55665	0.58098	0.60538
C	1.06855	-0.57843	0.03255
C	0.21984	-1.46950	-0.61960
C	-1.12436	-1.17539	-0.71856
C	-1.65713	-0.01615	-0.16103
H	0.61834	-2.36254	-1.08310
H	-1.77706	-1.83938	-1.26926
O	-1.23263	1.98035	1.12633
H	-2.19221	2.03896	1.12841
C	2.51642	-0.84126	0.05611
O	3.03854	-1.81989	0.72635
C	2.25564	-2.62650	1.63320
H	2.97346	-3.03536	2.33669
H	1.51423	-2.01066	2.13799
H	1.77527	-3.42532	1.07218
C	3.42038	-0.08295	-0.69515
C	3.25301	1.04904	-1.64252
H	2.71240	0.76720	-2.55126
H	2.75572	1.92752	-1.22553
Cl	1.58968	1.64105	1.49210
C	-3.08111	0.31307	-0.28532
C	-4.07742	-0.56395	-0.24524
C	-5.47647	-0.08320	-0.40269
O	-5.78988	1.06777	-0.53253
O	-6.34371	-1.09826	-0.37577
H	-7.23291	-0.73469	-0.47831
H	-3.35522	1.35537	-0.43789
H	-3.92347	-1.62398	-0.08650
C	4.89913	-0.12745	-0.83268
H	5.26955	-1.04979	-1.28908
H	5.43806	0.00571	0.10882

```
C            4.79973         1.10402        -1.77270
H            5.18210         0.94639        -2.77700
H            5.24102         2.00652        -1.35887

 Sum of electronic and zero-point Energies=          -1341.186671
 Sum of electronic and thermal Energies=             -1341.166695
 Sum of electronic and thermal Enthalpies=           -1341.165750
 Sum of electronic and thermal Free Energies=        -1341.238436
```

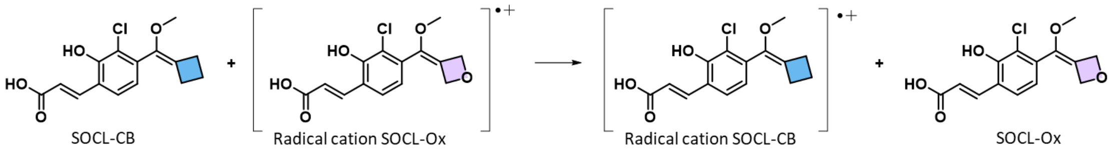

Probe	A	$B^{\cdot+}$	$A^{\cdot+}$	B
Free energies [Hartree]	-1341.502378	-1377.144901	-1341.238436	-1377.422633
Sum of free energies [Hartree]	-2718.647279		-2718.661069	
ΔG [Hartree]	-0.01379			

ΔG= -8.65335 [kcal/mol] ***Spontaneous reactions***

Table **S1.** (Top) Oxidation scheme of SOCL-CB compared to SOCL-Ox. The oxidation potential of probe SOCL-CB relative to probe SOCL-Ox is calculated based on the electron donation from SOCL-CB to the radical cation of SOCL-Ox. If the donation is spontaneous (ΔG < 0), it indicates that SOCL-CB has a higher HOMO energy, and thus a higher potential for electron donation. (Bottom) Summary table of the free energies and ΔG of the oxidation process. These free energies were obtained from DFT calculations using the **uwB97XD/def2-TZVP** level of theory. The calculations show that the reaction is spontaneous, supporting the conclusion that the SOCL-CB probe has a higher HOMO energy, indicating a stronger electron-donating character compared to SOCL-Ox.

Appendix II-Supplementary figures

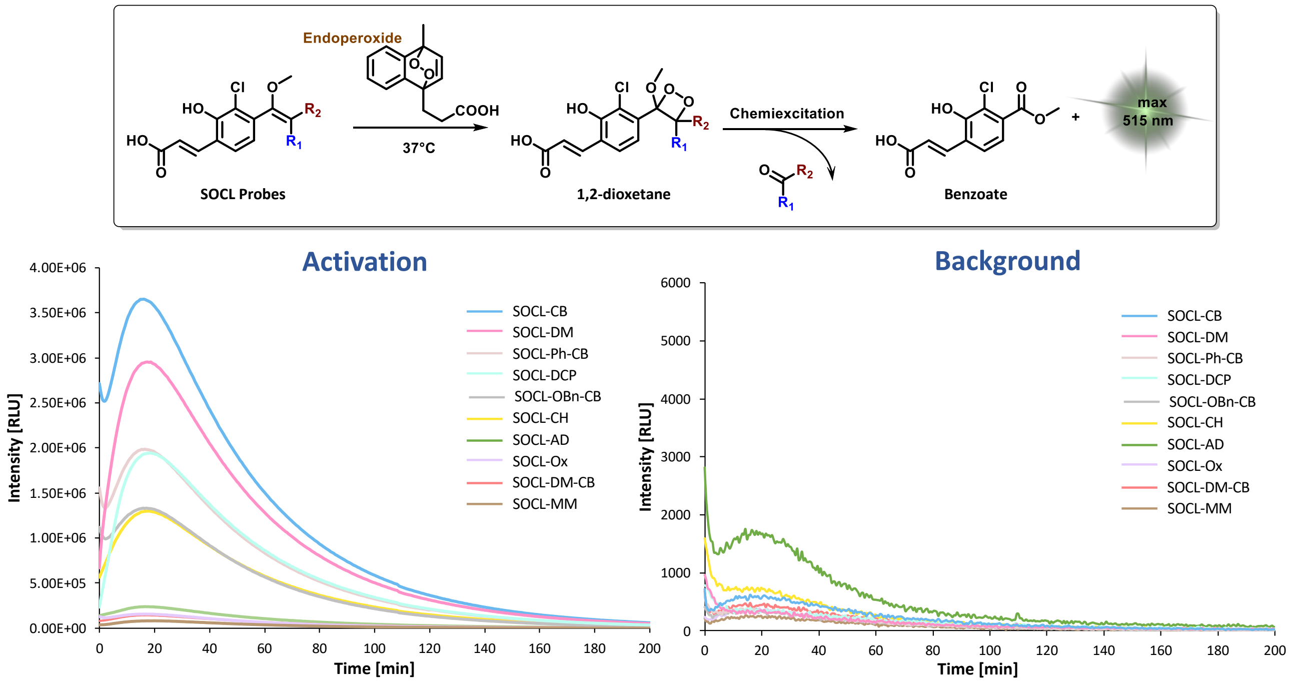

Figure **S1**. (Top) Activation scheme of the SOCL probes in the presence of endo-peroxide at 37 °C, and (bottom) chemiluminescent kinetic profiles of the ten SOCL probes [100 µM] with (left) and without (right) EP-1 [500 µM] in PBS (100 mM, pH 7.4), 10% DMSO, 37 °C. The detailed assay procedure is mentioned in the experimental protocols section.

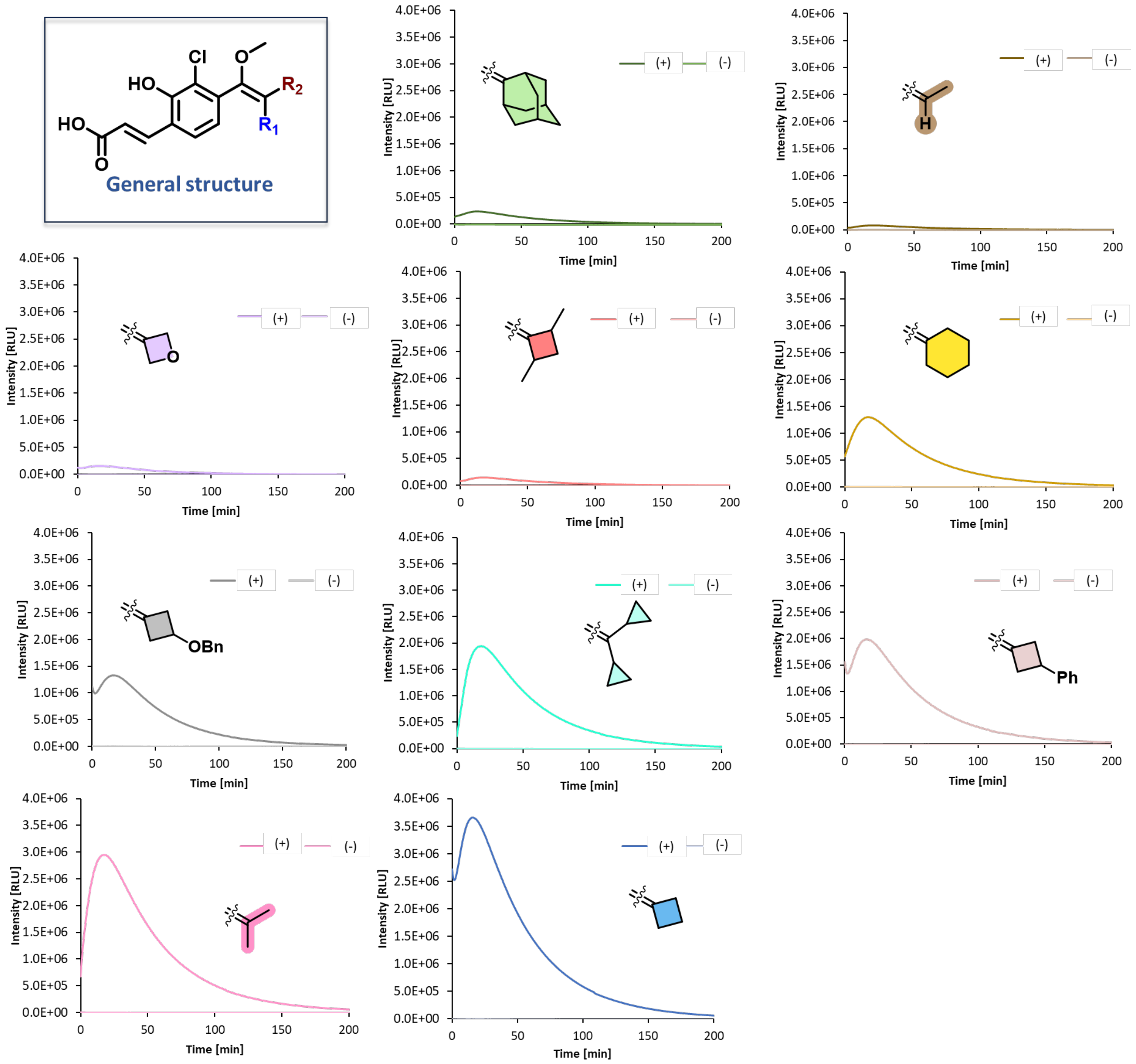

Figure **S2**. Chemiluminescent kinetic profiles of the ten SOCL probes [100 µM] with and without EP-1 [500 µM] in PBS (100 mM, pH 7.4), 10% DMSO, 37 °C.

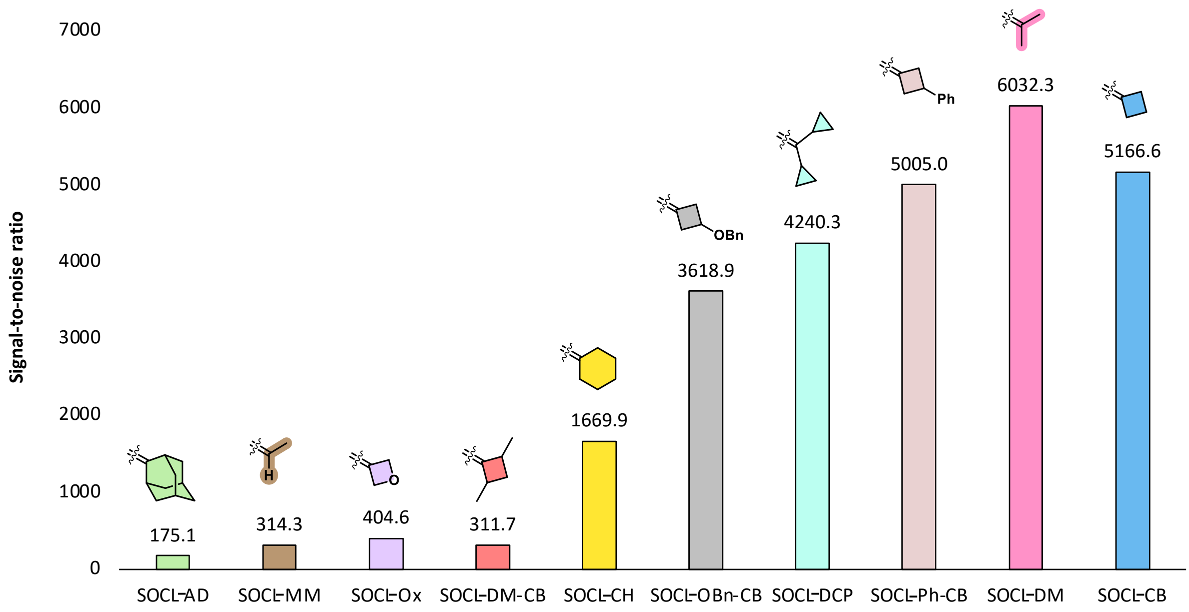

Figure **S3**. Signal-to-noise ratio (of the total light emitted in full profile) of the ten SOCL probes [100 µM] with and without EP-1 [500 µM] in PBS (100 mM, pH 7.4), 10% DMSO, 37 °C .

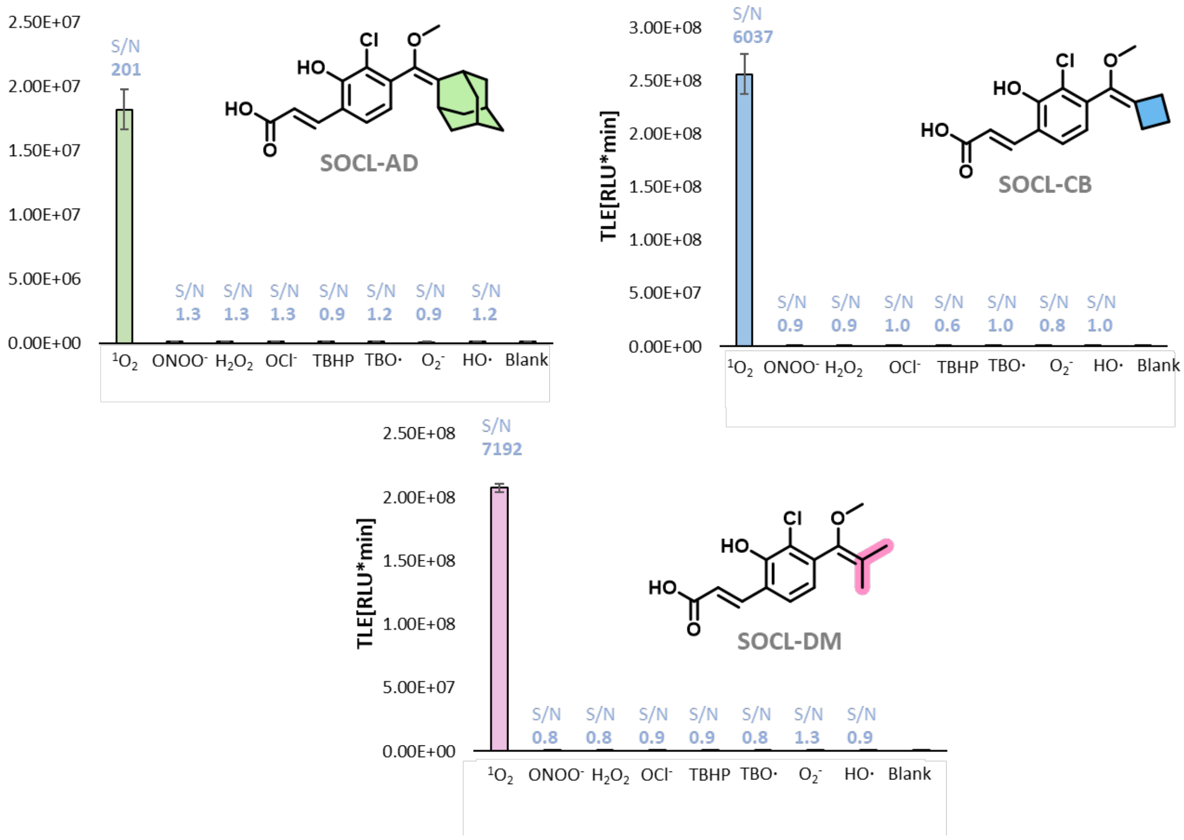

Figure **S4**. Selectivity assay of the three chemiluminescent SOCL probes- **SOCL-AD** (top-left), **SOCL-CB** (top-right), and **SOCL-DM** (bottom)- evaluated in the presence of eight analytes, including ROS. The chemiluminescent intensity of the SOCL probes [100 µM] was measured in the presence and absence

of each of the analytes: $ONOO^-$, $^1O_2$, H_2O_2, ClO^-, TBHP, TBO·, OH·and O_2^-, each at a concentration of [500 µM], PBS (100 mM, pH 7.4), 1% DMSO, 37°C. The detailed assay procedure is mentioned in the experimental protocols section.

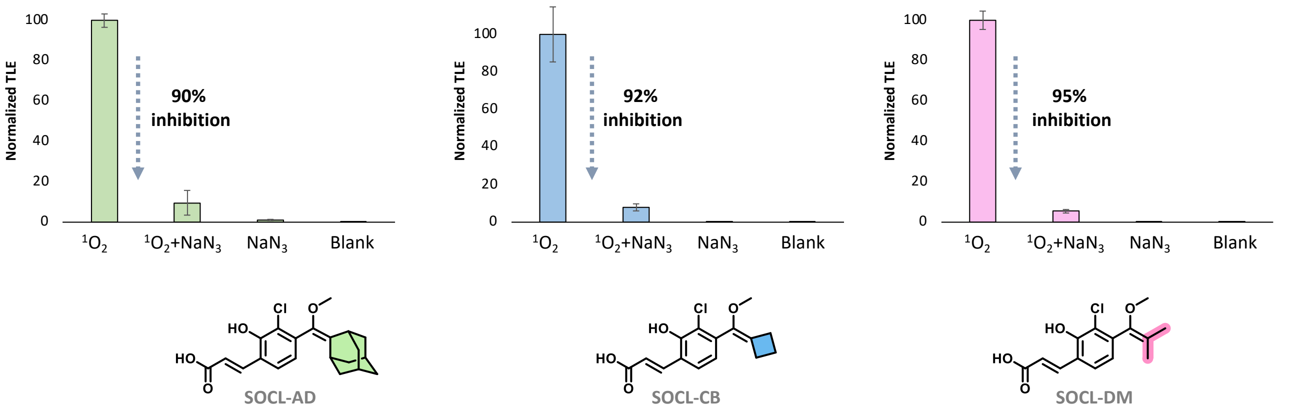

Figure **S5.** Chemiluminescence attenuation by NaN3, a known $^1O_2$ scavenger. (left) Normalized total light emitted over 160 minutes by **SOCL-AD** [100 µM] after incubation in the presence and absence of EP-1 [100 µM] in PBS (100 mM, pH 7.4) at 37°C, with and without NaN3 [10 mM]. (Middle) Normalized total light emitted over 160 minutes by **SOCL-CB** [100 µM] under the same conditions. (right) Normalized total light emitted over 160 minutes by **SOCL-DM** [100 µM] under the same conditions.

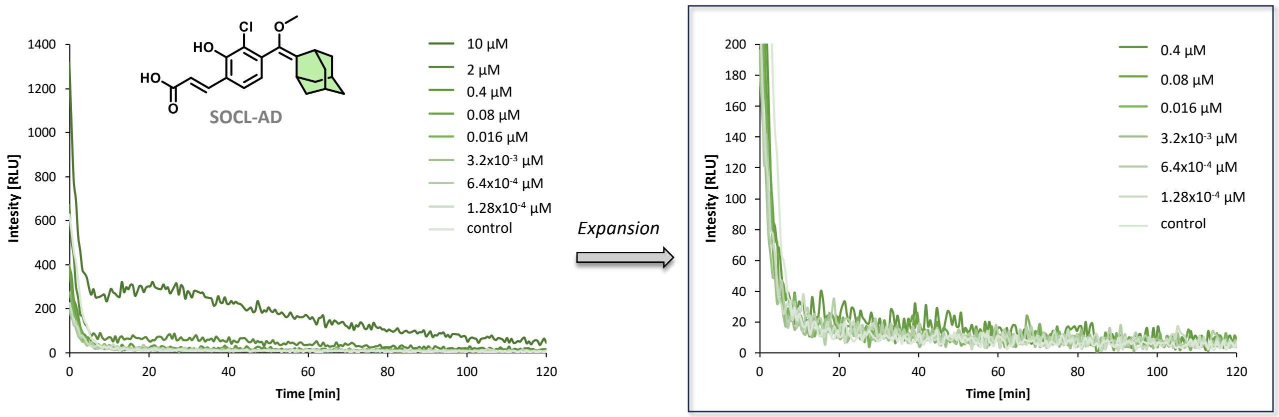

Figure **S6.** Chemiluminescent kinetic profiles during 2 hours of **SOCL-AD** probe [10 µM] with various concentrations of EP-1 [10 – $1.28 \cdot 10^{-4}$ µM] in PBS (100 mM, pH 7.4), 10% DMSO, 37°C. The detailed assay procedure is mentioned in the experimental protocols section.

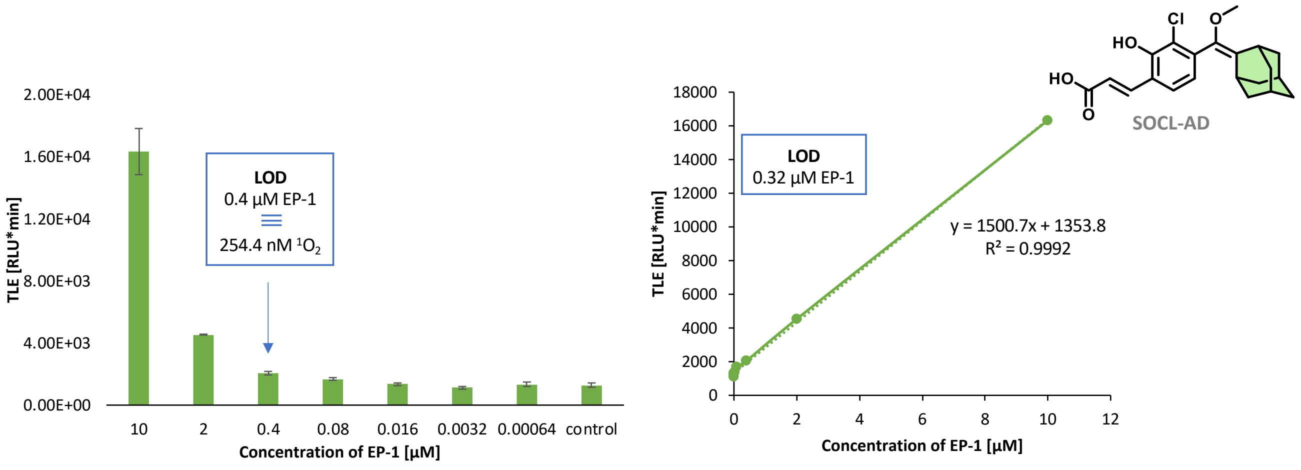

Figure **S7.** Total light emitted (left) and linear calibration curve (right) after 60 minutes of **SOCL-AD** probe [10 µM] with various concentrations of EP-1 [10 – 1.28·10^{-4} µM] in PBS (100 mM, pH 7.4), 10% DMSO, 37°C. The limit of detection (LOD) was determined using two methods: (1) the blank + 3SD (standard deviation) method (left), and (2) the linear calibration curve (right). For the latter, the limit of detection is defined as 3 times the standard deviation of the blank divided by the slope of the linear calibration curve (LOD = 3σ/k). The results of the LOD comparison presented in the manuscript are based on the first method. To evaluate the LOD in terms of $^1O_2$ concentrations, the LOD value obtained after 60 minutes was multiplied by 636 to convert the concentration into [nM] units. Details on the conversion method and the assay are described in the experimental protocols section.

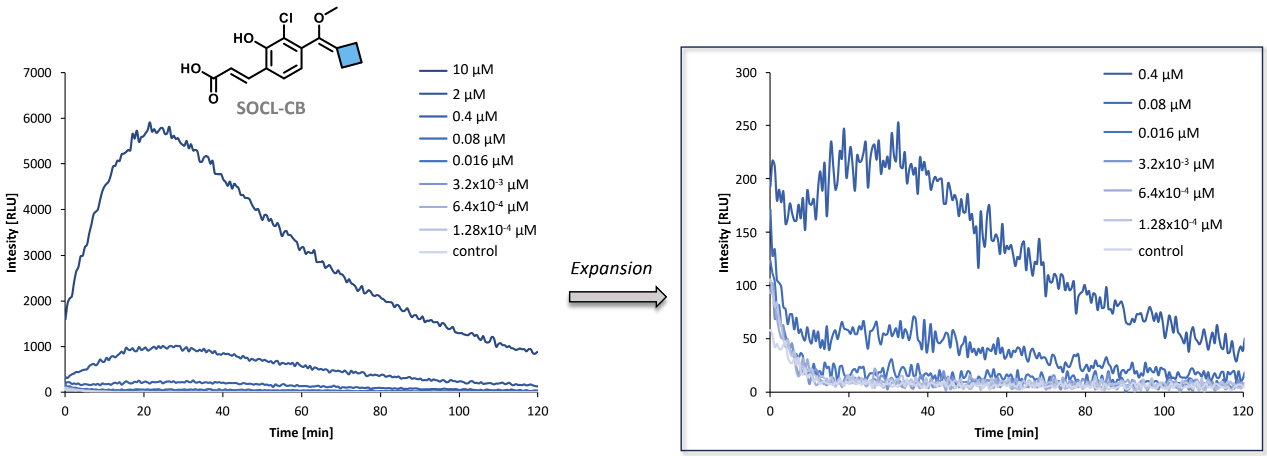

Figure **S8.** Chemiluminescent kinetic profiles during 2 hours of **SOCL-CB** probe [10 µM] with various concentrations of EP-1 [10 – 1.28·10^{-4} µM] in PBS (100 mM, pH 7.4), 10% DMSO, 37°C. The detailed assay procedure is mentioned in the experimental protocols section.

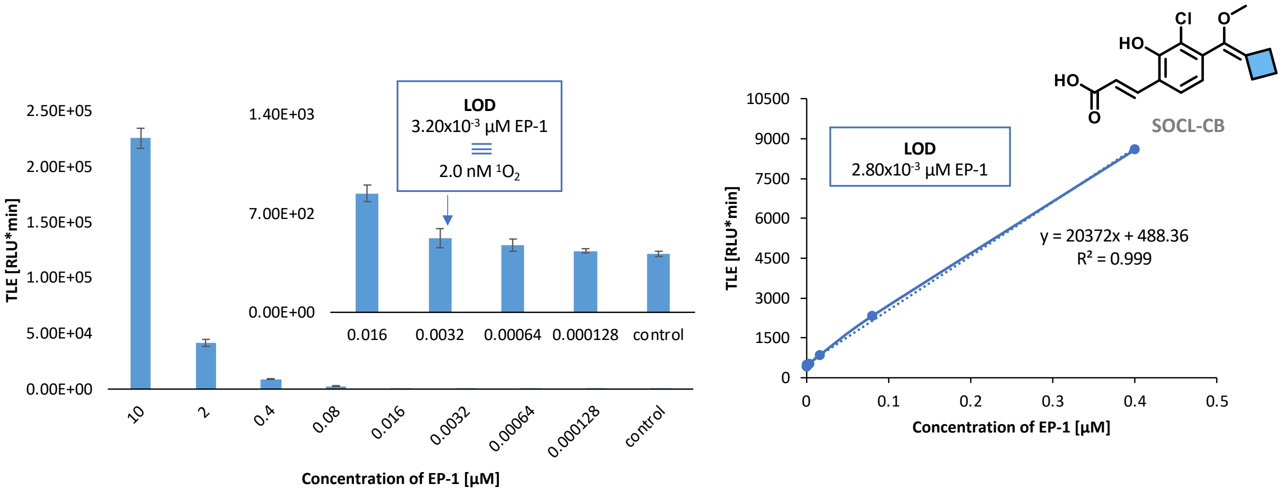

Figure **S9.** Total light emitted (left) and linear calibration curve (right) after <u>60 minutes</u> of **SOCL-CB** probe [10 µM] with various concentrations of EP-1 [10 – 1.28·10^{-4} µM] in PBS (100 mM, pH 7.4), 10% DMSO, 37°C. The LOD was calculated using the same approach as described in Figure **S7**.

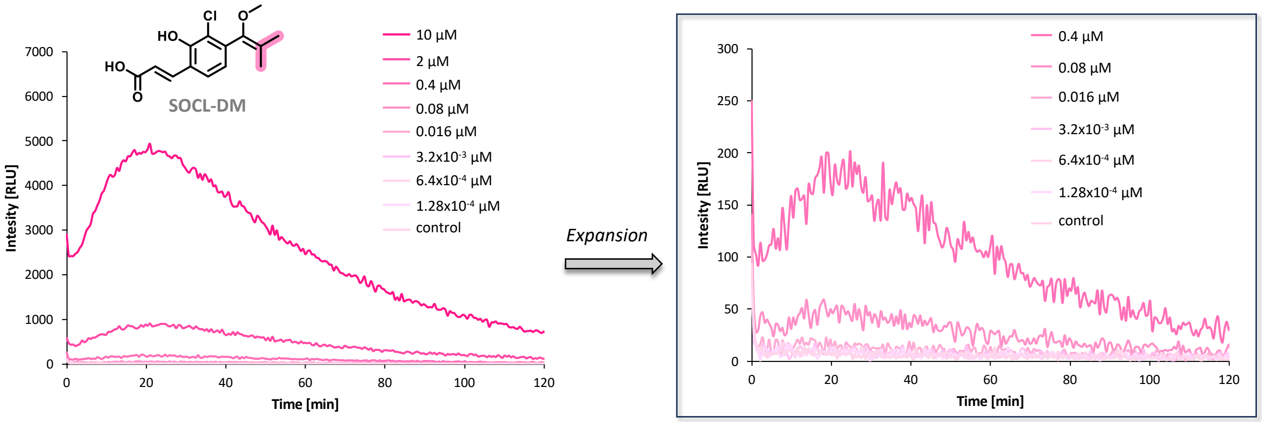

Figure **S10.** Chemiluminescent kinetic profiles during 2 hours of **SOCL-DM** probe [10 µM] with various concentrations of EP-1 [2 – 1.28·10^{-4} µM] in PBS (100 mM, pH 7.4), 10% DMSO, 37°C. The detailed assay procedure is mentioned in the experimental protocols section.

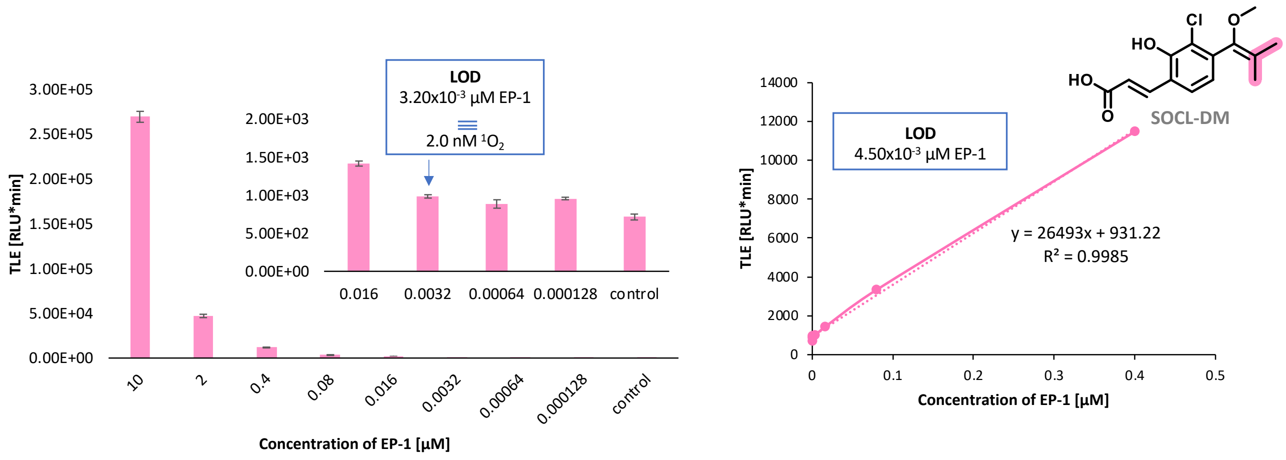

Figure **S11.** Total light emitted (left) and linear calibration curve (right) after <u>60 minutes</u> of **SOCL-DM** probe [10 μM] with various concentrations of EP-1 [10 – 1.28·10^{-4} μM] in PBS (100 mM, pH 7.4), 10% DMSO, 37°C. The LOD was calculated using the same approach as described in Figure **S7**.

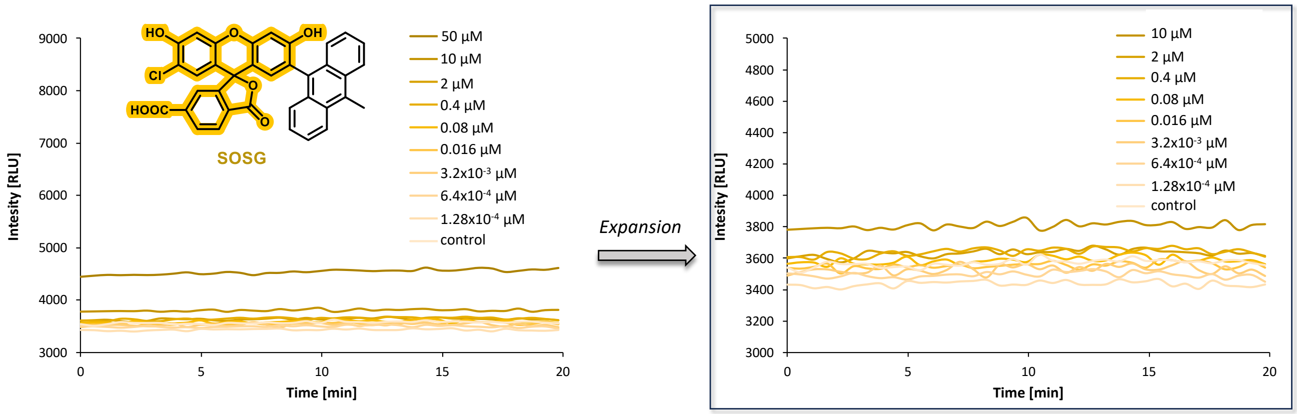

Figure **S12.** Fluorescent kinetic profiles were measured at λ_{em}=530nm after 2 hours of incubation of the commercial $^1O_2$ fluorescent probe (**SOSG**) [10 μM] with various concentrations of EP-1 [10–1.28·10^{-4} μM] in PBS (100 mM, pH 7.4), 10% DMSO, 37°C. λ_{ex}=500nm. The detailed assay procedure is mentioned in the experimental protocols section.

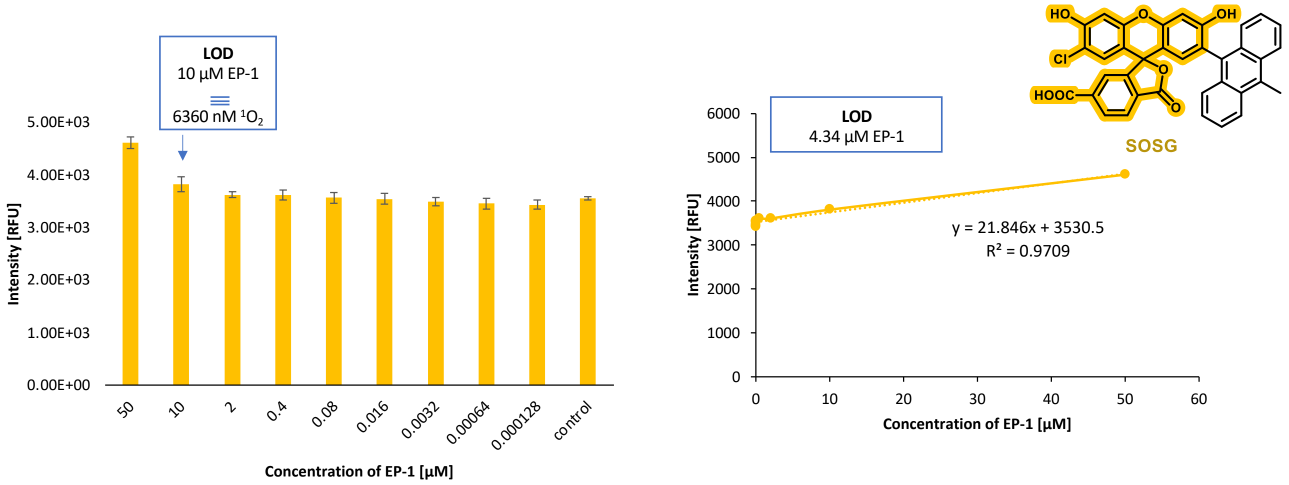

Figure **S13.** Light intensity (left) and linear calibration curve (right) after <u>60 minutes</u> of **SOSG** probe [10 µM] with various concentrations of EP-1 [10 – 1.28·10^{-4} µM] in PBS (100 mM, pH 7.4), 10% DMSO, 37°C. The LOD was calculated using the same approach as described in Figure **S7**. The signal was measured at λ_{em}=530 nm while exciting at λ_{ex}=500 nm.

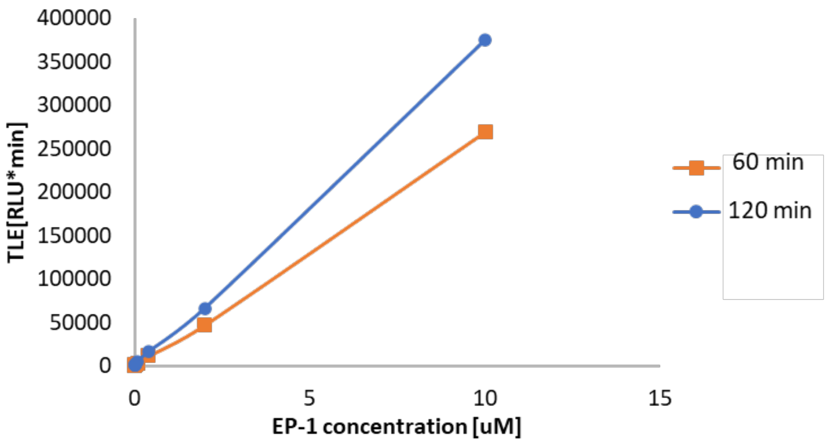

Figure **S14.** Total light emitted during <u>60 minutes</u> (orange) or <u>120 minutes</u> (blue) from probe SOCL-DM [10 µM] upon incubation with various concentrations of EP-1 [10 – 1.28·10^{-4} µM] in PBS (100 mM, pH 7.4), 10% DMSO, 37°C. The increased light emission at 120 minutes reflects higher overall singlet oxygen ($^1O_2$) production as EP-1 decomposition progresses according to first-order kinetics. The detailed assay procedure is mentioned in the experimental protocols section.

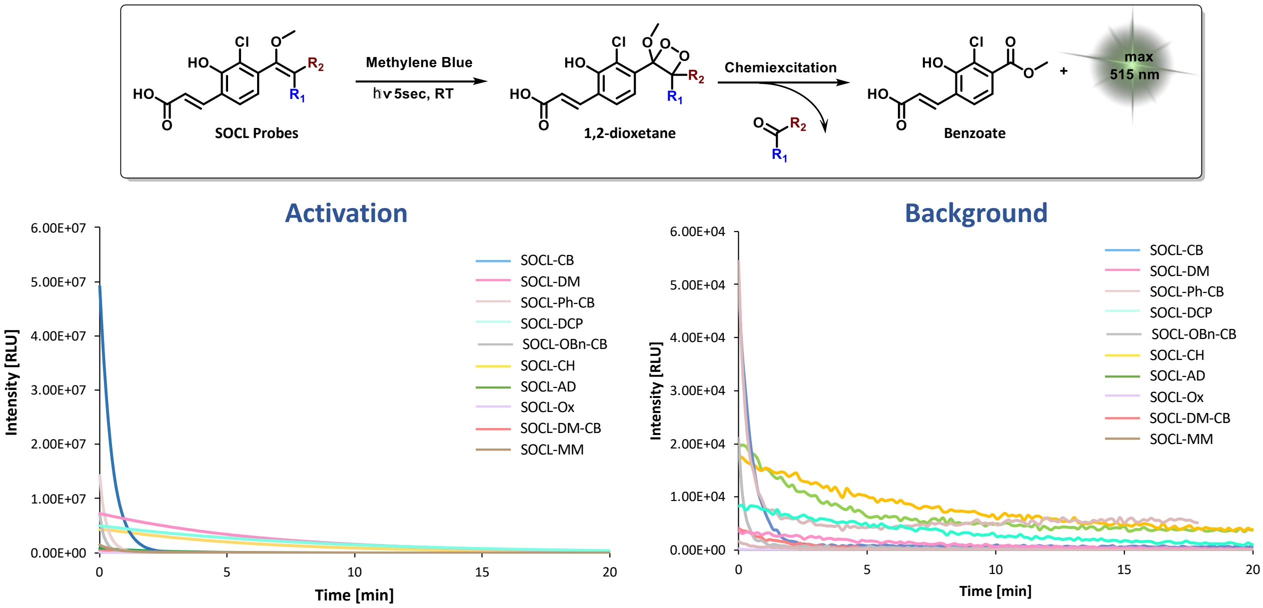

Figure **S15**. Chemiluminescent kinetic profiles of the ten SOCL probes [100 µM] with (left) and without (right) MB [10 µM] in PBS (100 mM, pH 7.4) with 1% DMSO measured at room temperature after irradiation for 5 sec. The detailed assay procedure is mentioned in the experimental protocols section.

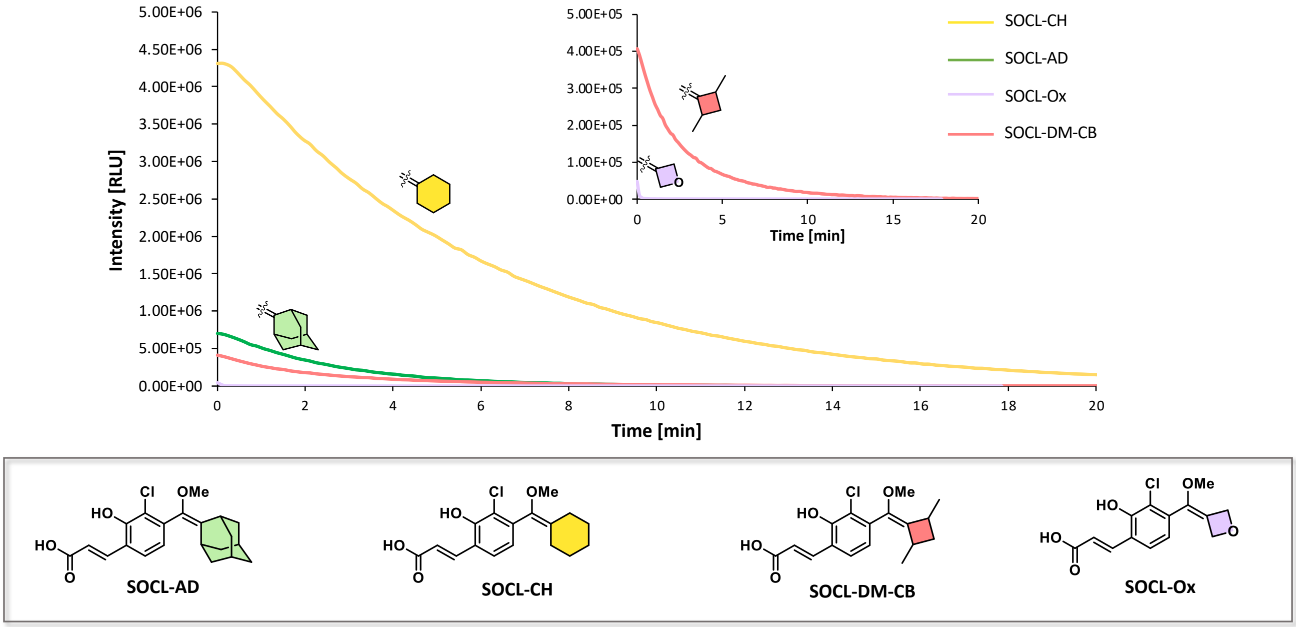

Figure **S16**. Chemiluminescent kinetic profiles of four SOCL probes (**SOCL-AD**, **SOCL-CH**, **SOCL-DM-CB**, and **SOCL-Ox**) [100 µM] with MB [10 µM] in PBS (100 mM, pH 7.4) with 1% DMSO after irradiation for 5 sec.

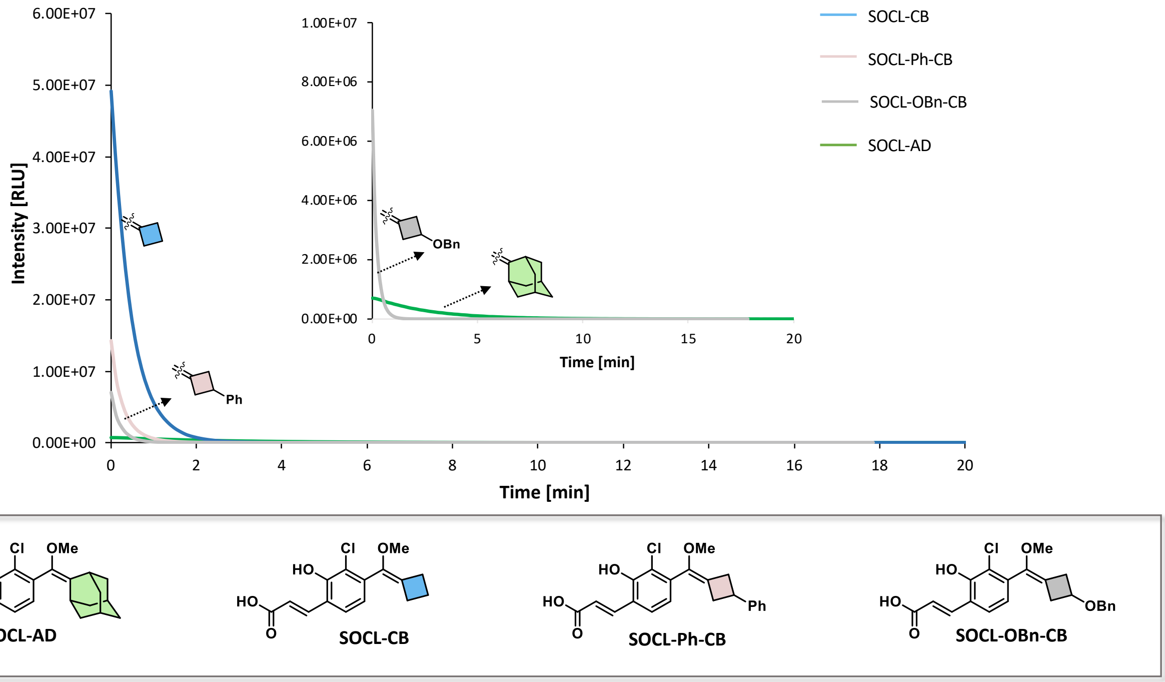

Figure **S17**. Chemiluminescent kinetic profiles of four SOCL probes (**SOCL-AD**, **SOCL-CB**, **SOCL-Ph-CB**, and **SOCL-OBn-CB**) [100 µM] with MB [10 µM] in PBS (100 mM, pH 7.4) with 1% DMSO after irradiation for 5 sec.

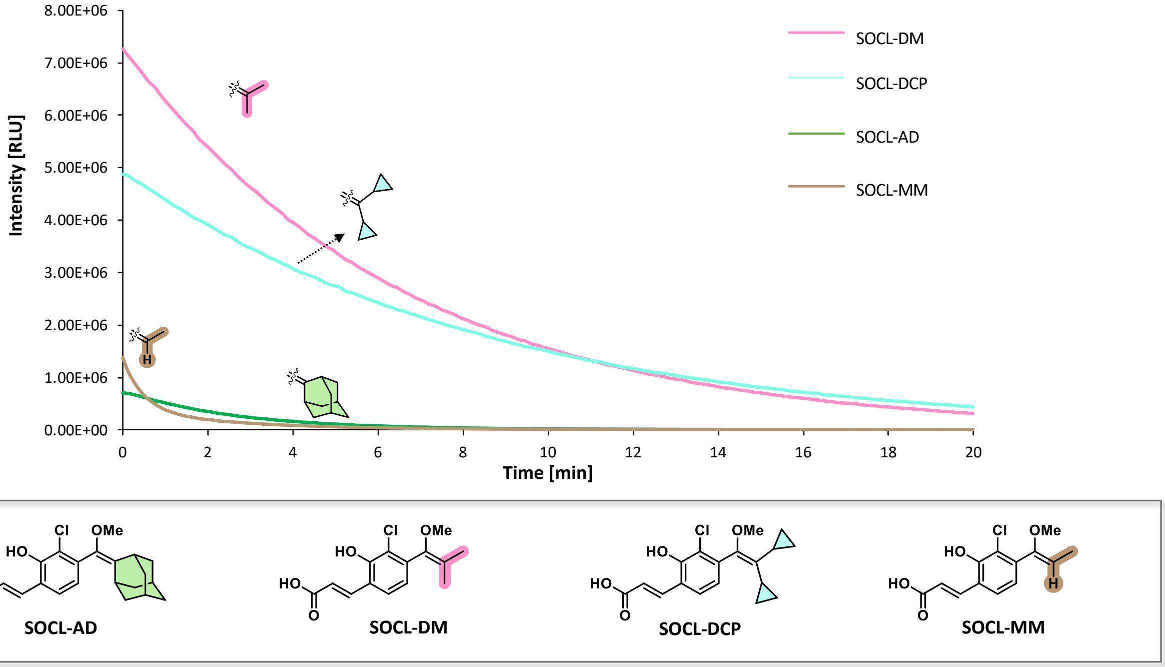

Figure **S18**. Chemiluminescent kinetic profiles of four SOCL probes (**SOCL-AD**, **SOCL-DM**, **SOCL-DCP**, and **SOCL-MM**) [100 µM] with MB [10 µM] in PBS (100 mM, pH 7.4) with 1% DMSO after irradiation for 5 sec.

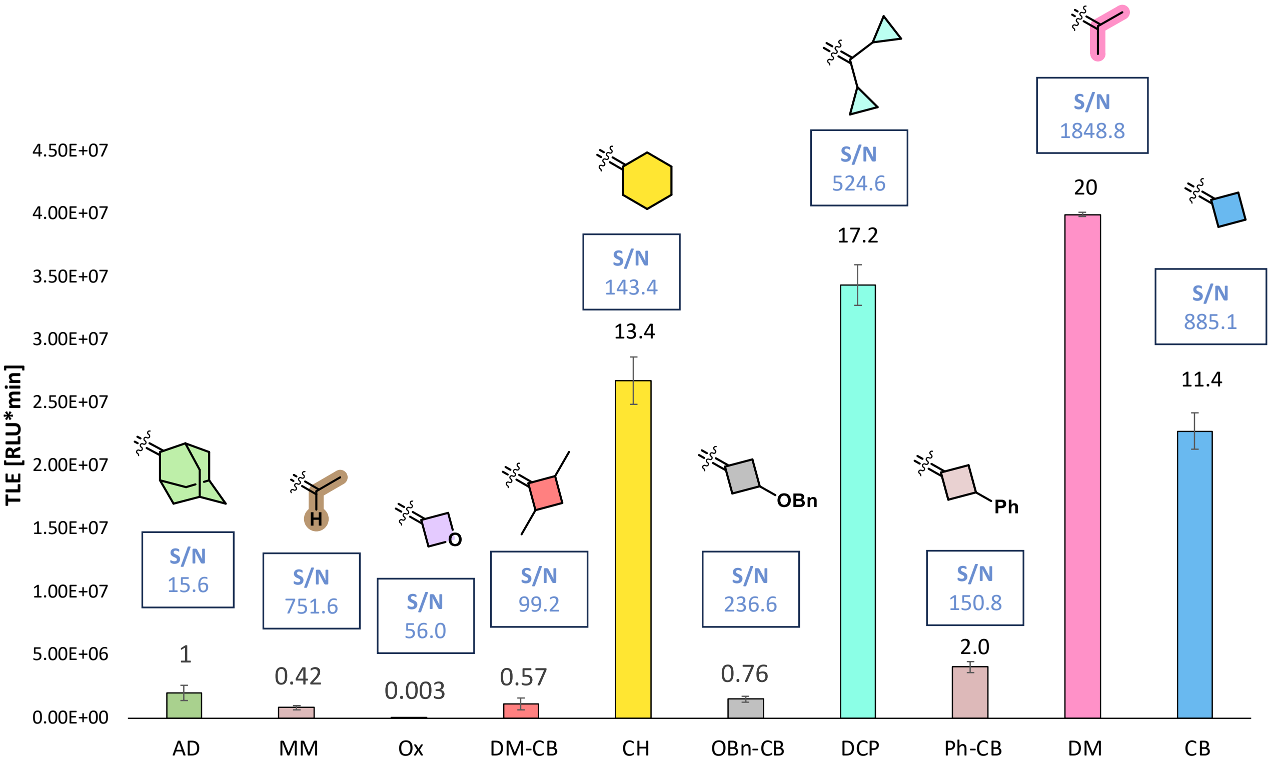

Figure **S19**. Total light emitted by ten SOCL probes [100 µM] after complete decay, and signal-to-noise ratio measured with and without MB [10 µM] in PBS (100 mM, pH 7.4) with 1% DMSO after irradiation for 5 sec at room temperature. **SOCL-AD** probe is considered as the reference

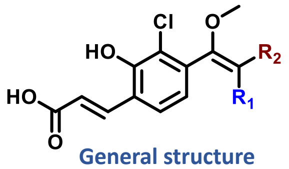

General structure

Probe	SOCL-AD	SOCL- MM	SOCL- OX	SOCL-DM-CB	SOCL-CH	SOCL-OBn-CB	SOCL-DCP	SOCL-Ph-CB	SOCL-DM	SOCL-CB
**Structure**	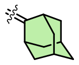	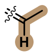	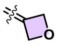	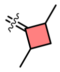	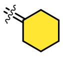	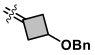	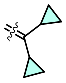	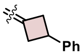	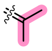	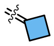
**TLE [RLU*min]**	$2.00 \cdot 10^6$	$8.43 \cdot 10^5$	$5.79 \cdot 10^3$	$1.14 \cdot 10^6$	[illegible]$.68 \cdot 10^7$	$1.51 \cdot 10^6$	$3.44 \cdot 10^7$	$4.07 \cdot 10^6$	$4.00 \cdot 10^7$	$2.28 \cdot 10^7$
**Rel. TLE**	1	0.4	0.003	0.6	13.4	0.7	17.2	2.0	20.0	11.4
**S/N**	15.6	751.6	56.0	99.2	143.4	236.3	524.6	150.8	1848.9	885.1

Figure **S20**. A table summarizes the chemiluminescent properties: total light emitted, relative total light emitted with probe **SOCL-AD** as the reference, and signal-to-noise ratio of ten SOCL probes [100 µM] with and without MB [10 µM] in PBS (100 mM, pH 7.4) with 1% DMSO after irradiation for 5 sec at room temperature.

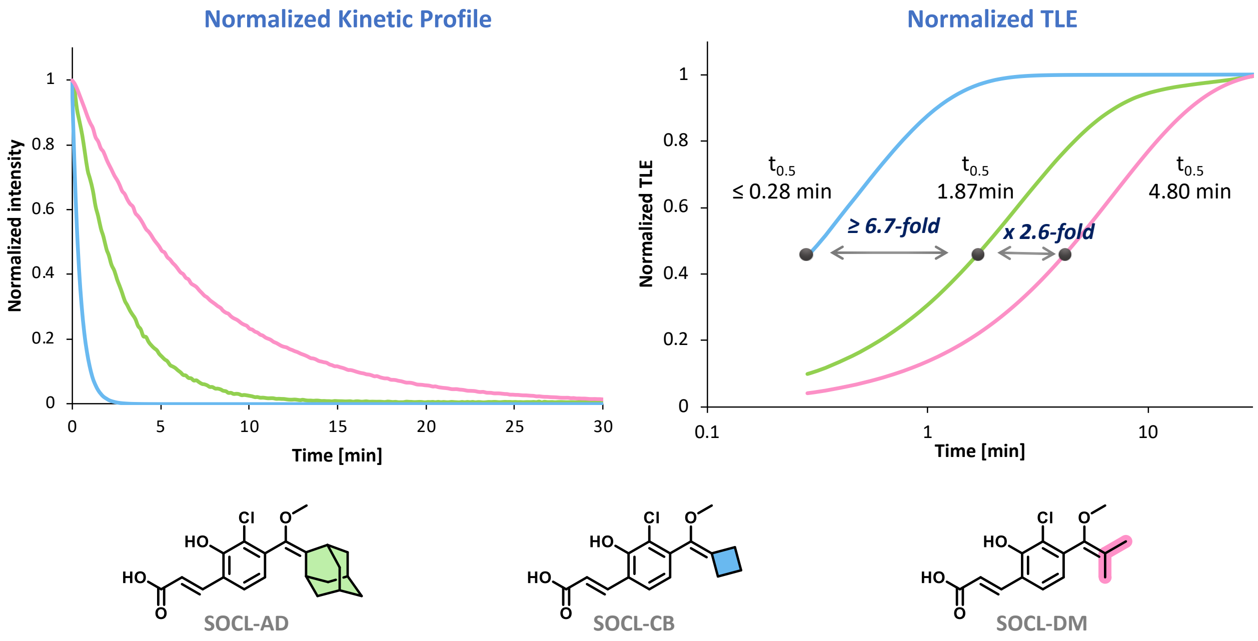

Figure **S21**. Normalized kinetic profiles (left) and normalized total light emission kinetic profile (right) (time represented in logarithmic scale) of **SOCL-AD**, **SOCL-CB,** and **SOCL-DM** probes [100 µM] with and without MB [10 µM] in PBS (100 mM, pH 7.4) with 1% DMSO after irradiation for 5 sec at room temperature.

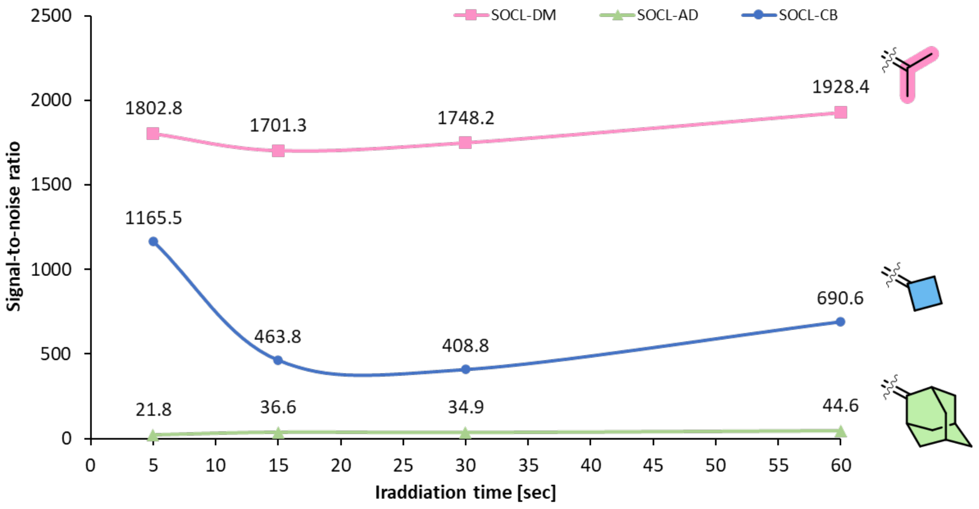

Figure **S22**. Signal-to-noise ratio (of the total light emitted in full profile), as a function of different irradiation durations: 5, 15 30, and 60 seconds, obtained from **SOCL-AD**, **SOCL-CB**, and **SOCL-DM** probes [100 µM] with and without MB [10 µM] in PBS (100 mM, pH 7.4) with 1% DMSO at room temperature.

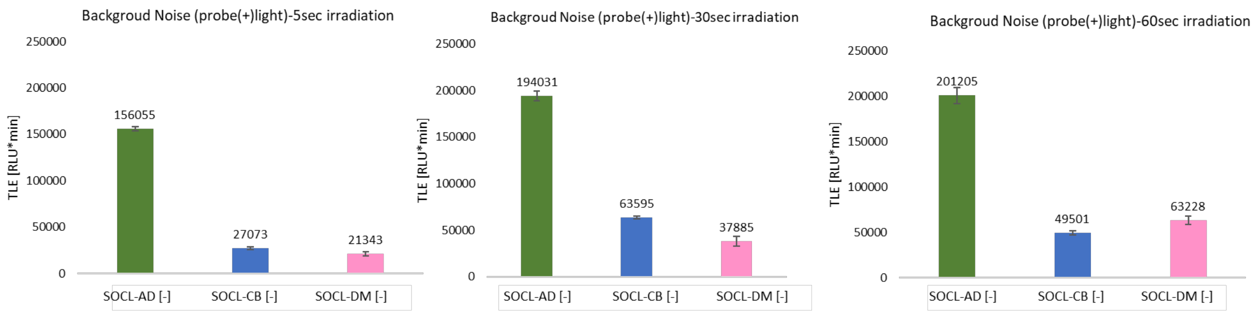

Figure **S23**. Self-photooxidation of SOCL probes.[17] Total light emitted by **SOCL-AD** (green), **SOCL-CB** (blue), and **SOCL-DM** (pink) probes [100 µM] after irradiation for 5 seconds (left), 30 seconds (middle), and 60 seconds (right) in PBS (100 mM, pH 7.4), 1% DMSO at room temperature.

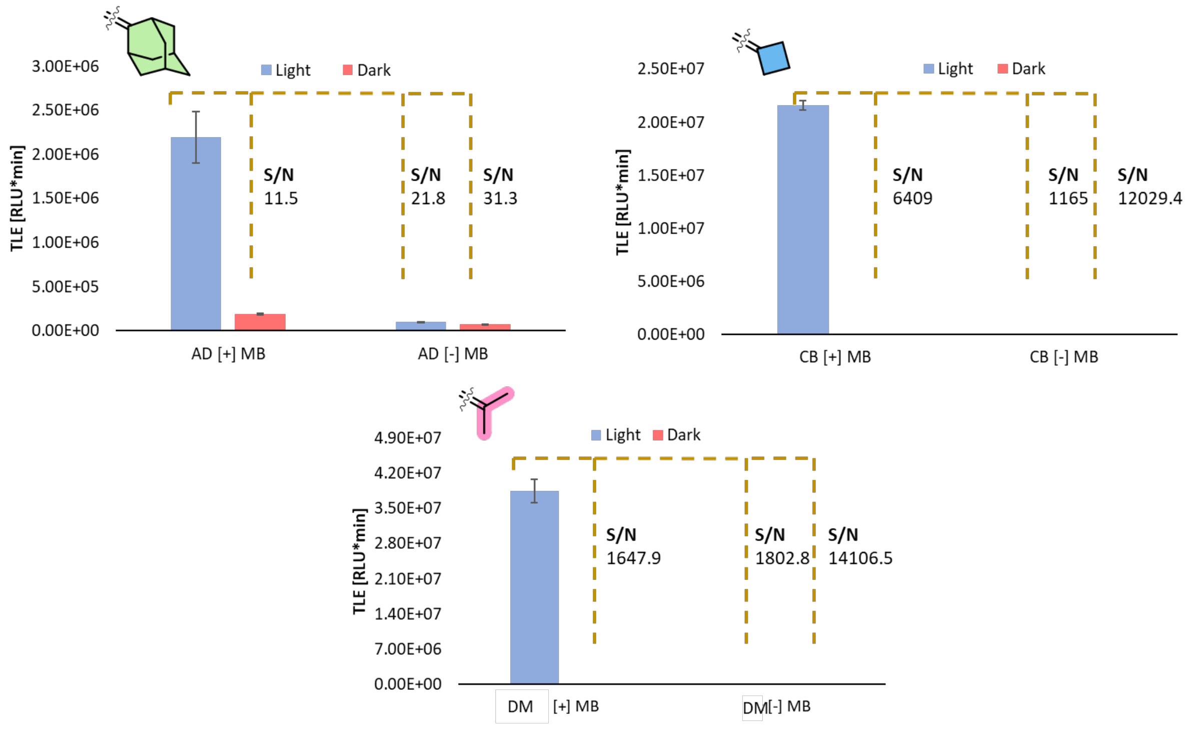

Figure **S24**. Total light emitted by **SOCL-AD** (left), **SOCL-CB** (right), and **SOCL-DM** (bottom) probes [100 µM] after incubation with or without MB [10 µM] in PBS (100 mM, pH 7.4) with 1% DMSO. The chemiluminescent kinetic profiles were then measured in the presence and absence of 5 sec of light irradiation.

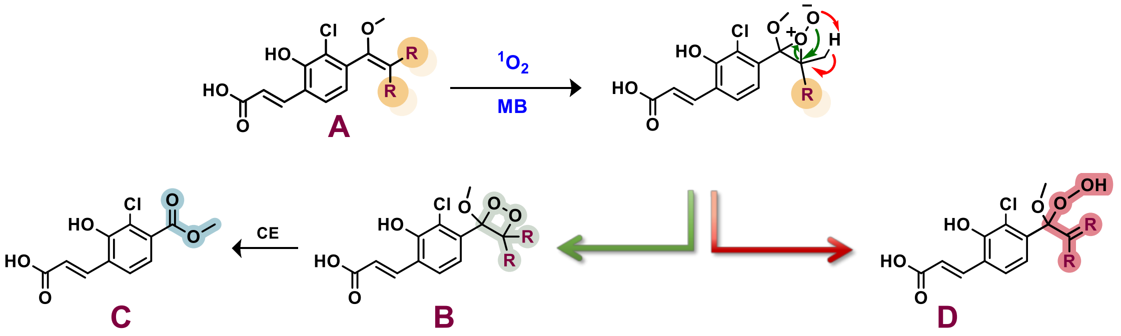

HPLC comparison between oxidation in **organic** and **aqueous** media

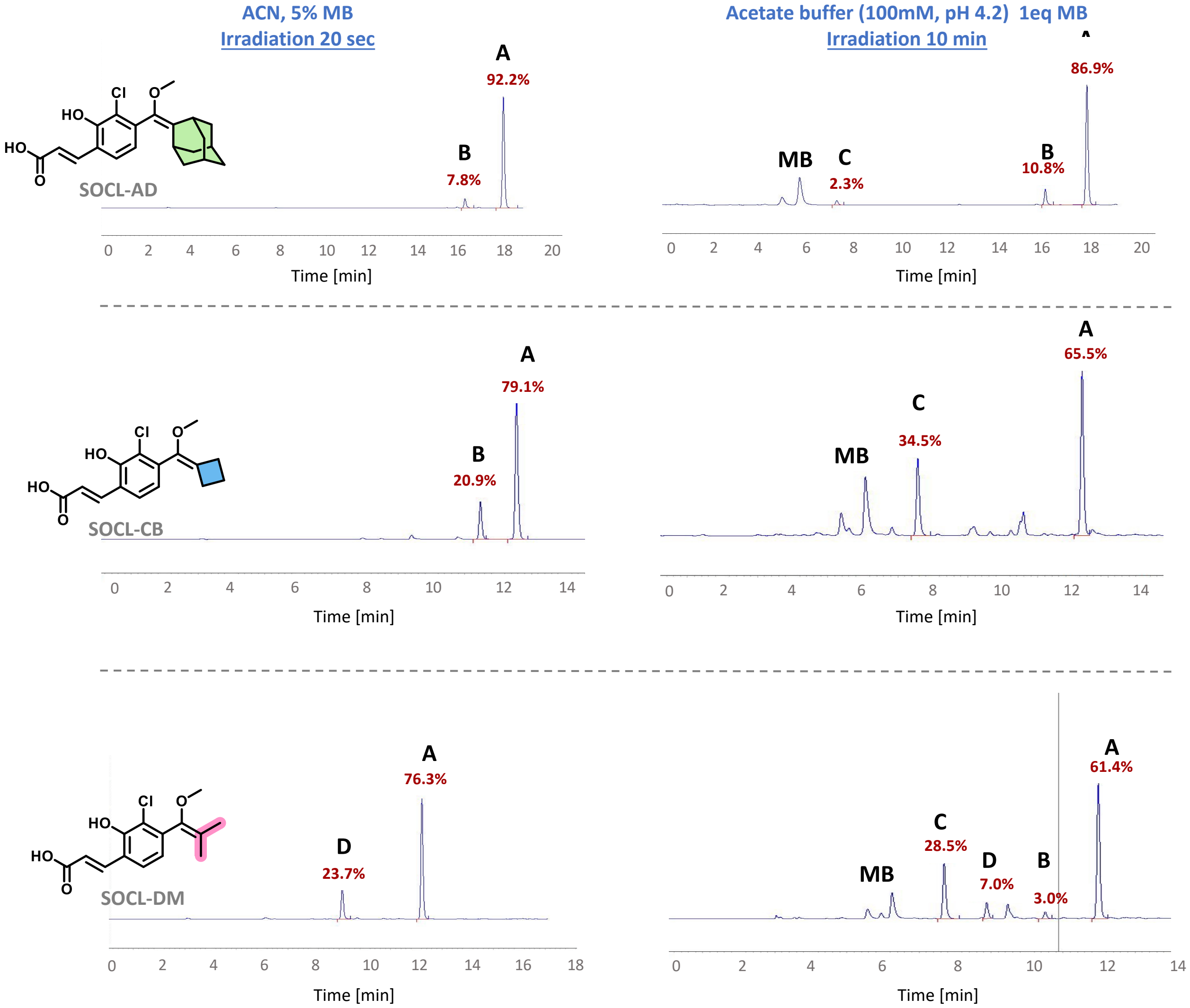

Figure **S25**. (Top) Oxidation pathways of SOCL probes (A) by singlet oxygen generated in the presence of methylene blue: formation of the 'Ene'-product (D) versus the 1,2-dioxetane product (B), which decomposes to benzoate (C). (Bottom) Oxidation of **SOCL-AD**, **SOCL-CB**, and **SOCL-DM** probes [300 µM] after incubation with MB [15 or 300 µM] in ACN or acetate buffer (pH 4.2), 3% DMSO, following 20 seconds or 10 minutes of irradiation, respectively. The product distribution ratio was determined by RP-HPLC (30–100% ACN in water with 0.1% TFA). The detailed assay procedure is mentioned in the experimental protocols section.

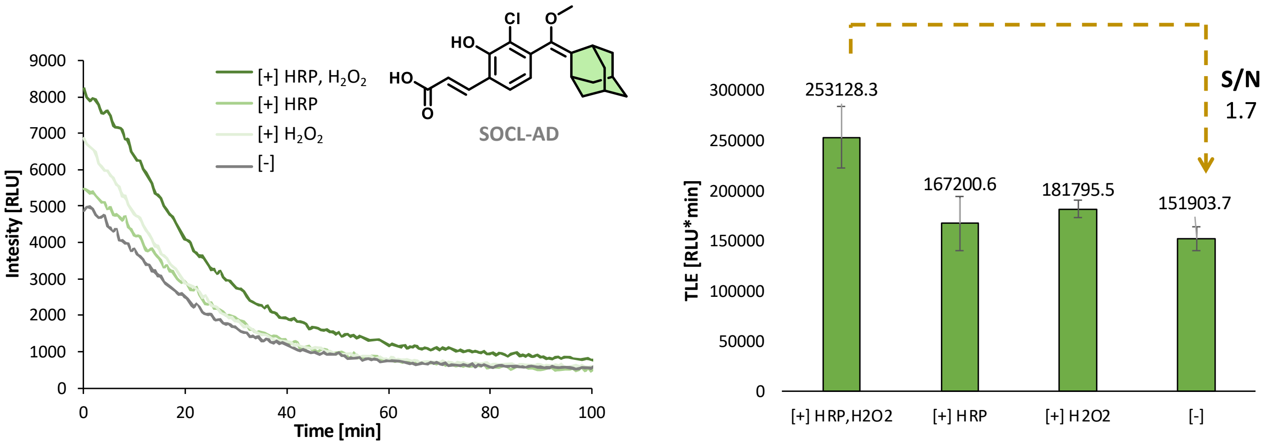

Figure **S26**. Chemiluminescence kinetic profiles (left) and Total light emitted (right) during 100 minutes by **SOCL-AD** probe [100 µM] after incubation with and without Horseradish peroxidase [10 µg/mL], and in the presence and absence of H_2O_2 [100 µM] in PB (50 mM, pH 6.0), 1% DMSO at 30°C. The detailed assay procedure is mentioned in the experimental protocols section.

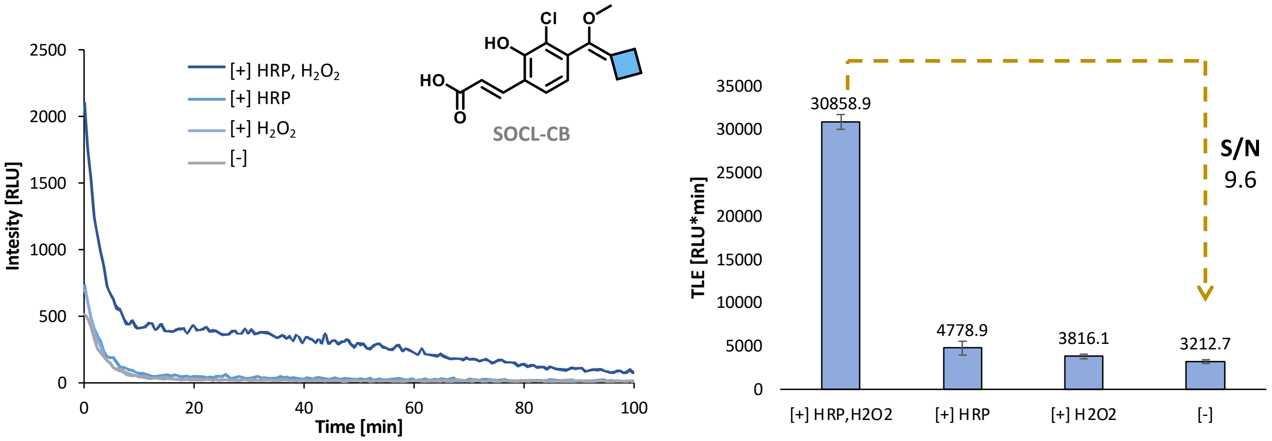

Figure **S27**. Chemiluminescence kinetic profiles (left) and Total light emitted (right) during 100 minutes by **SOCL-CB** probe [100 µM] after incubation with and without Horseradish peroxidase [10 µg/mL], and in the presence and absence of H_2O_2 [100 µM] in PB (50 mM, pH 6.0), 1% DMSO at 30°C. The detailed assay procedure is mentioned in the experimental protocols section.

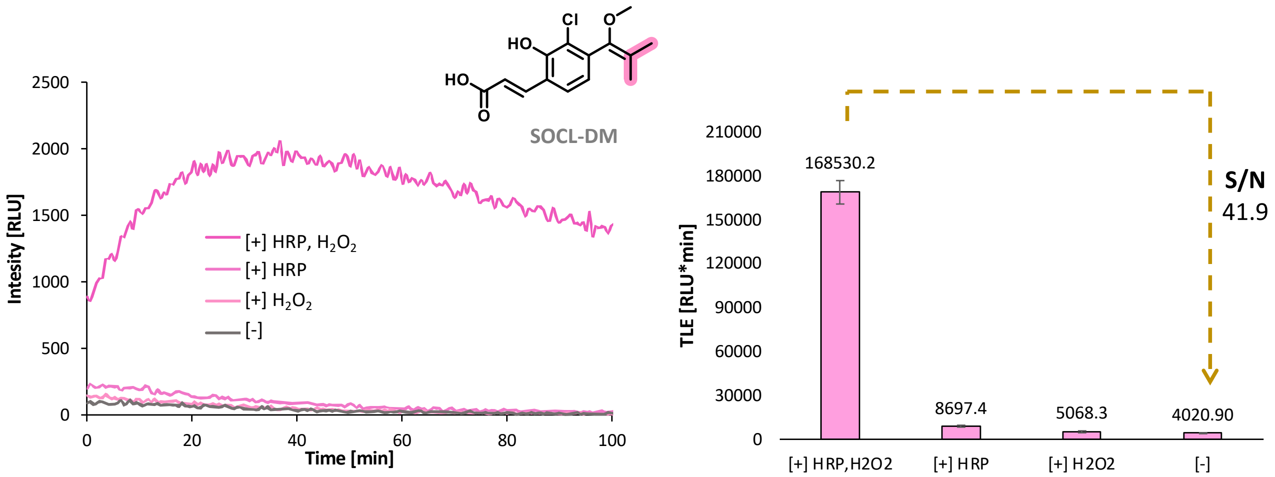

Figure **S28**. Chemiluminescence kinetic profiles (left) and Total light emitted (right) during 100 minutes by **SOCL-DM** probe [100 µM] after incubation with and without Horseradish peroxidase (HRP) [10 µg/mL], and in the presence and absence of H_2O_2 [100 µM] in PB (50 mM, pH 6.0), 1% DMSO at 30°C. The detailed assay procedure is mentioned in the experimental protocols section.

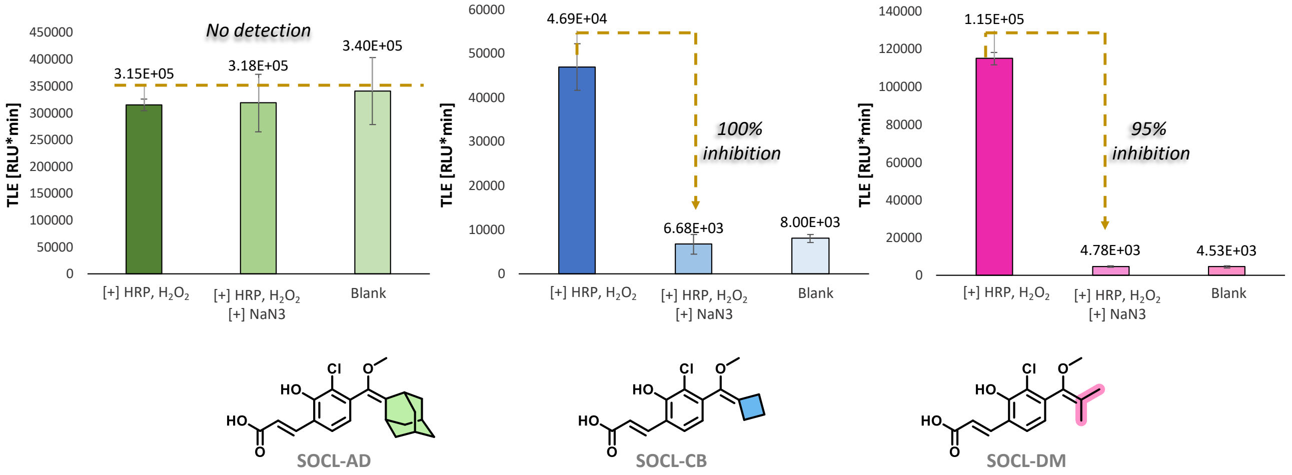

Figure **S29**. Chemiluminescence attenuation by NaN3, a known $^1O_2$ scavenger, and HRP inhibitor. (left) Total light emitted over 200 minutes by **SOCL-AD** [100 µM] in the presence of HRP [5 µg/mL] with and without NaN_3 [10 mM] in PB (50 mM, pH 6.0), 1% DMSO at 30°C, following 20 min incubation before the addition of H_2O_2 [100 µM]. (middle) Total light emitted over 200 minutes by **SOCL-CB** [100 µM] under the same conditions. (right) Total light emitted over 200 minutes by **SOCL-DM** [100 µM] under the same conditions.

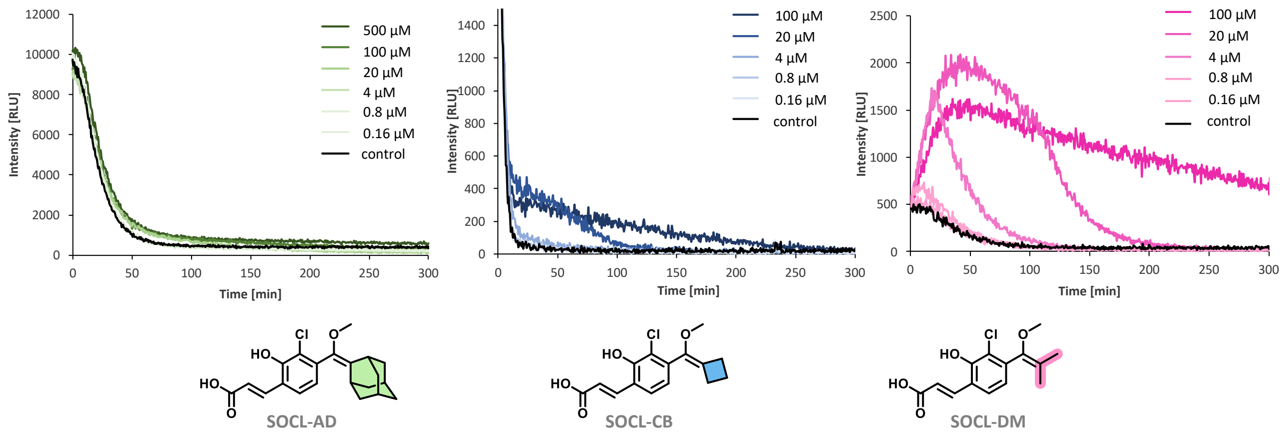

Figure **S30**. Chemiluminescent kinetic profiles of **SOCL-AD** (left), **SOCL-CB** (middle), and **SOCL-DM** (right) probes [100 µM] in the presence of HRP [10 µg/mL] with various concentrations of H_2O_2 [500 - 0.16 µM] in PB (50 mM, pH 6.0), 1% DMSO at 30°C. The detailed assay procedure is mentioned in the experimental protocols section.

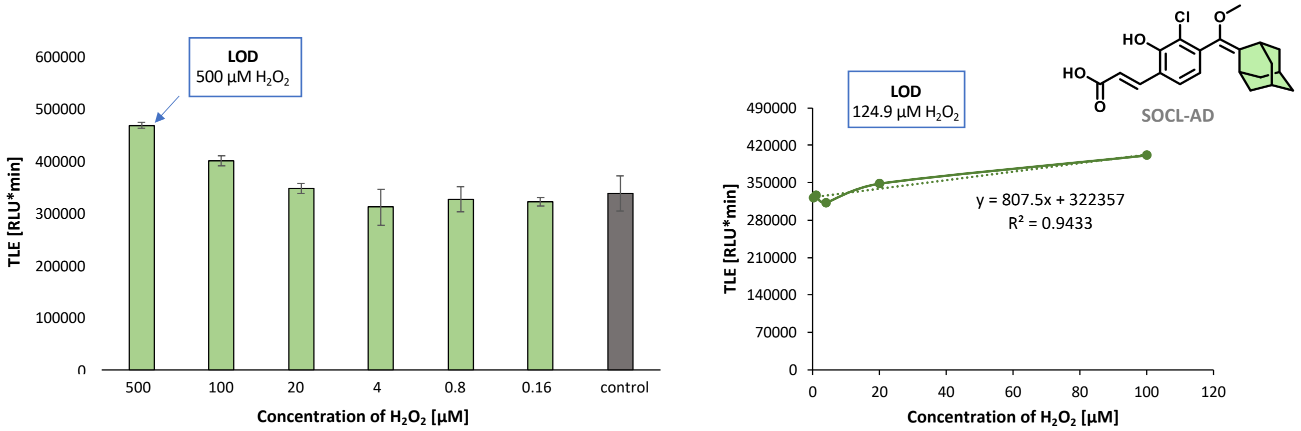

Figure **S31**. Total light emitted (left) and linear calibration curve (right) after 300 minutes of **SOCL-AD** probe [100 µM] in the presence of HRP [10 µg/mL] with various concentrations of H_2O_2 [500 - 0.16 µM] in PB (50 mM, pH 6.0), 1% DMSO at 30°C. The limit of detection (LOD) was calculated using the same approach as described in Figure **S7**. The detailed assay procedure is mentioned in the experimental protocols section.

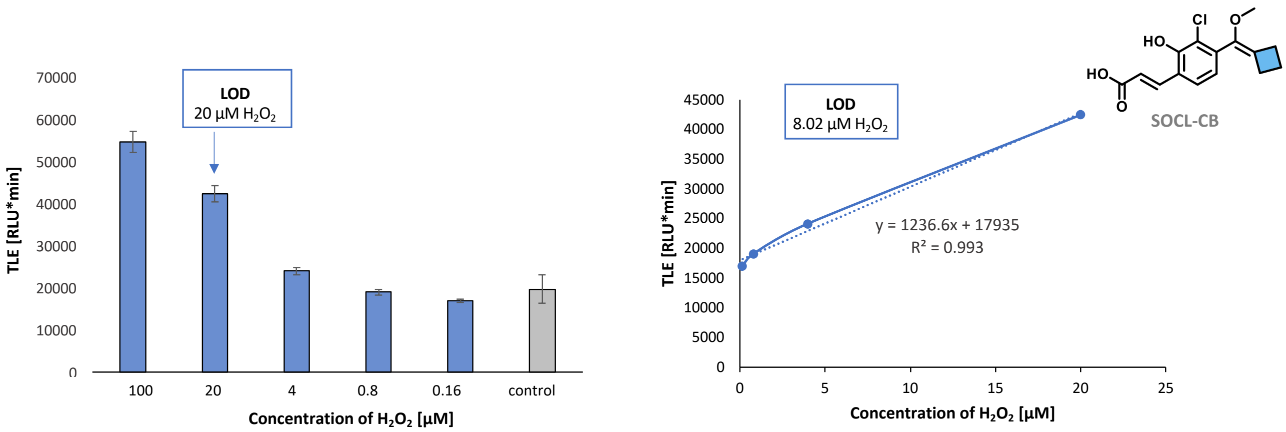

Figure **S32**. Total light emitted (left) and linear calibration curve (right) after 300 minutes of **SOCL-CB** probe [100 μM] in the presence of HRP [10 μg/mL] with various concentrations of H_2O_2 [100 - 0.16 μM] in PB (50 mM, pH 6.0), 1% DMSO at 30°C. The limit of detection (LOD) was calculated using the same approach as described in Figure **S7**. The detailed assay procedure is mentioned in the experimental protocols section.

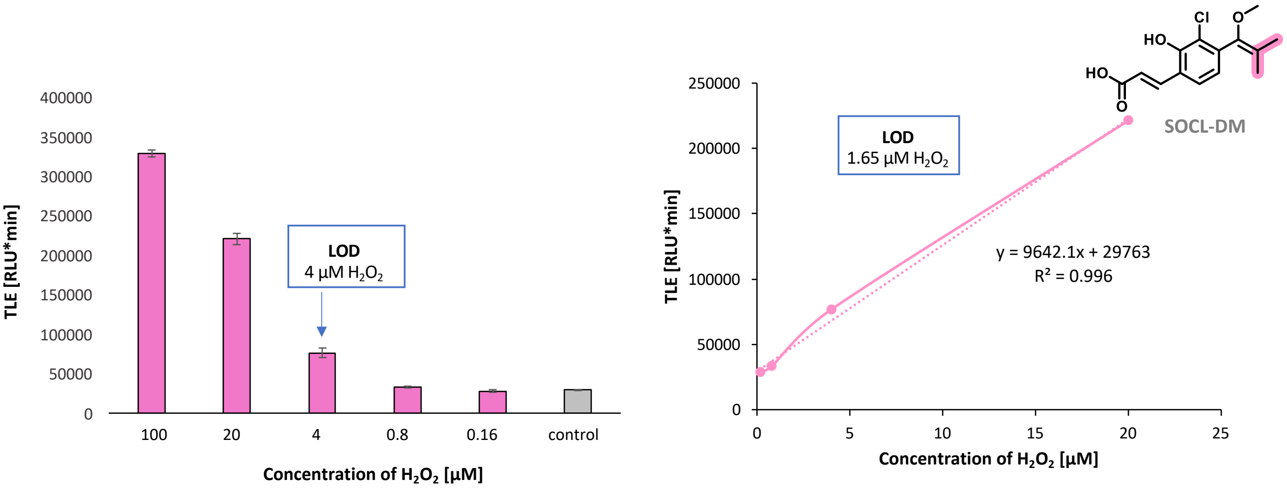

Figure **S33**. Total light emitted (left) and linear calibration curve (right) after 300 min of **SOCL-DM** probe [100 μM] in the presence of HRP [10 μg/mL] with various concentrations of H_2O_2 [100 - 0.16 μM] in PB (50 mM, pH 6.0), 1% DMSO at 30°C. The limit of detection (LOD) was calculated using the same approach as described in Figure **S7**. The detailed assay procedure is mentioned in the experimental protocols section.

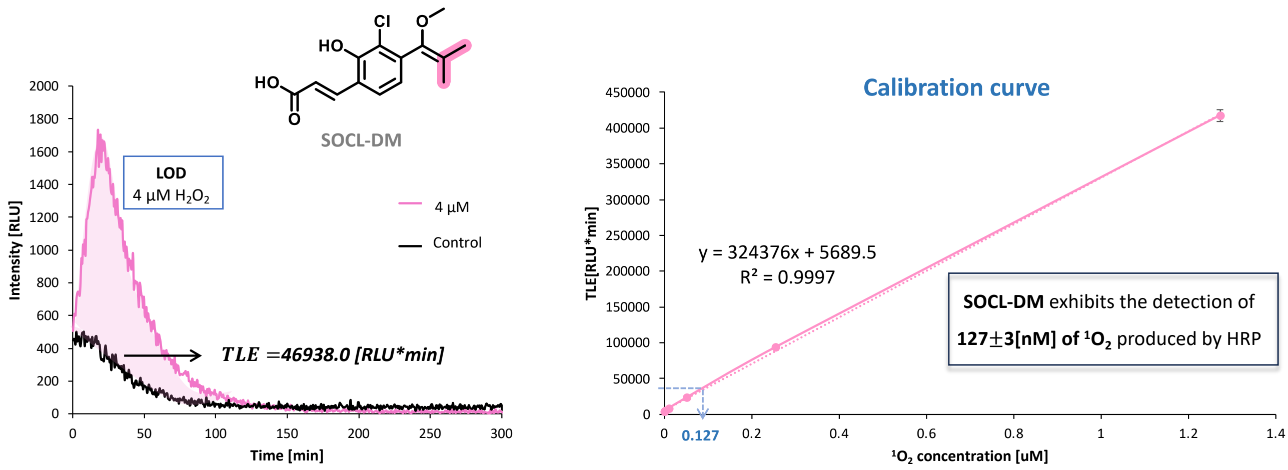

Figure **S34**. (Left) Chemiluminescence kinetic profile of **SOCL-DM** probe [100 µM] in the presence of HRP [10 µg/mL] with and without H_2O_2 [4 µM] in PB (50 mM, pH 6.0), 1% DMSO at 30°C. The total light emitted at the LOD concentration of H_2O_2 [4 µM] - $TLE_{4\ \mu M}$ - is obtained by subtracting the value measured in the absence of H_2O_2 from the value measured in its presence. (Right) Calibration curve of total light emitted (TLE) by **SOCL-DM** probe as a function of singlet oxygen ($^1O_2$) concentration. The calibration curve was generated using the TLE values measured after 60 minutes for the **SOCL-DM** probe [100 µM] with various concentrations of EP-1 [2 – $1.28 \cdot 10^{-4}$ µM]. EP-1 concentrations were then converted to $^1O_2$ concentrations by multiplying in 0.636, yielding a range of [1.27 – $8.14 \cdot 10^{-5}$ µM]. To calculate the LOD for $^1O_2$, $TLE_{4\ \mu M}$ was plotted on the linear calibration curve. The detailed assay procedure is mentioned in the experimental protocols section.

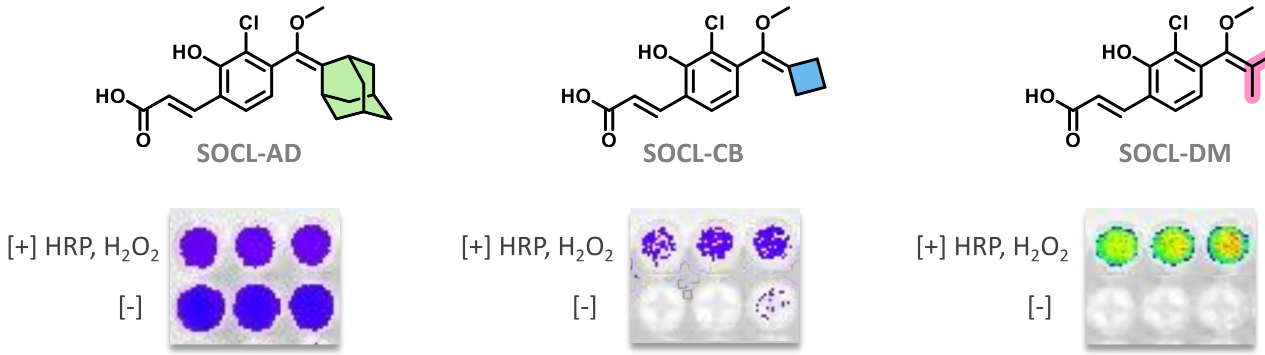

Figure **S35**. Chemiluminescence imaging using IVIS® Lumina for **SOCL-AD** (left), **SOCL-CB** (middle), and **SOCL-DM** (right) [100 µM] in the presence and absence of HRP [50 µg/mL] with and without H_2O_2 [500 µM] in PB (50 mM, pH 6.0), 1% DMSO at 30°C. The images were taken after 20 minutes of incubation with an exposure time of 50 sec. The detailed assay procedure is mentioned in the experimental protocols section.

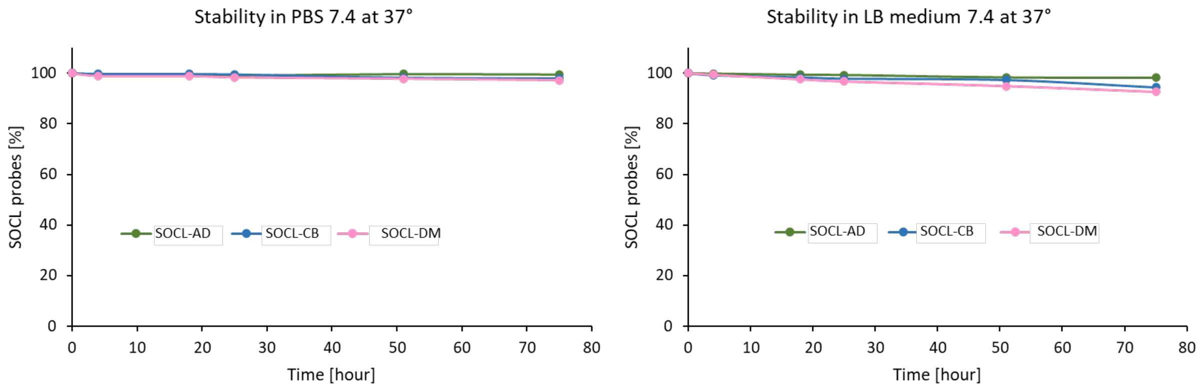

Figure **S36.** Stability of **SOCL-AD** (green), **SOCL-CB** (blue), and **SOCL-DM** (pink) probes in PBS (pH 7.4) and LB medium at 37 °C. The probes [300 µM] were incubated in either PBS (100 mM, pH 7.4, with 1% DMSO; left) or LB medium (right) for 75 hours at 37 °C. During this time, samples were taken at different time points and injected into RP-HPLC (30–100% ACN in water, 0.1% TFA) to monitor decomposition. Decomposition was quantified based on the integration of the SOCL probe and its decomposition product. The results show that all SOCL probes remain highly stable under these conditions over the tested period.

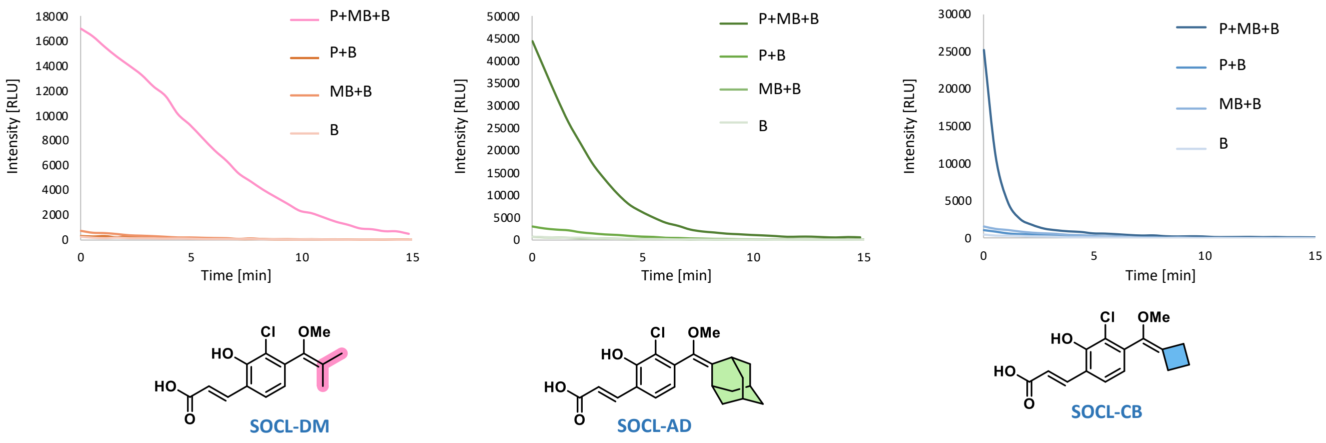

Figure **S37.** Intracellular detection of singlet oxygen in *Bacillus subtilis*. Kinetic profiles of chemiluminescence from (left) probe SOCL-DM, (middle) probe SOCL-AD, and (right) probe SOCL-CB [100 µM] following incubation with and without methylene blue (MB) [50 µM] in *B. subtilis* (OD_{600} = 0.85) for 3 hours. After incubation, the samples were washed twice with PBS (pH 7.4) and irradiated for 1 minute. Detailed assay procedures are provided in the bacterial experimental protocols section. **Abbreviations:** P – probe, MB – methylene blue, B – Bacteria.

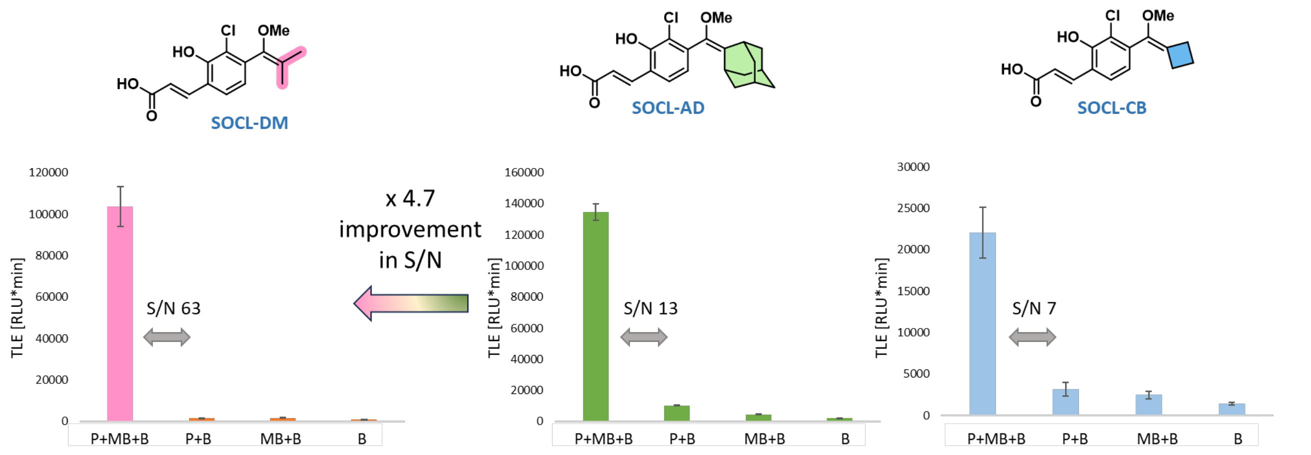

Figure **S38.** Intracellular detection of singlet oxygen in *Bacillus subtilis*. Total light emitted after 15 minutes after treatment with (left) probe SOCL-DM, (middle) probe SOCL-AD, and (right) probe SOCL-CB (100 µM each), with or without methylene blue (MB, 50 µM), in *B. subtilis* (OD_{600} = 0.85) following 3 hours of incubation. Samples were washed twice with PBS (pH 7.4) and irradiated for 1 minute. The results demonstrate that SOCL-DM exhibits a 4.7-fold higher sensitivity for detecting intracellular singlet oxygen compared to SOCL-AD in *B. subtilis*. Detailed procedures are described in the bacterial experimental protocols section. **Abbreviations:** P – probe, MB – methylene blue, B – bacteria.

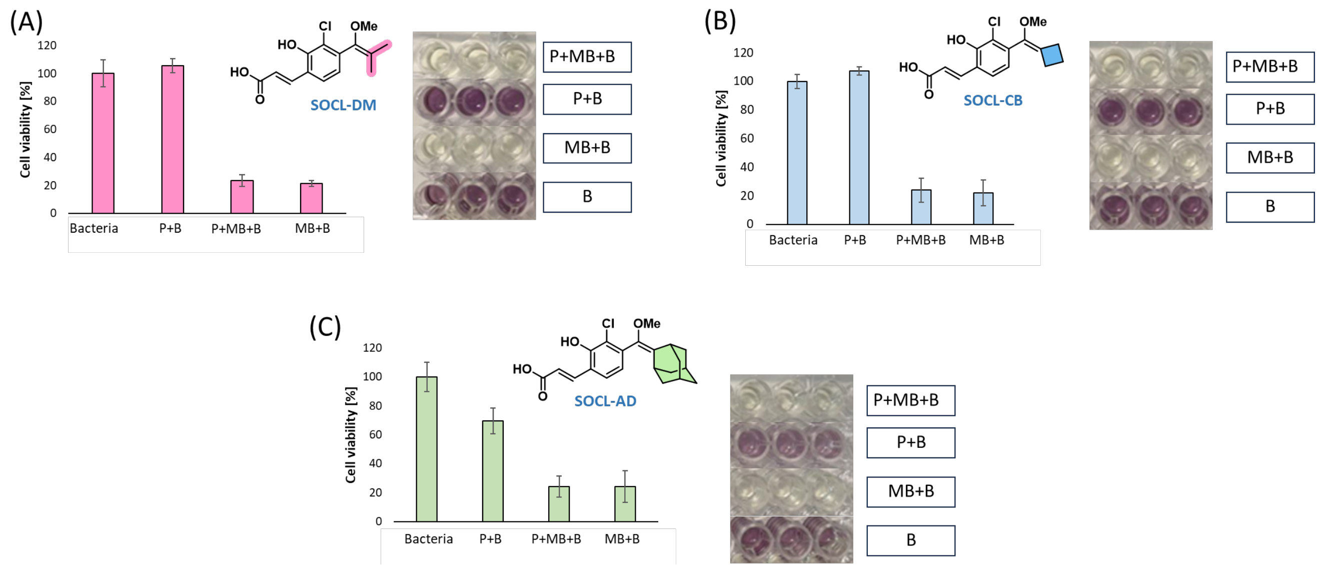

Figure **S39.** MTT assay of *B. subtilis* (OD_{600} = 0.85) after incubation with (A) probe SOCL-DM, (B) probe SOCL-CB, and (C) probe SOCL-AD at 100 µM, methylene blue (MB, 10 µM), or both, followed by 5 minutes of LED lamp irradiation. The results indicate minimal cytotoxicity of the probes, particularly SOCL-DM and SOCL-CB, towards bacterial cells upon irradiation. Representative MTT images are shown for each condition. Detailed procedures are described in the bacterial experimental protocols section. **Abbreviations:** P – probe, MB – methylene blue, B – bacteria.

Appendix III-NMR and MS Spectra

Probe 2 – SOCL-CH

^{1}H-NMR

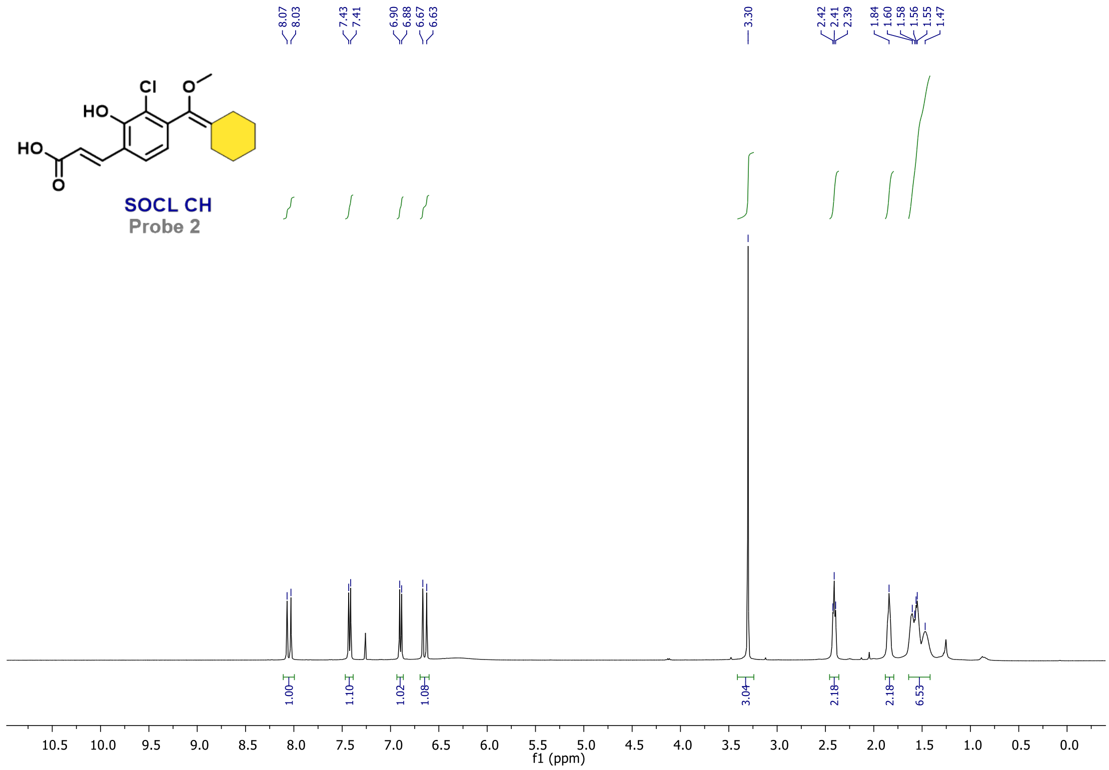

^{13}C-NMR

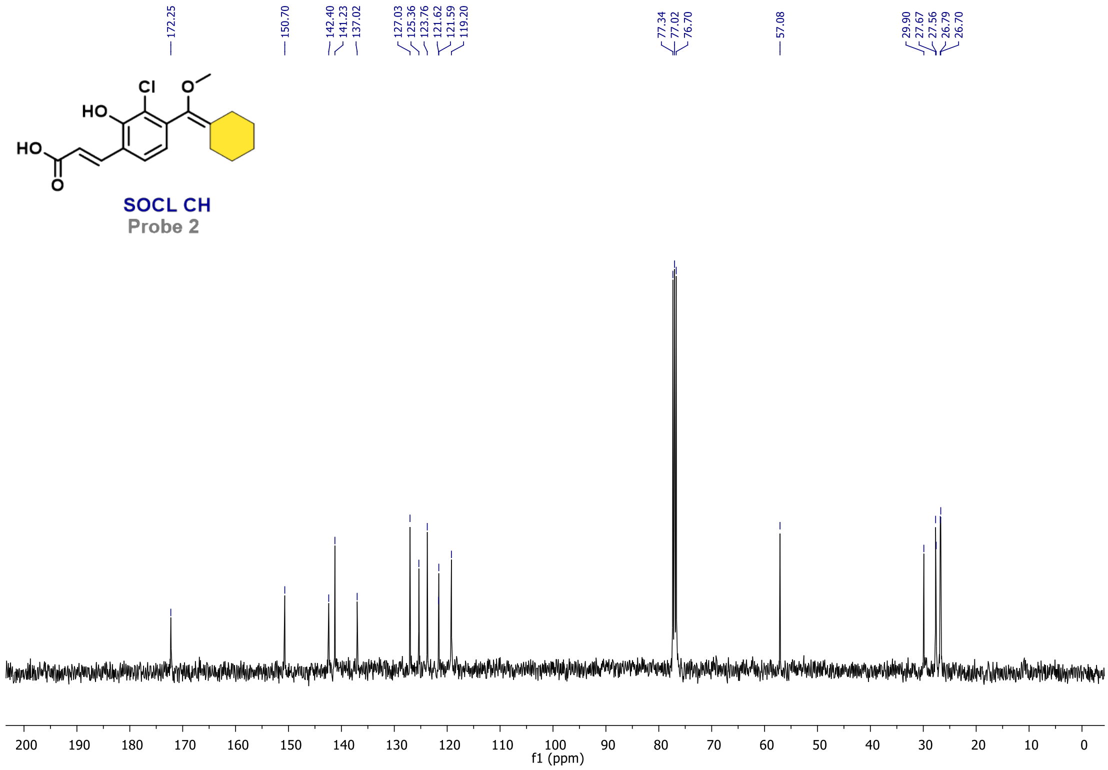

Mass spectra

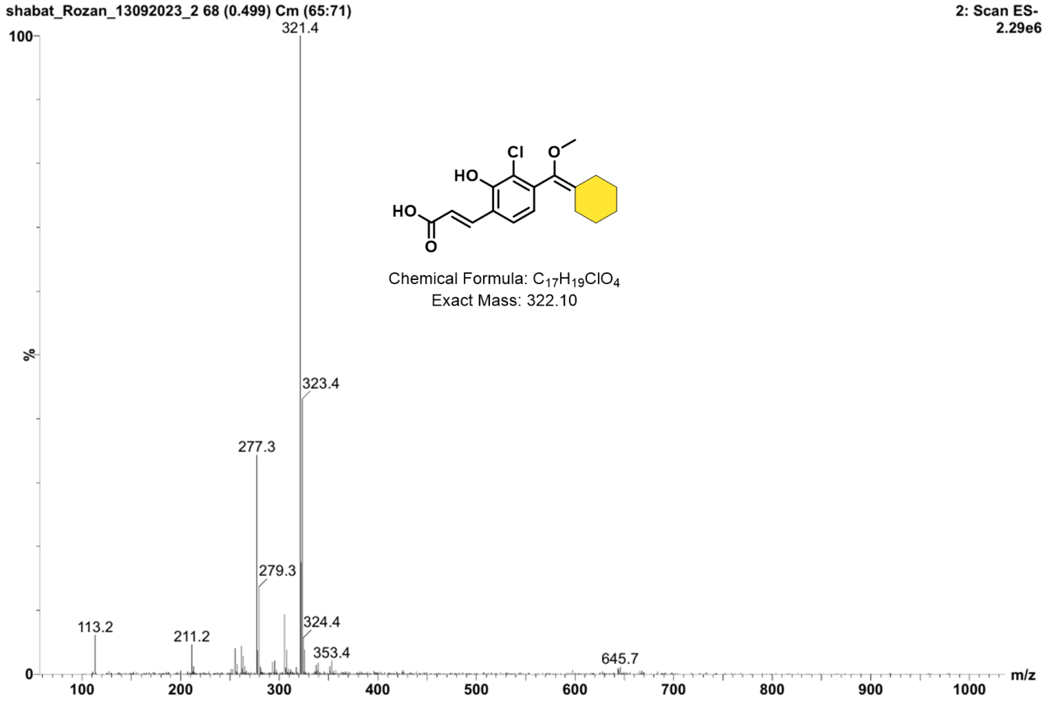

Probe 3 – SOCL-CB

^{1}H-NMR

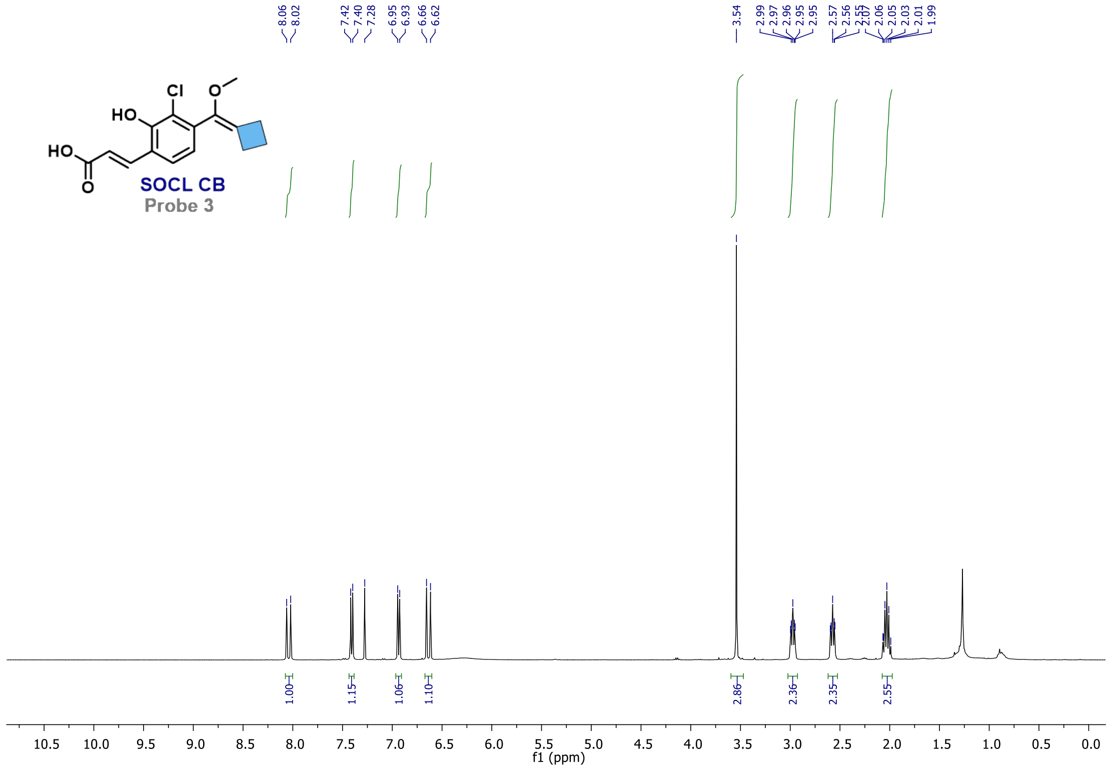

^{13}C-NMR

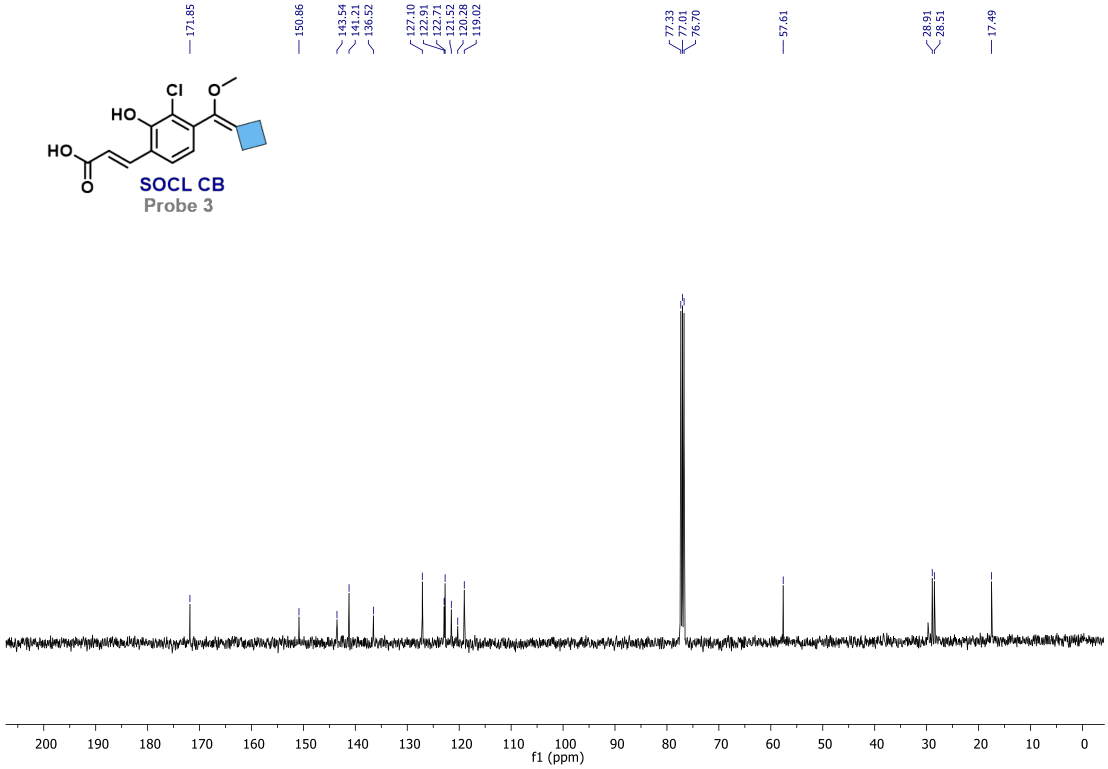

Mass spectra

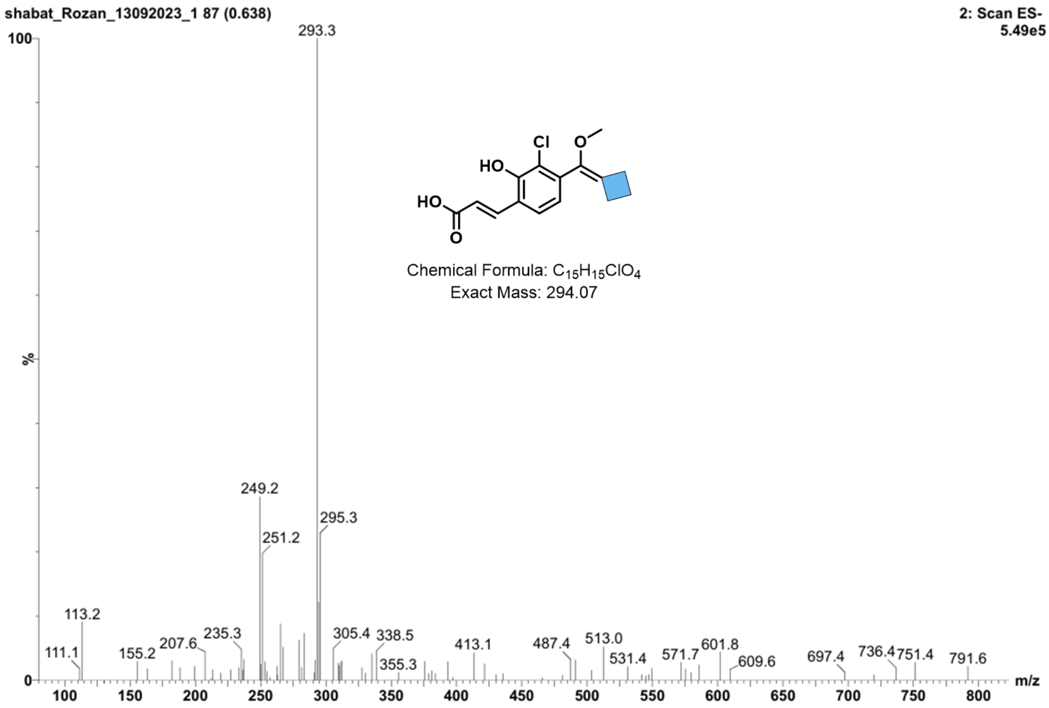

Compound 4a

^{1}H-NMR

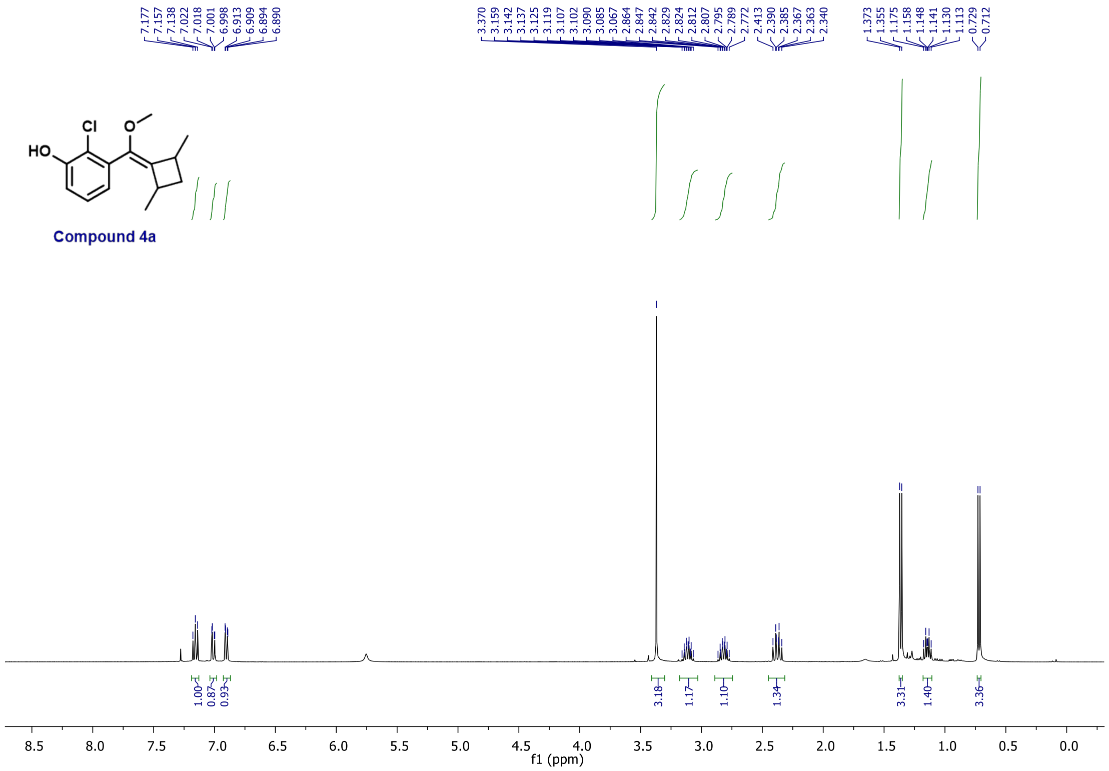

^{13}C-NMR

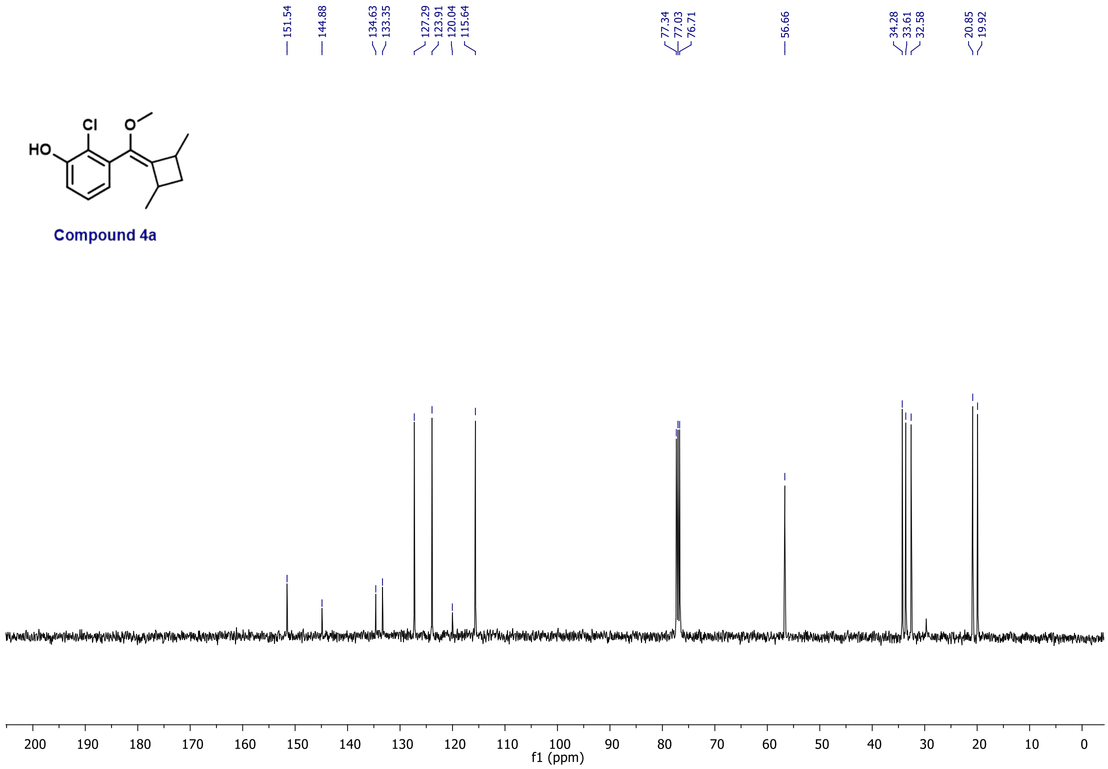

Mass spectra

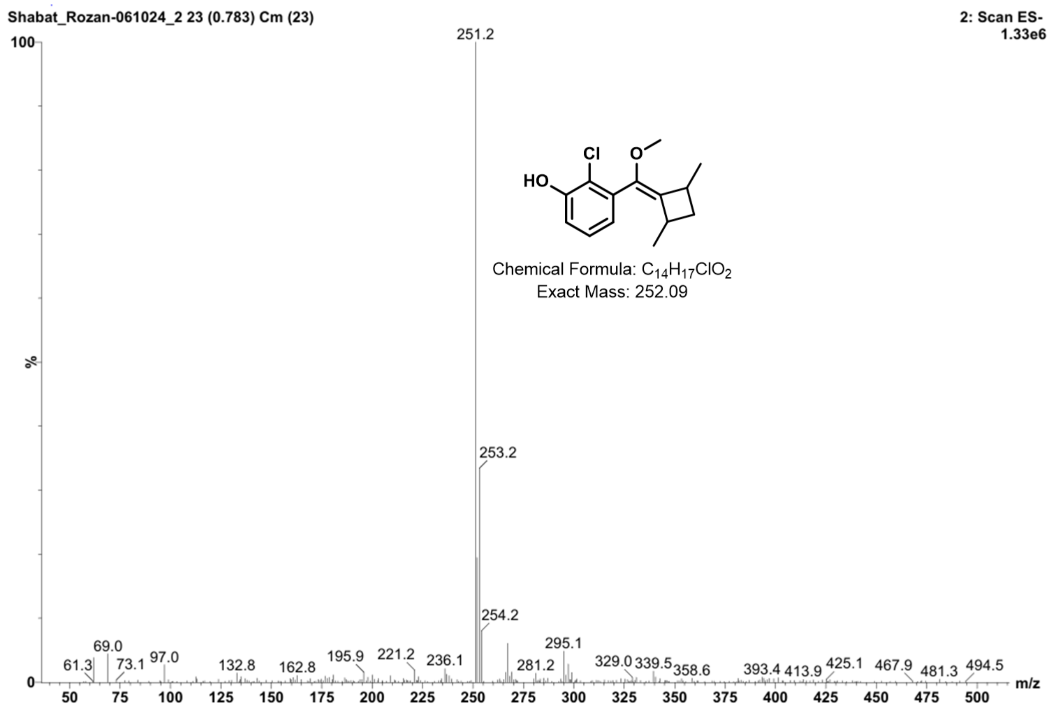

Compound 4b

^{1}H-NMR

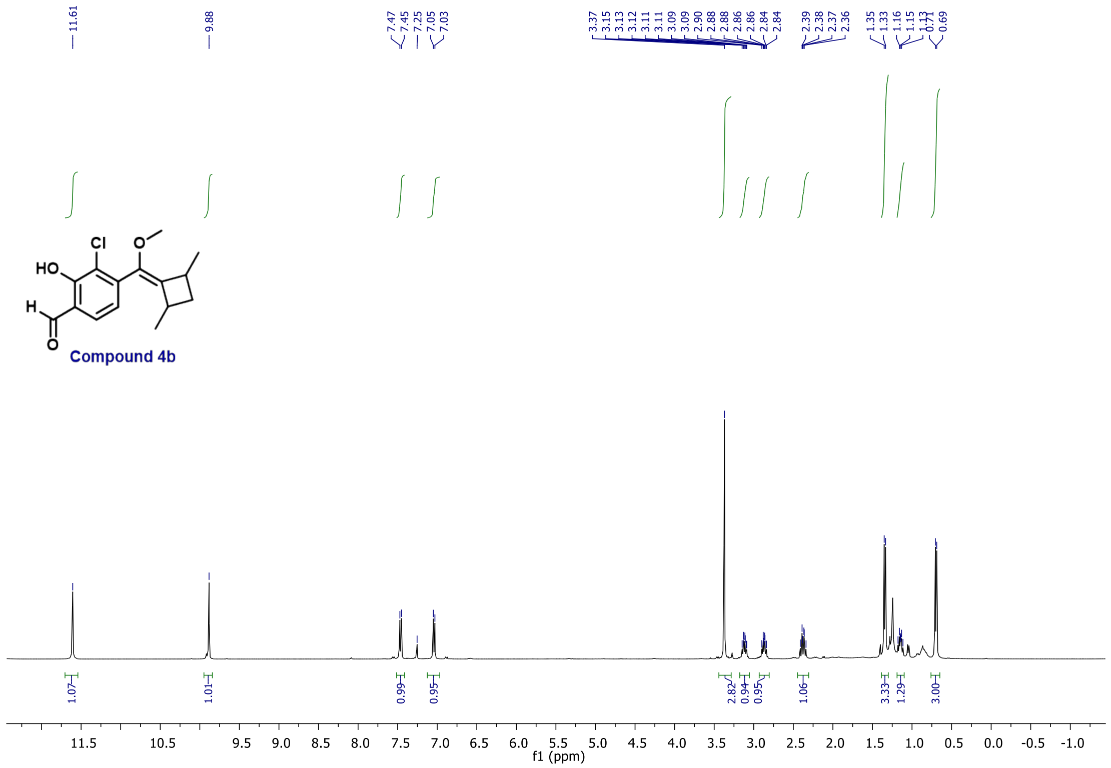

^{13}C-NMR

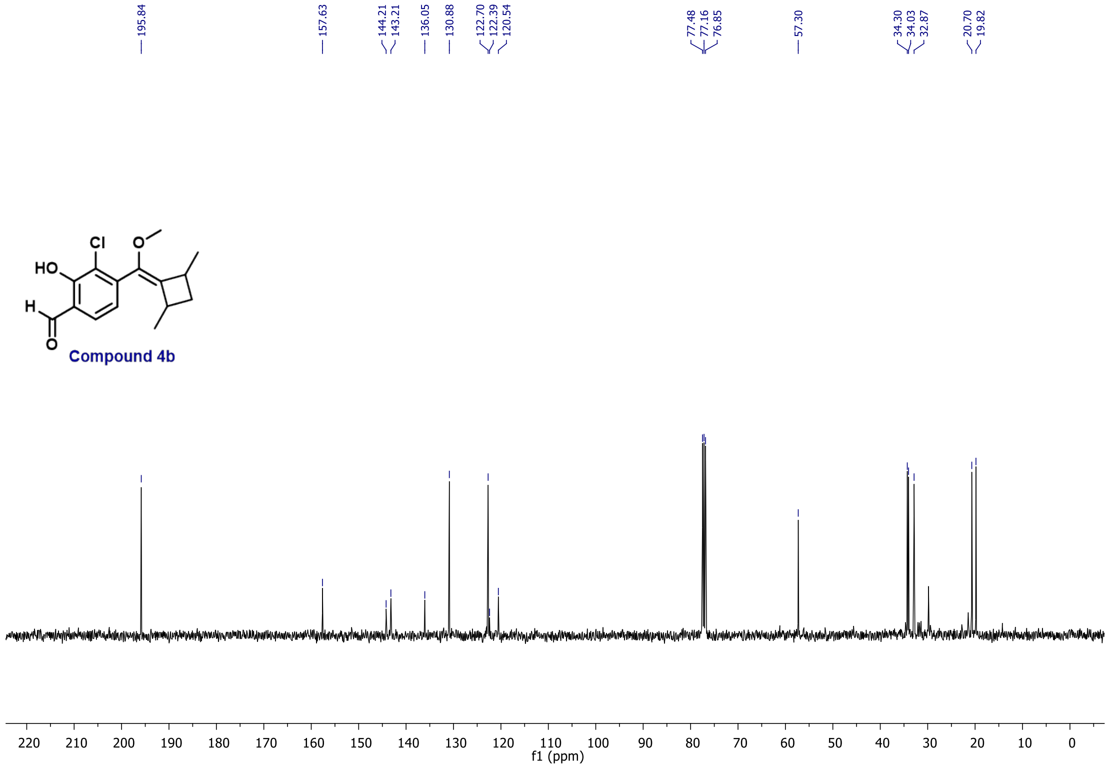

Mass spectra

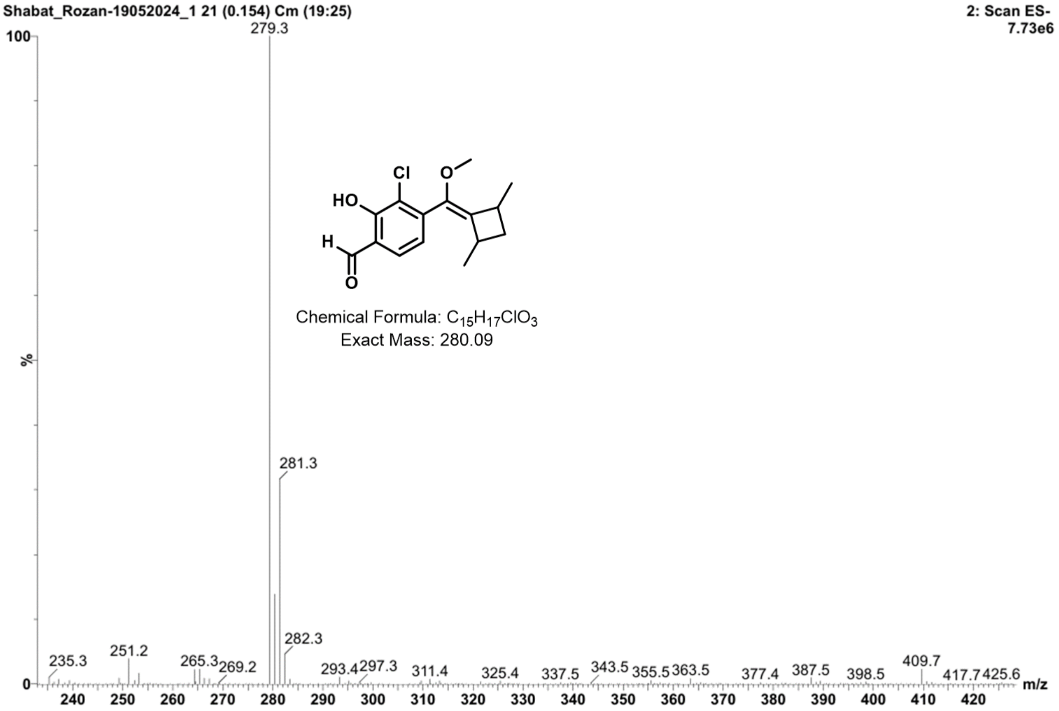

Compound 4b

^{1}H-NMR

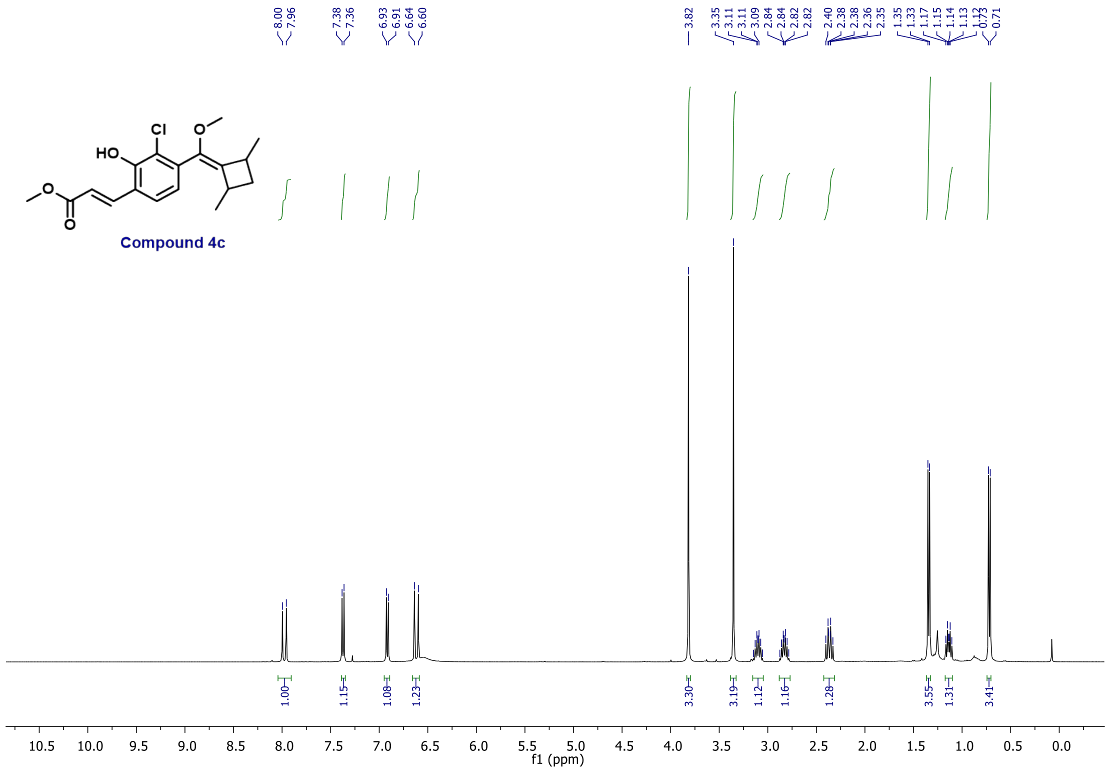

^{13}C-NMR

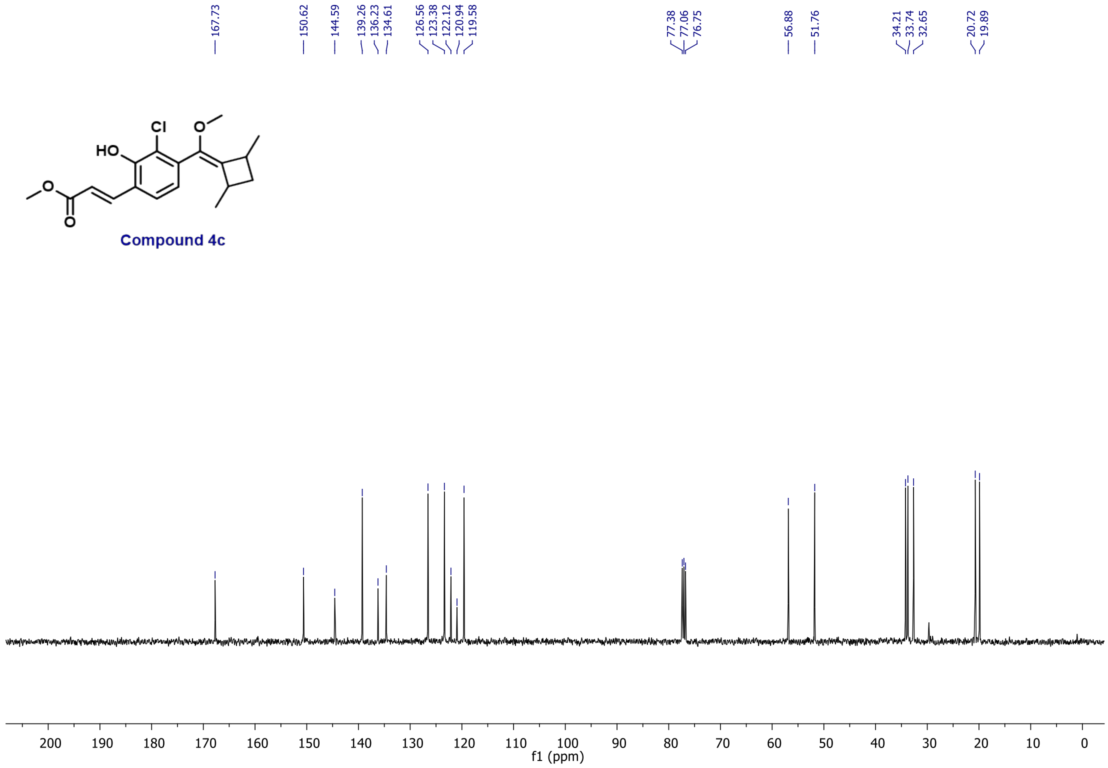

Mass spectra

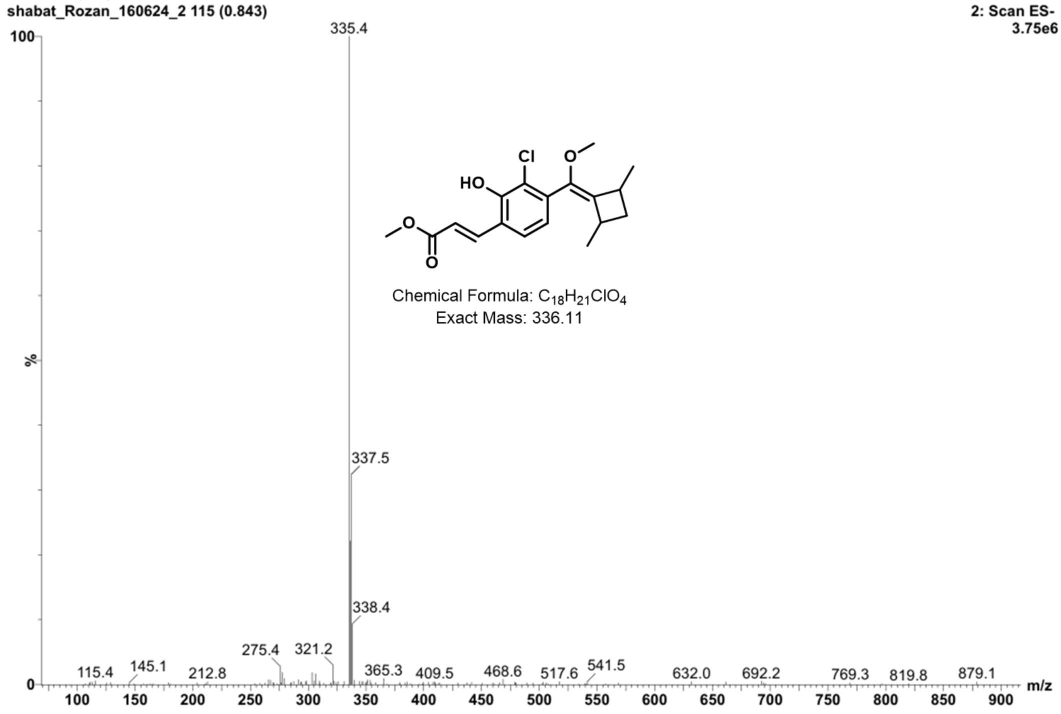

Probe 4 – SOCL-DM-CB

^{1}H-NMR

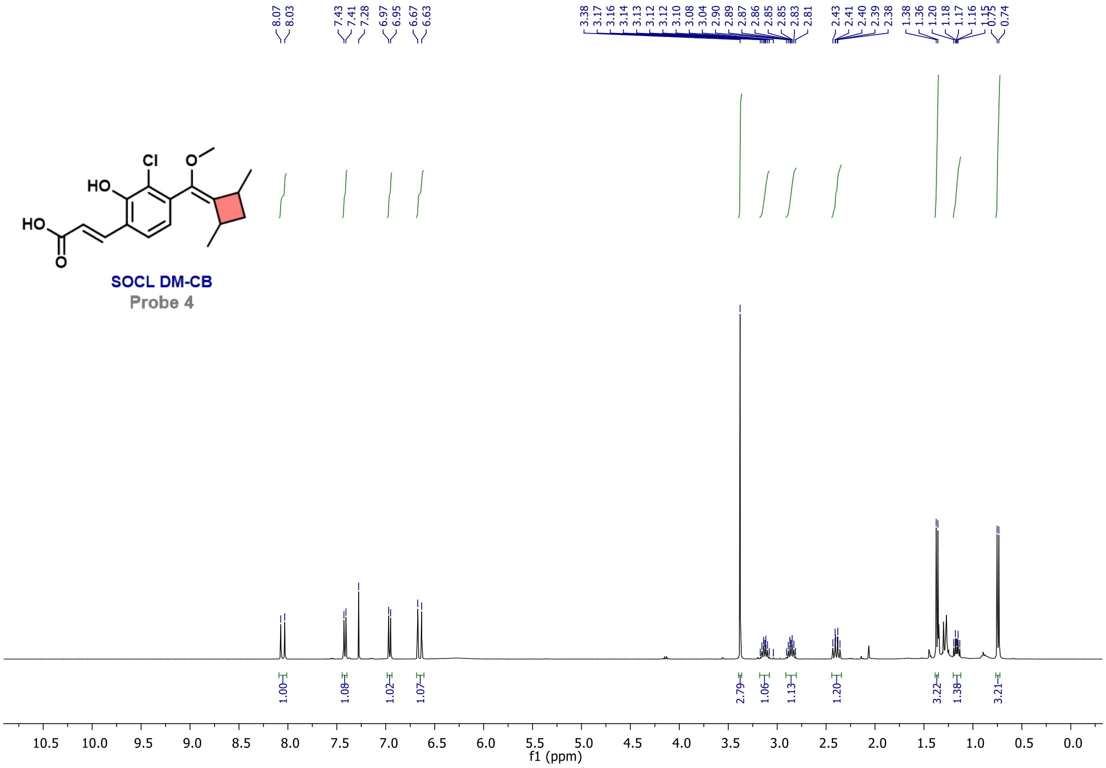

^{13}C-NMR

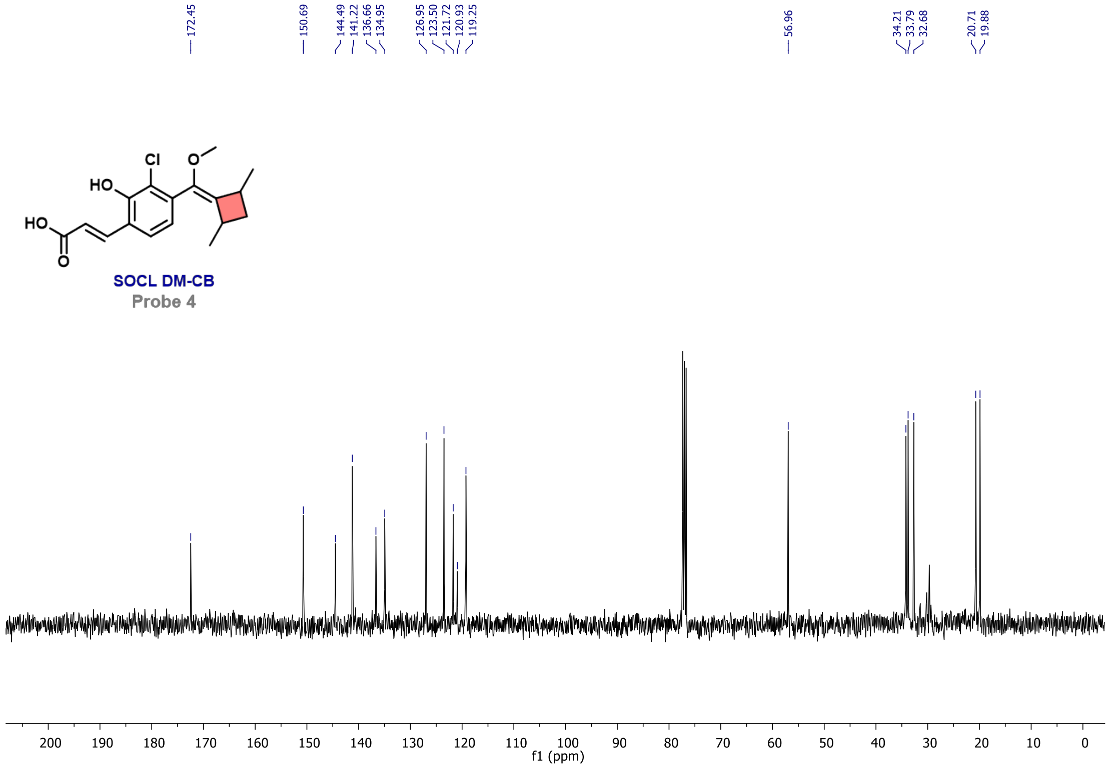

Mass spectra

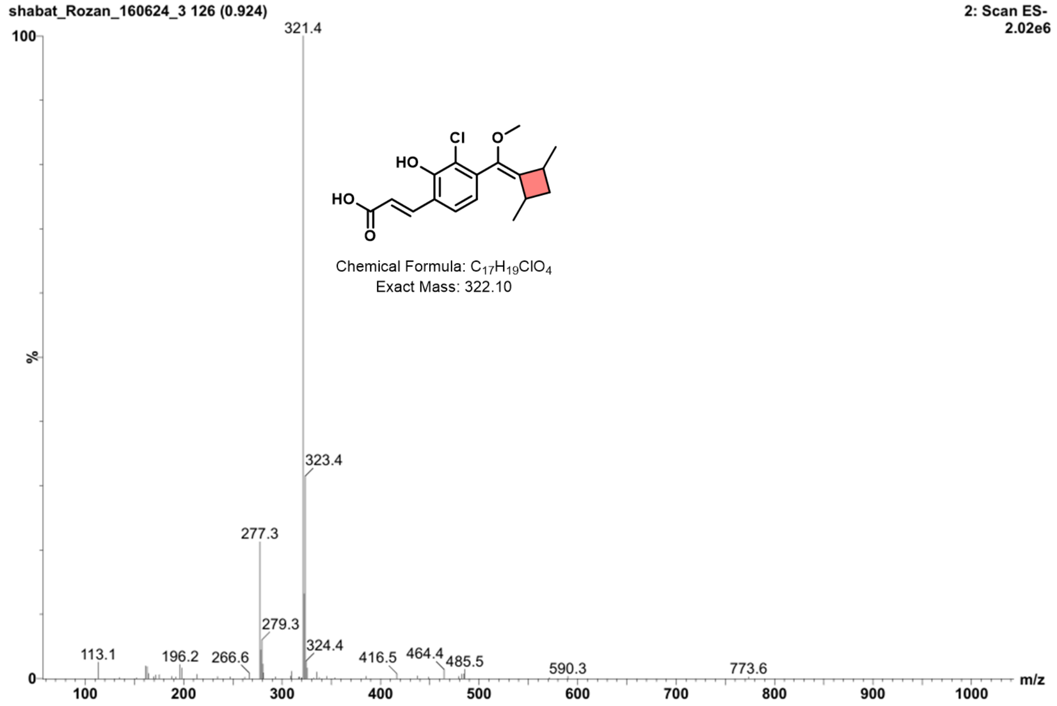

Compound 5b

^{1}H-NMR

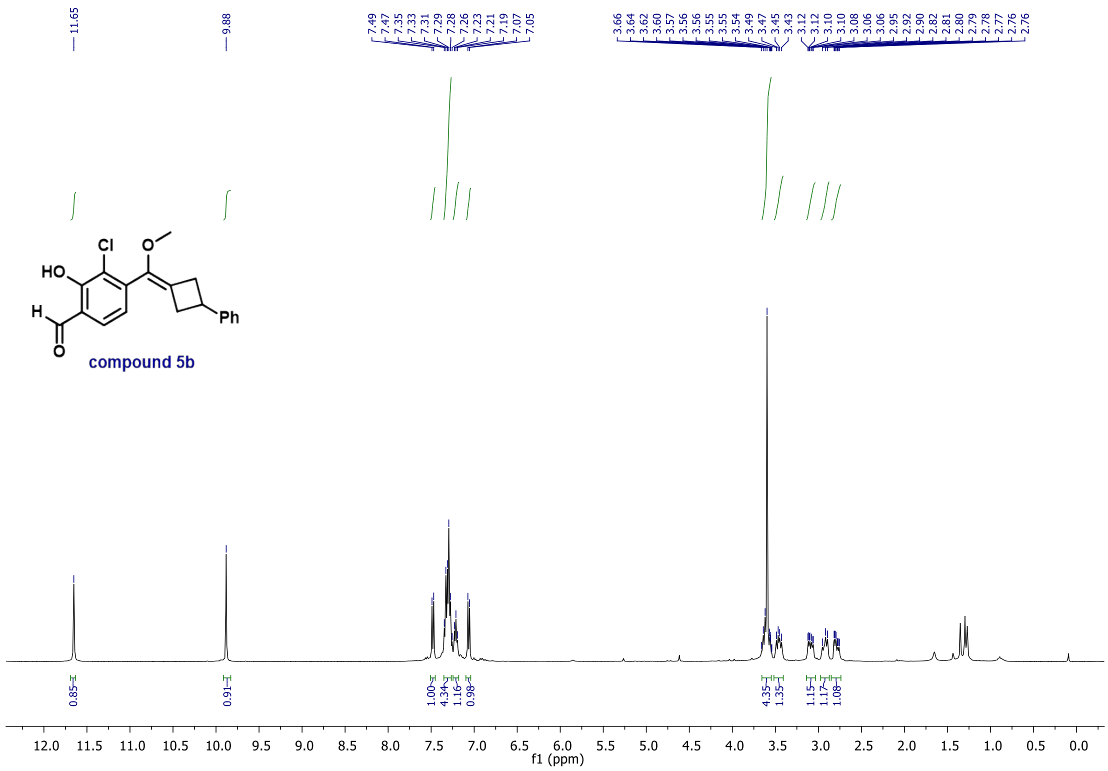

^{13}C-NMR

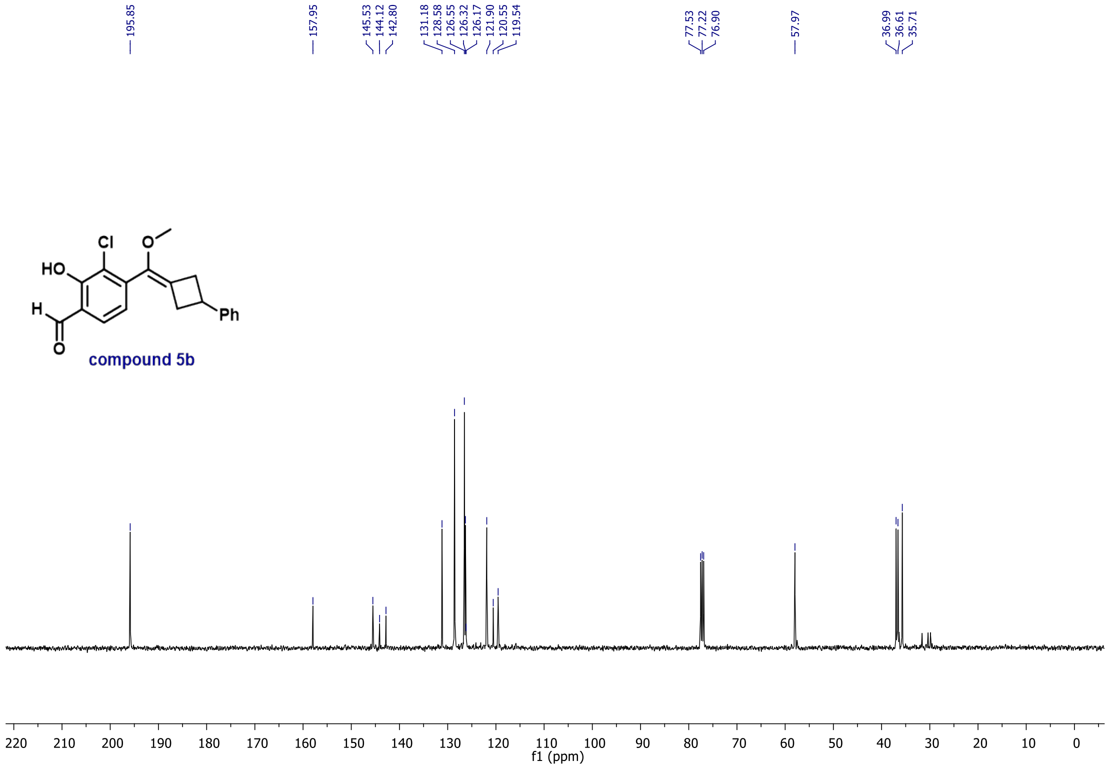

Mass spectra

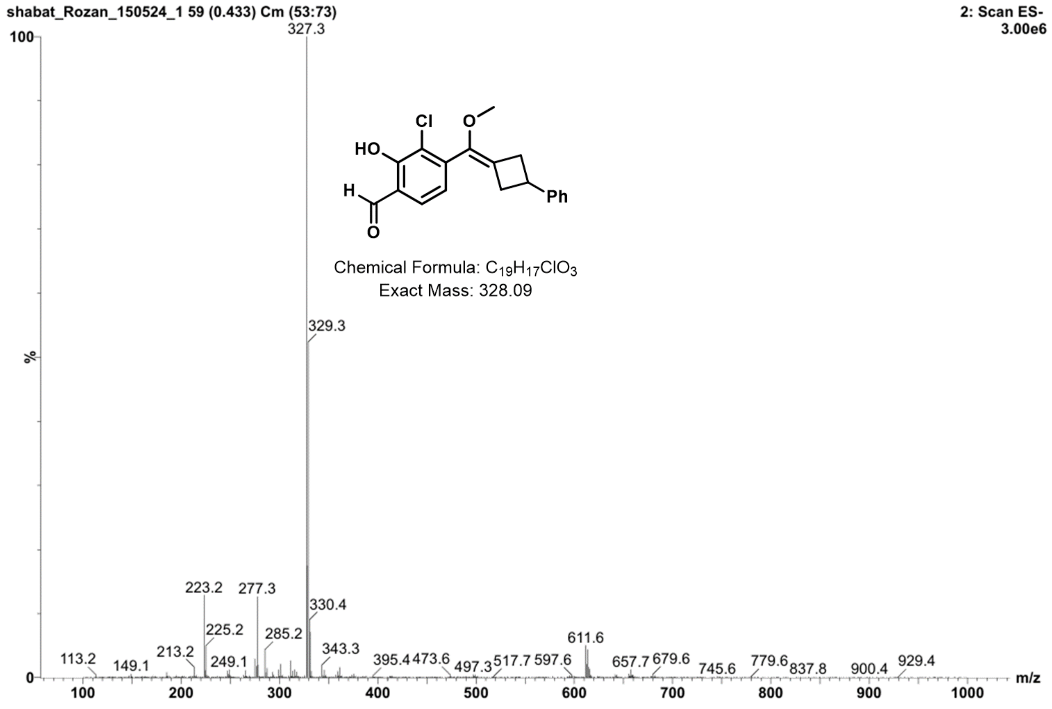

Compound 5c

^{1}H-NMR

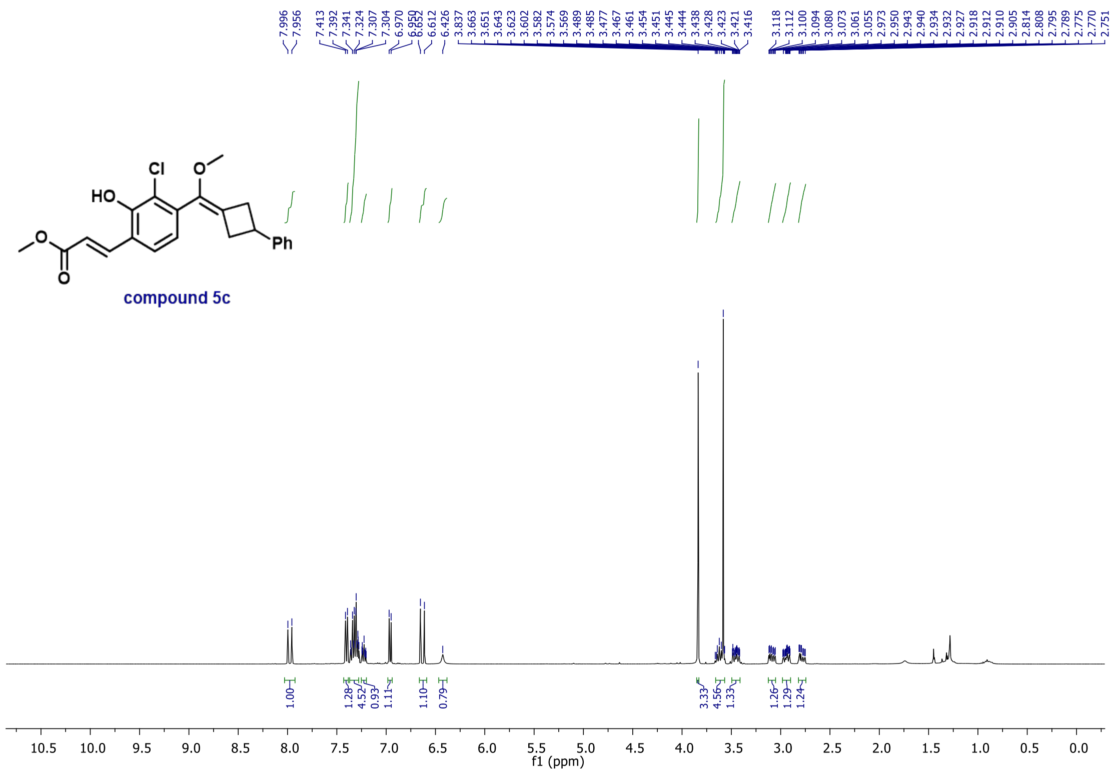

^{13}C-NMR

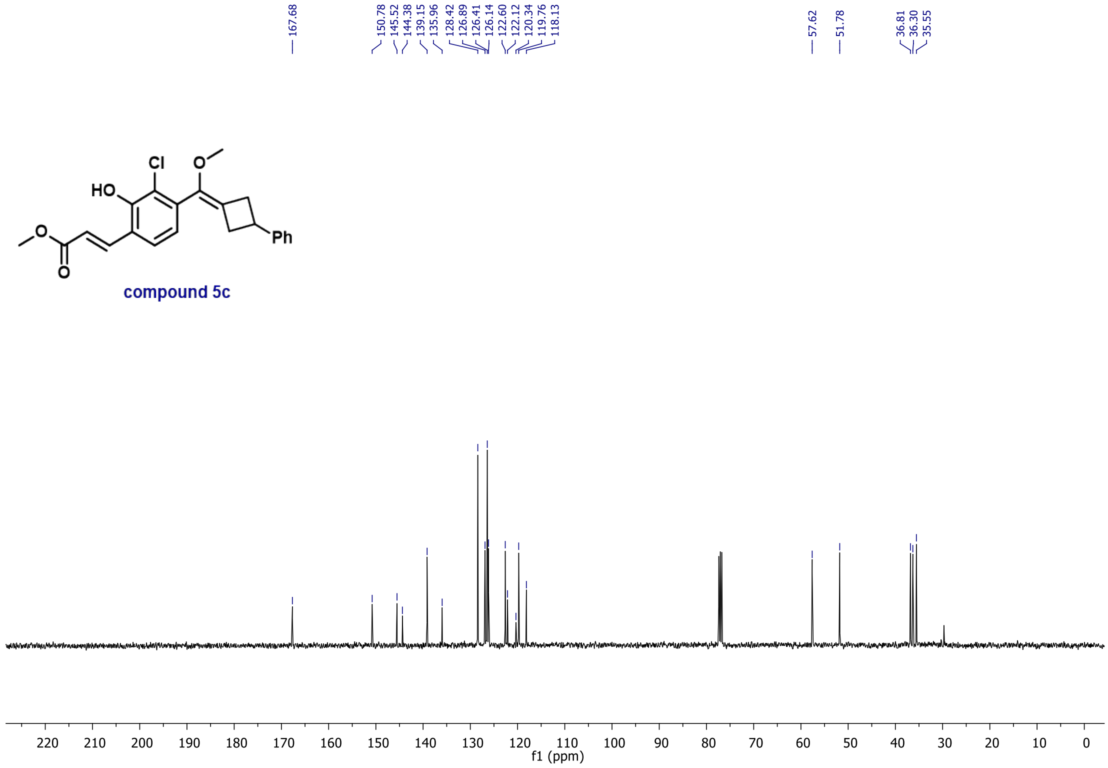

compound 5c

Mass spectra

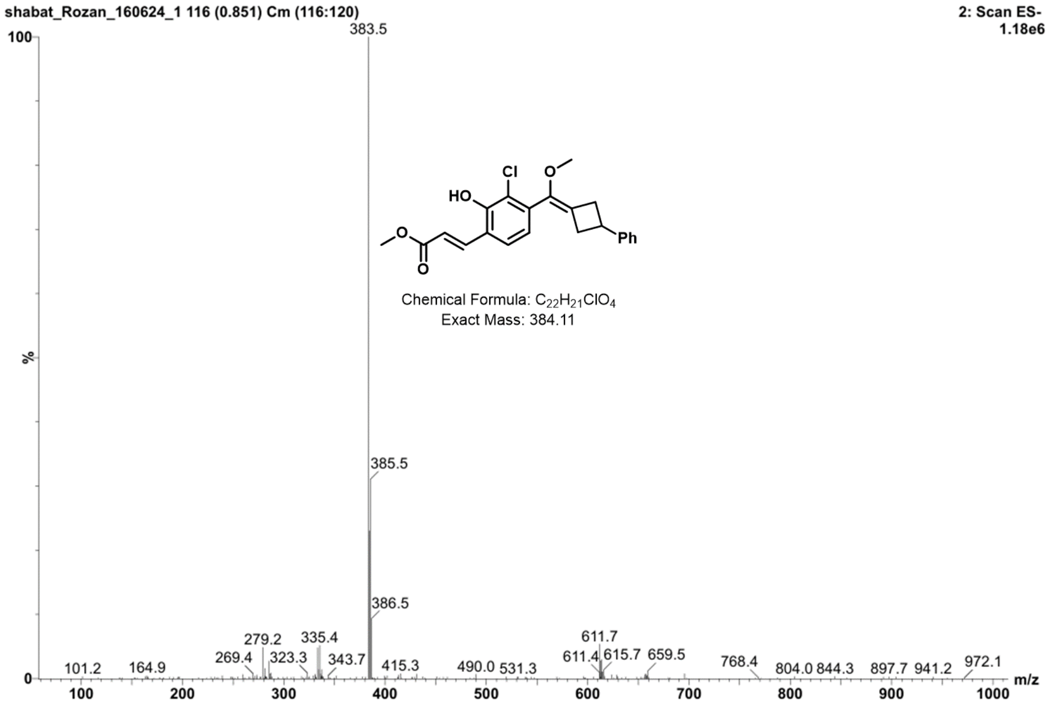

Probe 5 – SOCL-Ph-CB

^{1}H-NMR

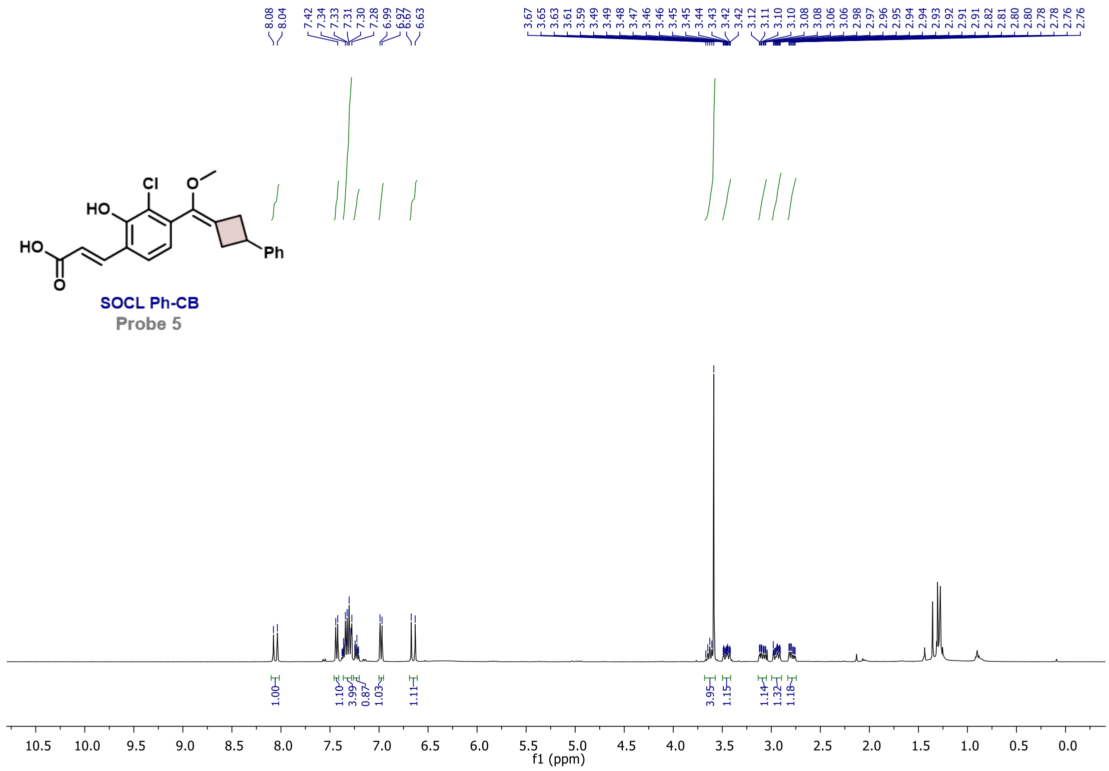

^{13}C-NMR

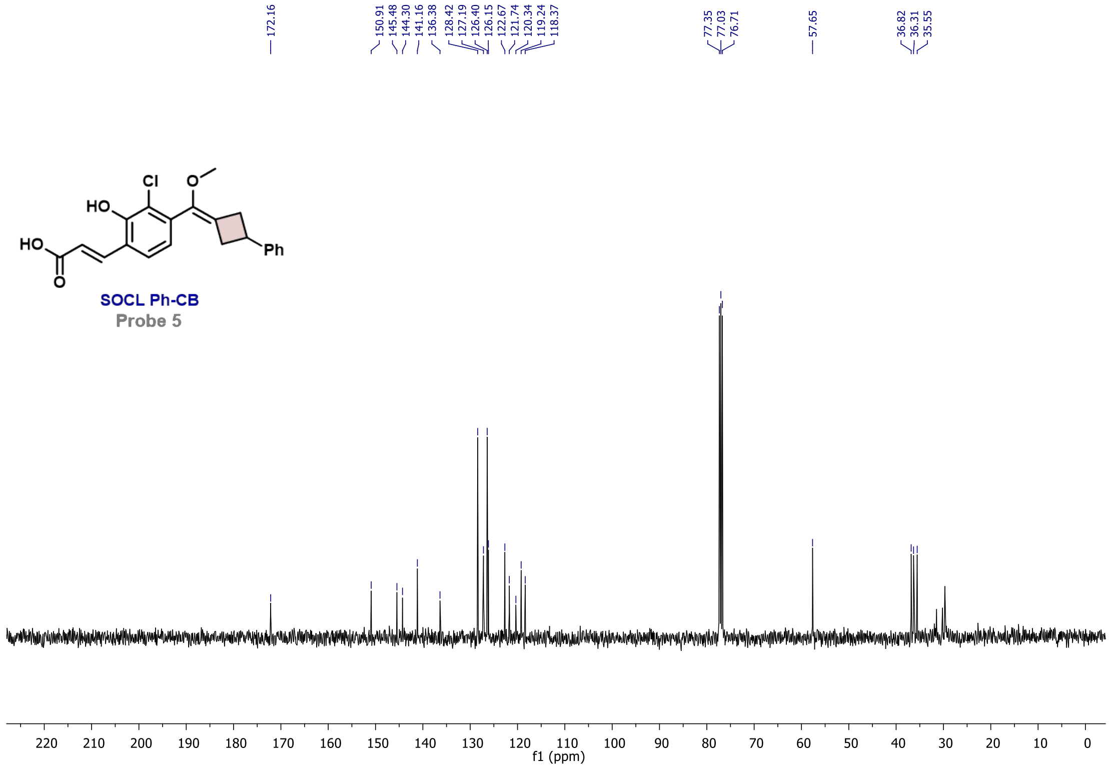

Mass spectra

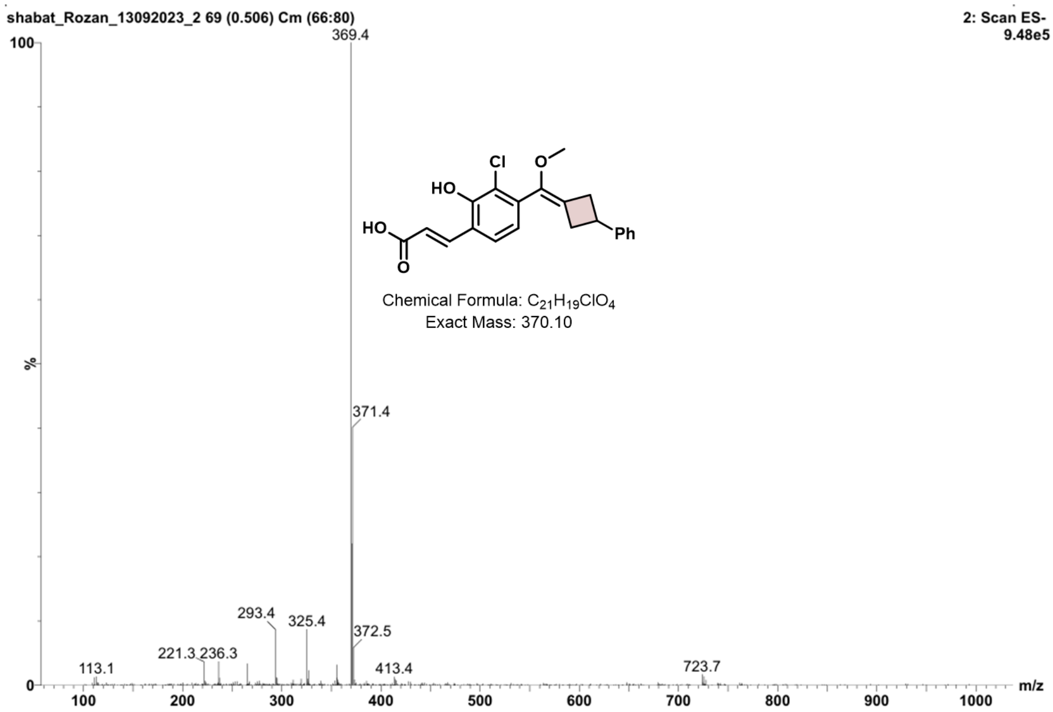

Compound 6b

^{1}H-NMR

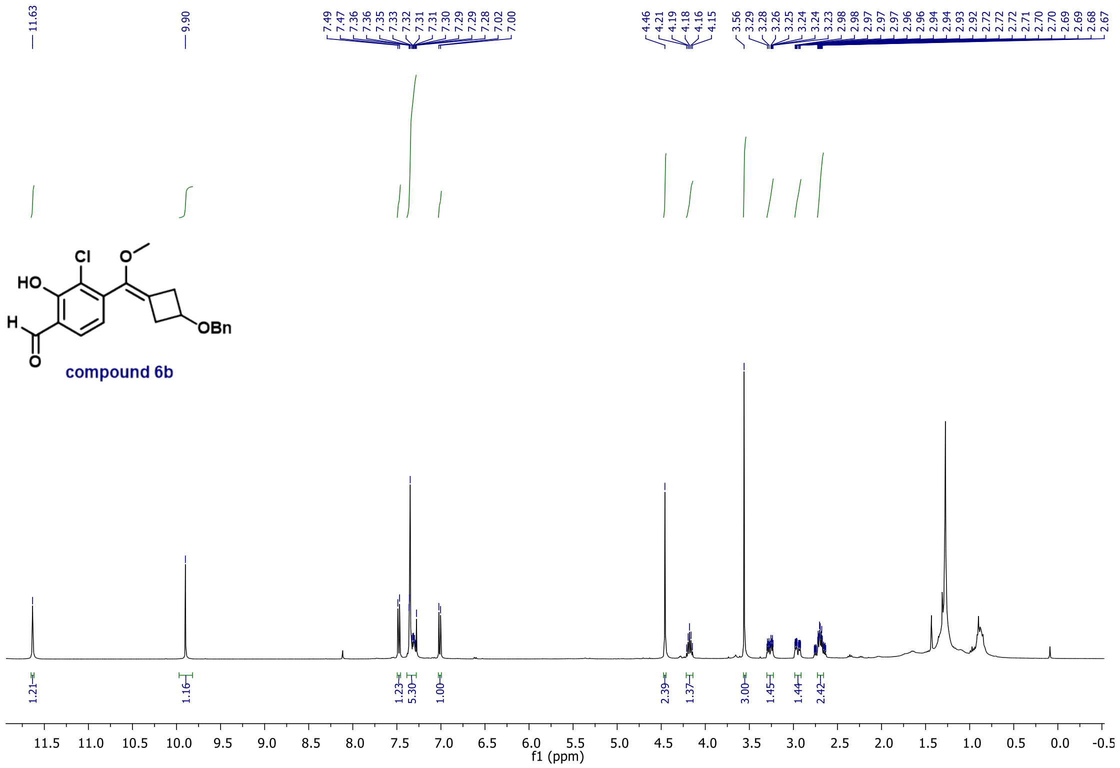

^{13}C-NMR

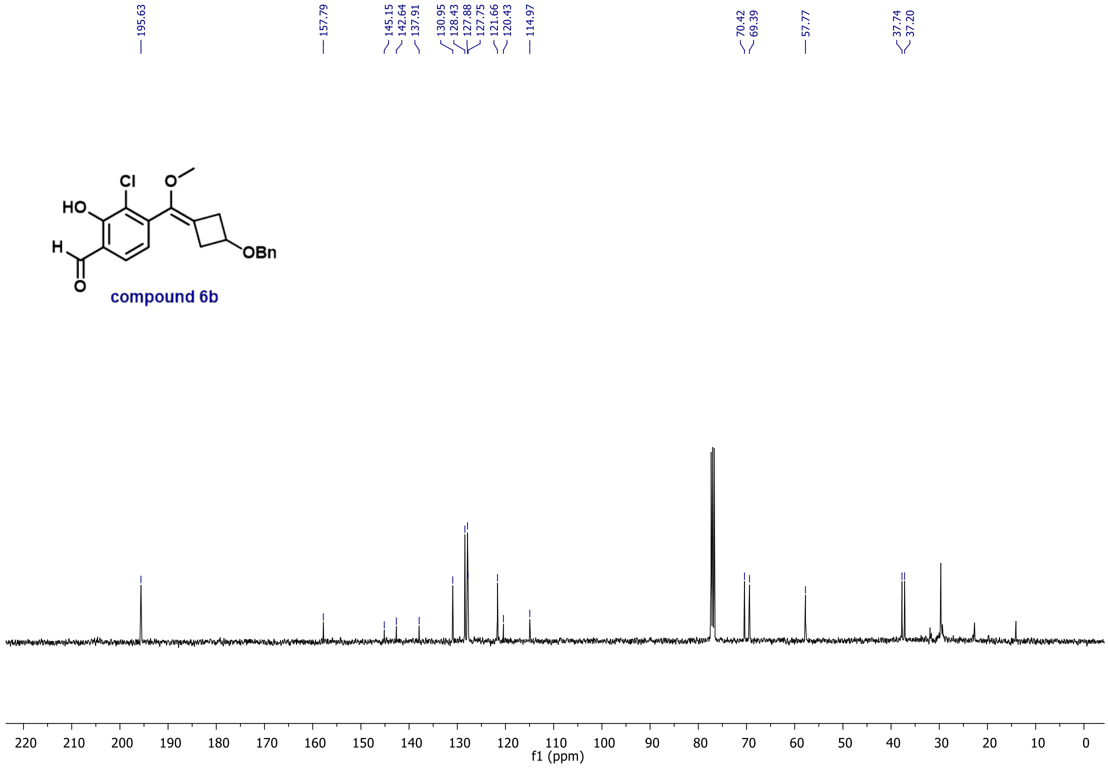

compound 6b

Mass spectra

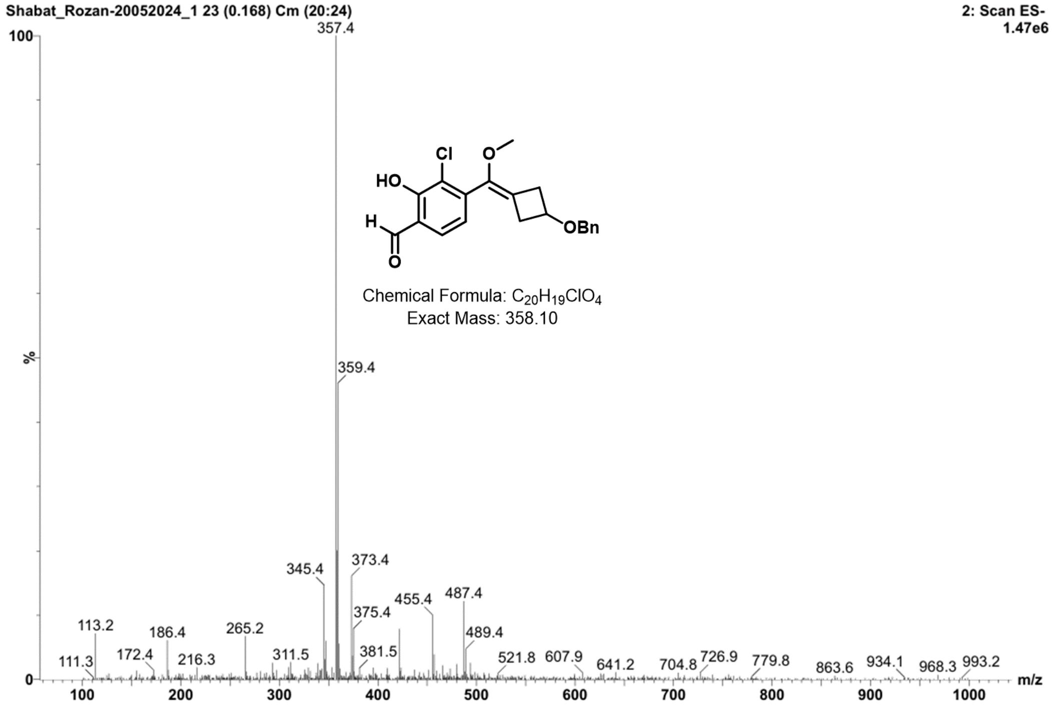

Compound 6c

^{1}H-NMR

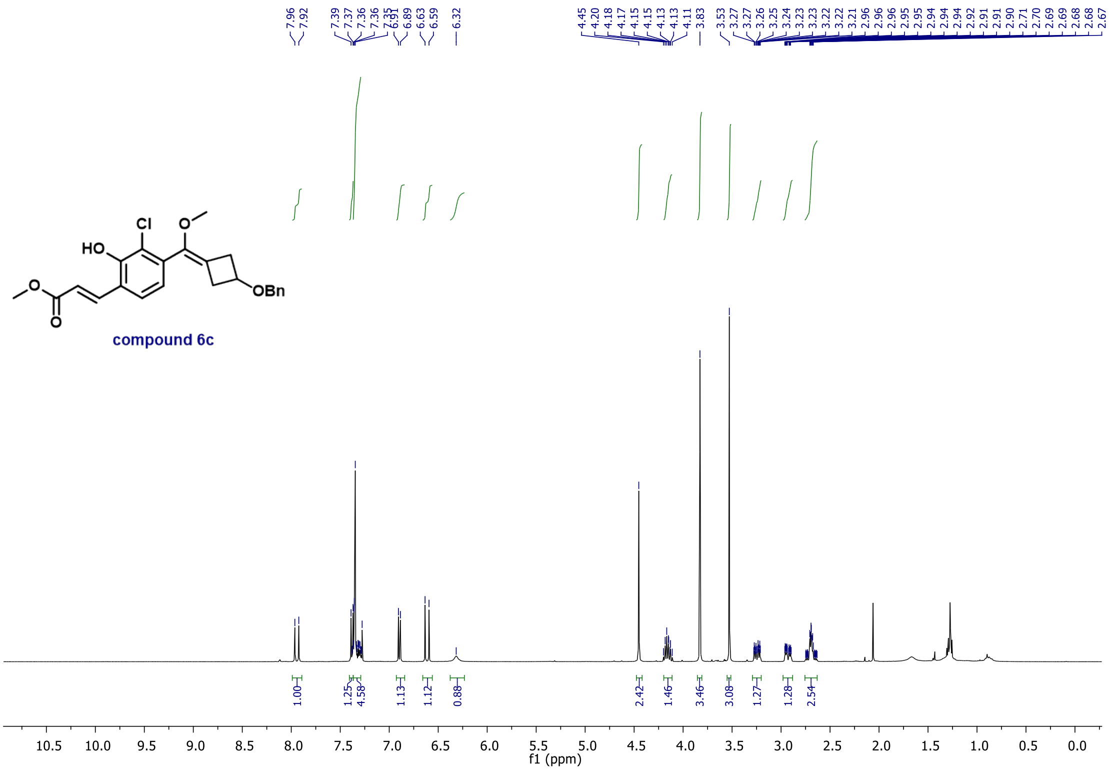

^{13}C-NMR

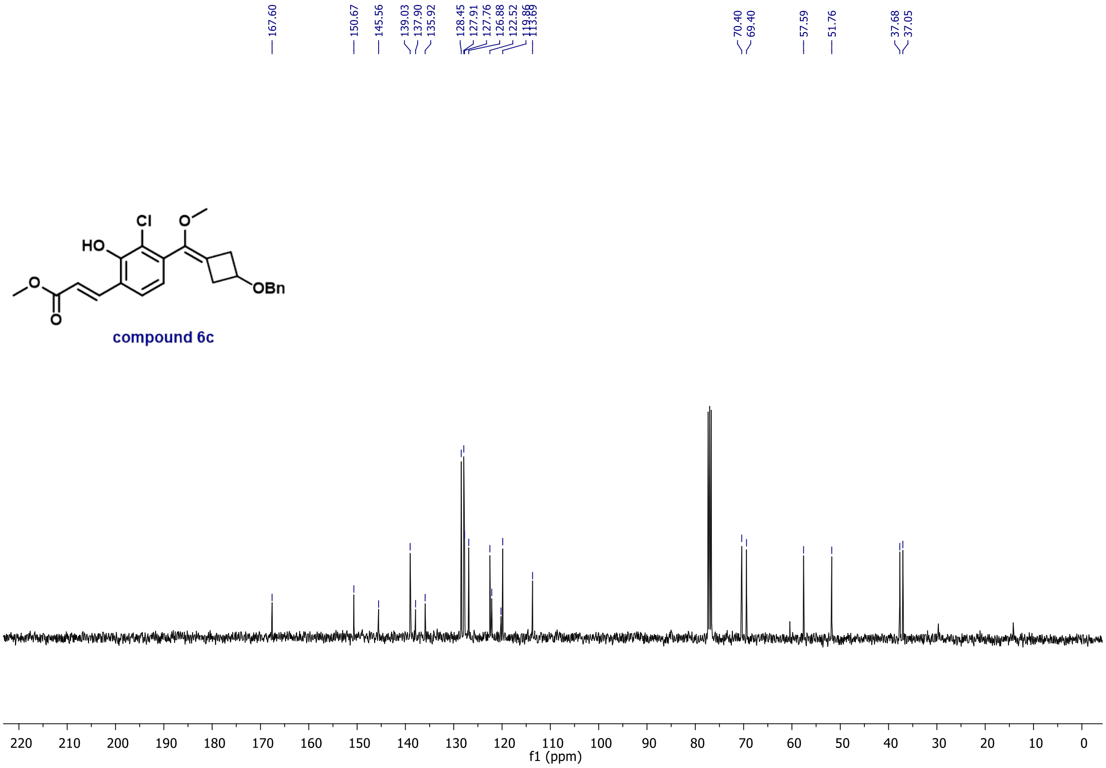

Mass spectra

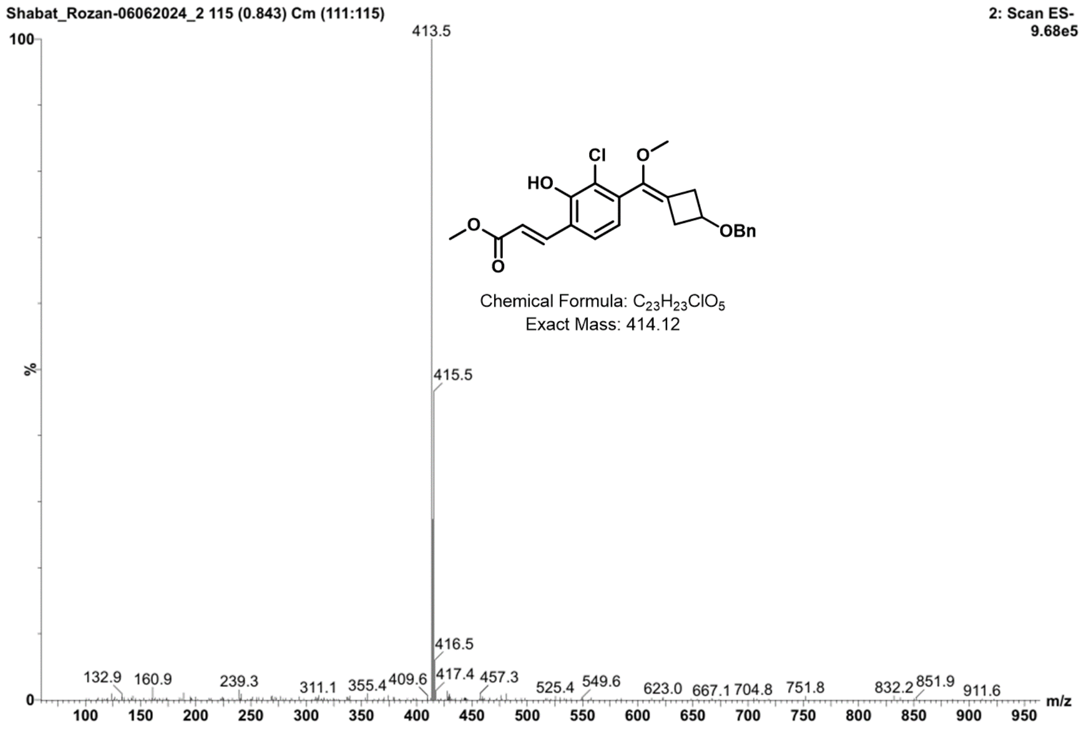

Probe 6 – SOCL-OBn-CB

^{1}H-NMR

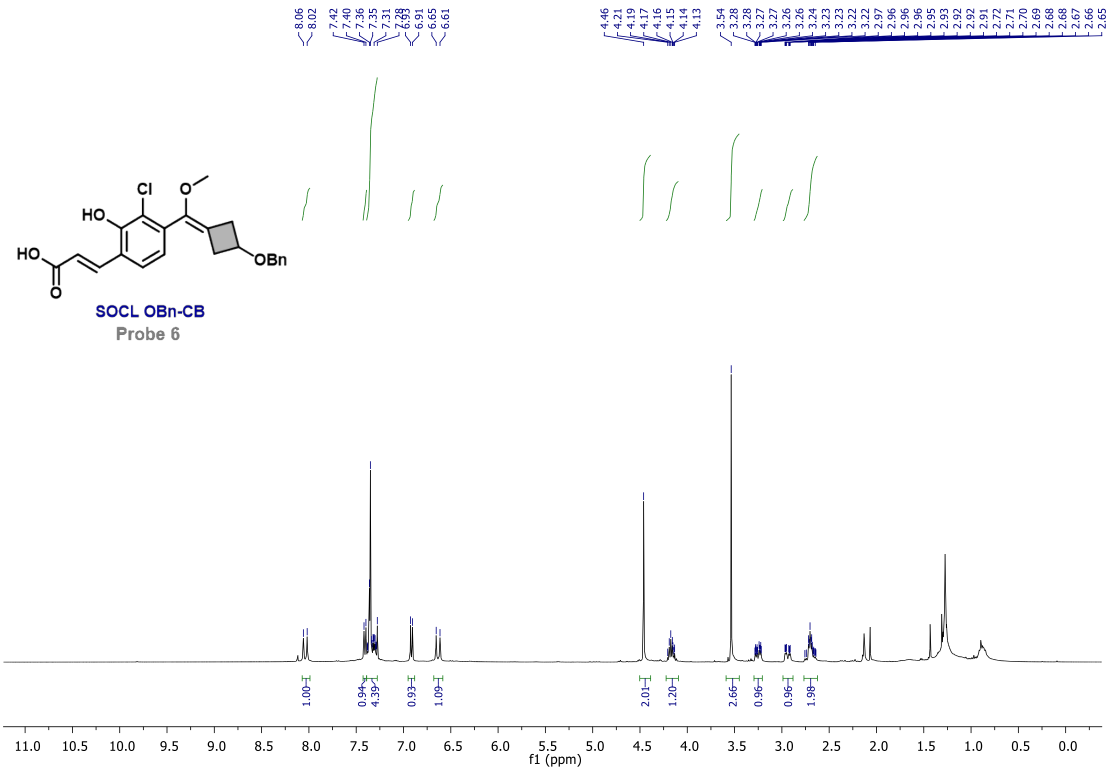

^{13}C-NMR

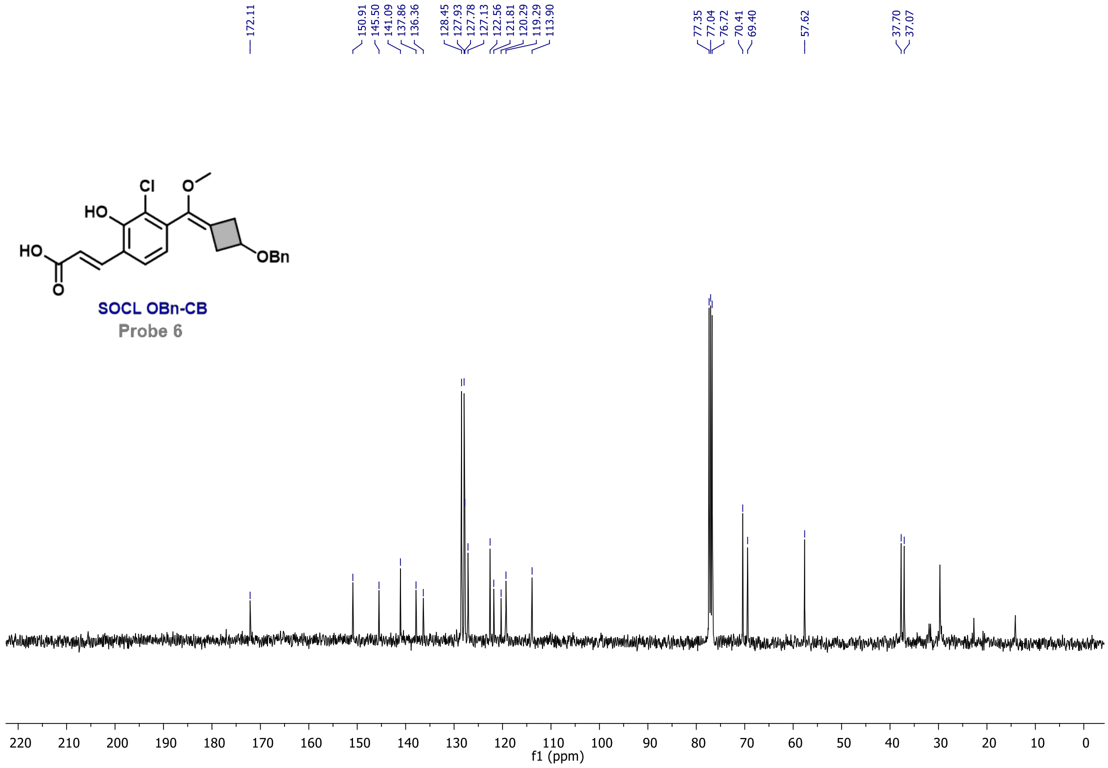

Mass spectra

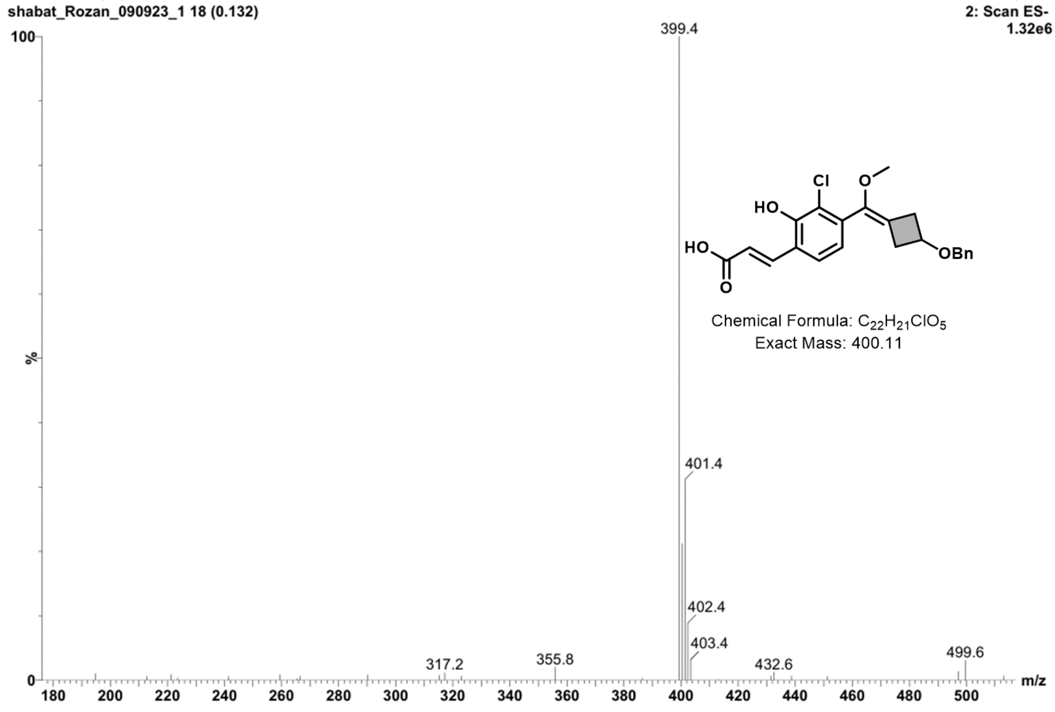

Compound 7b

^{1}H-NMR

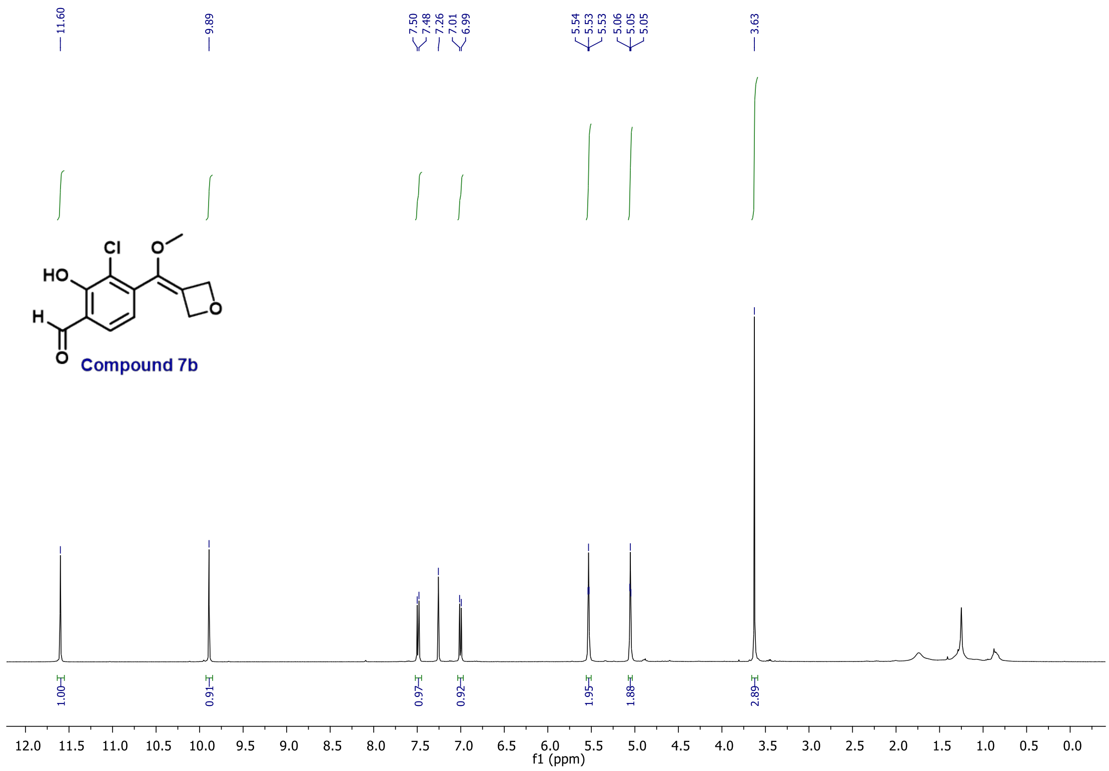

^{13}C-NMR

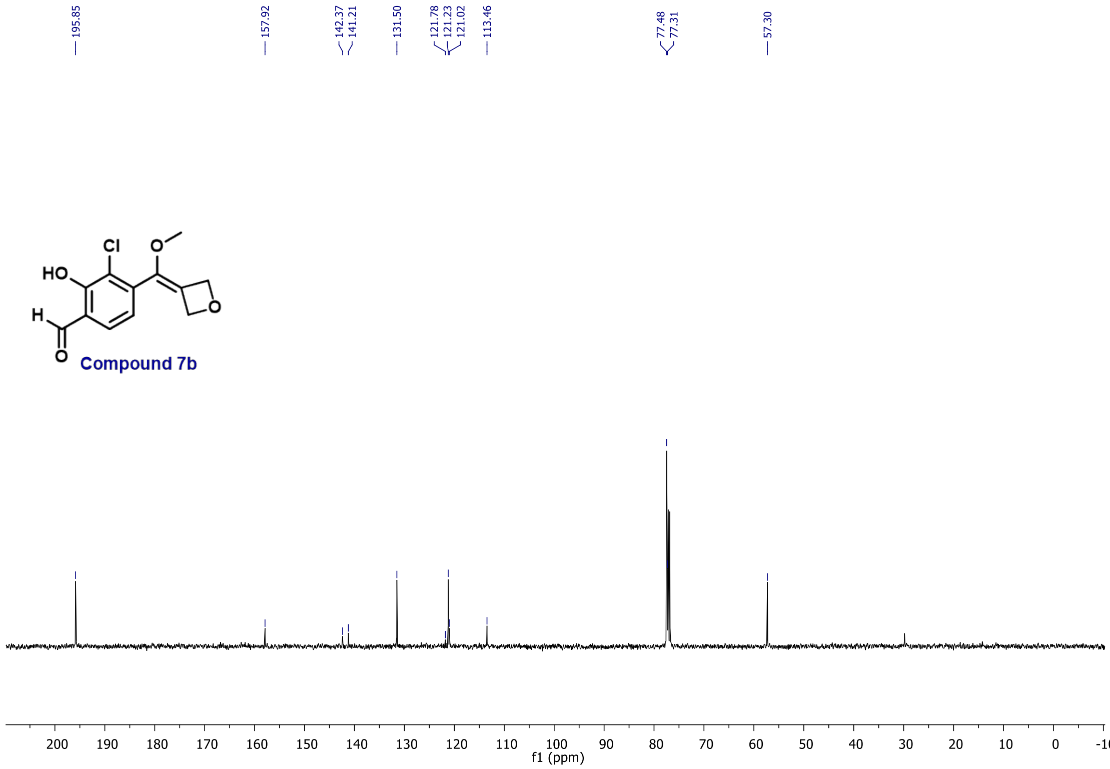

Mass spectra

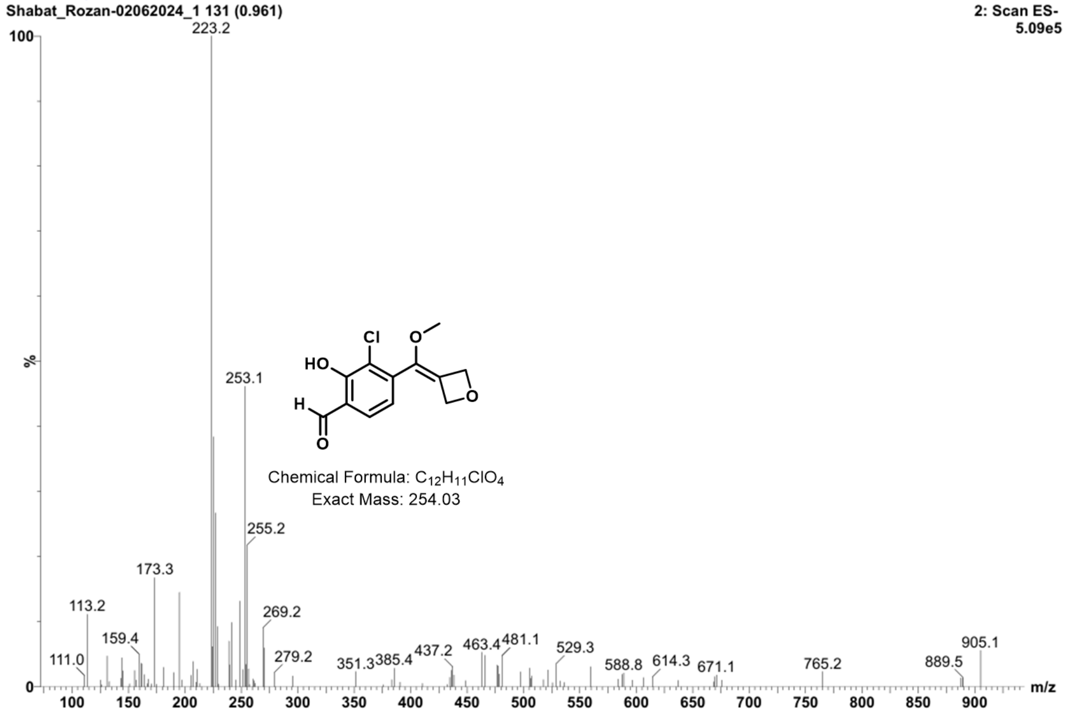

Compound 7c

^{1}H-NMR

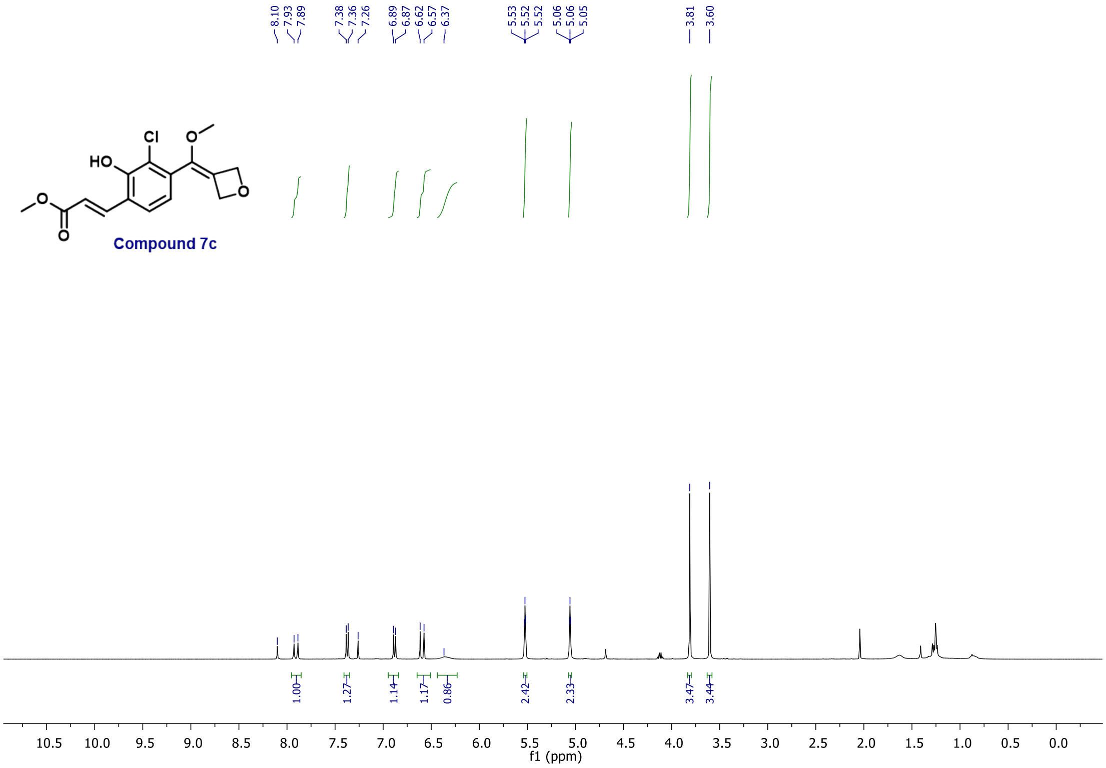

^{13}C-NMR

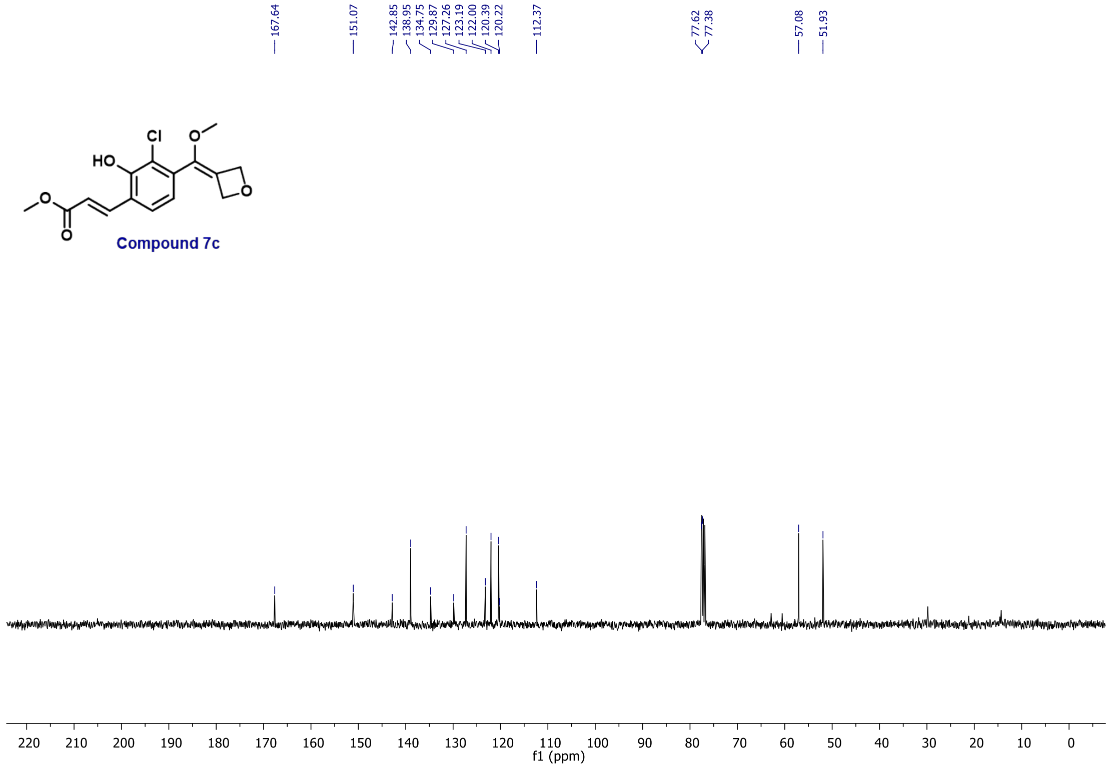

Mass spectra

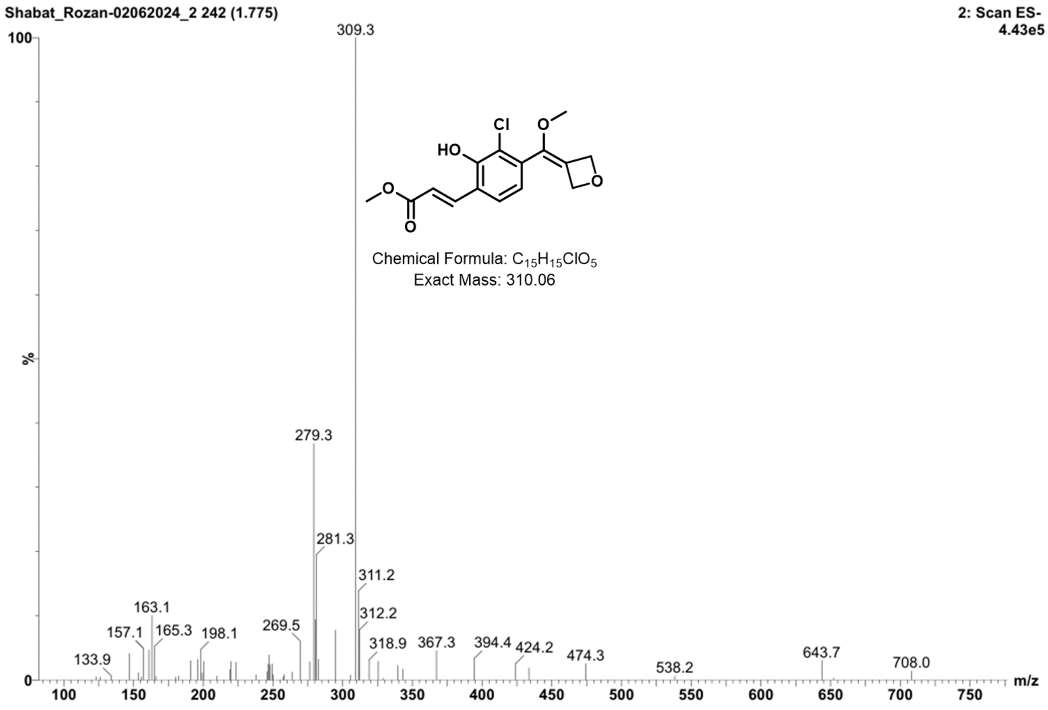

Probe 7 – SOCL-Ox

^{1}H-NMR

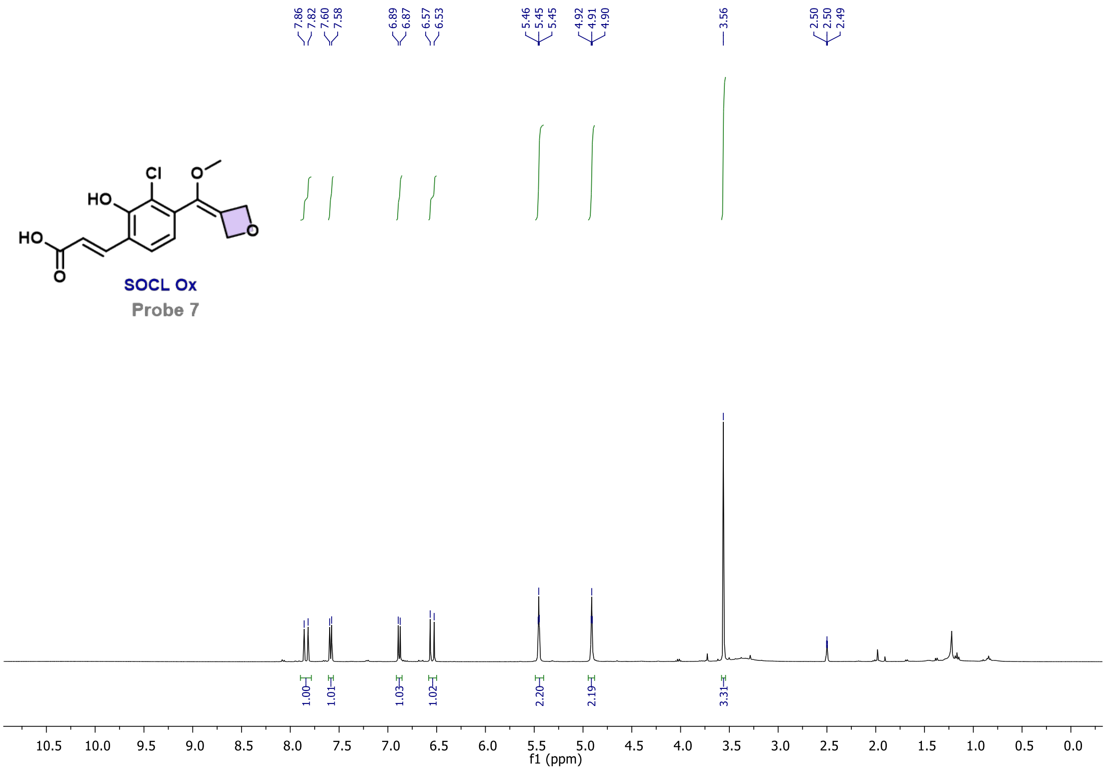

^{13}C-NMR

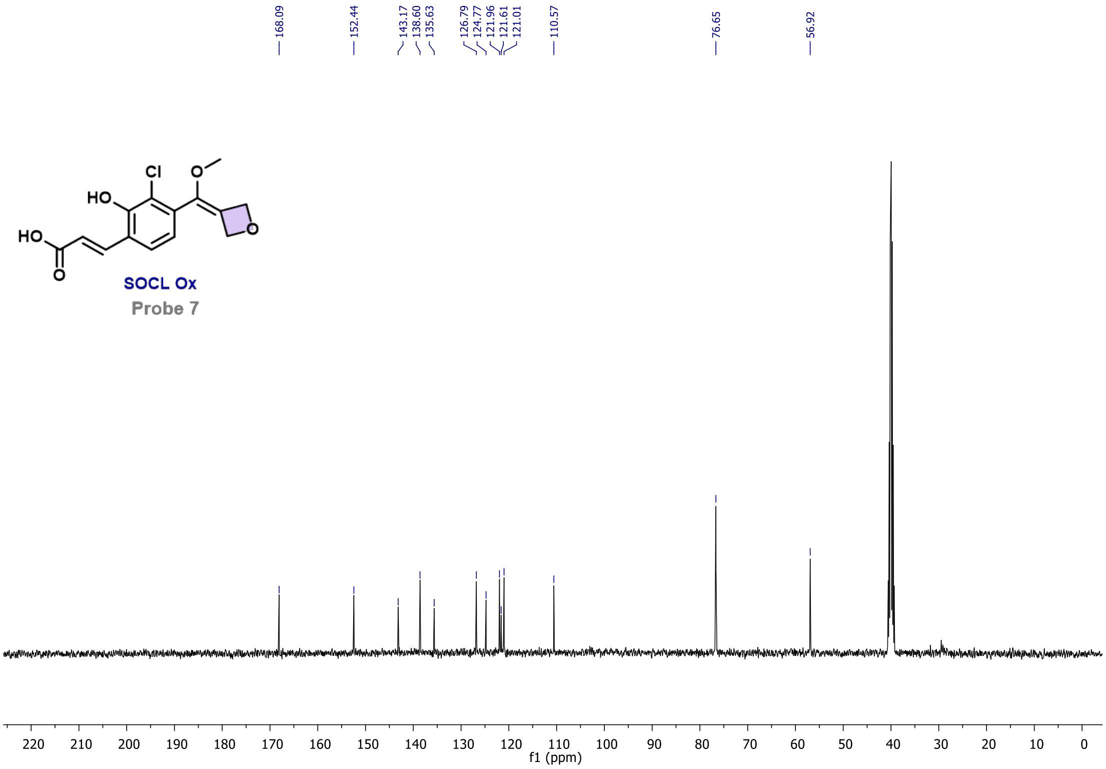

Mass spectra

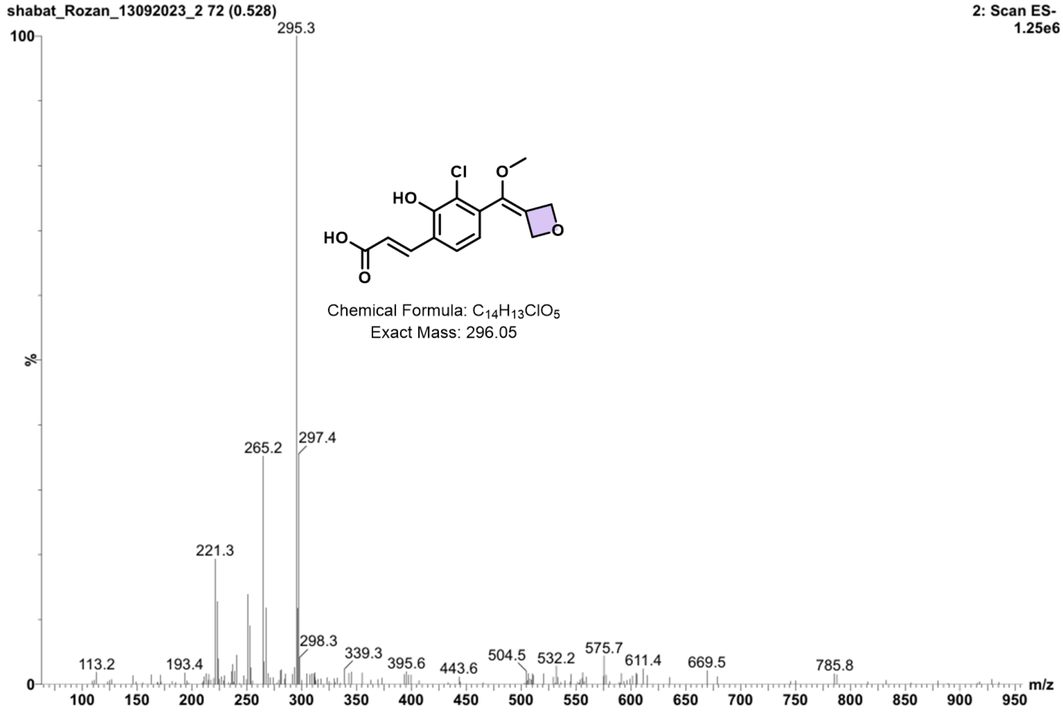

Compound 8a

^{1}H-NMR

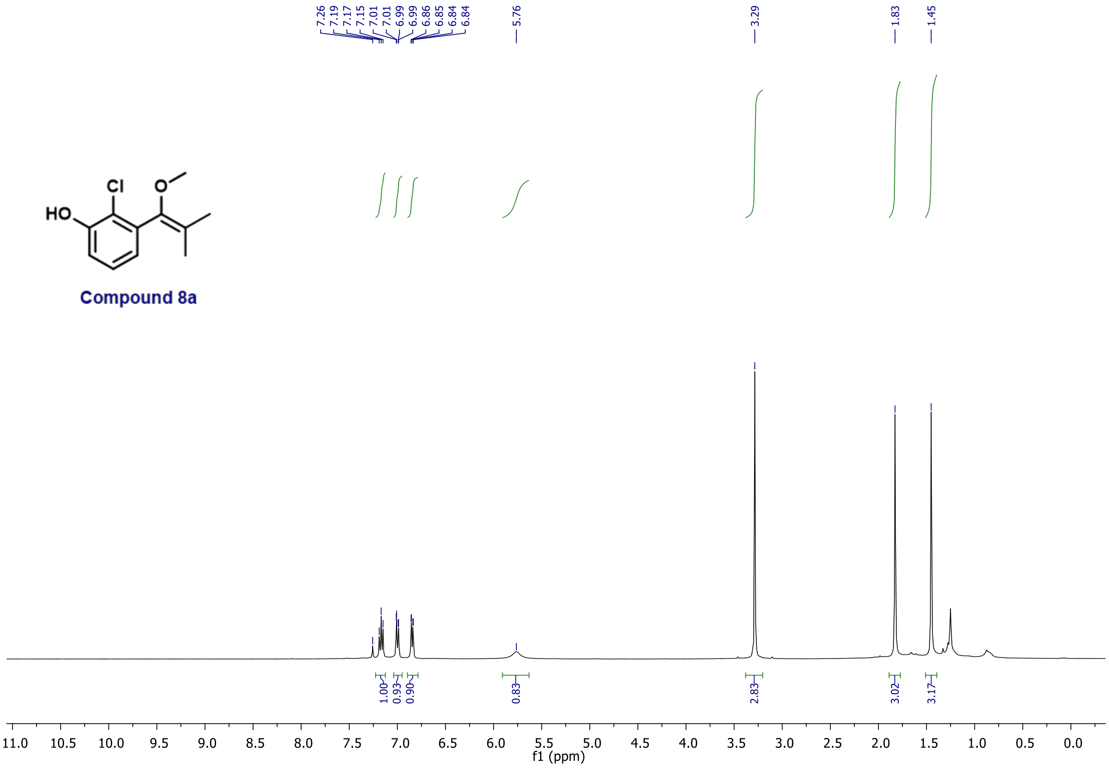

^{13}C-NMR

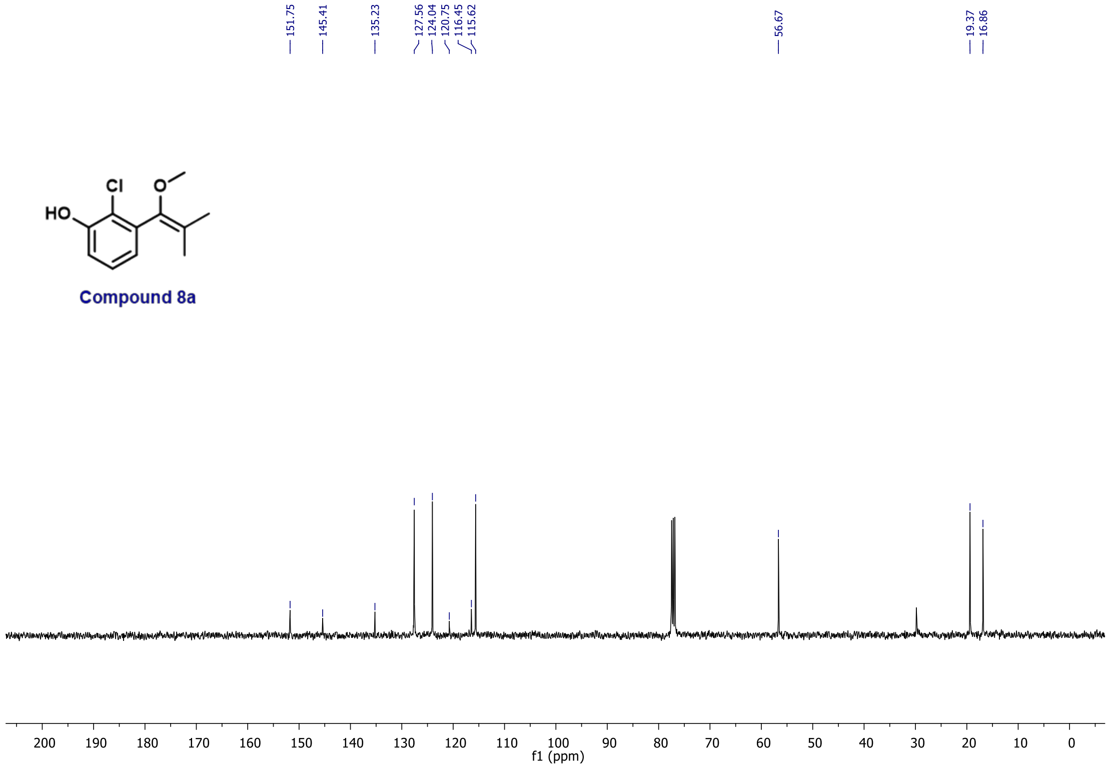

Mass spectra

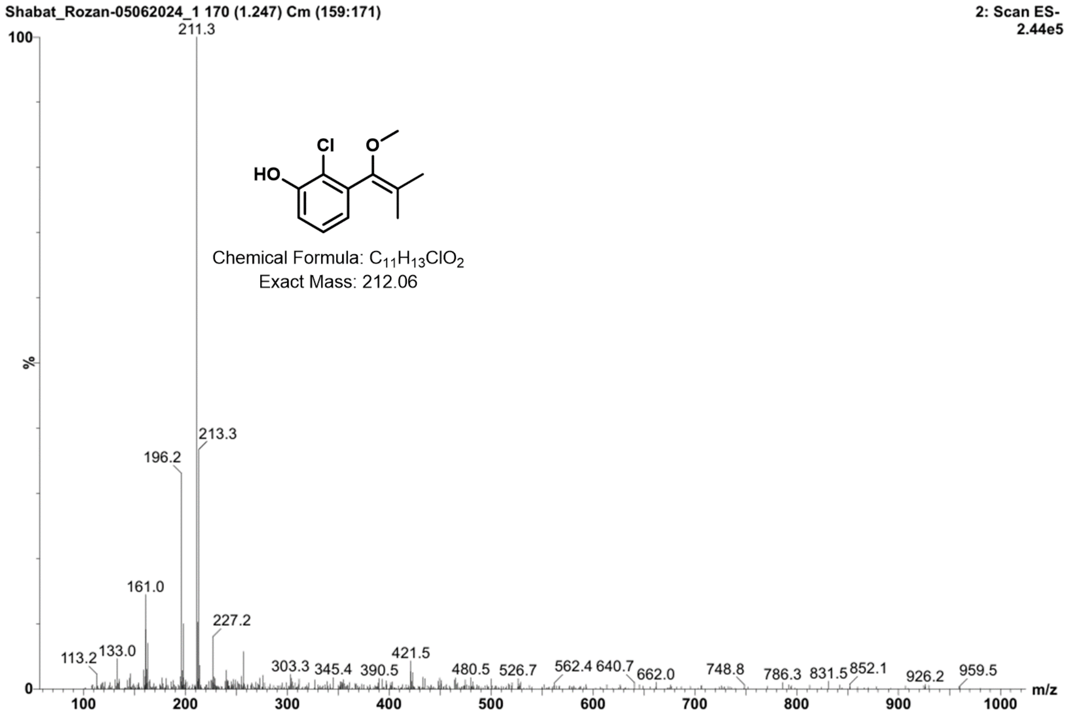

Compound 8b

^{1}H-NMR

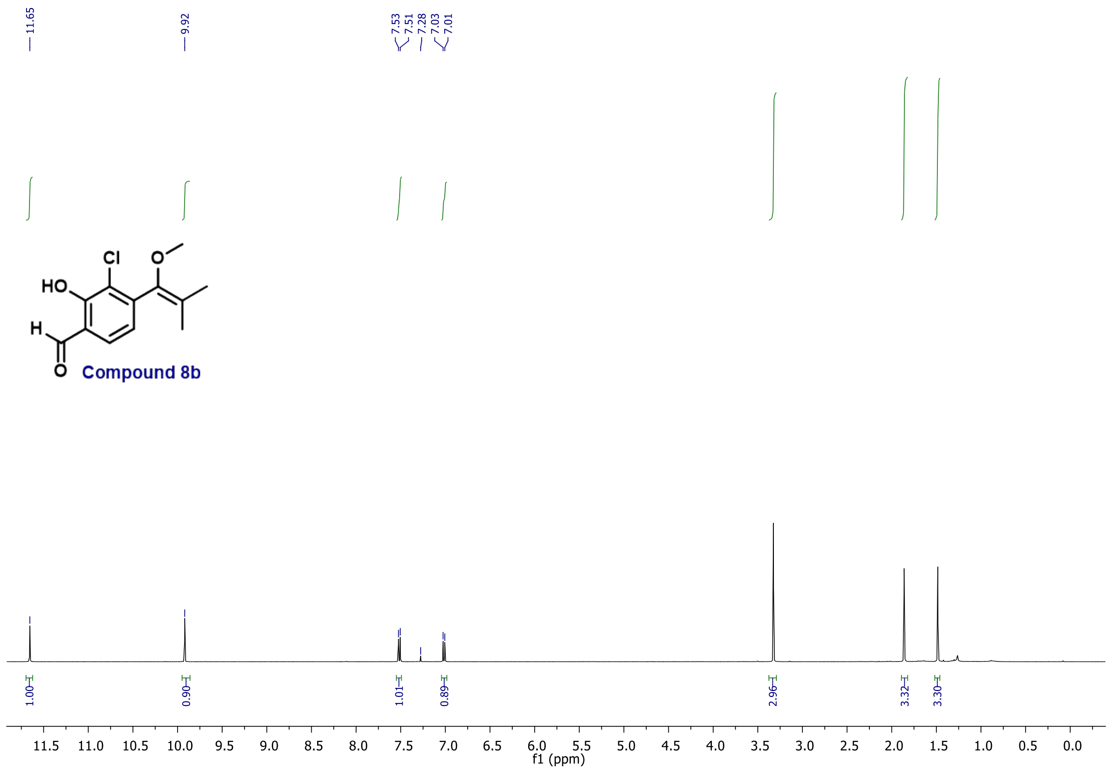

^{13}C-NMR

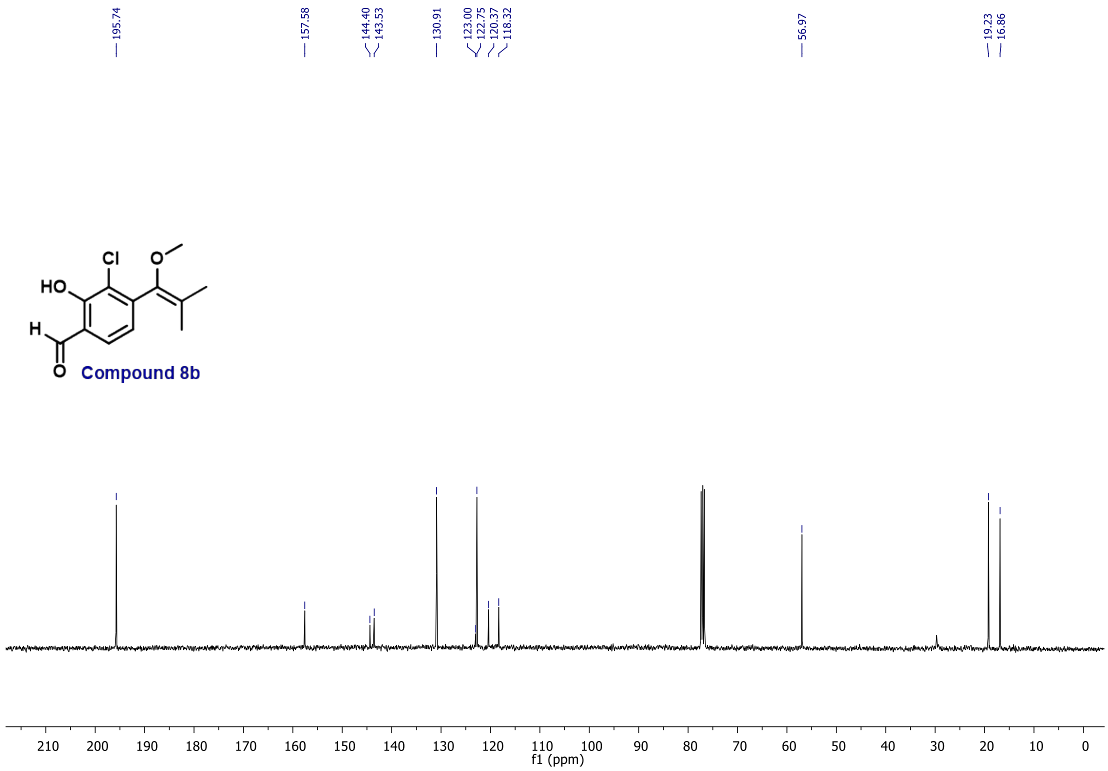

Mass spectra

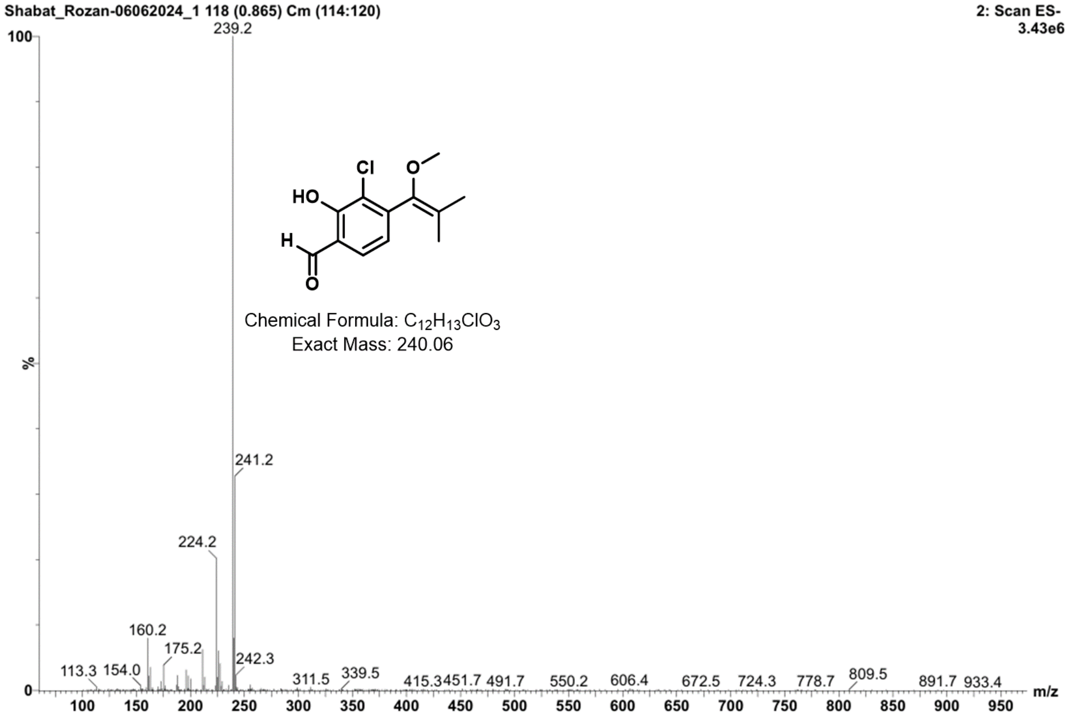

Compound 8c

^{1}H-NMR

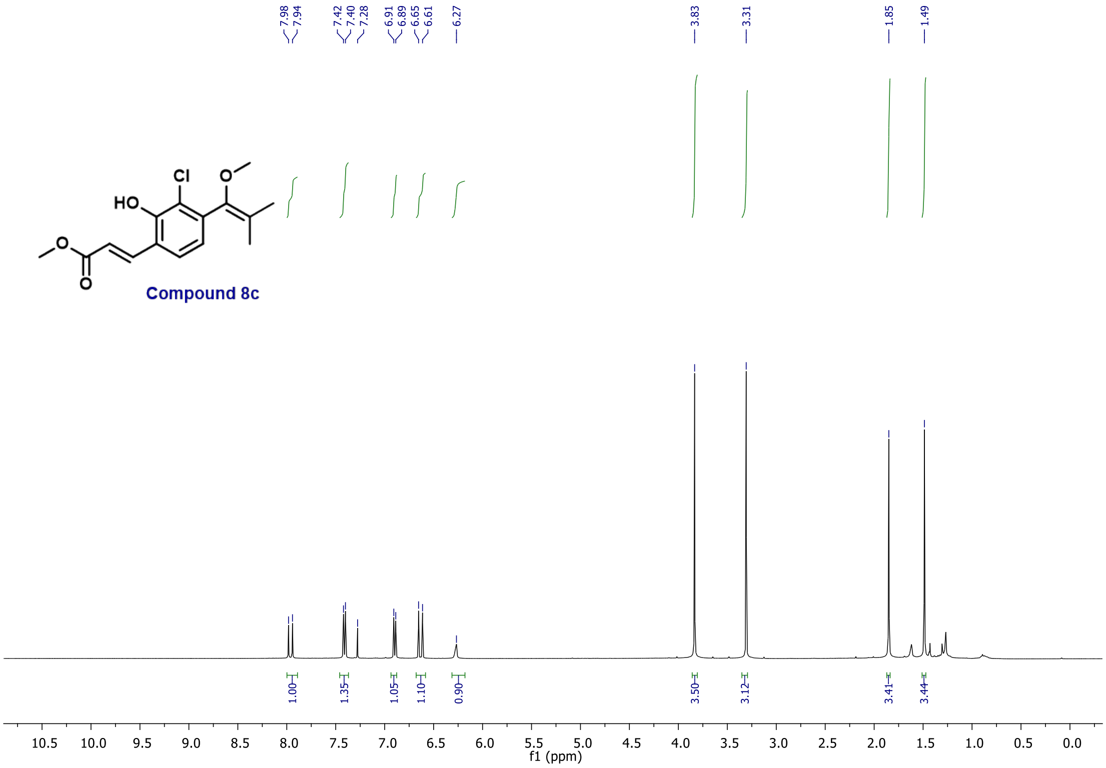

^{13}C-NMR

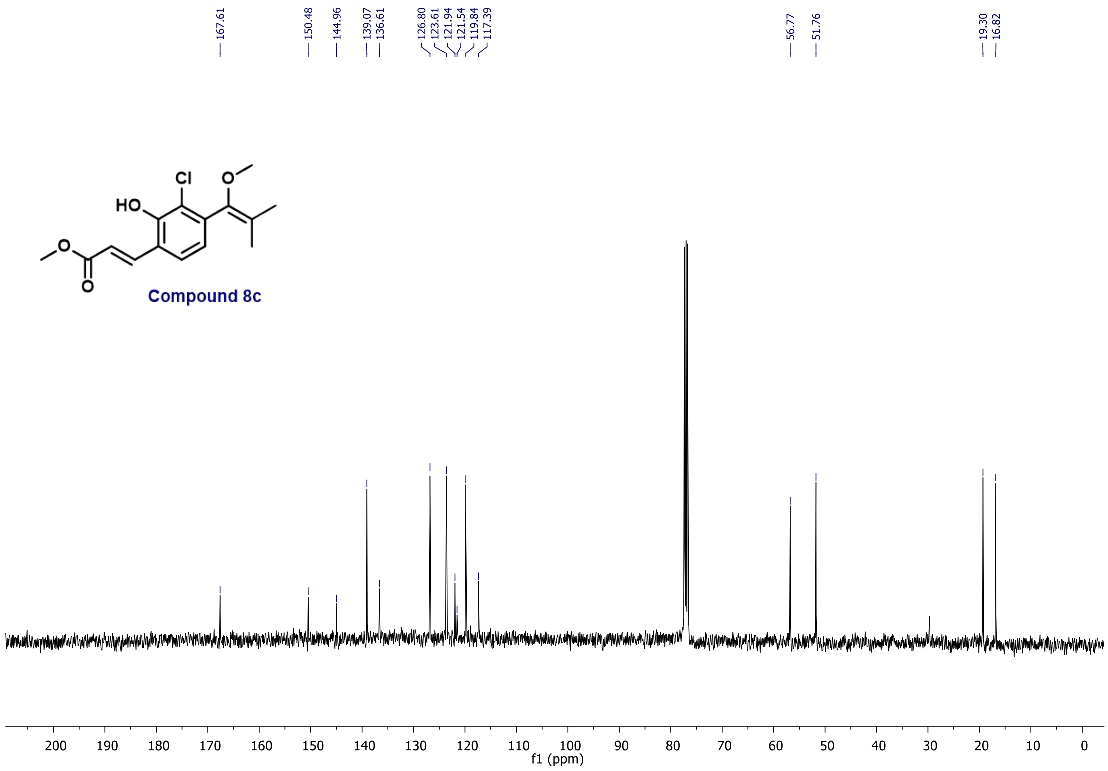

Mass spectra

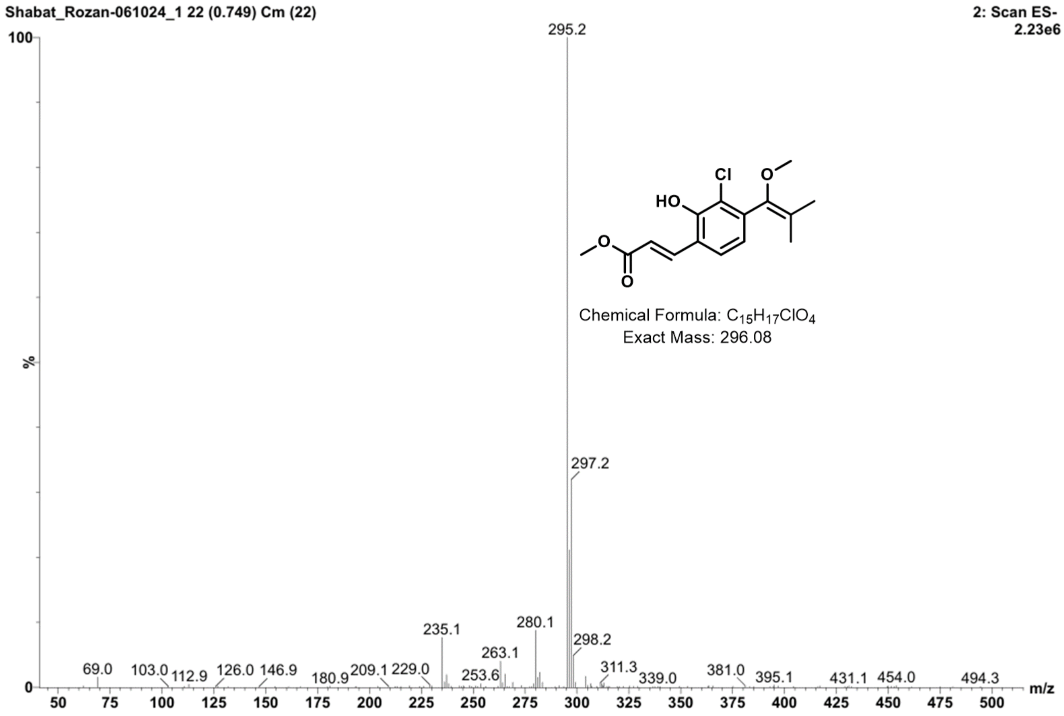

Probe 8 – SOCL-DM

^{1}H-NMR

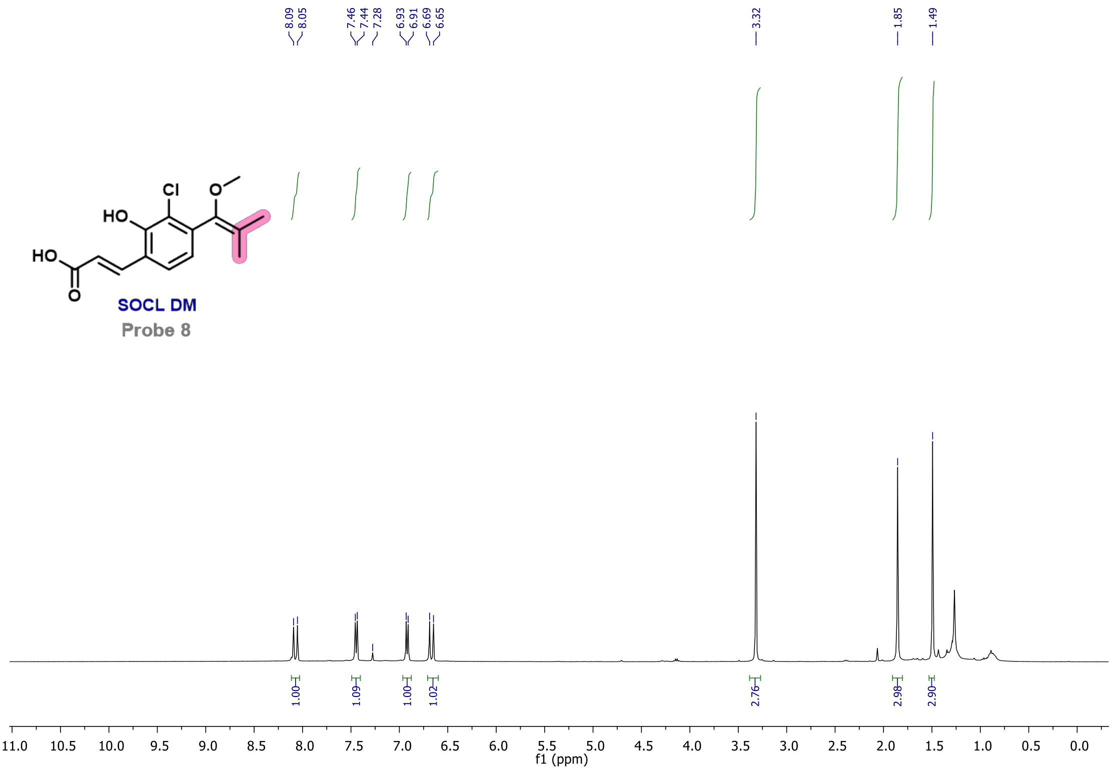

^{13}C-NMR

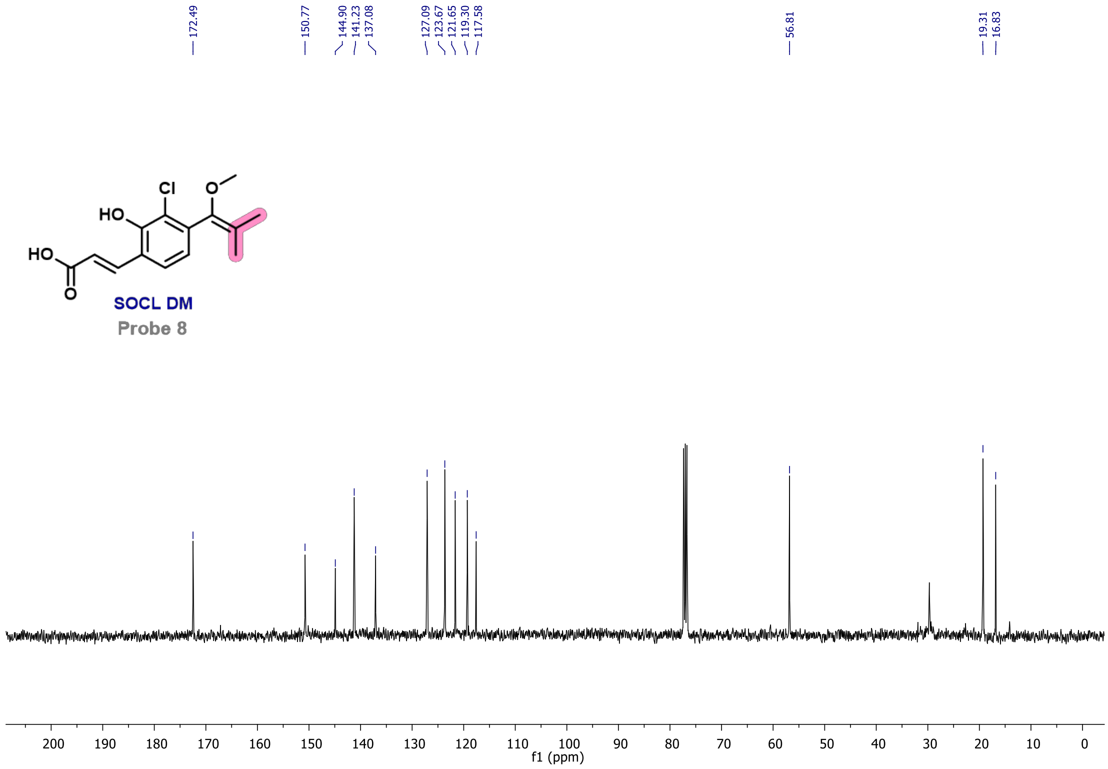

Mass spectra

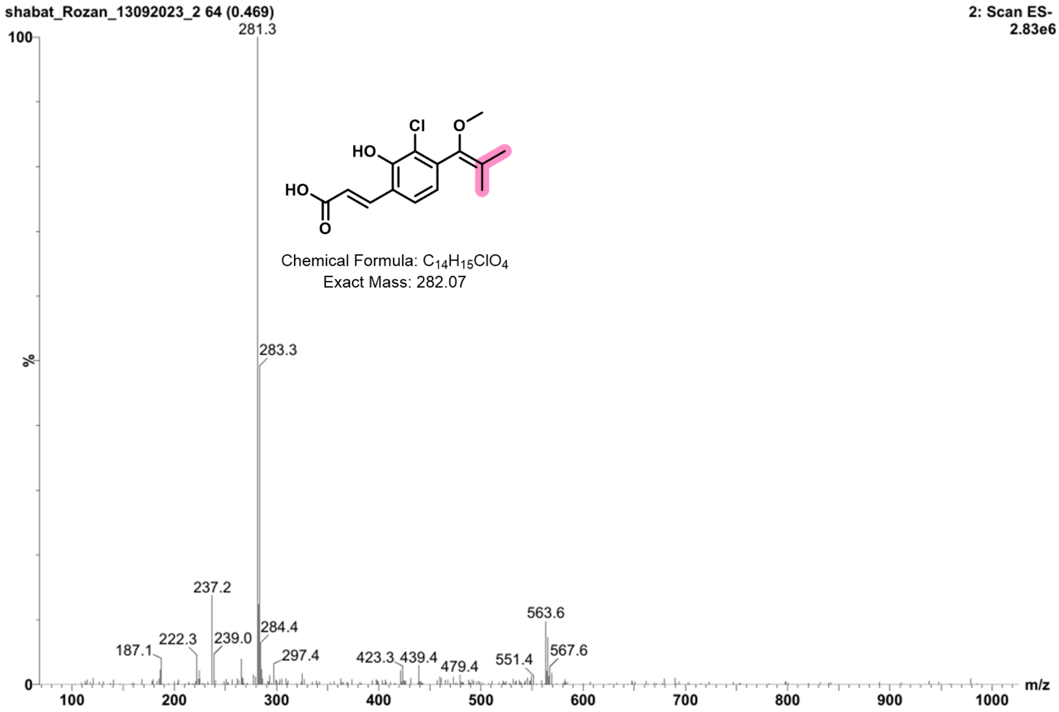

Compound 9a

^{1}H-NMR

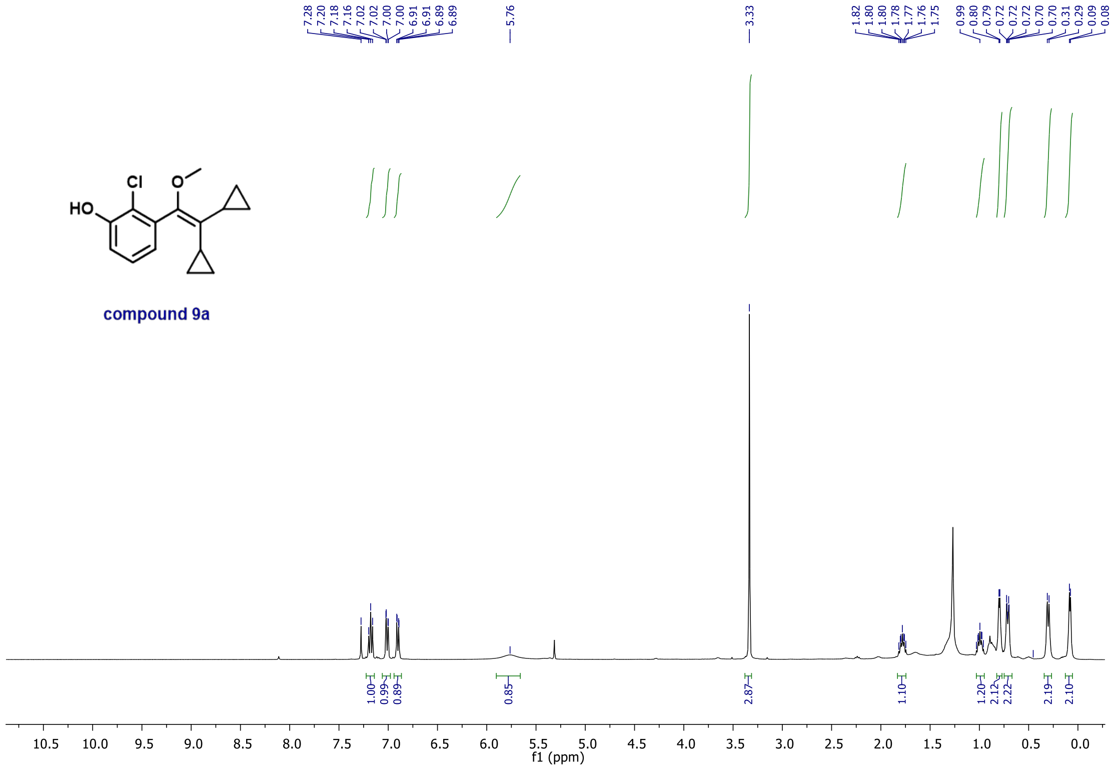

^{13}C-NMR

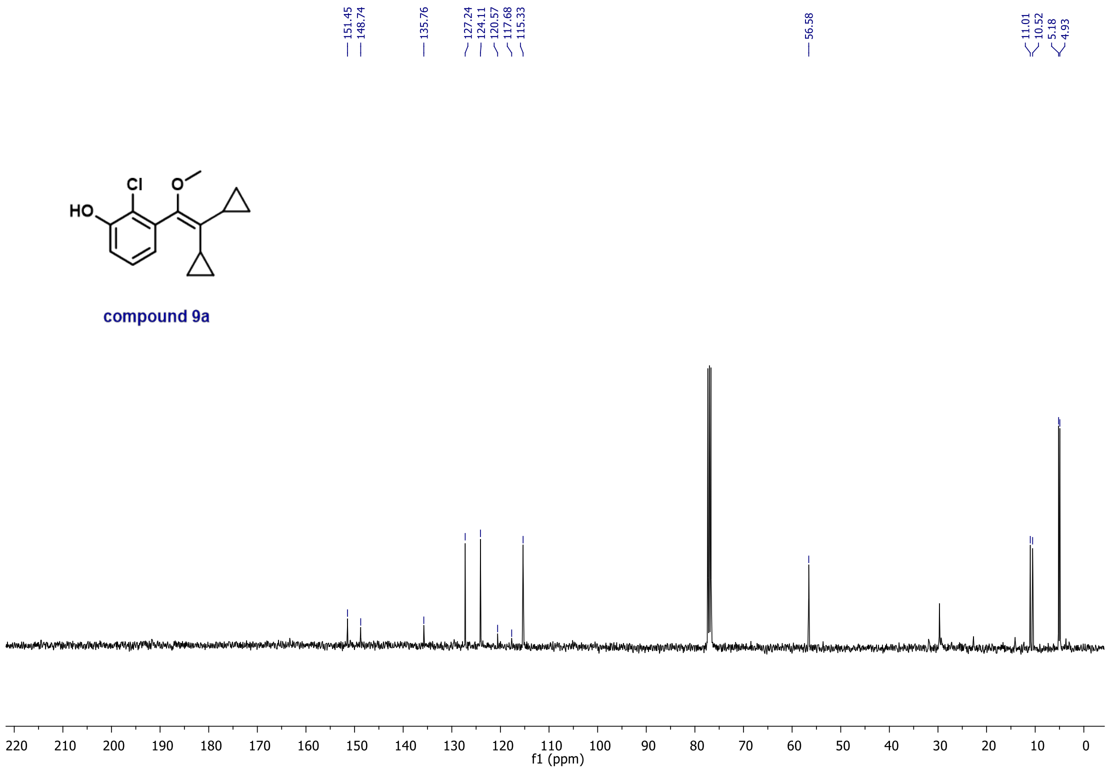

Mass spectra

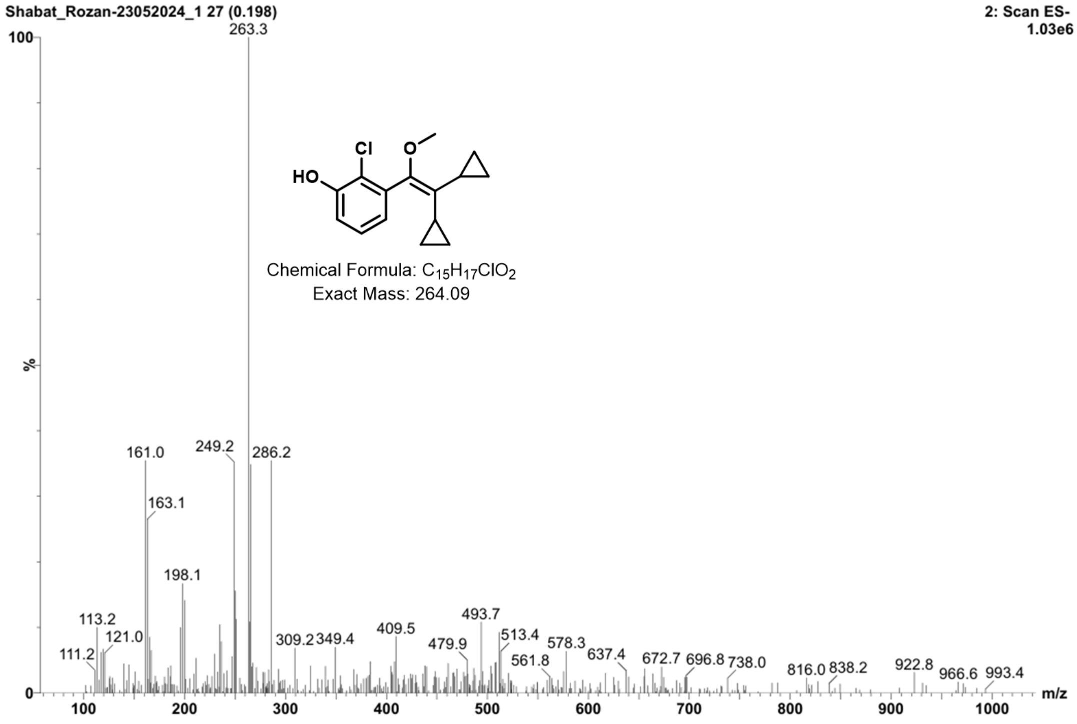

Compound 9b

^{1}H-NMR

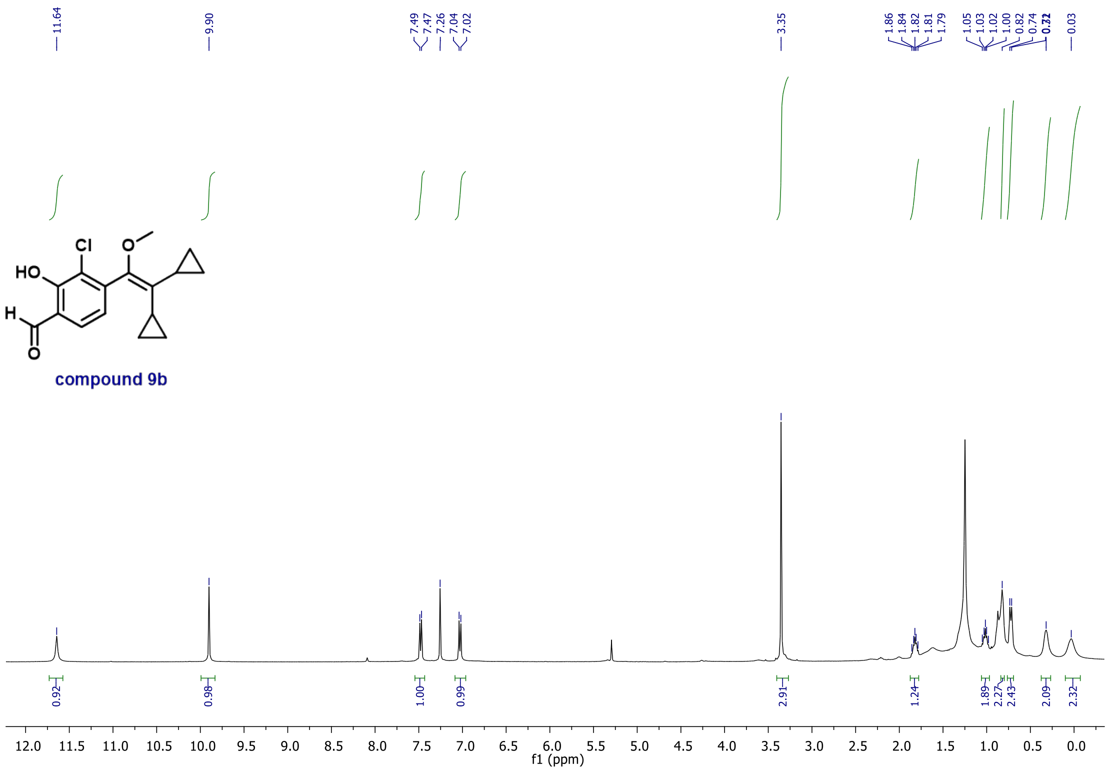

^{13}C-NMR

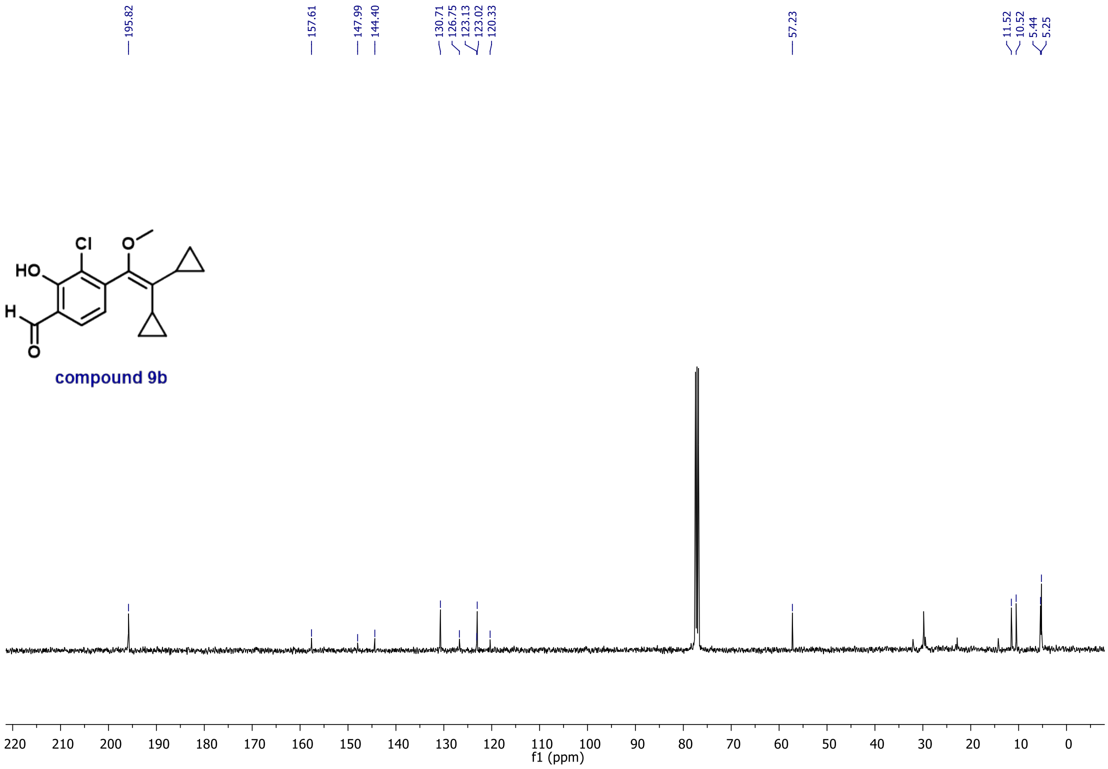

Mass spectra

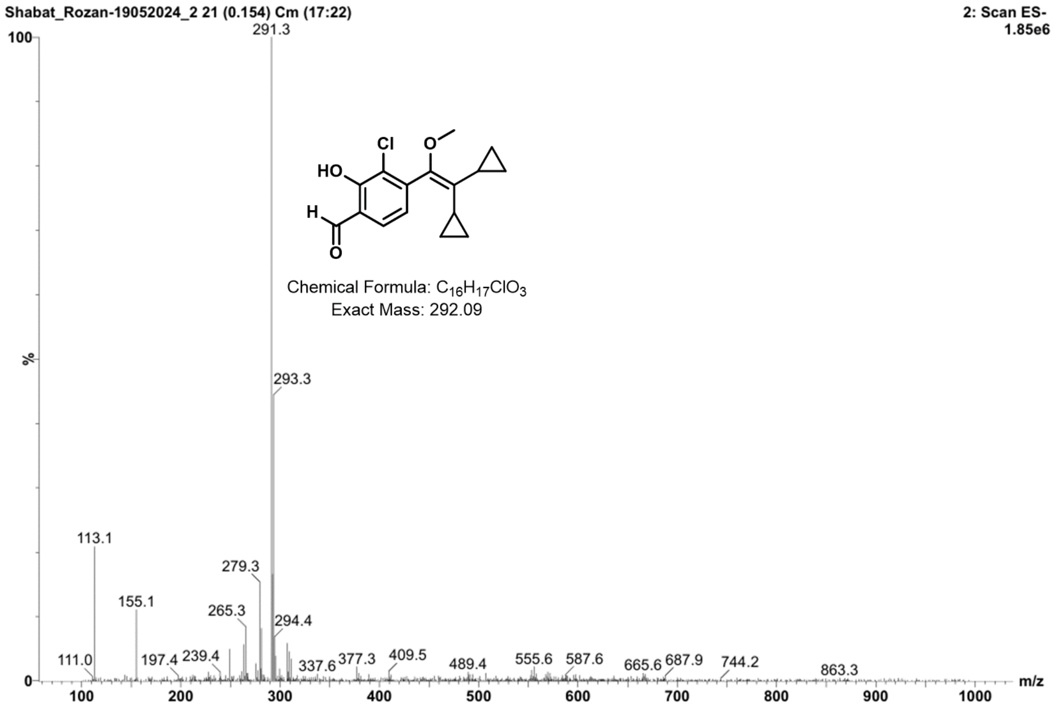

Compound 9c

^{1}H-NMR

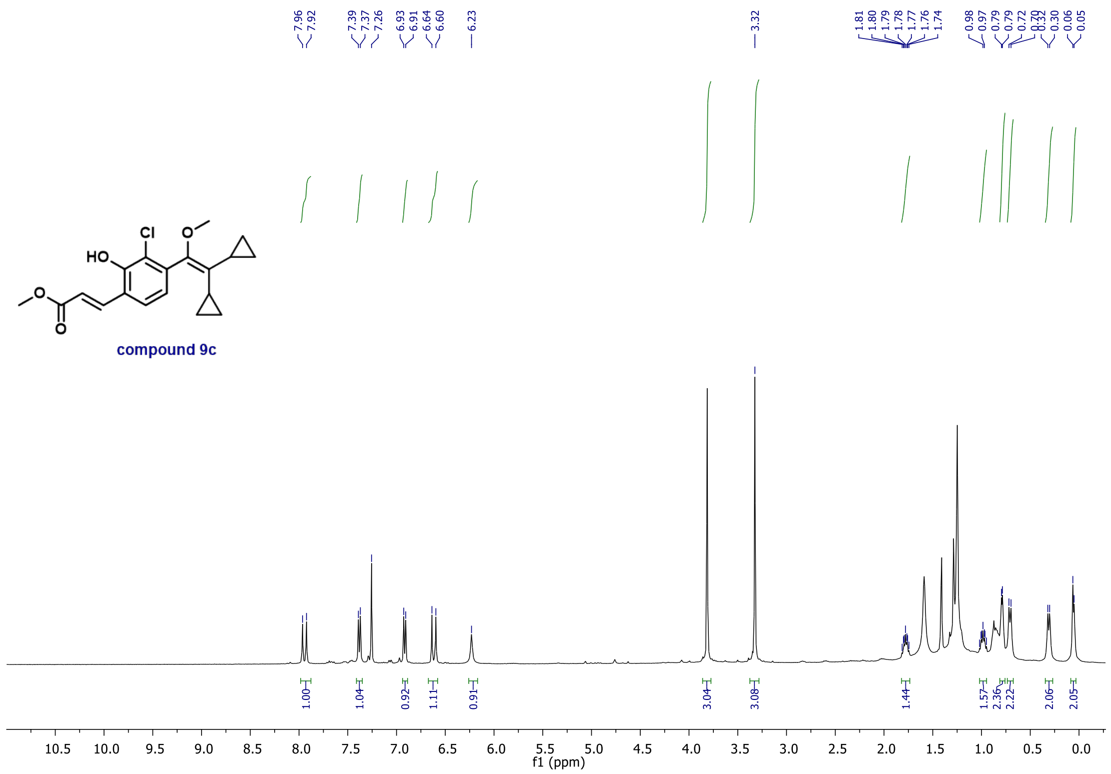

^{13}C-NMR

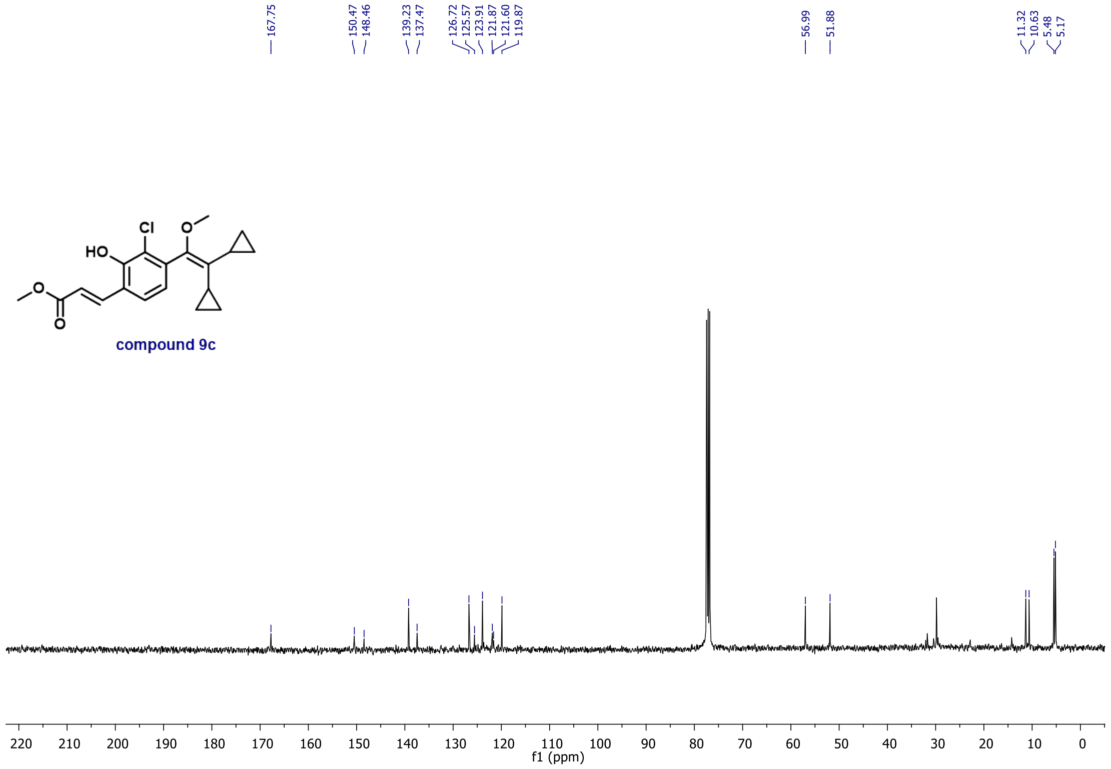

Mass spectra

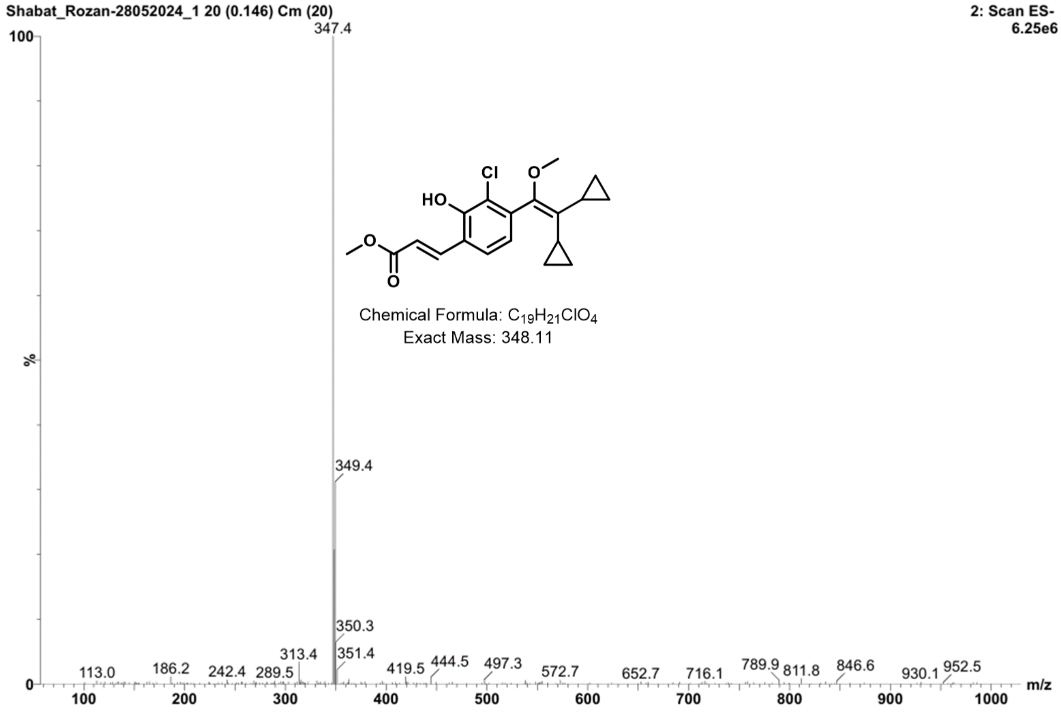

Probe 9 – SOCL-DCP

^{1}H-NMR

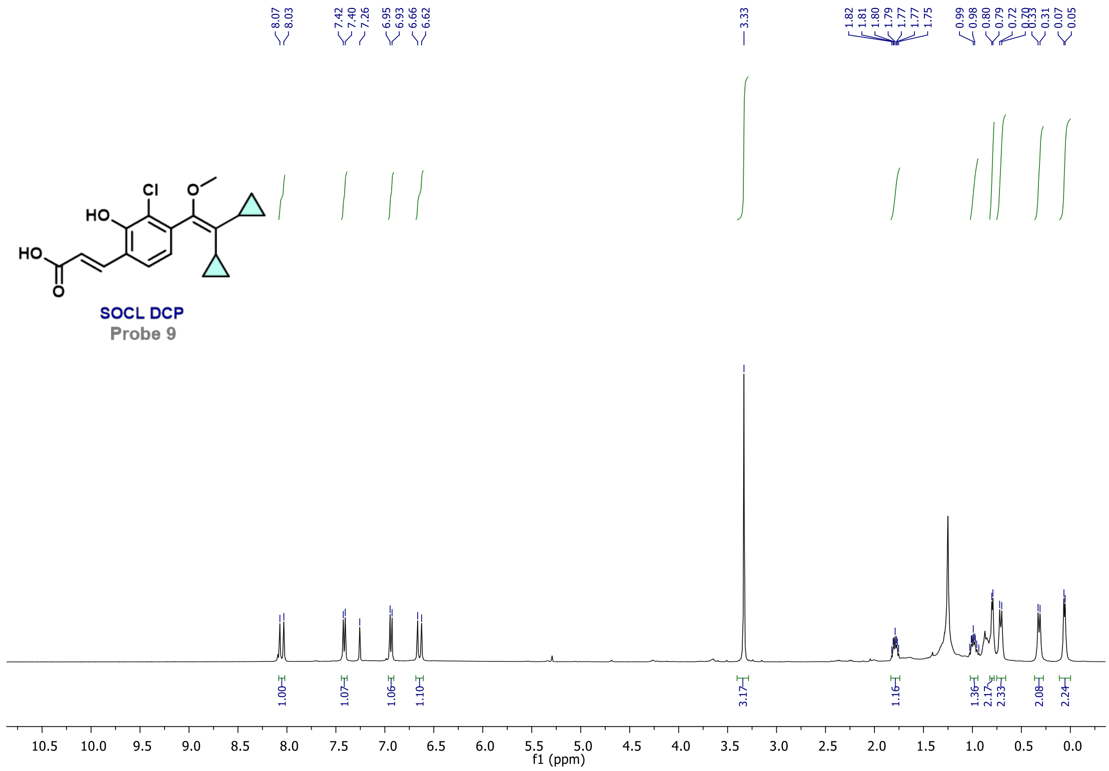

^{13}C-NMR

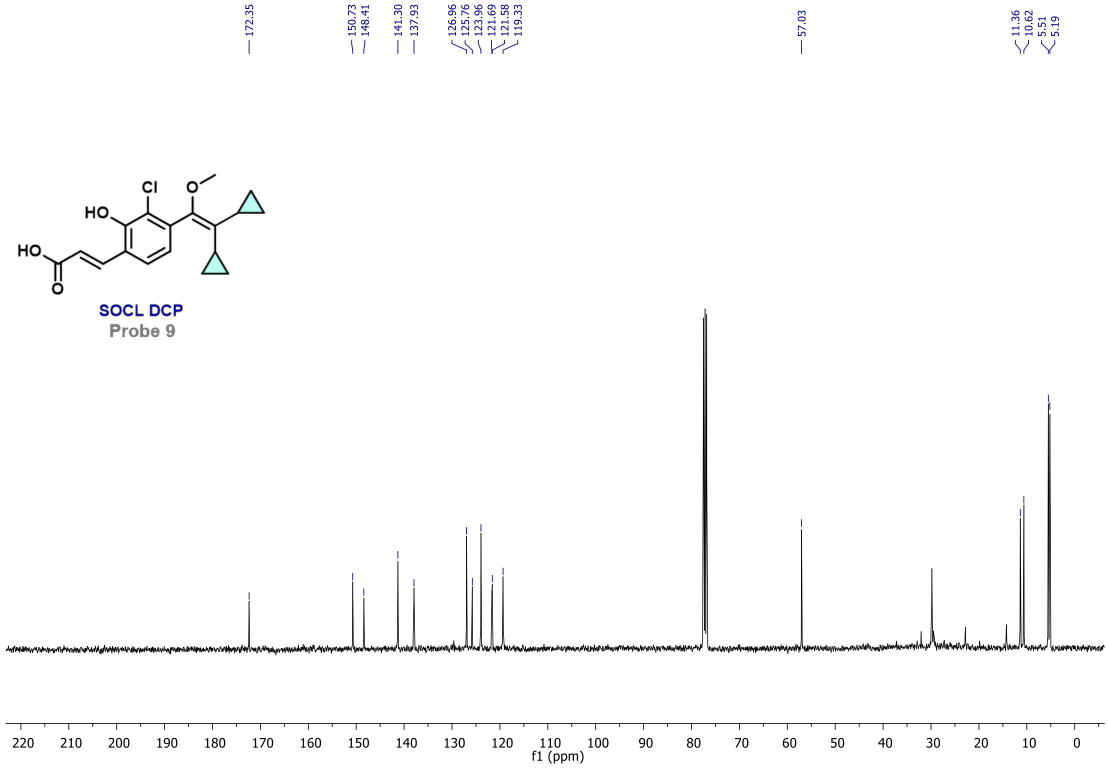

Mass spectra

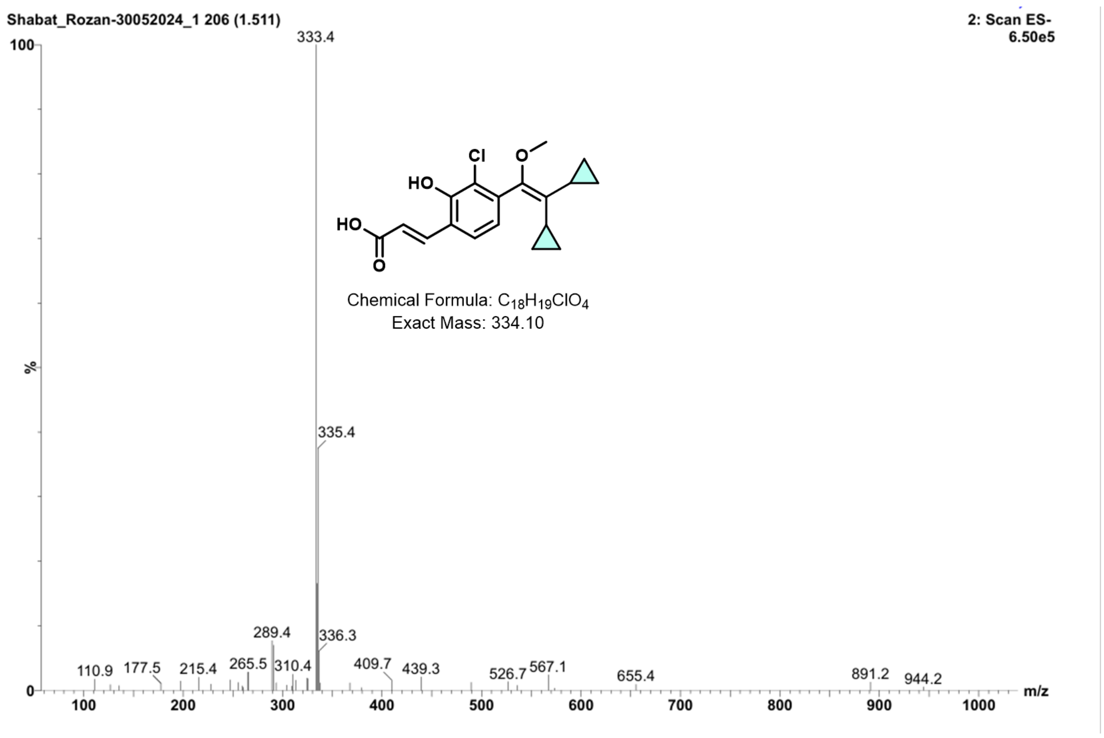

Compound 10a

^{1}H-NMR

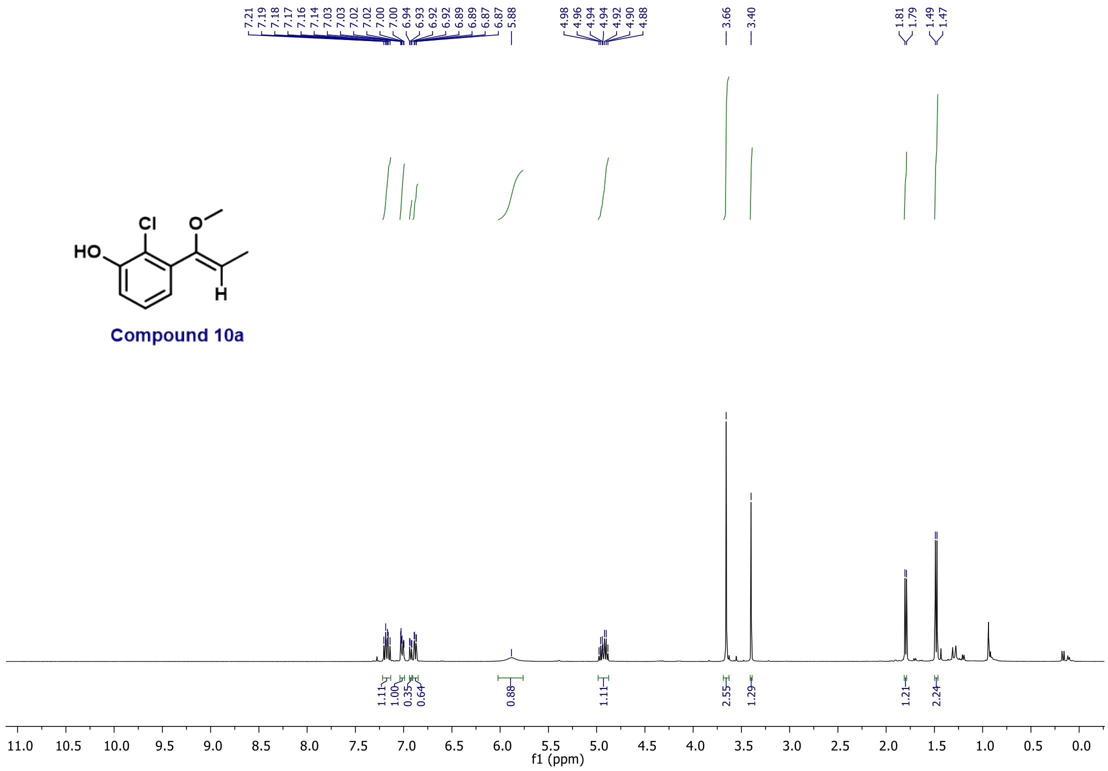

^{13}C-NMR

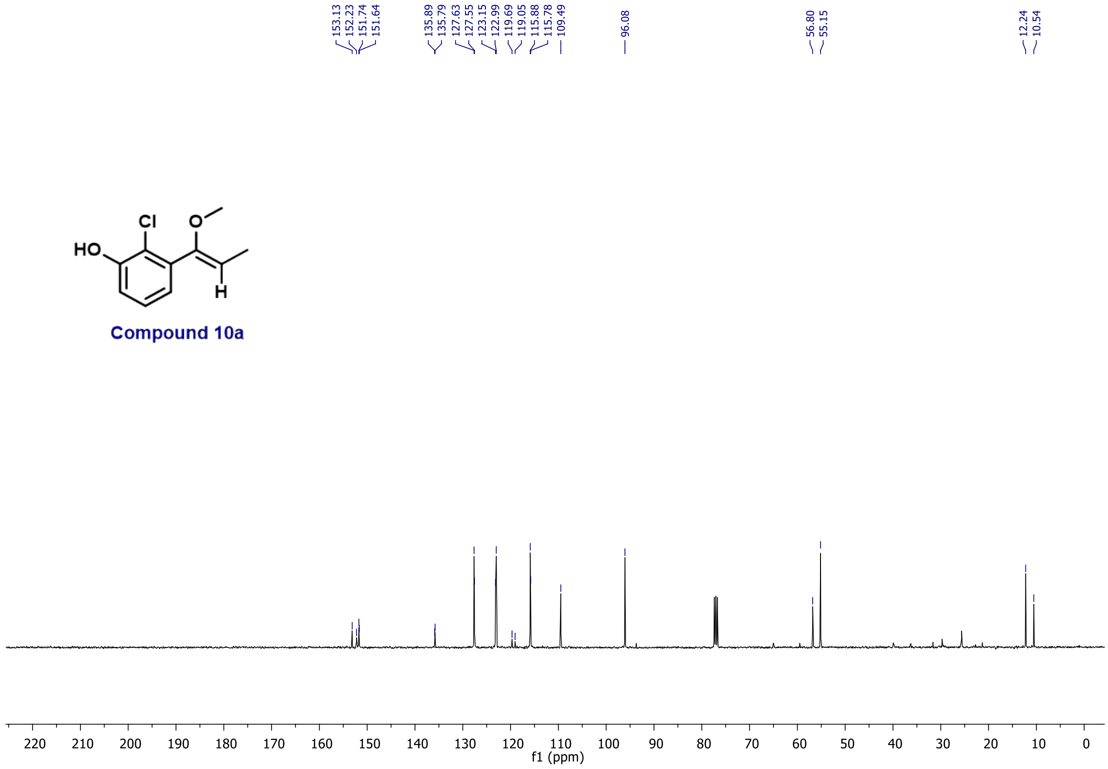

Mass spectra

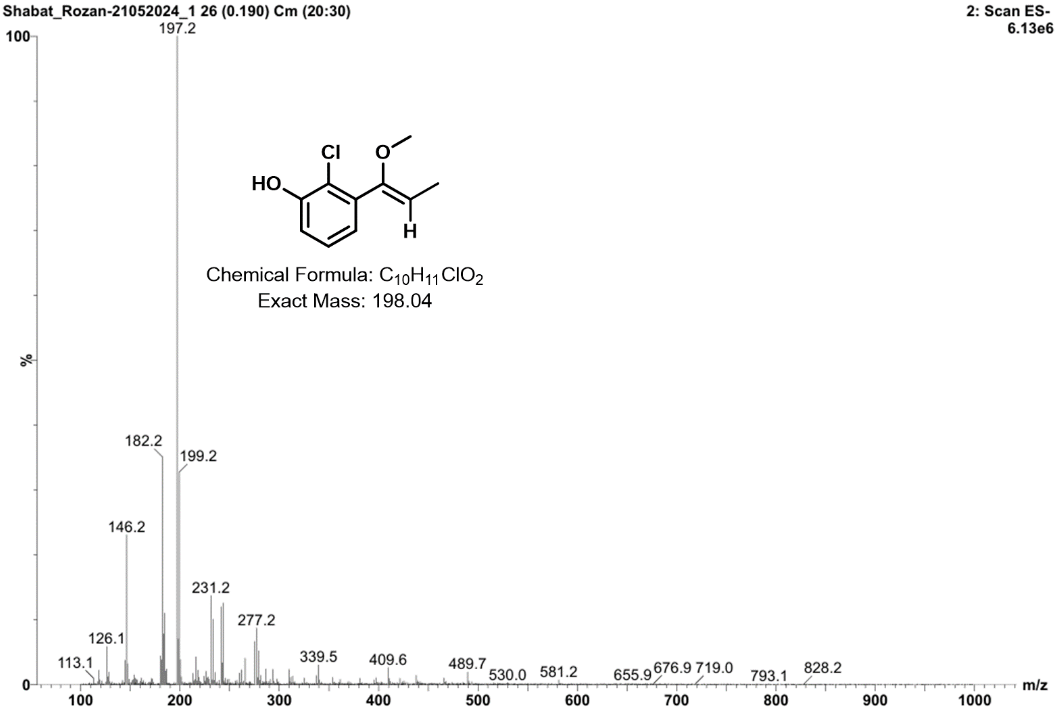

Compound 10b

^{1}H-NMR

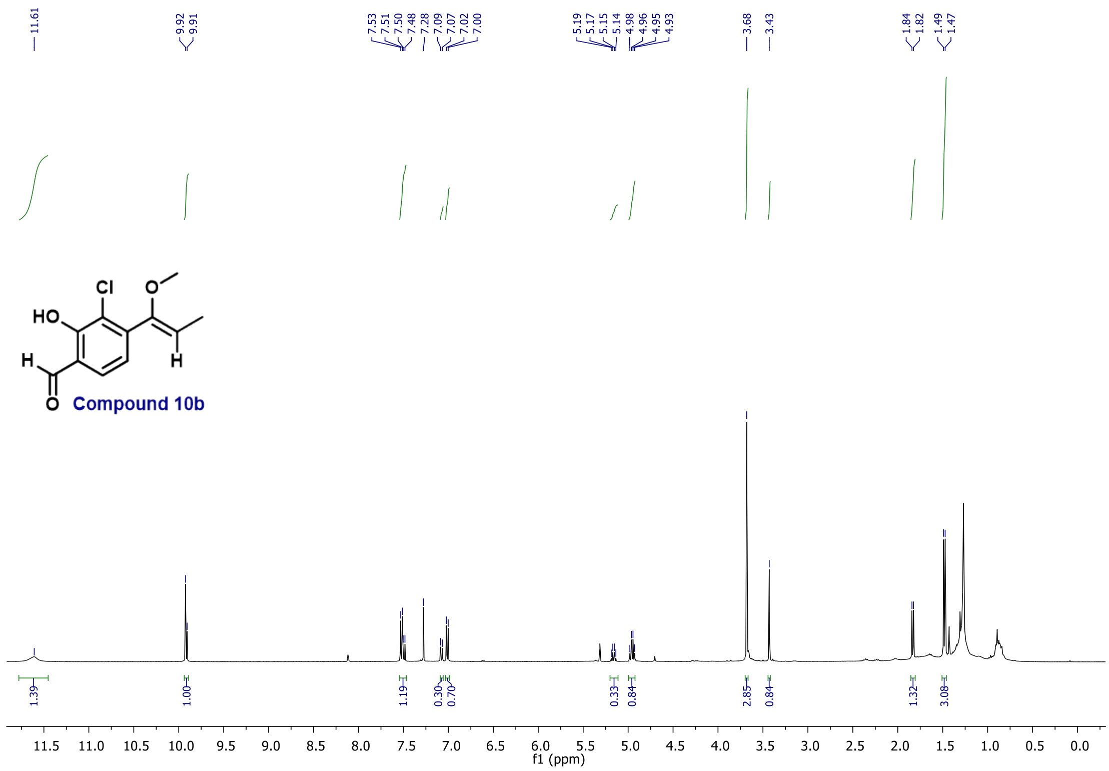

^{13}C-NMR

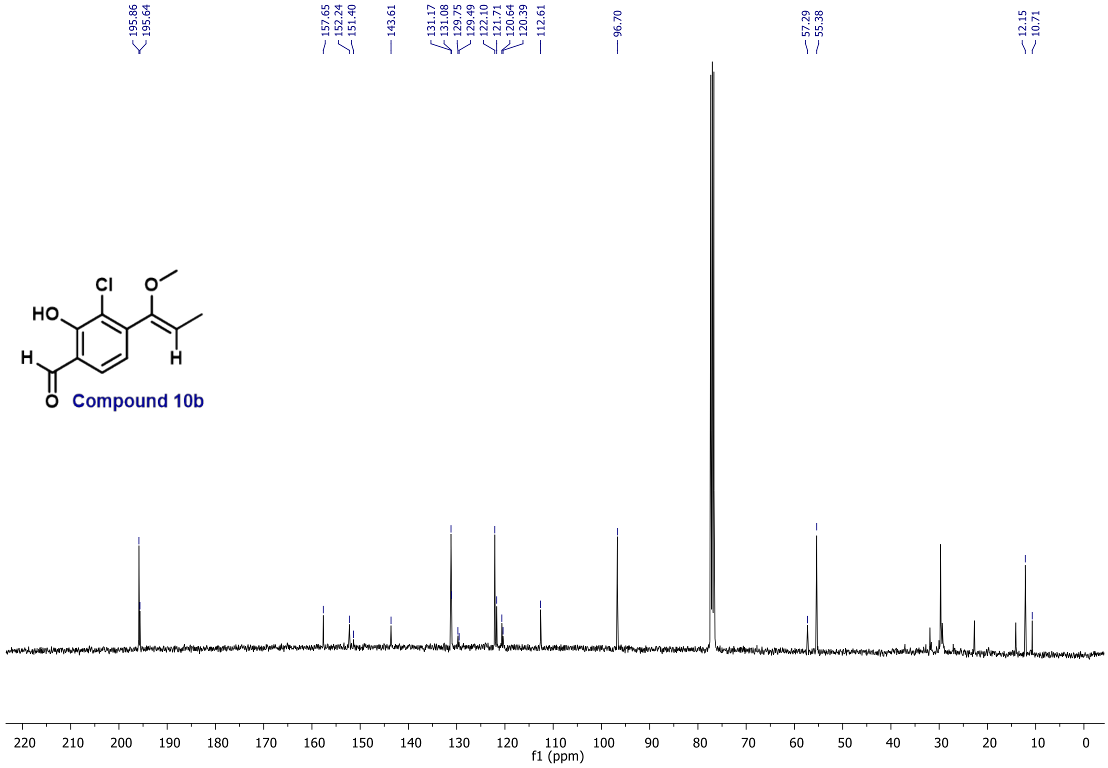

Mass spectra

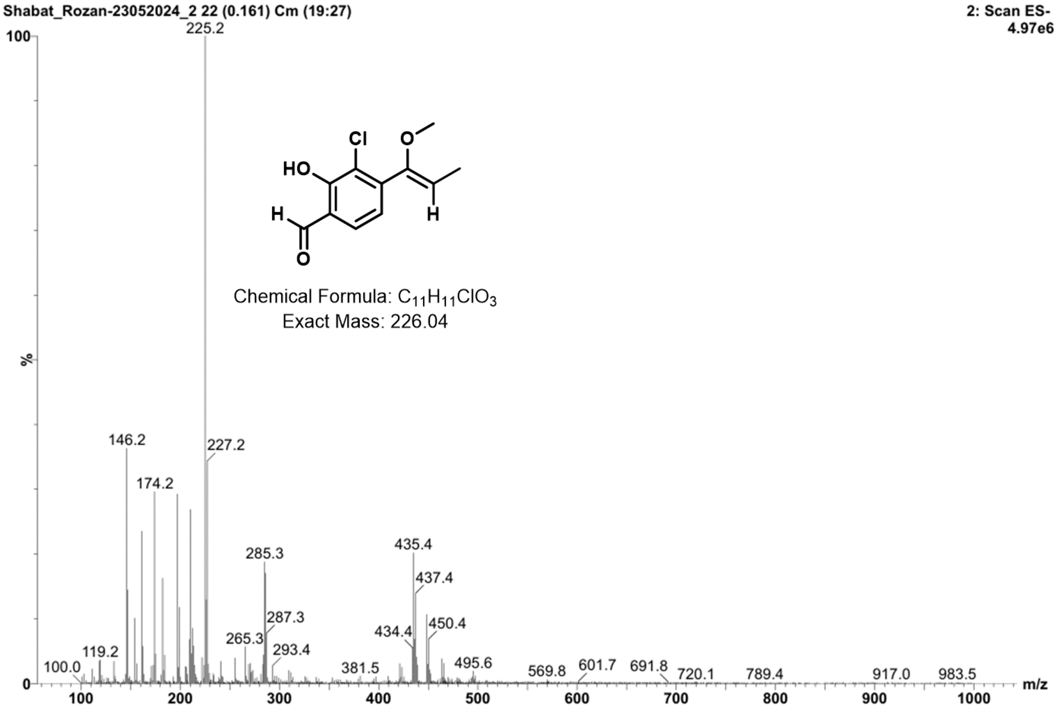

Compound 10c

^{1}H-NMR

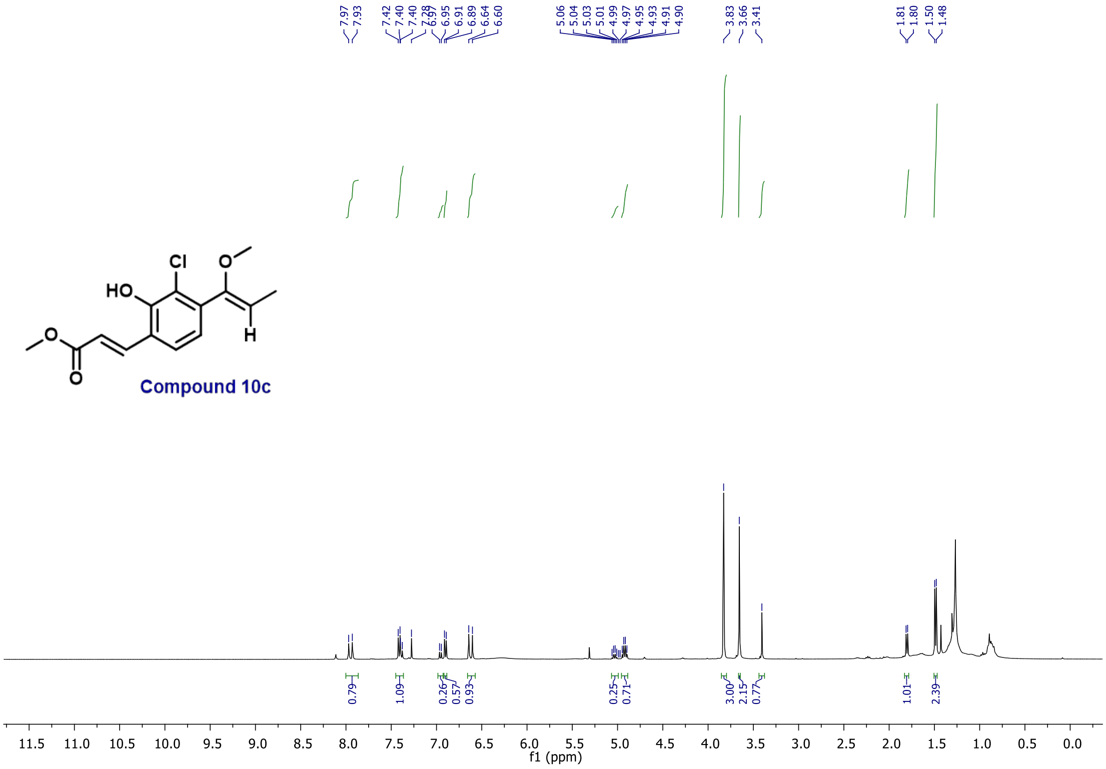

^{13}C-NMR

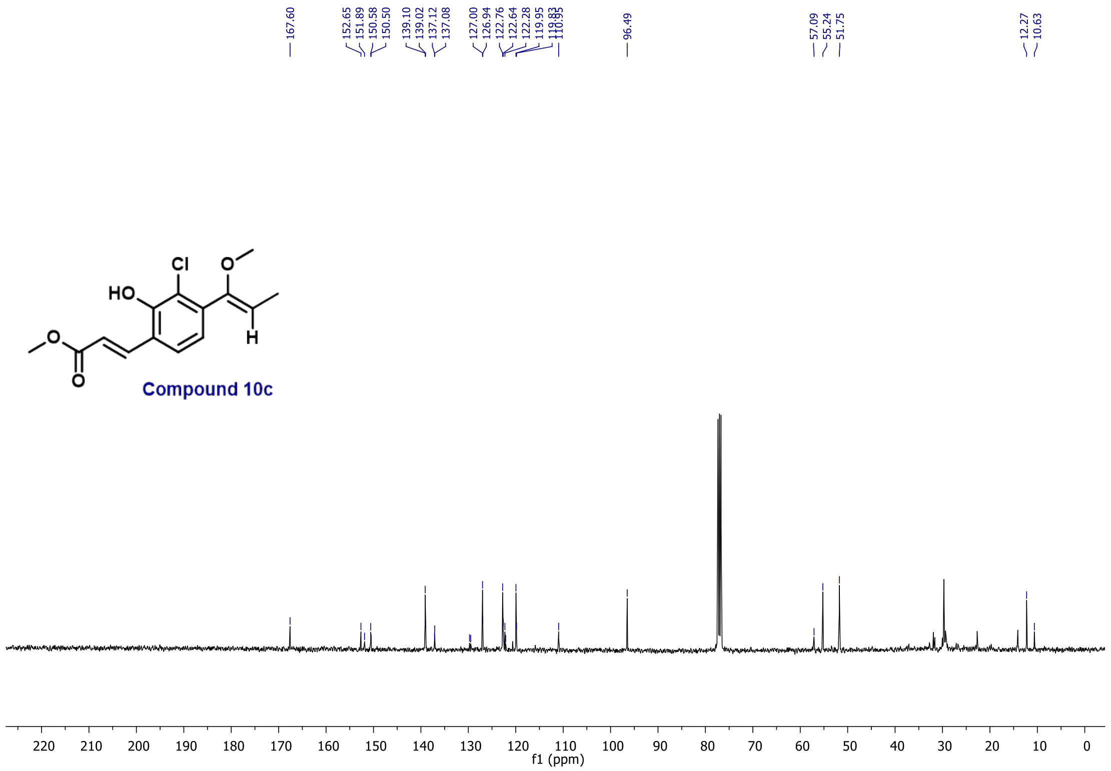

Mass spectra

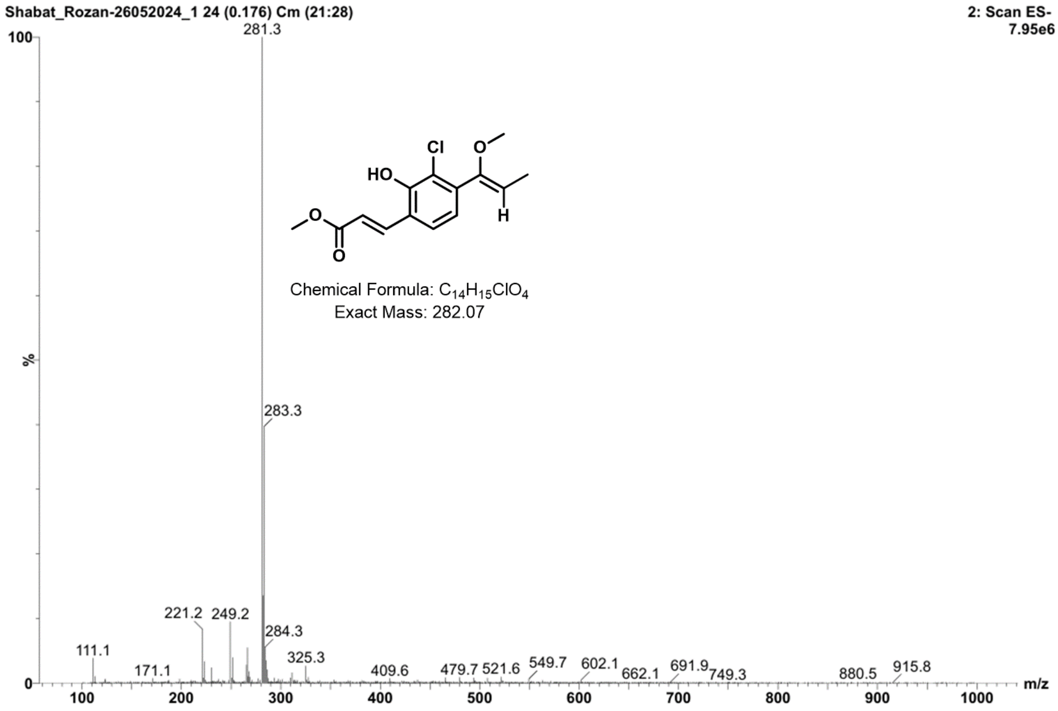

Probe 10 – SOCL-MM

^{1}H-NMR

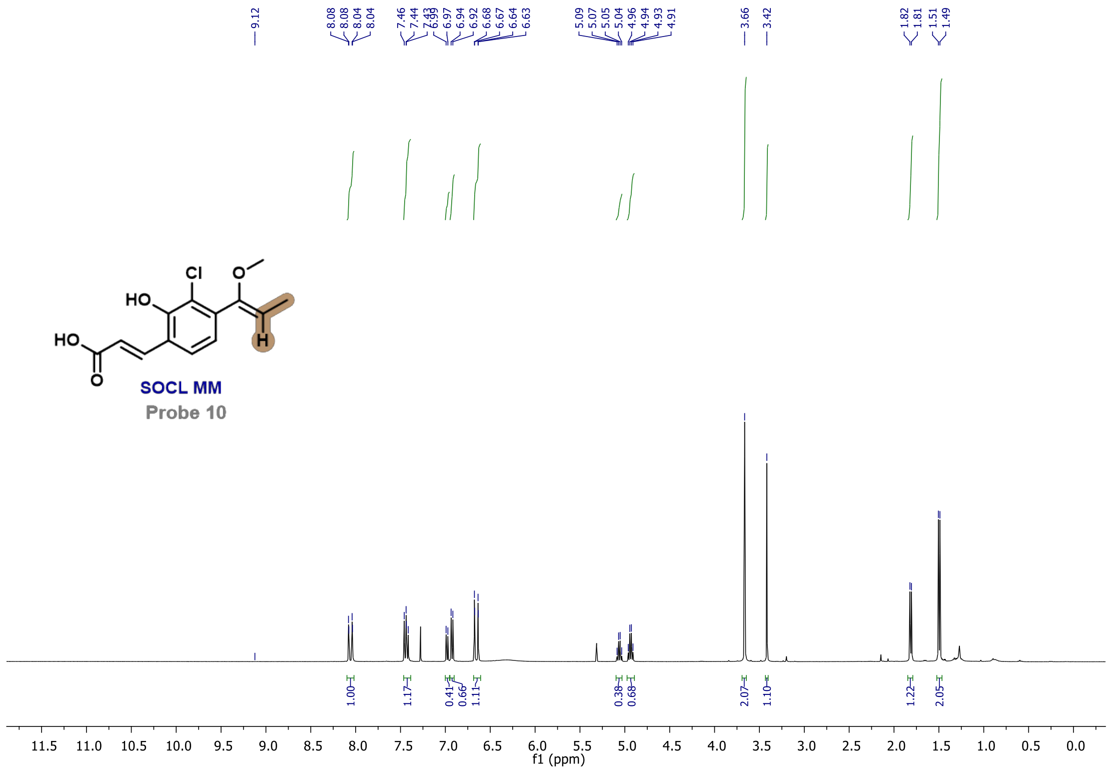

^{13}C-NMR

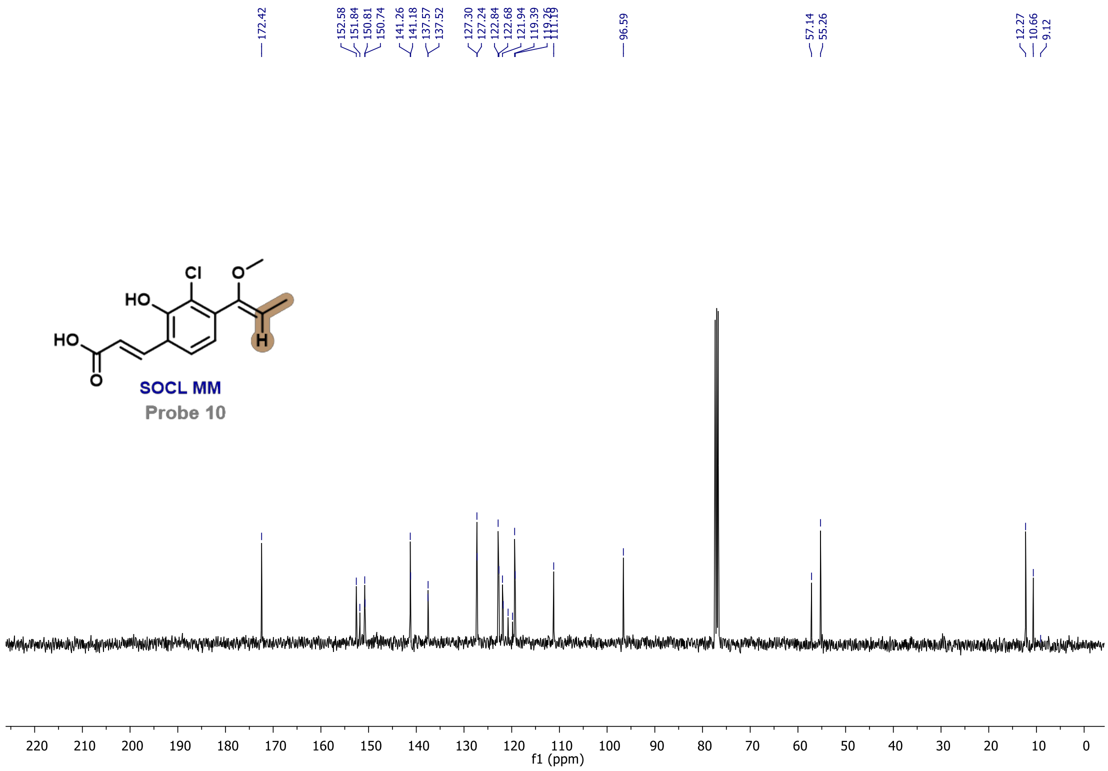

Mass spectra

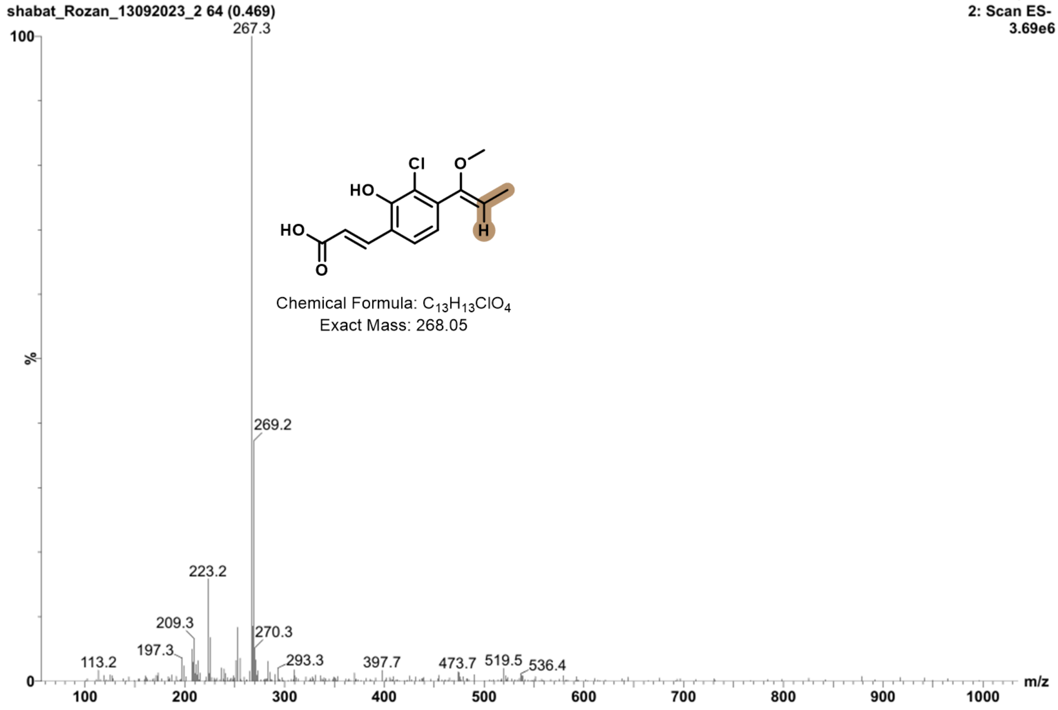

‘Ene’-product

^{1}H-NMR

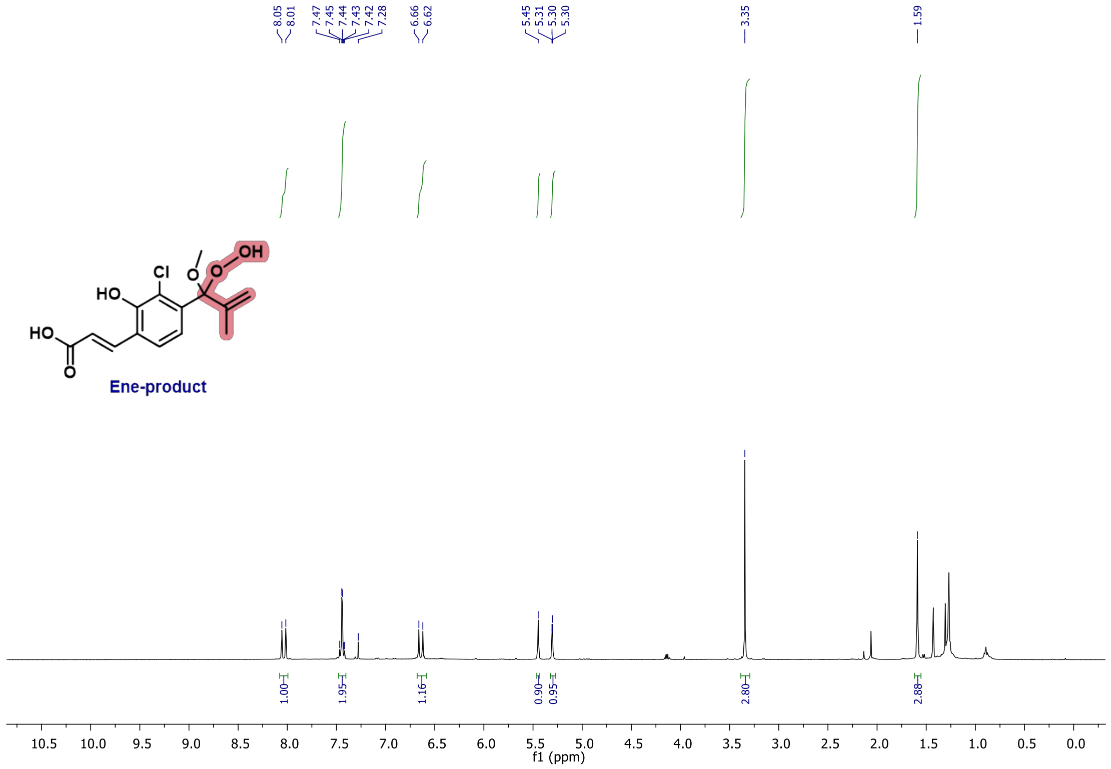

^{13}C-NMR

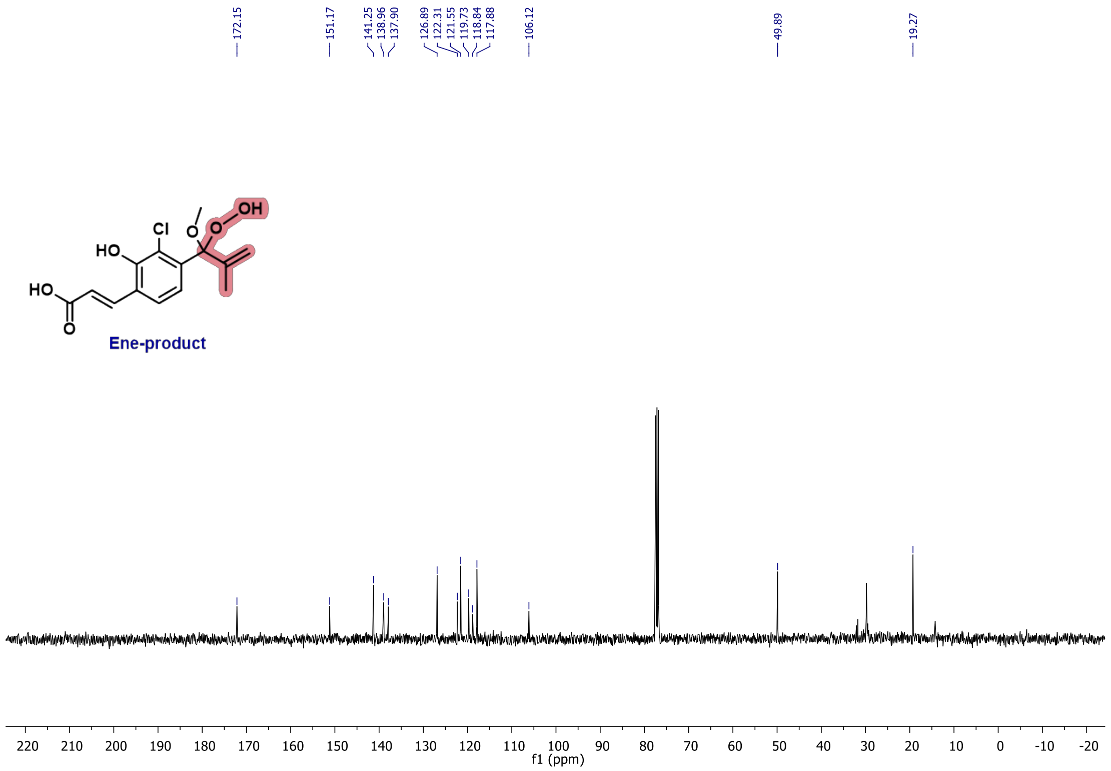

Mass spectra

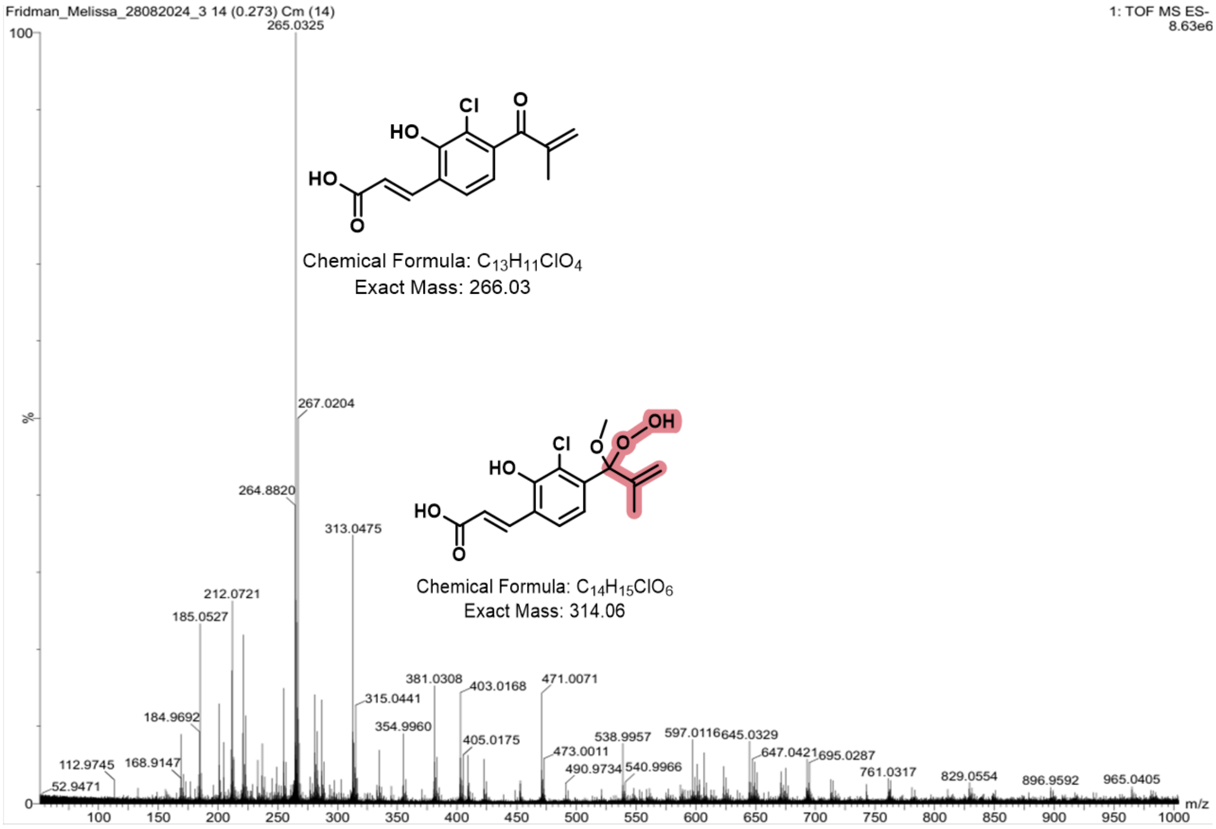

Appendix IV-HPLC of key compounds

Probe 2 – SOCL-CH

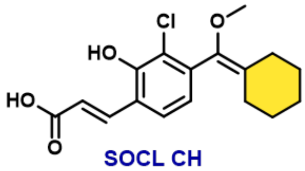

SOCL CH
Probe 2

3D HPLC spectra (30-100% ACN in water, 0.1%TFA)

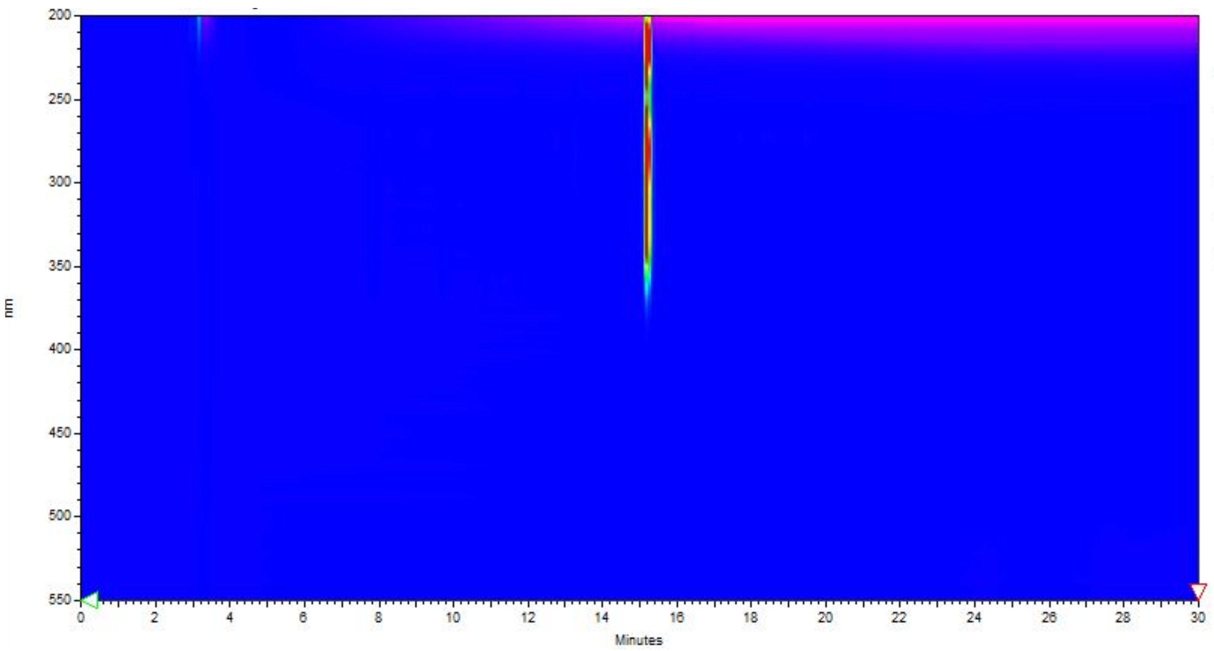

2D HPLC spectra (Absorbance measured at 350nm)

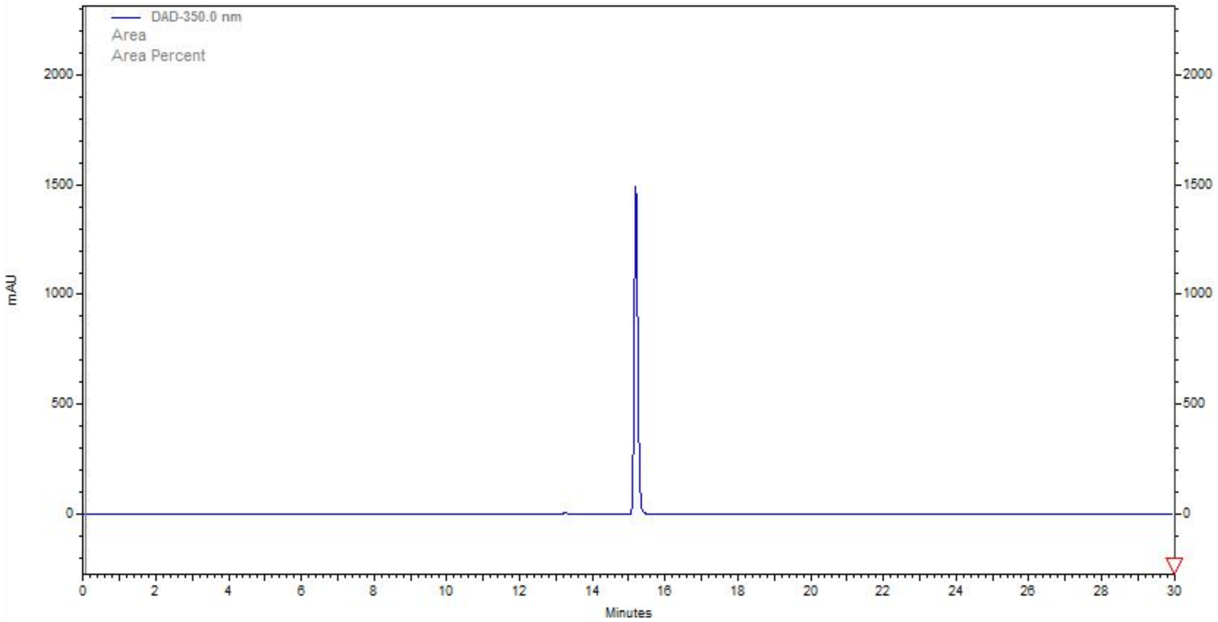

Probe 3 – SOCL-CB

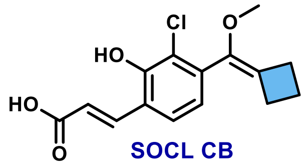

SOCL CB
Probe 3

3D HPLC spectra (30-100% ACN in water, 0.1%TFA)

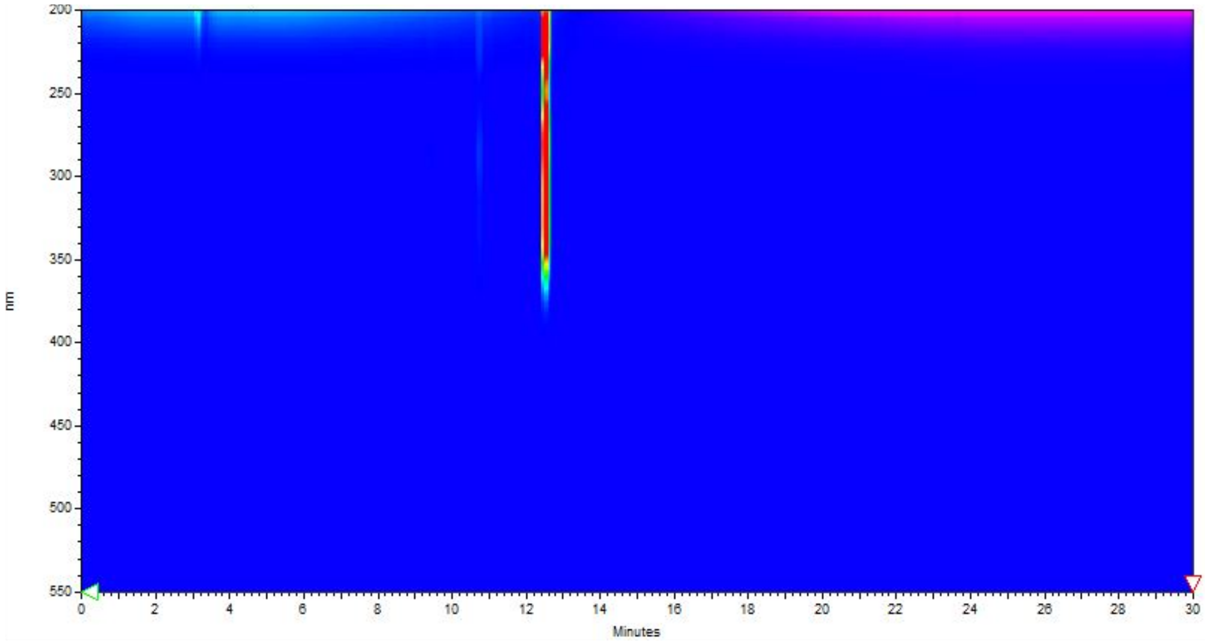

2D HPLC spectra (Absorbance measured at 350nm)

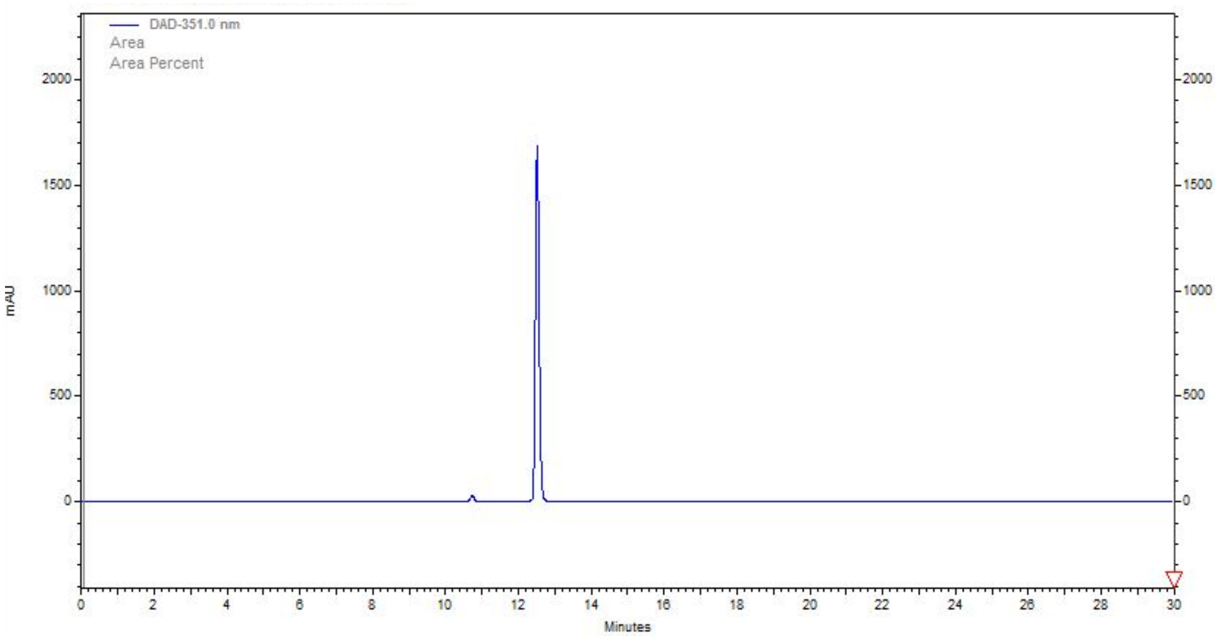

Probe 4 – SOCL-DM-CB

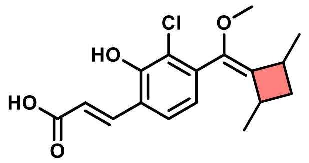

SOCL DM-CB
Probe 4

3D HPLC spectra (30-100% ACN in water, 0.1%TFA)

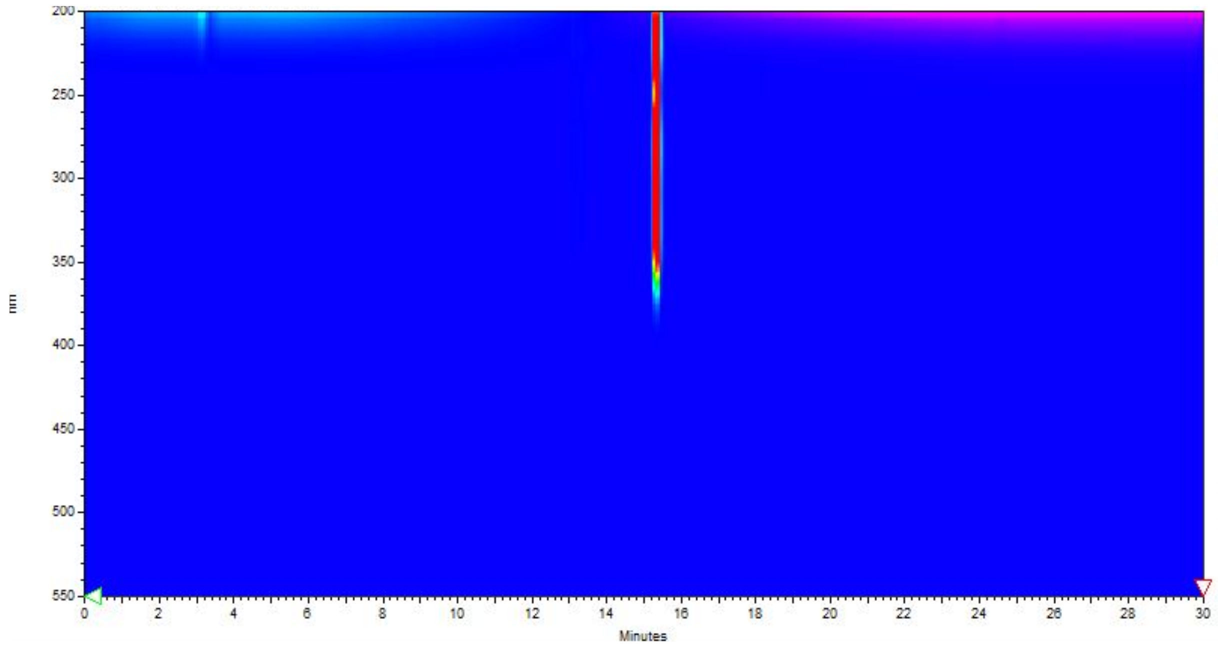

2D HPLC spectra (Absorbance measured at 350nm)

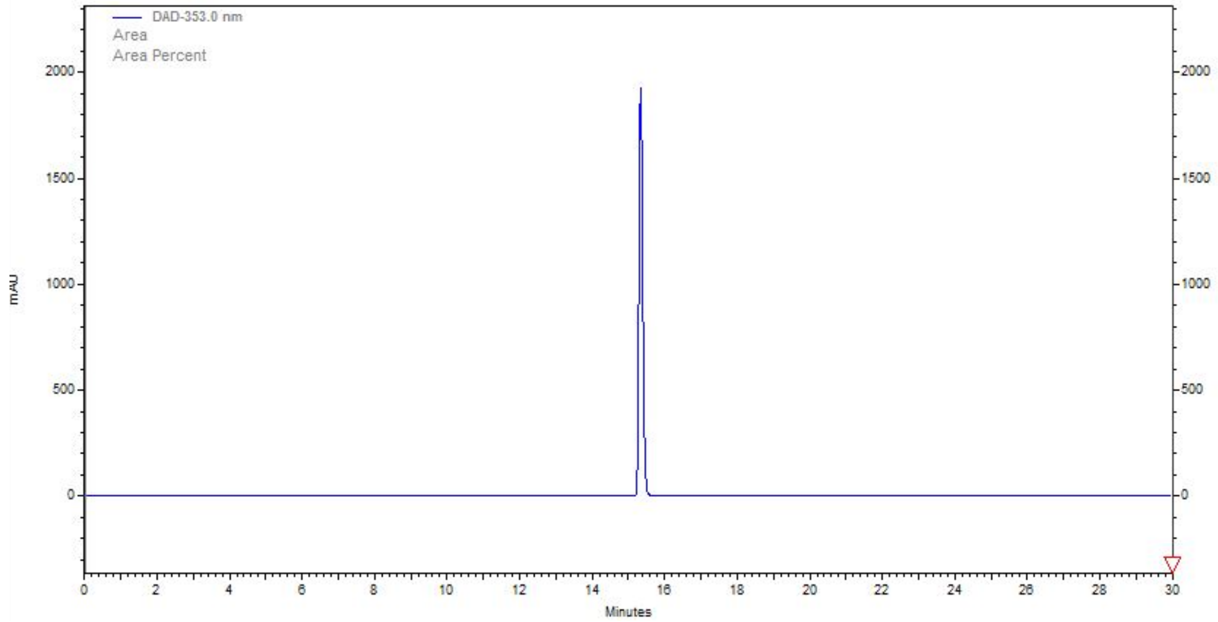

Probe 5 – SOCL-Ph-CB

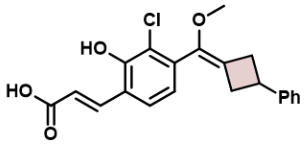

SOCL Ph-CB
Probe 5

3D HPLC spectra (30-100% ACN in water, 0.1%TFA)

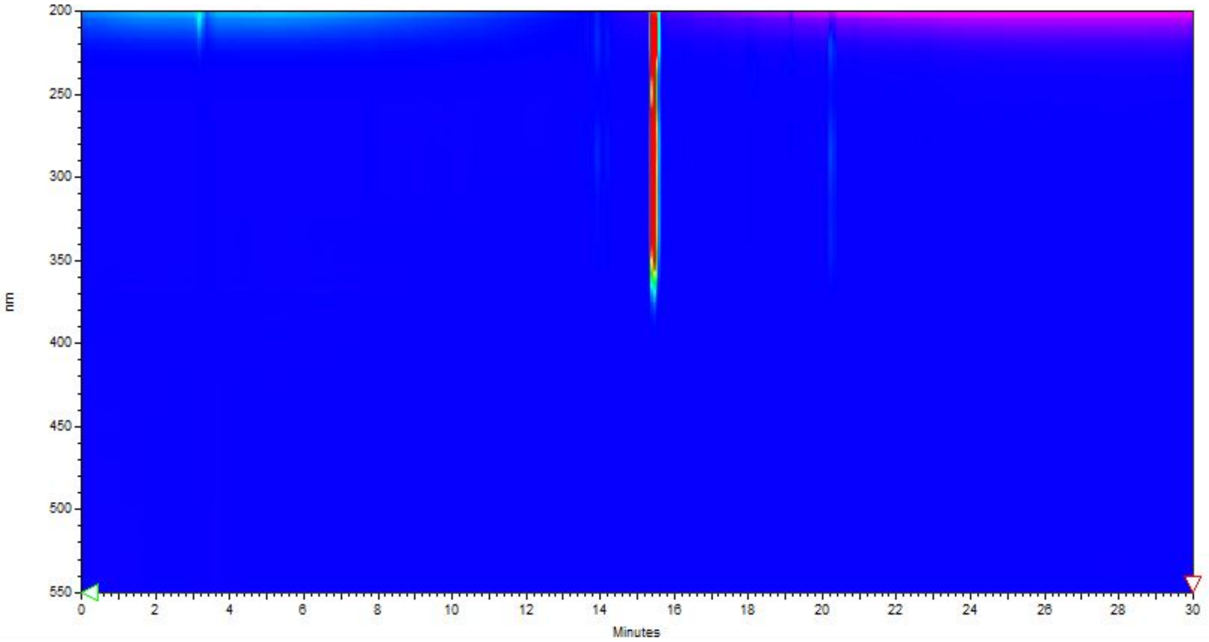

2D HPLC spectra (Absorbance measured at 370nm)

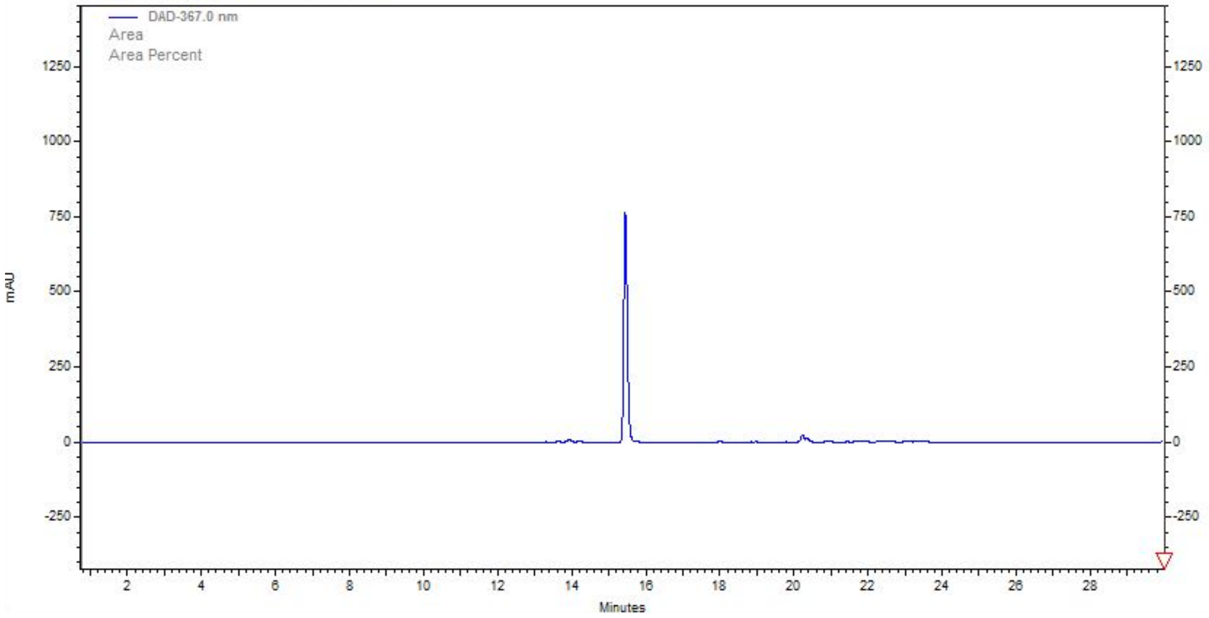

Probe 6 – SOCL-OBn-CB

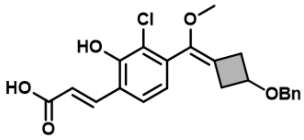

SOCL OBn-CB
Probe 6

3D HPLC spectra (30-100% ACN in water, 0.1%TFA)

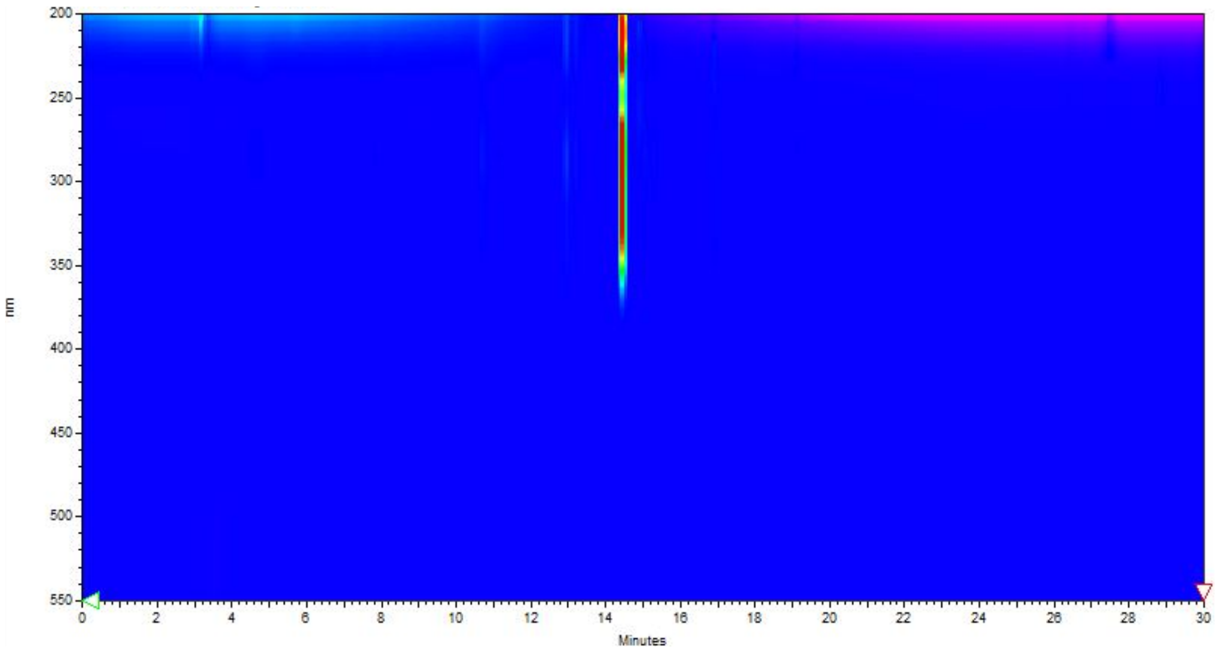

2D HPLC spectra (Absorbance measured at 350nm)

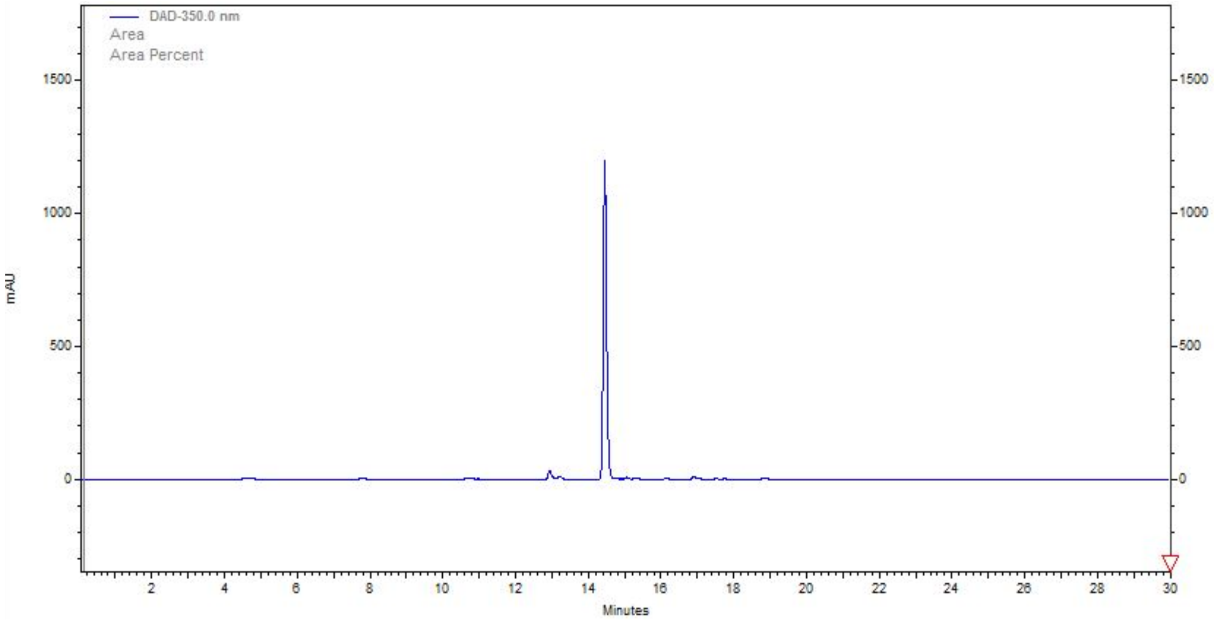

Probe 7 – SOCL-Ox

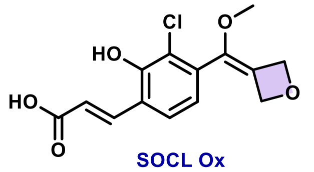

SOCL Ox
Probe 7

3D HPLC spectra (30-100% ACN in water, 0.1%TFA)

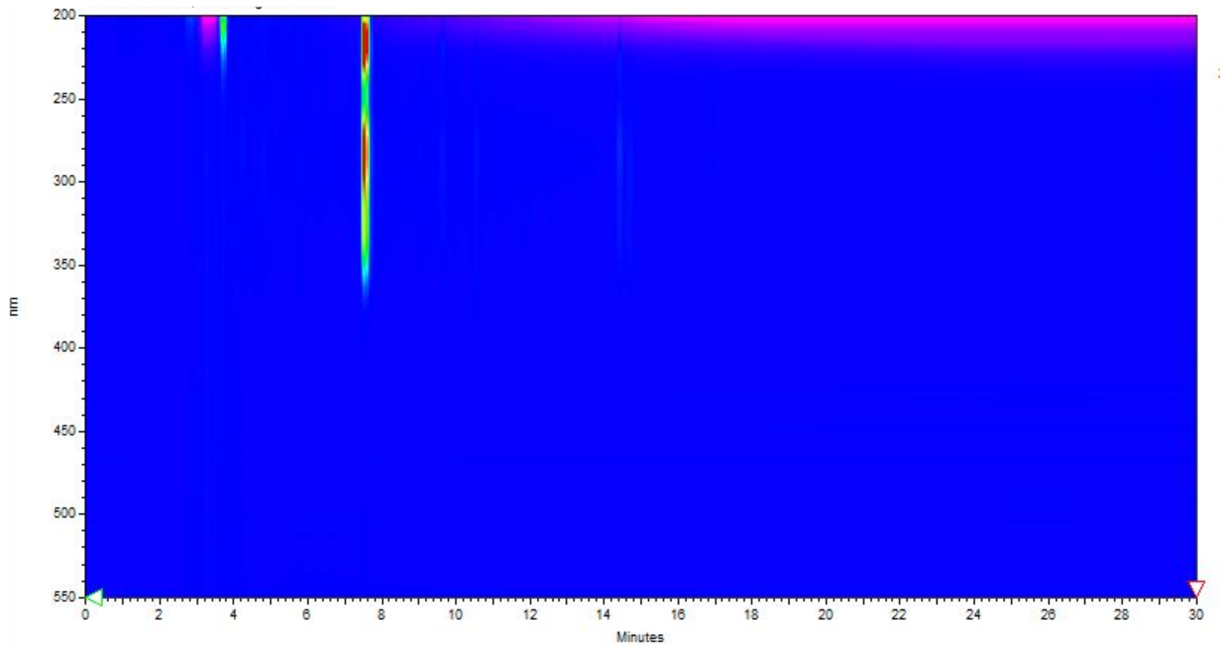

2D HPLC spectra (Absorbance measured at 350nm)

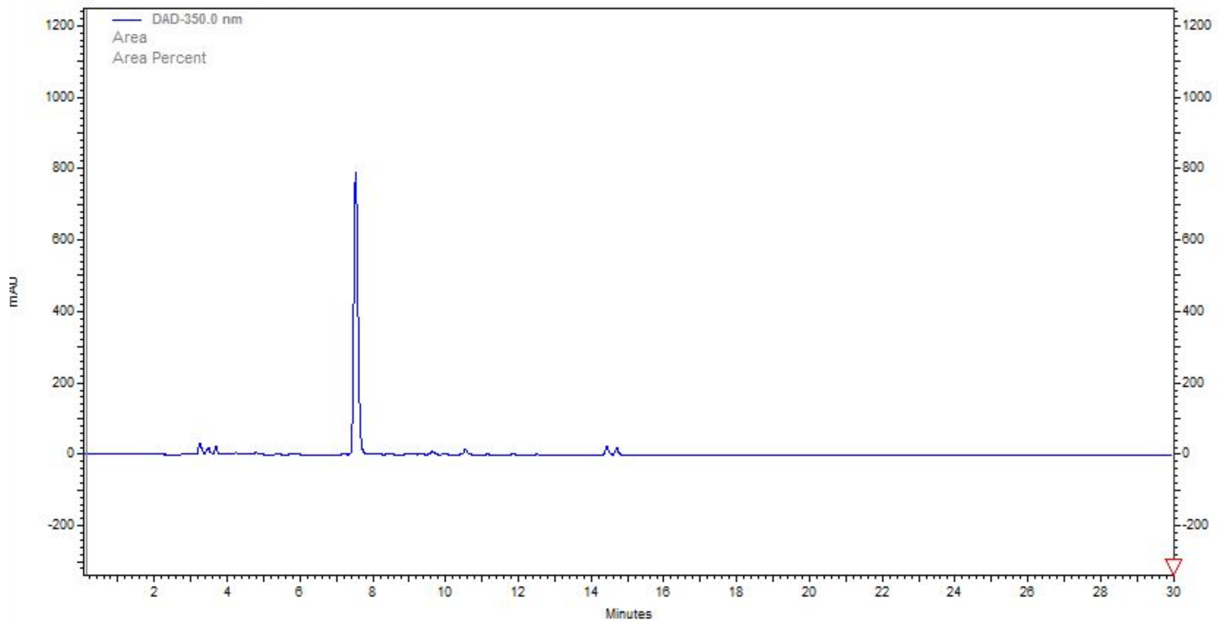

Probe 8 – SOCL-DM

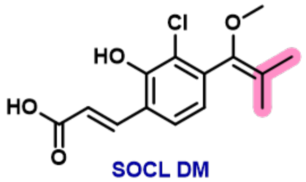

SOCL DM

Probe 8

3D HPLC spectra (30-100% ACN in water, 0.1%TFA)

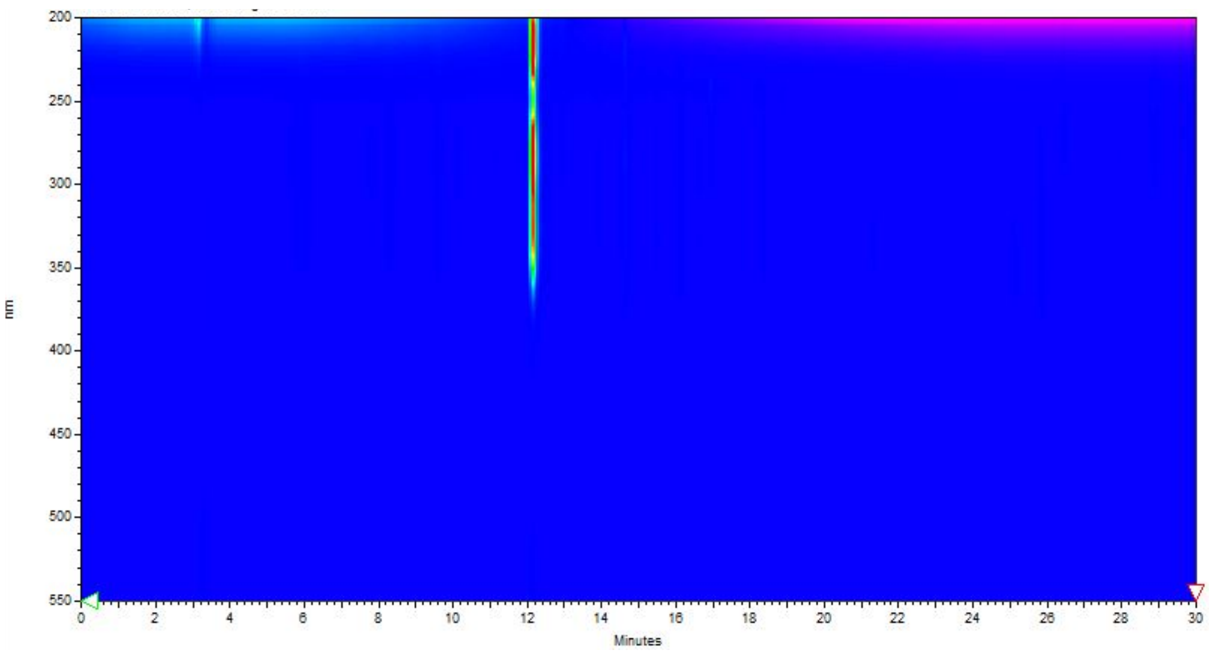

2D HPLC spectra (Absorbance measured at 350nm)

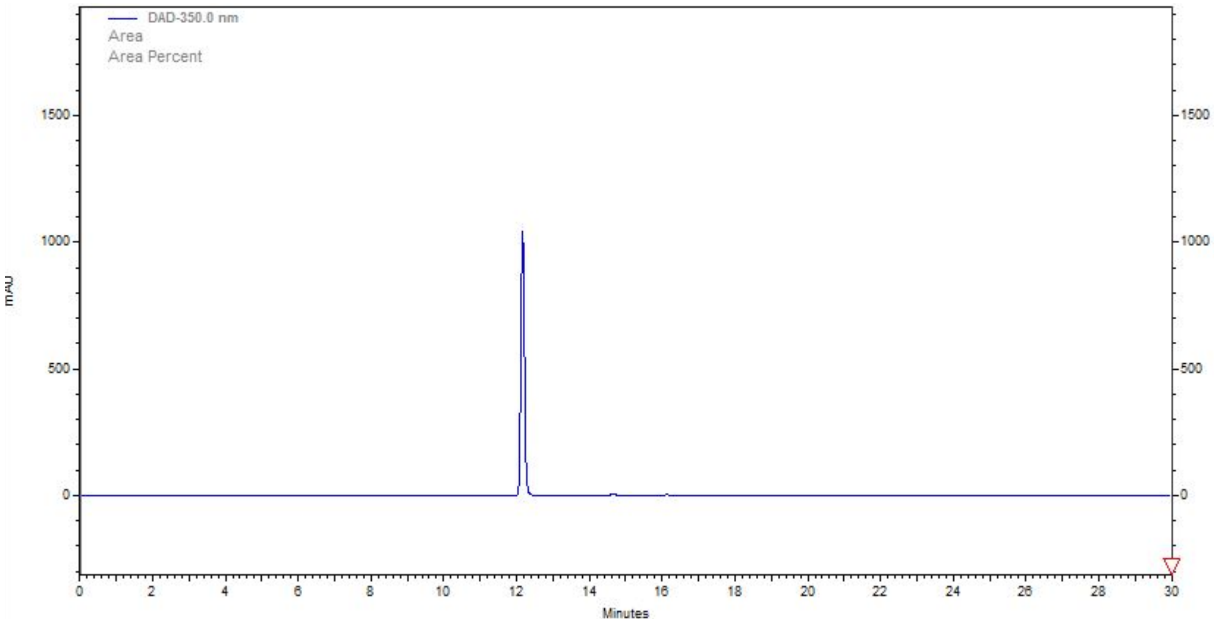

Probe 9 – SOCL-DCP

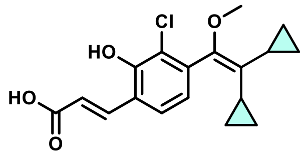

SOCL DCP
Probe 9

3D HPLC spectra (30-100% ACN in water, 0.1%TFA)

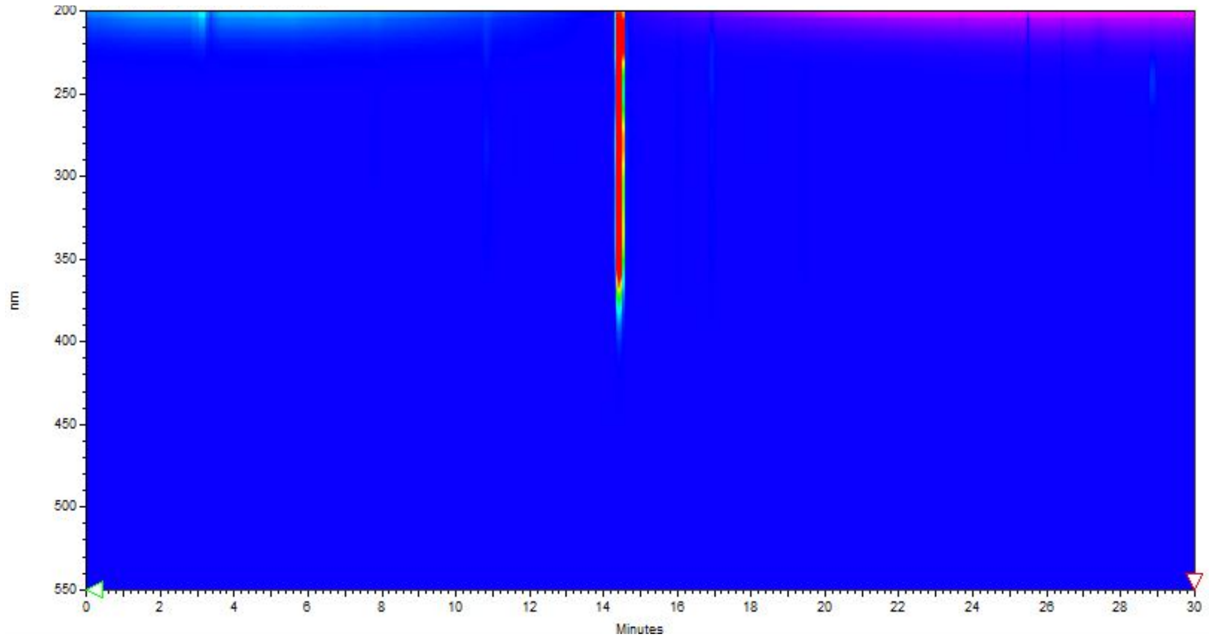

2D HPLC spectra (Absorbance measured at 365nm)

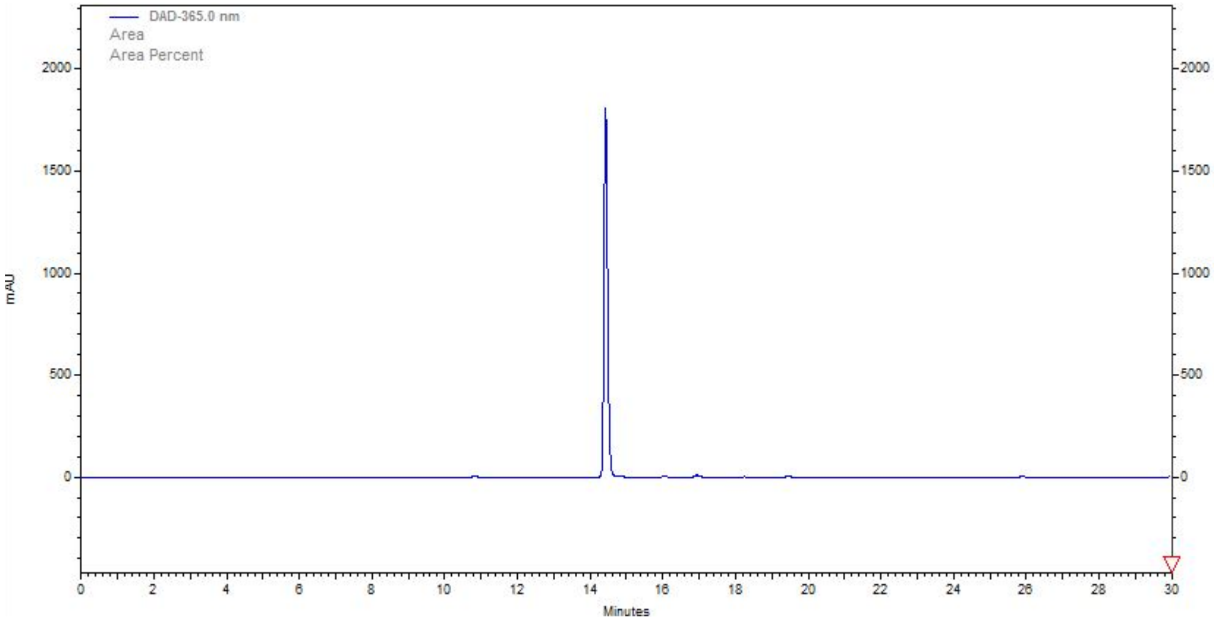

Probe 10 – SOCL-MM

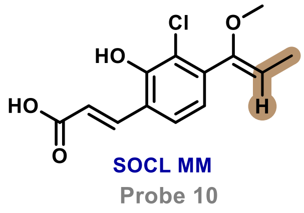

3D HPLC spectra (30-100% ACN in water, 0.1%TFA)

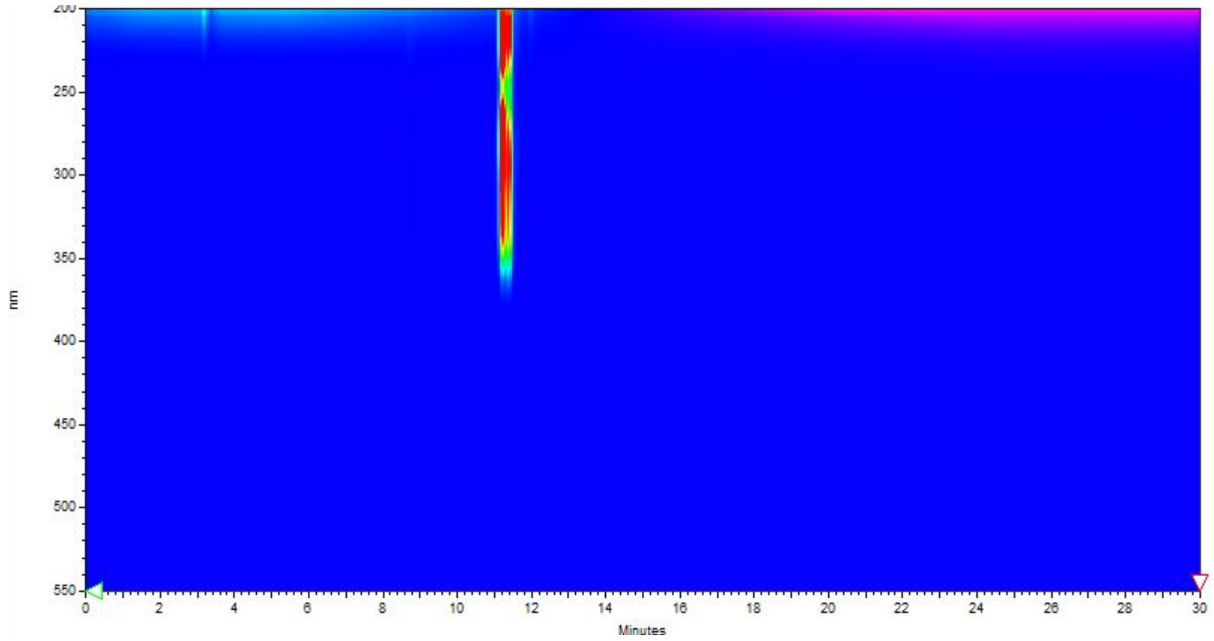

2D HPLC spectra (Absorbance measured at 333nm)

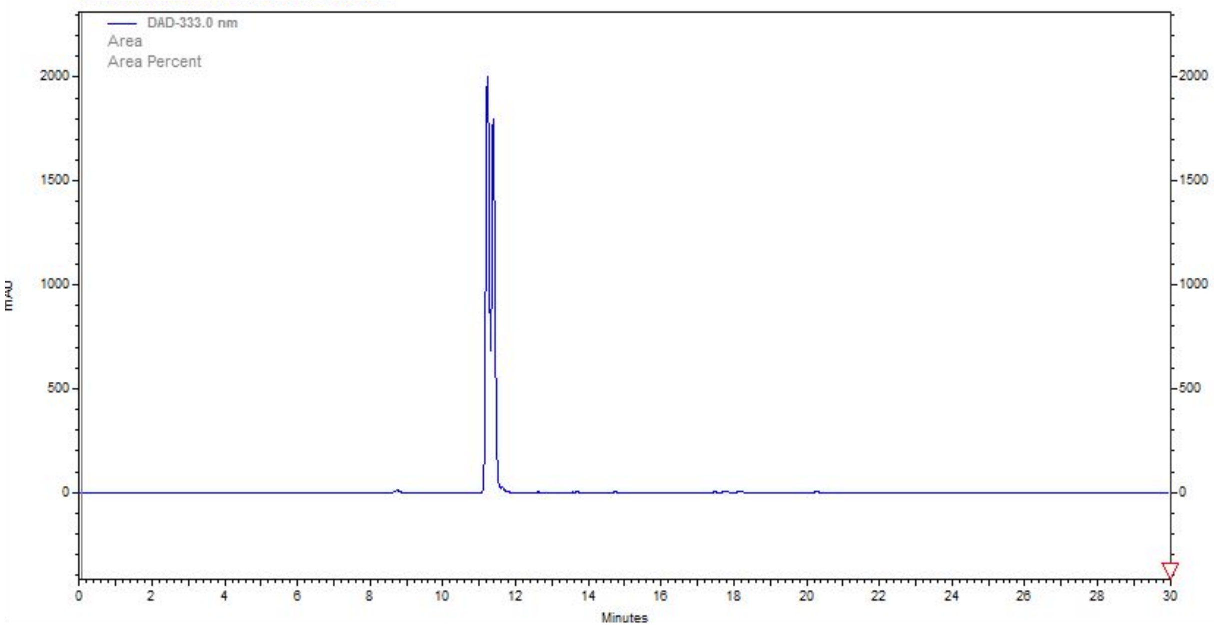

‘ene’-product

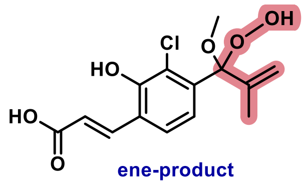

3D HPLC spectra (30-100% ACN in water, 0.1%TFA)

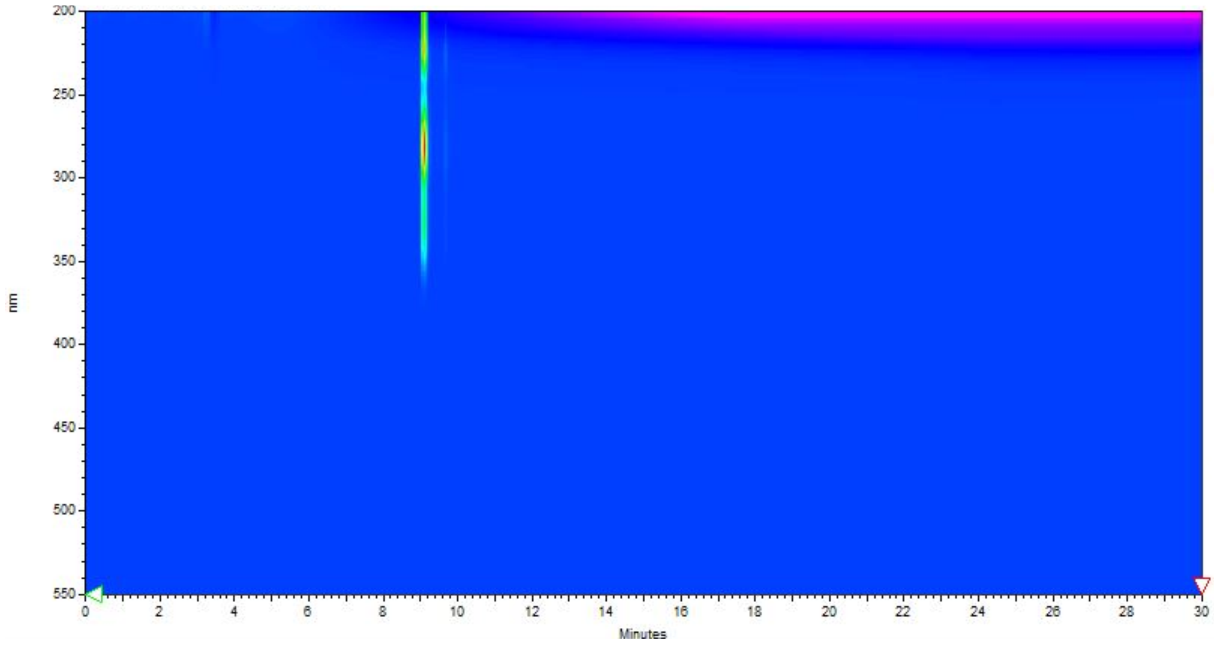

2D HPLC spectra (Absorbance measured at 333nm)

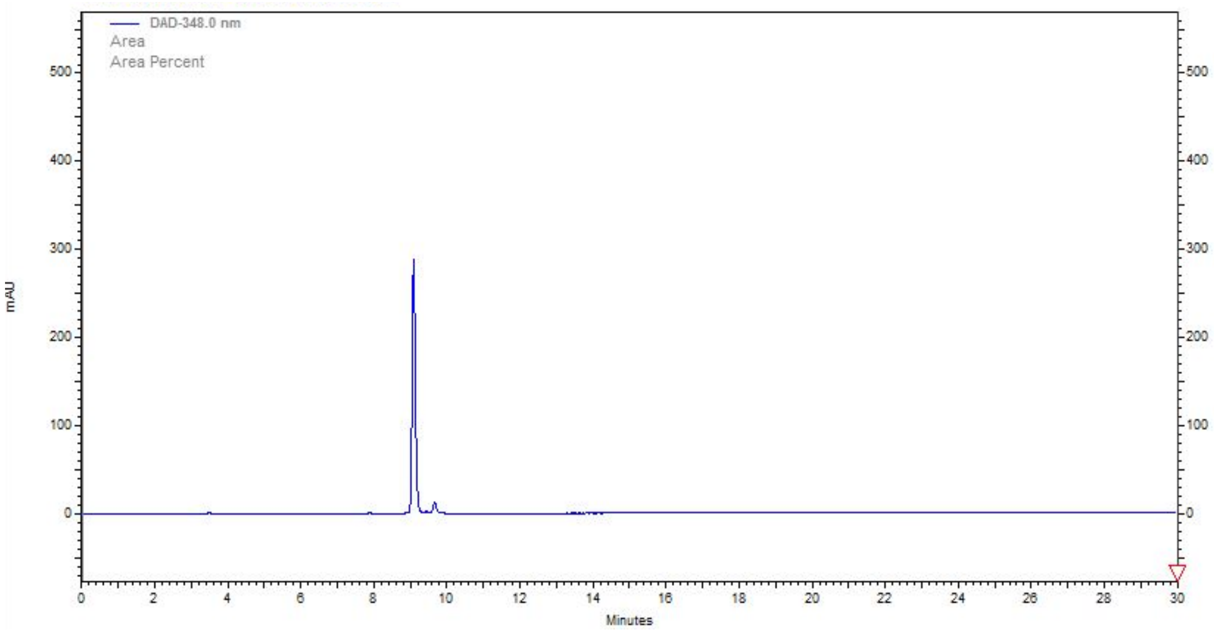

References

(1) Hananya, N.; Green, O.; Blau, R.; Satchi-Fainaro, R.; Shabat, D. A Highly Efficient Chemiluminescence Probe for the Detection of Singlet Oxygen in Living Cells. *Angew. Chem. Int. Ed.* **2017**, *56* (39), 11793-11796. DOI: 10.1002/anie.201705803.

(2) Hananya, N.; Boock, A. E.; Bauer, C. R.; Satchi-Fainaro, R.; Shabat, D. Remarkable Enhancement of Chemiluminescent Signal by Dioxetane-Fluorophore Conjugates: Turn-ON Chemiluminescence Probes with Color Modulation for Sensing and Imaging. *J. Am. Chem. Soc.* **2016**, *138* (40), 13438-13446. DOI: 10.1021/jacs.6b09173.

(3) David, M.; Leirikh, T.; Shelef, O.; Gutkin, S.; Kopp, T.; Zhou, Q.; Ma, P.; Fridman, M.; Houk, K. N.; Shabat, D. Chemiexcitation Acceleration of 1,2-Dioxetanes by Spiro-Fused Six-Member Rings with Electron-Withdrawing Motifs. *Angew. Chem. Int. Ed.* **2024**, *63* (46), e202410057. DOI: 10.1002/anie.202410057.

(4) Tannous, R.; Shelef, O.; Gutkin, S.; David, M.; Leirikh, T.; Ge, L.; Jaber, Q.; Zhou, Q. Y.; Ma, P. C.; Fridman, M.; et al. Spirostrain-Accelerated Chemiexcitation of Dioxetanes Yields Unprecedented Detection Sensitivity in Chemiluminescence Bioassays. *ACS Cent. Sci.* **2023**, *10* (1), 28-42. DOI: 10.1021/acscentsci.3c01141.

(5) Yang, M.; Zhang, J.; Shabat, D.; Fan, J.; Peng, X. Near-Infrared Chemiluminescent Probe for Real-Time Monitoring Singlet Oxygen in Cells and Mice Model. *ACS Sens.* **2020**, *5* (10), 3158-3164. DOI: 10.1021/acssensors.0c01291.

(6) Kundu, K.; Knight, S. F.; Willett, N.; Lee, S.; Taylor, W. R.; Murthy, N. Hydrocyanines: A Class of Fluorescent Sensors That Can Image Reactive Oxygen Species in Cell Culture, Tissue, and In Vivo. *Angew. Chem. Int. Ed.* **2009**, *48* (2), 299-303. DOI: 10.1002/anie.200804851.

(7) Oushiki, D.; Kojima, H.; Terai, T.; Arita, M.; Hanaoka, K.; Urano, Y.; Nagano, T. Development and Application of a Near-Infrared Fluorescence Probe for Oxidative Stress Based on Differential Reactivity of Linked Cyanine Dyes. *J. Am. Chem. Soc.* **2010**, *132* (8), 2795-2801. DOI: 10.1021/ja910090v.

(8) Fujimori, K.; Komiyama, T.; Tabata, H.; Nojima, T.; Ishiguro, K.; Sawaki, Y.; Tatsuzawa, H.; Nakano, M. Chemiluminescence of luciferin analogs.: Part 3.: MCLA chemiluminescence with singlet oxygen generated by the retro-Diels-Alder reaction of a naphthalene endoperoxide. *Photochem. Photobiol.* **1998**, *68* (2), 143-149. DOI: 10.1111/j.1751-1097.1998.tb02481.x.

(9) Tanaka, K.; Miura, T.; Umezawa, N.; Urano, Y.; Kikuchi, K.; Higuchi, T.; Nagano, T. Rational design of fluorescein-based fluorescence probes, mechanism-based design of a maximum fluorescence probe for singlet oxygen. *J. Am. Chem. Soc.* **2001**, *123* (11), 2530-2536. DOI: 10.1021/ja0035708.

(10) Frisch, M. J., Trucks, G. W., Schlegel, H. B., Scuseria, G. E., Robb, M. A., Cheeseman, J. R., Scalmani, G., Barone, V., Mennucci, B., Petersson, G. A., Nakatsuji, H., Caricato, M., Li, X., Hratchian, H. P., Izmaylov, A. F., Bloino, J., Zheng, G., Sonnengerg, J. L., Hada, M., Ehara, M., Toyota, K., Fukuda, R., Hasegawa, J., Ishida, Nakajima, T., Honda, Y., Kitao, O., Nakai, H., Vreven, T., Montgomery Jr, J. A., Peralta, J. E., Ogliaro, F., Bearpark, M., Heyd, J. J., Brothers, E., Kudin, K. N., Staroverov, V. N., Keith, T., Kobayashi, R., Normand, J., Raghavachari, K., Rendell, A., Burant, J. C., Iyengar, S. S., Tomasi, J., Cossi, M., Rega, N., Millam, N. J., Klene, M., Knox, J. E., Cross, J. B., Bakken, V., Adamo, C., Jaramillo,

J., Gomperts, R., Stratmann, R. E., Yazyev, O., Austin, A. J., Cammi, R., Pomelli, C., Ochterski, J. W., Martin, R. L., Morokuma, K., Zakrzewski, V. G., Voth, G. A., Salvador, P., Dannenberg, J. J., Dapprich, S., Daniels, A. D., Farkas, Ö., Foresman, J. B., Ortiz, J. V., Cioslowski, J. & Fox, D. J. *Gaussian 09, Revision D.01*. Gaussian, Inc., 2010. (accessed 09.04.2025).

(11) Perdew, J. P. Density-Functional Approximation for the Correlation-Energy of the Inhomogeneous Electron-Gas. *Phys. Rev. B.* **1986**, *33* (12), 8822-8824. DOI: 10.1103/PhysRevB.33.8822.

(12) Becke, A. D. Density-Functional Exchange-Energy Approximation with Correct Asymptotic-Behavior. *Phys. Rev. A.* **1988**, *38* (6), 3098-3100. DOI: 10.1103/PhysRevA.38.3098.

(13) Weigend, F.; Ahlrichs, R. Balanced basis sets of split valence, triple zeta valence and quadruple zeta valence quality for H to Rn: Design and assessment of accuracy. *Phys. Chem. Chem. Phys.* **2005**, *7* (18), 3297-3305. DOI: 10.1039/b508541a.

(14) Grimme, S.; Antony, J.; Ehrlich, S.; Krieg, H. A consistent and accurate ab initio parametrization of density functional dispersion correction (DFT-D) for the 94 elements H-Pu. *J. Chem. Phys.* **2010**, *132* (15). DOI: 10.1063/1.3382344.

(15) Grimme, S.; Ehrlich, S.; Goerigk, L. Effect of the Damping Function in Dispersion Corrected Density Functional Theory. *J. Comput. Chem.* **2011**, *32* (7), 1456-1465. DOI: 10.1002/jcc.21759.

(16) Hanwell, M. D.; Curtis, D. E.; Lonie, D. C.; Vandermeersch, T.; Zurek, E.; Hutchison, G. R. Avogadro: an advanced semantic chemical editor, visualization, and analysis platform. *J. Cheminformatics* **2012**, *4*. DOI: 10.1186/1758-2946-4-17.

(17) Zhang, Y.; Yan, C.; Wang, C.; Guo, Z.; Liu, X.; Zhu, W. H. A Sequential Dual-Lock Strategy for Photoactivatable Chemiluminescent Probes Enabling Bright Duplex Optical Imaging. *Angew. Chem. Int. Ed.* **2020**, *59* (23), 9059-9066. DOI: 10.1002/anie.202000165.
